# Copper-catalyzed defluorinative arylboration of vinylarenes with polyfluoroarenes†

**DOI:** 10.1039/d2sc06472c

**Published:** 2023-02-06

**Authors:** Fu-Peng Wu, Xing-Wei Gu, Hui-Qing Geng, Xiao-Feng Wu

**Affiliations:** a Leibniz-Institut für Katalyse e.V. Albert-Einstein-Straße 29a 18059 Rostock Germany xiao-feng.wu@catalysis.de; b Dalian National Laboratory for Clean Energy, Dalian Institute of Chemical Physics, Chinese Academy of Sciences 116023 Dalian Liaoning China xwu2020@dicp.ac.cn

## Abstract

An unprecedented but challenging defluorinative arylboration has been achieved. Enabled by a copper catalyst, an interesting procedure on defluorinative arylboration of styrenes has been established. With polyfluoroarenes as the substrates, this methodology offers flexible and facile access to provide a diverse assortment of products under mild reaction conditions. In addition, by using a chiral phosphine ligand, an enantioselective defluorinative arylboration was also realized, affording a set of chiral products with unprecedented levels of enantioselectivity.

## Abstract 2

Catalyzed by CuCl and DPEphos, various β-polyfluoroaryl boronates were obtained from styrenes and polyfluoroarenes *via* defluorinative arylboration. With the use of a chiral ligand, the first example of chiral version was realized.

## Graphical abstract

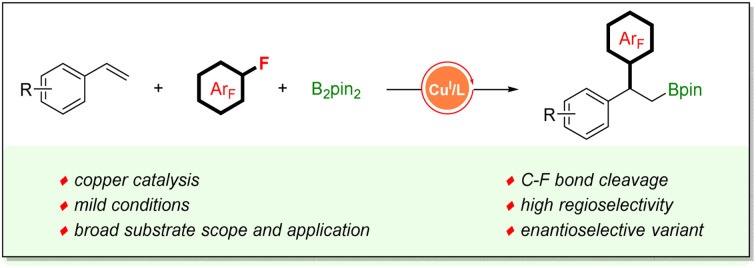

Polyfluorinated aromatics are highly prized molecules in pharmaceutical chemistry^1^ and materials science^2^ due to their special properties such as metabolic stability and intermolecular π–π_F_-interactions (Scheme 1).^3^ Two new polyfluorinated aromatic-containing drugs were approved by the FDA in 2021.^4^ During the past decades, the dominant fluorination approaches were the introduction of a single fluorine atom into an aromatic ring *via* C–H activation^5^ or C–X substitution.^6^ While these methods are more applicable for the preparation of mono-fluorinated arenes, they are unsuitable for polyfluoroarenes due to the requirement of the preinstallation of multiple functional groups or unique directing groups. Actually, polyfluorinated aromatics were produced by the substitution of a fluorine atom (defluorinative functionalization) of easily available simple polyfluoroarenes, including nucleophilic aromatic substitution (S_N_Ar), reaction *via* a radical polyfluoroaryl intermediate, and metal-catalyzed C–F bond functionalization (Scheme 2a).^7^ For example, a simple and robust hydrodefluorination of polyfluoroarenes (HDF) has been well established.^8^ In addition, defluorinative borylation of polyfluoroarenes has also been realized in recent years.^9^

**Scheme 1:**
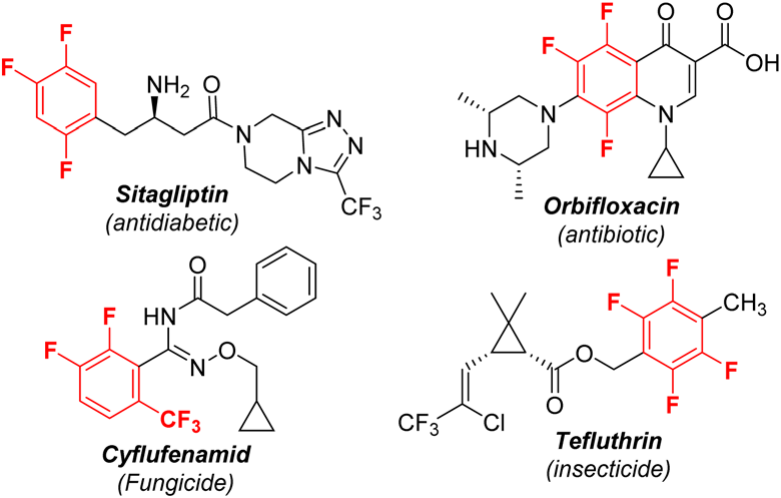
Selected examples of important poylfluoroaryl compounds.

**Scheme 2:**
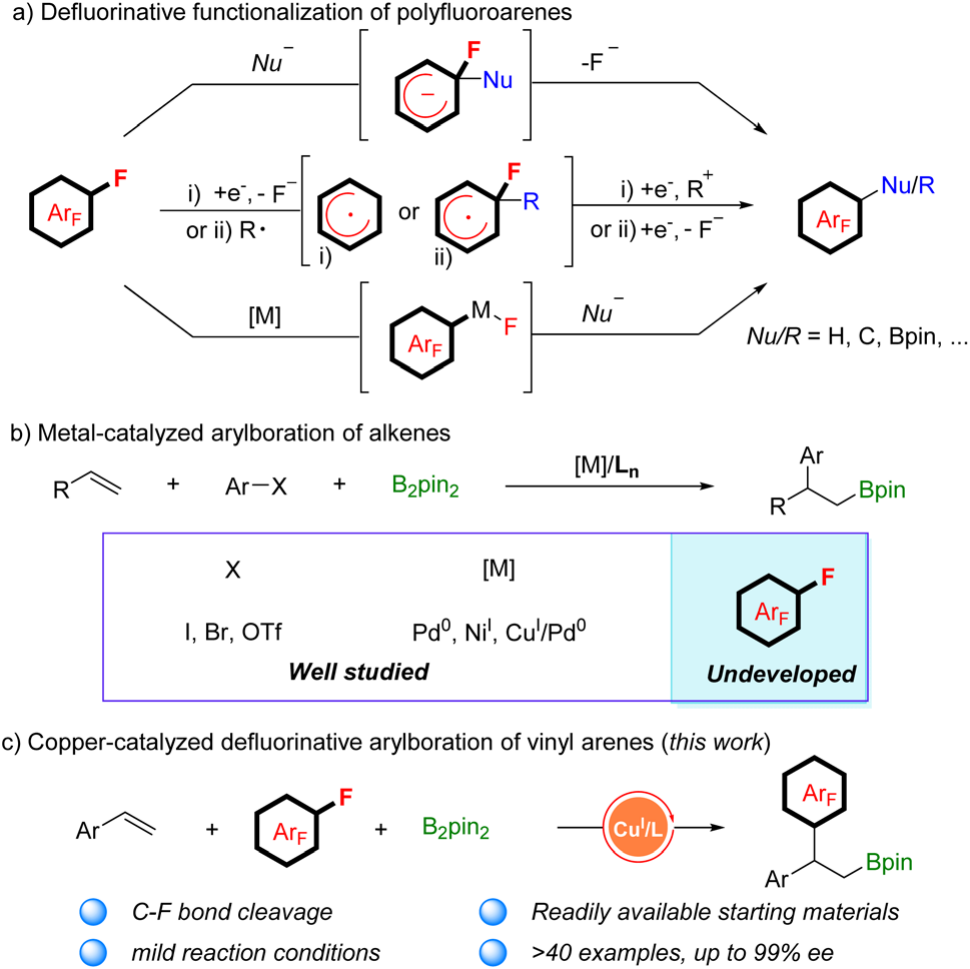
Defluorinative functionalization of polyfluoroarenes.

Among the synthetic transformations, carbon–carbon cross-coupling of simple polyfluoroarenes is one of the most widespread applications of defluorinative functionalization, offering a general approach for synthesizing more complex polyfluoroaryl-containing products. However, the reactions are usually limited to carbon nucleophiles and using stoichiometric amounts of organometallic reagents such as alkyl- or arylmetallic reagents (metal: lithium, magnesium, and zinc), which is a great challenge for complex molecules because of the poor compatibility with diverse functional groups.^10^ To avoid the use of stoichiometric organometallic regents, Ritter's group reported a photocatalytic decarbonylative polyfluoroarylation of aliphatic carboxylic acid *via* radical addition to polyfluoroarenes and then elimination of a fluoride.^11^ Additionally, several examples of transition-metal-catalyzed carbon–carbon cross-coupling of polyfluoroarenes have also been disclosed in the absence of organometallic reagents.^12^ Radius and co-workers^13^ described a nickel-catalyzed C–F bond arylation of polyfluoroarenes with aryl boronic acid as the nucleophilic reagent in 2006. Subsequently, such reactions were extended to palladium based catalytic systems.^14^ Recently, Xiong and co-workers reported a defluorinative hydroarylation of alkenes with polyfluoroarenes by *in situ* catalyst formation.^15^

Additionally, the metal-catalyzed arylboration of alkenes has recently emerged as a general method to access diverse alkyl boronic esters with good regioselectivity.^16^ In 2014, Semba and Nakao^17^ reported their results on arylboration of alkenes by cooperative Pd/Cu catalysis. The reaction was generally initiated by alkene migratory insertion into a Cu–Bpin species, which leads to a β-boryl alkylcopper(i) intermediate. The intermediate reacts with an aryl electrophile under the assistance of palladium catalysis to give 1,2-arylboration products. Subsequently, great advances have been achieved in alkene carboboration with Pd^0^ or Ni^I^ catalysis by research groups of Brown,^18^ Engle,^19^ Yin,^20^ and Liao.^21^ However, most of the aryl electrophile leaving groups are restricted to I, Br, or OTf (Scheme 2b). The arylboration of aryl fluorides with alkenes has not been realized because of the strength of the C–F bond. Inspired by their creative achievements and our continual interest in borylation of alkenes,^22^ we attempted to develop a new catalyst system for the simultaneous borylation and defluorinative C–C cross coupling. We speculated that the β-boryl alkylcopper(i) complex, which is generated *in situ*, might attack the C–F bond of polyfluoroarenes through the S_N_Ar mechanism, bypassing the metal oxidative addition step. In addition, stereospecific transformation of the chiral β-boryl alkylcopper(i) complex could be realized in the presence of a chiral ligand.^23^ Herein, we report a copper-catalyzed defluorinative arylboration of vinylarene with polyfluoroarenes to access β-polyfluoroaryl boronates with excellent reactivity and regioselectivity. With slight modifications of the reaction conditions, an enantioselective defluorinative arylboration of alkenes was developed as well, producing a set of chiral β-polyfluoroaryl boronates with unprecedented levels of enantioselectivity (Scheme 2c).

To study this defluorinative arylboration transformation, styrene 1a, pentafluorobenzonitrile 2a, and B_2_pin_2_ were selected as the model substrates. As shown in Table 1, by using low valent CuCl as the catalyst, xantphos L1 as the ligand, and NaO^*t*^Bu as the base at 60 °C for 16 h, *para*-defluorinative β-polyfluoroaryl boronates 3a were successfully obtained in 35% yield and a small amount of *ortho*-defluorinative product was detected (Table 1, entry 1). In the testing of solvents, non-polar solvent *n*-heptane decreased the reaction efficiency and regioselectivity (Table 1, entry 2). When the reaction was performed in coordinative solvents such as 1,4-dioxane or THF, product 3a was obtained in moderate yield with excellent regioselectivity. Then bidentate phosphine ligands were examined, and smaller bite angle DPEphos L3 delivered the desired product 3a in 64% yield, while side product β-boryl styrene was detected (Table 1, entry 7). Increased selectivity was observed by decreasing the temperature (Table 1, entry 10). Finally, the yield can be further improved to 90% by increasing the amount of base applied (Table 1, entry 11).

**Table 1:** Optimization of the reaction conditions^a^

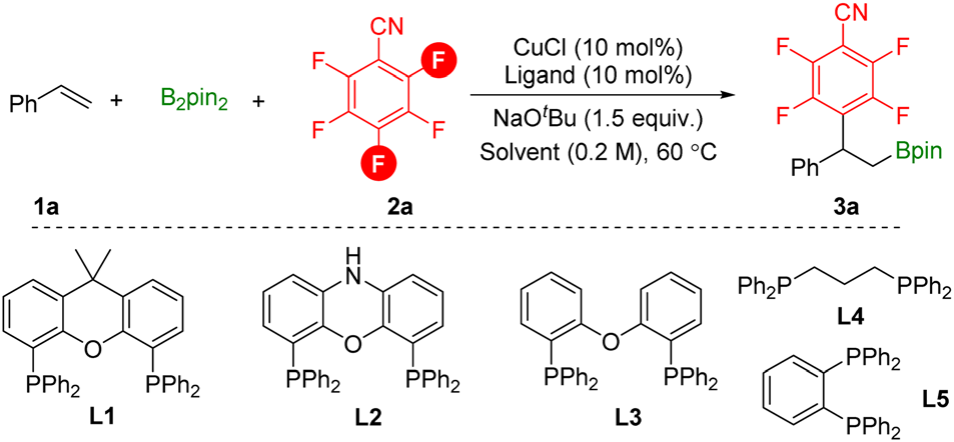				
Entry	Ligand	Solvent	Yield (%)	Ratio (*p* : *o*)
1	L1	Toluene	35	80/20
2	L1	*n*-Heptane	16	75/25
3	L1	1,4-Dioxane	45	86/14
4	L1	THF	57	93/7
5	L1	DMAc	0	n.d.
6	L2	THF	33	94/6
7	L3	THF	64	93/7
8	L4	THF	25	94/6
9	L5	THF	0	n.d.
10^b^	L3	THF	65	95/5
11^c^	L3	THF	90 (75)^d^	95/5

a Reaction conditions: styrene (0.2 mmol), pentafluorobenzonitrile (1.5 equiv.), B_2_pin_2_ (1.5 equiv.), CuCl (10 mol%), ligand (10 mol%), NaO^*t*^Bu (1.5 equiv.), solvent (0.2 M), 60 °C, 16 h.

b Stirred at room temperature (23 °C).

c NO^*t*^Bu (2.0 equiv.).

d Isolated yield.

With optimized reaction conditions in hand, we investigated various vinylarenes for this transformation. As shown in Scheme 3, styrenes bearing electron-donating groups can be utilized successfully and deliver the desired products in moderate to good yields with excellent regioselectivity (3b–3g). *meta*-, *ortho*-Substituted or disubstituted styrenes underwent this transformation smoothly to give the target products (3i–3n). Electron-withdrawing groups such as F, acetyl, and highly lipophilic OCF_3_ groups (3o–3q) on the styrenes were suitable as well. Furthermore, functional groups including borates, indole, pyrrole, morpholine, methylthio, amino, and furan groups (3r–3y) are well compatible, offering the corresponding products in moderate to good yields with excellent regioselectivity. In addition, the substrate containing terminal alkene (3z) was tolerated and selectively transformed. It is noteworthy that when 1,2-dihydronaphthalene was used, the corresponding *anti*-addition product (3aa) can be produced with good regioselectivity and diastereoselectivity which could benefit from the benzo fused system. *ortho*-Defluorinative products were obtained when styrenes contain sterically bulky groups or strong electron-withdrawing groups (3bb–3dd). More complex vinylarenes were successfully transformed under our defluorinative arylboration conditions, delivering the corresponding products in moderate to good yields. However, no reaction occurred with aliphatic alkenes (both internal and terminal).

**Scheme 3:**
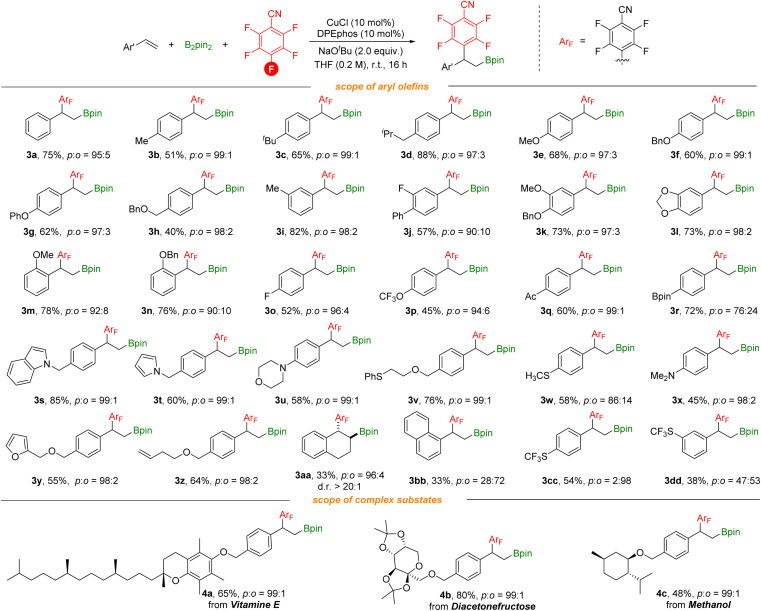
Reaction conditions: styrene (0.2 mmol), polyfluoroarenes (1.5 equiv.), B_2_pin_2_ (1.5 equiv.), CuCl (10 mol%), DPEphos (10 mol%), NaO^*t*^Bu (2.0 equiv.), THF (0.2 M), stirred at room temperature (23 °C) for 16 h, isolated yields. Site selectivity and ratio were determined by ^1^H NMR, ^19^F NMR and GC analysis.

To further explore the substrate scope of this defluorinative arylboration reaction, we further evaluated a series of polyfluoroarenes (Scheme 4). Tetrafluoro-substituted benzonitriles were competent coupling partners, providing the arylboration product 5a in moderate yield with poor regioselectivity and 5b with excellent regioselectivity. 3,4,5-Trifluorobenzonitrile was also assessed, and *meta*-defluorinative arylboration product 5c was obtained in moderate yield. Moreover, *p*-fluorobenzonitrile was tested as well, and a 15% yield of the corresponding product was detected by ^1^H NMR. CF_3_-substituted polyfluoroarenes and polyfluoropyridine were tested as well and corresponding products (5e and 5f) were obtained in moderate to good yields. However, this transformation is unsuitable with electron-rich polyfluoroarenes. The results obtained here are due to the joint effects from electronic and steric influences. Additionally, besides as the activating group, the nitrile group could also coordinate with the copper catalyst to facilitate C–F bond activation.

**Scheme 4:**
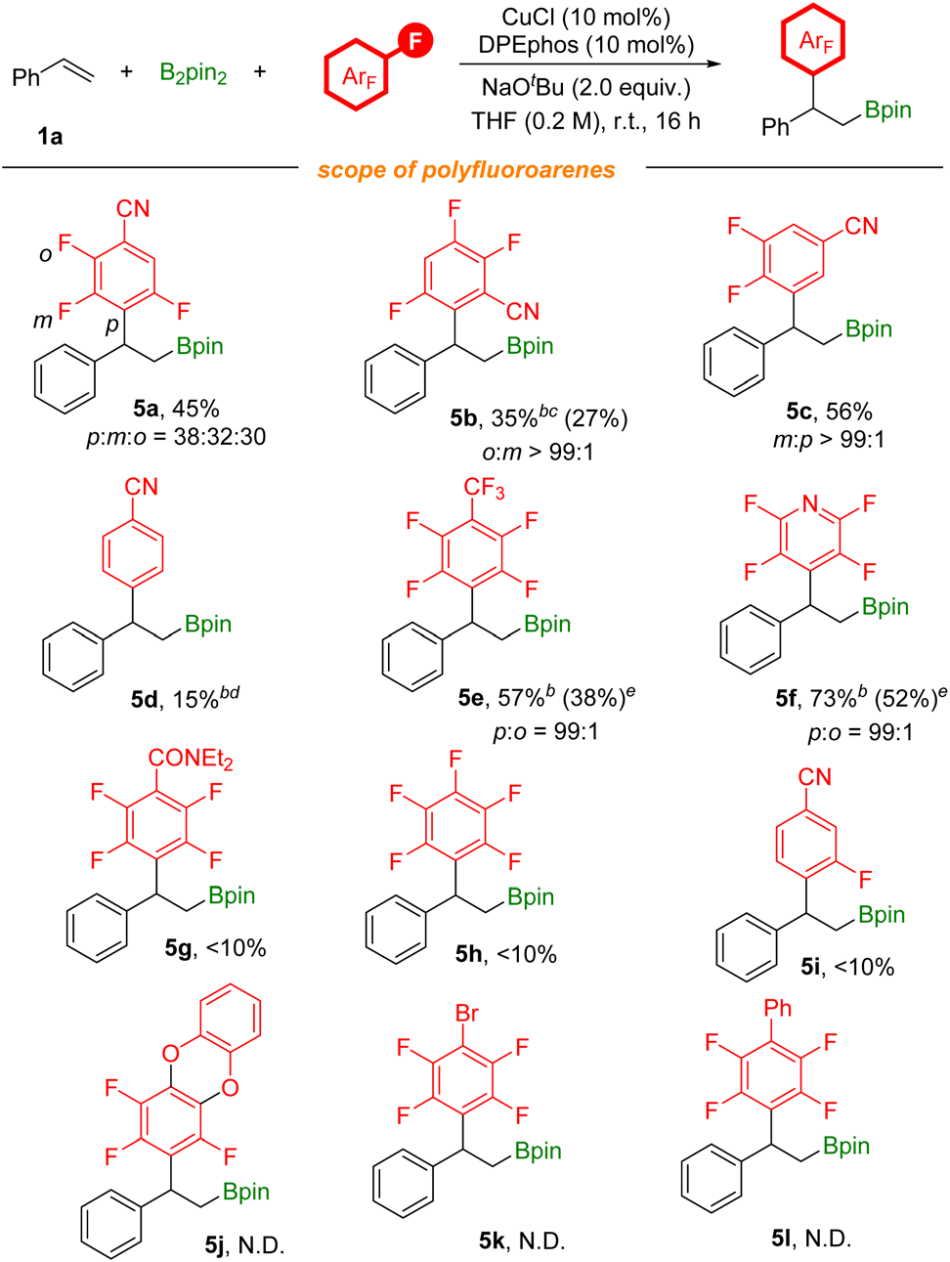
[a] Reaction conditions: styrene (0.2 mmol), polyfluoroarenes (1.5 equiv.), B_2_pin_2_ (1.5 equiv.), CuCl (10 mol%), DPEphos (10 mol%), NaO^*t*^Bu (2.0 equiv.), THF (0.2 M), stirred at room temperature (23 °C) for 16 h, isolated yields. Site selectivity and ratio were determined by ^1^H NMR, ^19^F NMR and GC analysis. [b] NMR yields using 1,3,5-trimethoxybenzene as the internal standard. [c] Xantphos (10 mol%), *n*-heptane (0.2 M), stirred at 60 °C. [d] Xantphos (10 mol%). [e] 5e and 5f were oxidized to the corresponding alcohols before isolation.

In order to further demonstrate the synthetic value of these defluorinative arylboration reactions, synthetic transformation of product 3a was carried out (Scheme 5). A gram-scale reaction was performed, and 3a was obtained in 60% yield. The C–B bond can be easily converted into a hydroxyl group by NaBO_3_ oxidation, providing the corresponding β-hydroxy polyfluoroarene (6a). Furthermore, high-value potassium borate salt (6b) was obtained in a simple step with KHF_2_. Subsequently, functional groups including bromo and vinyl were produced through bromination (6c) and vinylation (6d). Additionally, the product 3a and also the potassium borate salt (6b) are suitable for palladium-catalyzed C–C bond formation reactions based on reported procedures.^24^

**Scheme 5:**
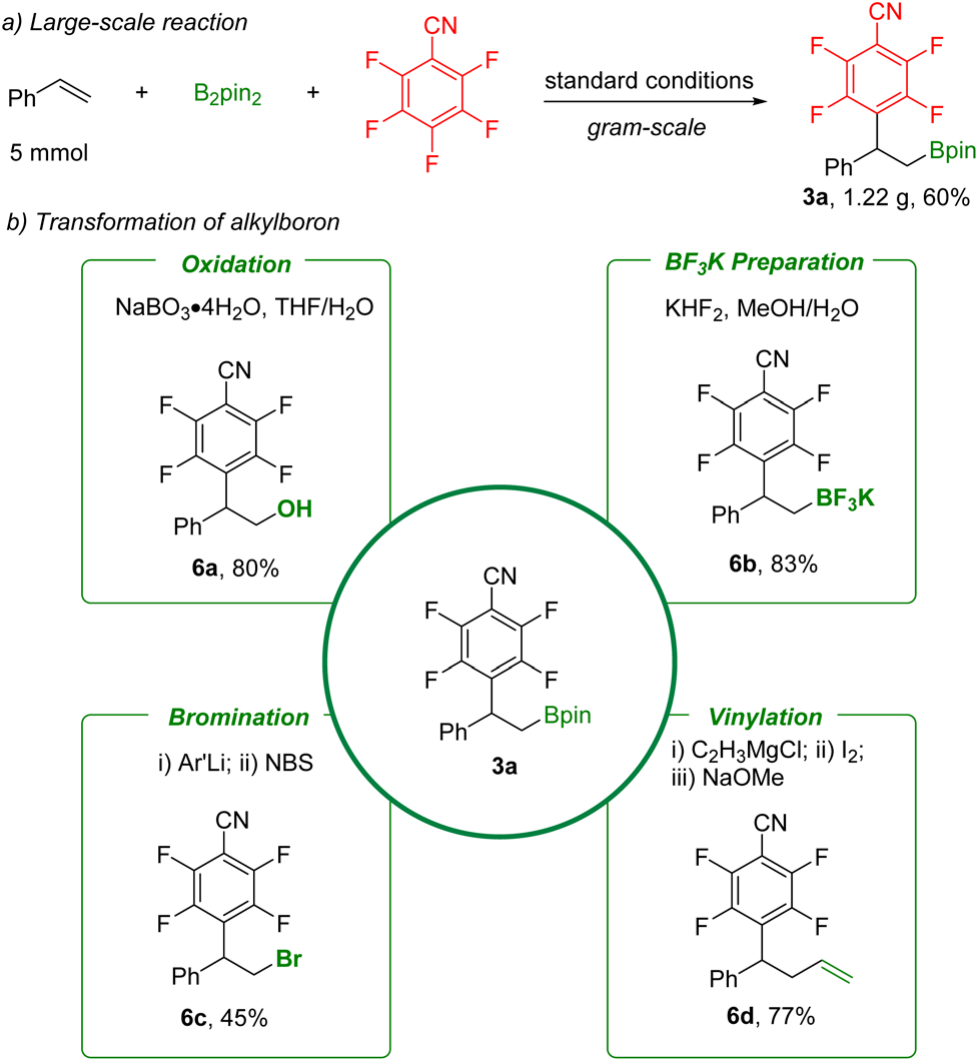
Large-scale reaction and transformations of organoboranes.

We subsequently set out to develop an enantioselective variant of the copper-catalyzed defluorinative arylboration of vinylarenes (Scheme 6). Styrene 1a and pentafluorobenzonitrile 2a were selected as model coupling partners. Copper complexes supported by chiral bidentate phosphine ligands were evaluated. In the presence of the chiral (*S*,*S*)-Ph-BPE L6*, the chiral 3a′ was afforded in 48% yield with 73% ee. Low conversions and enantioselectivity were measured with other commercially available chiral phosphine ligands L7*–L11*. Although, (*R*,*S*_p_)-Josiphos ligand L12* effectively improves the yield of 3a′, enantioselectivity was very poor. Thus, with the L6* as the best ligand, we further evaluated other parameters. The temperature has little effect on enantioselectivity, but an increase in the chemical yield was achieved. To our surprise, the transformation was processed with LiO^*t*^Bu as the base and delivered the 3a′ in moderate yield with excellent enantioselectivity. However, other bases, mixed bases, and solvents all failed to improve the yields since the formation of the side hydroboration products and vinyl boronate products could not be avoided (see ESI†).

**Scheme 6:**
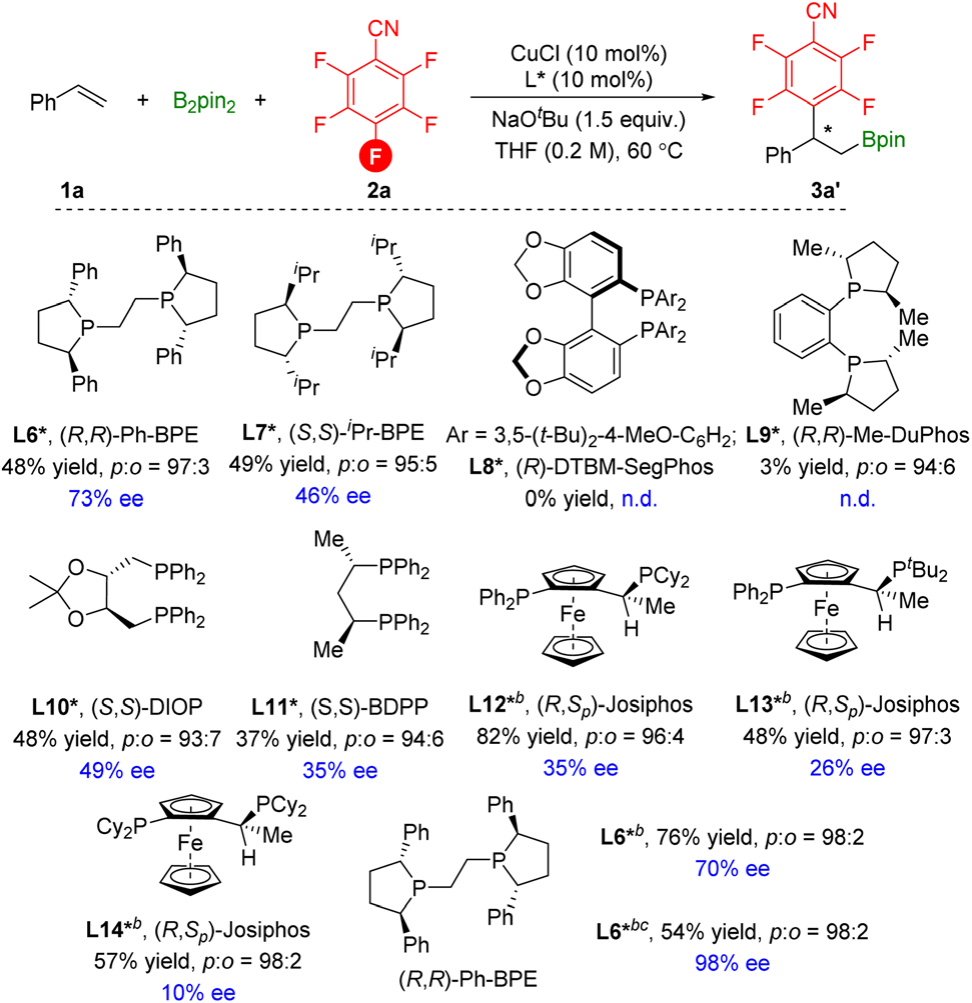
[a] Reaction conditions: styrene (0.2 mmol), pentafluorobenzonitrile (1.5 equiv.), B_2_pin_2_ (1.5 equiv.), CuCl (10 mol%), ligand (10 mol%), NaO^*t*^Bu (1.5 equiv.), THF (0.2 M), 60 °C, 16 h. [b] stirred at room temperature (23 °C). [c] LiO^*t*^Bu (2.0 equiv.) instead of NaO^*t*^Bu.

After obtaining the optimized asymmetric reaction conditions, we investigated the substrate scope of the reaction (Scheme 7). Overall, various styrene derivatives worked well under the catalytic system, leading to the corresponding chiral products in moderate yields with excellent enantioselectivities. Functional groups including boron, sulfur, and terminal alkene were all compatible to deliver the desired products with excellent enantioselectivities. The absolute configuration of 3c′ was clearly confirmed by X-ray crystallography.

**Scheme 7:**
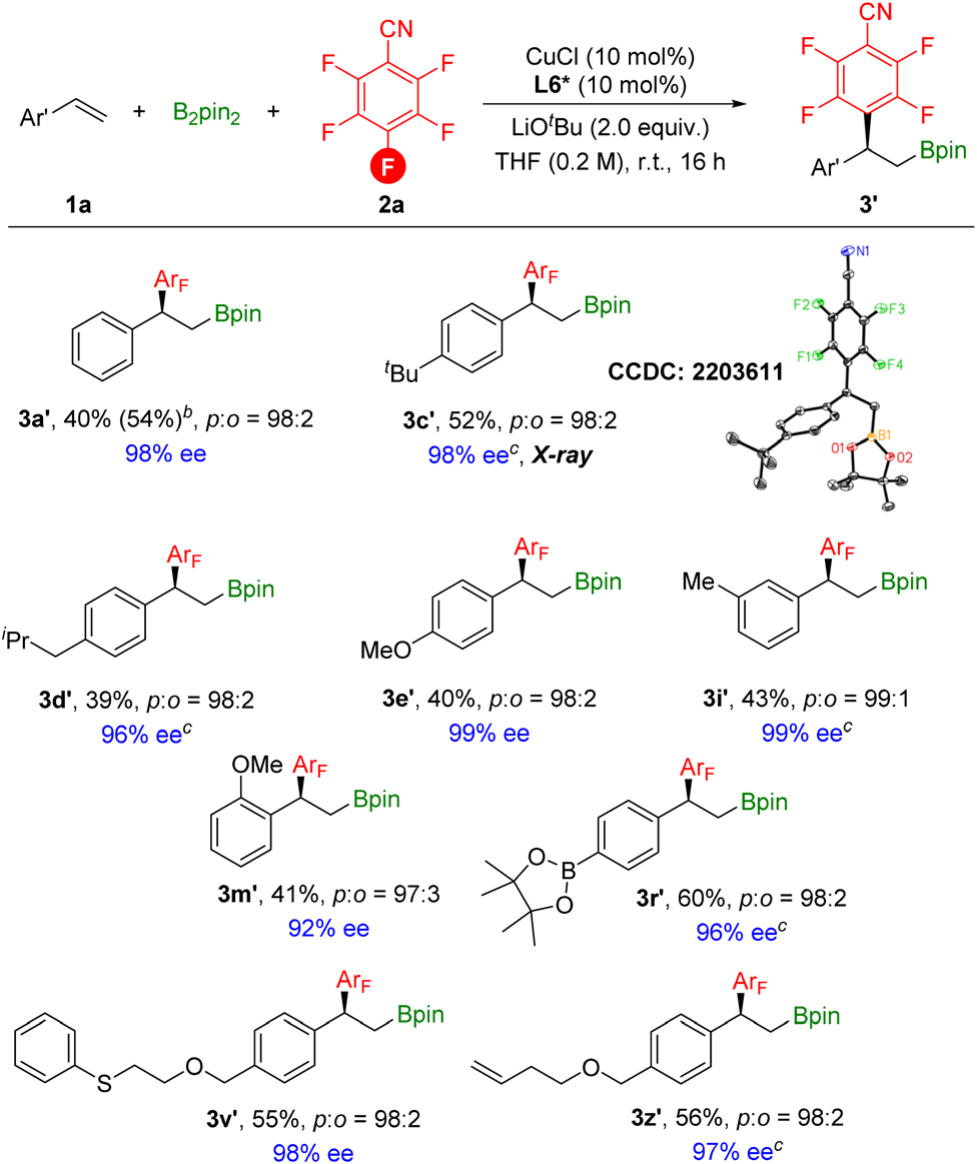
[a] Reaction conditions: styrene (0.2 mmol), 2,3,4,5,6-pentafluorobenzonitrile (1.5 equiv.), B_2_pin_2_ (1.5 equiv.), CuCl (10 mol%), L6* (10 mol%), LiO^*t*^Bu (2.0 equiv.), THF (0.2 M), stirred at room temperature (23 °C) for 16 h, isolated yields. Site selectivity and ratio were determined by ^1^H NMR, ^19^F NMR and GC analysis. Enantiomeric excess (ee) was determined by chiral-phase HPLC; displacement ellipsoid plot of 3c′ (30% probability level, without H and the second orientation of the disordered ^*t*^Bu group). [b] GC yield. [c] Ee was determined based on the corresponding alcohol.

In summary, starting from readily available vinylarenes and polyfluorenes, a copper-catalyzed defluorinative arylboration has been described in this study. The transformation provides a direct approach for the synthesis of β-polyfluoroaryl boronates and displays a broad functional group tolerance. Synthetic transformations of the β-polyfluoroaryl boronates demonstrate their utility. Notably, by using (*S*,*S*)-Ph-BPE as the ligand, an enantioselective defluorinative arylboration was also achieved.

## Author contributions

XFW directed this project and revised the manuscript. FPW, XWG and HQG performed all the experiments and prepared the manuscript and ESI.†

## Conflicts of interest

There are no conflicts to declare.

## Supplementary Material

SC-014-D2SC06472C-s001

SC-014-D2SC06472C-s002

## References

[cit1] Meanwell N. A. (2018). J. Med. Chem..

[cit2] Berger R., Resnati G., Metrangolo P., Weber E., Hulliger J. (2011). Chem. Soc. Rev..

[cit3] Bacchi S., Benaglia M., Cozzi F., Demartin F., Filippini G., Gavezzotti A. (2006). Chem.–Eur. J..

[cit4] He J., Li Z., Dhawan G., Zhang W., Sorochinsky A. E., Butler G., Soloshonok V. A., Han J. (2023). Chin. Chem. Lett..

[cit5] Szpera R., Moseley D. F. J., Smith L. B., Sterling A. J., Gouverneur V. (2019). Angew. Chem., Int. Ed..

[cit6] Champagne P. A., Desroches J., Hamel J. D., Vandamme M., Paquin J. F. (2015). Chem. Rev..

[cit7] Xie J., Rudolph M., Rominger F., Hashmi A. S. K. (2017). Angew. Chem., Int. Ed..

[cit8] Cybulski M. K., Nicholls J. E., Lowe J. P., Mahon M. F., Whittlesey M. K. (2017). Organometallics.

[cit9] Tian Y. M., Guo X. N., Kuntze-Fechner M. W., Krummenacher I., Braunschweig H., Radius U., Steffen A., Marder T. B. (2018). J. Am. Chem. Soc..

[cit10] Yi X., Mao R., Lavrencic L., Hu X. (2021). Angew. Chem., Int. Ed..

[cit11] Sun X., Ritter T. (2021). Angew. Chem., Int. Ed..

[cit12] Saijo H., Sakaguchi H., Ohashi M., Ogoshi S. (2014). Organometallics.

[cit13] Schaub T., Backes M., Radius U. (2006). J. Am. Chem. Soc..

[cit14] Ohashi M., Saijo H., Shibata M., Ogoshi S. (2013). Eur. J. Org. Chem..

[cit15] Li X., Fu B., Zhang Q., Yuan X., Zhang Q., Xiong T., Zhang Q. (2020). Angew. Chem., Int. Ed..

[cit16] Whyte A., Torelli A., Mirabi B., Zhang A., Lautens M. (2020). ACS Catal..

[cit17] Semba K., Nakao Y. (2014). J. Am. Chem. Soc..

[cit18] Trammel G. L., Kuniyil R., Crook P. F., Liu P., Brown M. K. (2021). J. Am. Chem. Soc..

[cit19] Liu Z., Chen J., Lu H. X., Li X., Gao Y., Coombs J. R., Goldfogel M. J., Engle K. M. (2019). Angew. Chem., Int. Ed..

[cit20] Wang W., Ding C., Li Y., Li Z., Li Y., Peng L., Yin G. (2019). Angew. Chem., Int. Ed..

[cit21] Liao Y., Yin X., Wang X., Yu W., Fang D., Hu L., Wang M., Liao J. (2020). Angew. Chem., Int. Ed..

[cit22] Wu F.-P., Yang Y., Fuentes D. P., Wu X.-F. (2022). Chem.

[cit23] Dherbassy Q., Manna S., Shi C., Prasitwatcharakorn W., Crisenza G. E. M., Perry G. J. P., Procter D. J. (2021). Angew. Chem., Int. Ed..

[cit24] Chemler S. R., Trauner D., Danishefsky S. J. (2001). Angew. Chem., Int. Ed..

