# Supplementary material for: Copper-catalyzed defluorinative arylboration of vinylarenes with polyfluoroarenes
Source: Chem Sci. 2023 Feb 6;14(9):2342–7. doi: 10.1039/d2sc06472c (PMC9977451; doi:10.1039/d2sc06472c)
Supplement: SC-014-D2SC06472C-s001 [file SC-014-D2SC06472C-s001.pdf]

Electronic Supplementary Material (ESI) for Chemical Science.
This journal is © The Royal Society of Chemistry 2023

Copper-Catalyzed Defluorinative Arylboration of Vinylarenes with Polyfluoroarenes

Fu-Peng Wu[1], Xing-Wei Gu[1], Hui-Qing Geng,[1] and Xiao-Feng Wu*,[1,2]

[1]Leibniz-Institut für Katalyse e.V., Albert-Einstein-Str. 29a, 18059, Rostock, Germany

[2]Dalian National Laboratory for Clean Energy, Dalian Institute of Chemical Physics, Chinese Academy of Sciences, 116023, Liaoning, China

*Correspondence to: xiao-feng.wu@catalysis.de

Contents

1. General information **S2**

2. Optimization of carbonylative catenation conditions **S3**

3. General procedure **S7**

4. Spectroscopic Data of Products **S8**

5. Derivatization of the B–C bond **S28**

6. X-ray crystal structure analysis of 3c' **S31**

7. References **S31**

8. NMR Spectra of the Products **S32**

9. Enantiomeric ratio monitoring **S124**

1. General information

Reagents, solvents, and analytical methods:

Unless otherwise noted, all reactions were carried out under a carbon monoxide or nitrogen atmosphere. The styrenes were synthesized according to existing method, and polyfluoroarenes and reagents were ordered from Sigma-Aldrich, TCI, ABCR, and Acros, and used without purification. All solvents were dried by standard techniques and distilled prior to use. Column chromatography was performed on silica gel (200-300 meshes) using *N*-pentane (bp. 36.1 °C), dichloromethane and ethyl acetate as eluent. All NMR spectra were recorded at ambient temperature using Bruker Avance III HD 300 NMR (^{1}H, 300 MHz; ^{13}C{^{1}H}, 75 MHz; ^{11}B, 96 MHz, ^{19}F, 282 MHz). ^{1}H NMR chemical shifts are reported relative to TMS and were referenced via residual proton resonances of the corresponding deuterated solvent ($CDCl_3$: 7.26 ppm; d_6-DMSO: 2.50 ppm) whereas ^{13}C{^{1}H} NMR spectra are reported relative to TMS via the carbon signals of the deuterated solvent ($CDCl_3$: 77.0 ppm; d_6-DMSO:39.5 ppm). Data for ^{1}H are reported as follows: chemical shift (δ ppm), multiplicity (s = singlet, d = doublet, t = triplet, q = quartet, quint = quintet, m = multiplet, br = broad), coupling constant (Hz), and integration. All ^{13}C NMR spectra were broad-band ^{1}H decoupled. **However, signals for the carbon attach to boron, C(alkyl)-B, are usually too broad to observe in the ^{13}C{^{1}H} NMR spectra**. Gas chromatography (**GC**) analyses were performed on an Agilent HP-7890A instrument with an FID detector and HP-5 capillary column (polydimethylsiloxane with 5% phenyl groups, 30 m, 0.32 mm i.d. 0.25 µm film thickness) using argon as carrier gas. High resolution mass spectra (**HRMS**) were recorded on an Agilent 6210 system. For chiral **HPLC-analysis** a device Agilent 1100 Series was used.

2. Optimization of carbonylative catenation conditions

Table S1. Optimization of solvent.

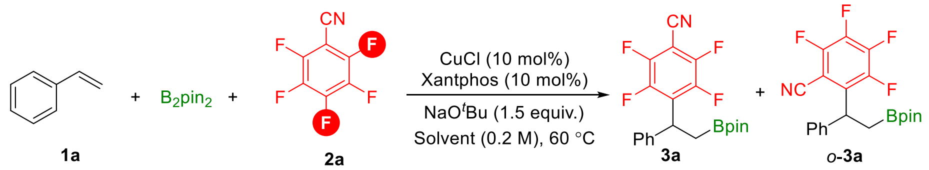

Entry	Conditions	Yield (%)	Ratio (*p*:*o*)
1	Toluene	35	80/20
2	*n*-Heptane	16	75/25
3	1,4-Dioxane	45	86/14
4	THF	57	93/7
5	2-MeTHF	11	93/7
6	MTBE	38	90/10
7	Et_2O	27	90/10
8	MeCN	13	94/6
9	DMAc	0	-

Reaction conditions: styrene (0.2 mmol), pentafluorobenzonitrile (1.5 equiv.), B_2pin_2 (1.5 equiv.), CuCl (10 mol%), Xantphos (10 mol%), NaO^{t}Bu (1.5 equiv.), Solvent (0.2 M), 60 ºC, 16 h.

Table S2. Optimization of base.

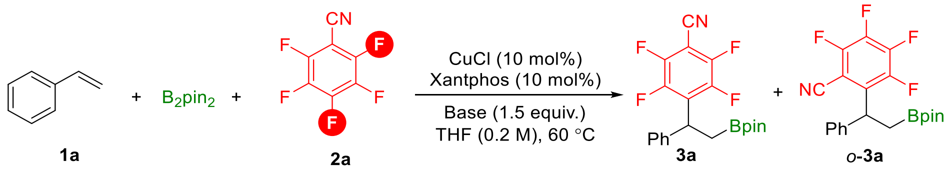

Entry	Conditions	Yield (%)	Ratio (*p*:*o*)
1	NaO^{t}Bu	57	93/7
2	KO^{t}Bu	37	93/7
3	LiO^{t}Bu	16	93/7
4	NaOEt	36	93/7
5	NaOMe	0	-
6	LiOMe	6	94/6
7	NaOTMS	17	96/4

Reaction conditions: styrene (0.2 mmol), pentafluorobenzonitrile (1.5 equiv.), B_2pin_2 (1.5 equiv.), CuCl (10 mol%), Xantphos (10 mol%), Base (1.5 equiv.), THF (0.2 M), stirred at 60 ºC for 16 h. GC yield.

Table S3. Optimization of copper source.

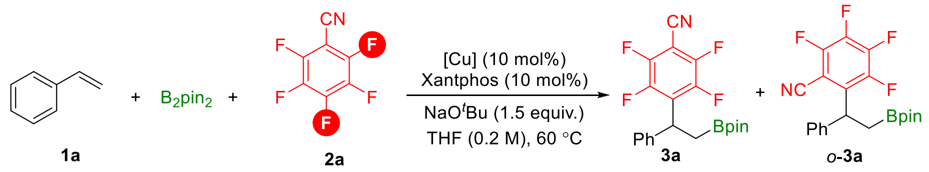

Entry	Conditions	Yield (%)	Ratio (*p*:*o*)
1	CuCl	57	93/7
2	CuBr	38	93/7
3	$Cu(OAc)_2$	43	93/7
4	$CuCl_2$	34	93/7
5	IPrCuCl	28	93/7
6	IMesCuCl	0	-

Reaction conditions: styrene (0.2 mmol), pentafluorobenzonitrile (1.5 equiv.), B_2pin_2 (1.5 equiv.), [Cu] (10 mol%), Xantphos (10 mol%), NaO^{t}Bu (1.5 equiv.), THF (0.2 M), stirred at 60 ºC for 16 h. GC yield.

Table S4. Optimization of ligand.

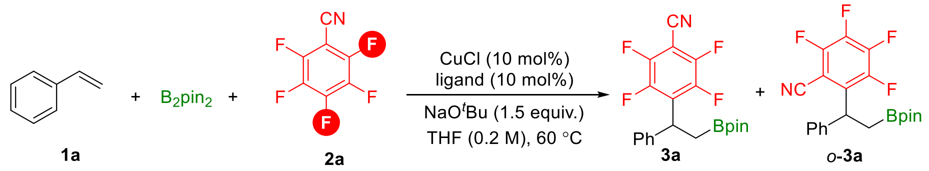

Entry	Conditions	Yield (%)	Ratio (*p*:*o*)
1	Xantphos	55	92/8
2	Ni-Xantphos	33	94/6
3	DPEphos	64	93/7
4	DPPP	25	94/6
5	Ph-BPE	48	97/3
6	QuinoxP*	6	92/8
7[b]	DPEphos	65	95/5
8[c]	DPEphos	90 (75)[d]	95/5

Reaction conditions: styrene (0.2 mmol), pentafluorobenzonitrile (1.5 equiv.), B_2pin_2 (1.5 equiv.), CuCl (10 mol%), ligand (10 mol%), NaO^{t}Bu (1.5 equiv.), THF (0.2 M), stirred at 60 ºC for 16 h. GC yield. [b]room temperature (23 ºC). [c]NaO^{t}Bu (2.0 equiv.). [d]isolated yiled.

Table S5. Asymmetric defluorinative arylboration.

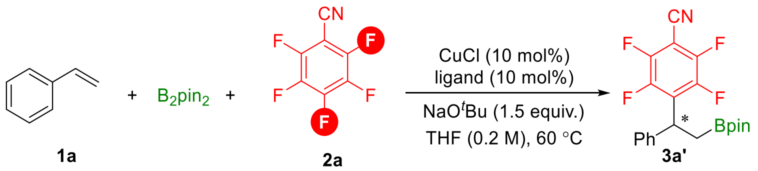

1a **2a** **3a'**

Entry	Conditions	Yield (%)	Ratio (*p*:*o*)	ee (%)
1	(*R*,*R*)-Ph-BPE (**L6***)	48	97/3	73
2	(*R*,*R*)-Me-BPE	8	92/8	n.d.
3	(*S*,*S*)-^{i}Pr-BPE	49	95/5	46
4	(*R*)-DTBM-SegPhos	0	n.d.	n.d.
5	(*S*)-Segphos	19	88/12	n.d.
6	(*R*,*R*)-Me-DuPhos	3	94/6	n.d.
7	(*S*,*S*)-DIOP	48	93/7	49
8	(*S*,*S*)-BDPP	37	94/6	32
9	(*R*,*R*)-QuinoxP	6	92/8	n.d.
10	(*S*,*S*)-Chiraphos	trace	n.d.	n.d.
11[b]	(*R*,*S*$_p$)-Josiphos	82	96/4	35
12[b]	(*R*,*S*$_p$)-Josiphos-2	48	97/3	26
13[b]	(*R*,*S*$_p$)-Josiphos-3	57	97/3	10
14[b]	(*S*)-MeO-Biphep	54	93/7	30
15[b]	(*R*,*R*)-Ph-BPE (**L6***)	76 (60)[d]	98/2	70
16[bc]	(*R*,*R*)-Ph-BPE (**L6***)	54 (40)[d]	98/2	98

Reaction conditions: styrene (0.2 mmol), pentafluorobenzonitrile (1.5 equiv.), B_2pin_2 (1.5 equiv.), CuCl (10 mol%), ligand (10 mol%), NaO^{t}Bu (1.5 equiv.), THF (0.2 M), 60 ºC, 16 h. [b]stirred at room temperature (23 ºC). [c]LiO^{t}Bu (2.0 equiv.). [d]Isolated yield.

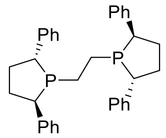

(*R*,*R*)-Ph-BPE

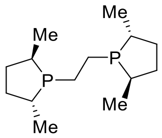

(*R*,*R*)-Me-BPE

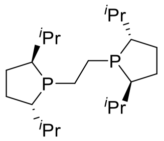

(*S*,*S*)-^{i}Pr-BPE

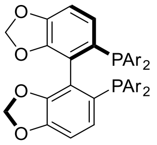

Ar = 3,5-(*t*-Bu)$_2$-4-MeO-C_6H_2;
(*R*)-DTBM-SegPhos

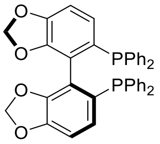

(*S*)-Segphos

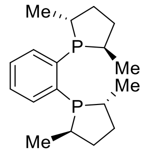

(*R*,*R*)-Me-DuPhos

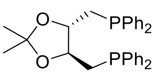

(*S*,*S*)-DIOP

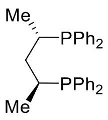

(S,S)-BDPP

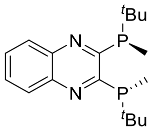

(*R*,*R*)-QuinoxP

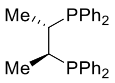

(*S*,*S*)-Chiraphos

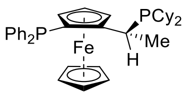

(*R*,*S*$_p$)-Josiphos

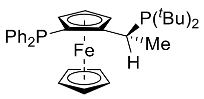

(*R*,*S*$_p$)-Josiphos-2

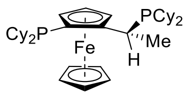

(*R*,*S*$_p$)-Josiphos-3

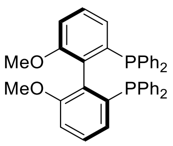

(S)-MeO-Biphep

Table S6. Variations from standard conditions.

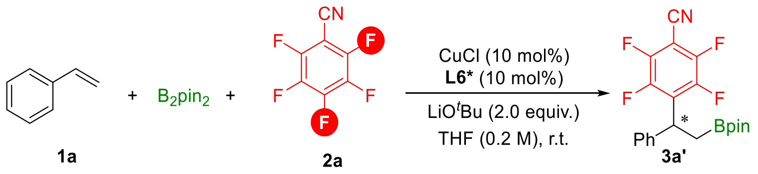

Entry	Variations from standard conditions	Yield (%)	Ratio (*p*:*o*)	ee (%)
1	-	54	98/2	98
2	NaO^{t}Bu instead of LiO^{t}Bu	76	98/2	70
3	KO^{t}Bu instead of LiO^{t}Bu	42	98/2	73
4	KOMe instead of LiO^{t}Bu	41	98/2	-
5	LiO^{t}Bu (1.5 equiv.)	16	98/2	-
6	CuCl (5 mol%) and **L6***(5 mol%)	20	98/2	-
7	CuCl (2 mol%) and **L6***(2 mol%)	8	98/2	-
8	LiO^{t}Bu (1.0 equiv.) and NaO^{t}Bu (1.0 equiv.)	81	98/2	70
9	1,4-dioxane instead of THF	39	97/3	-

Reaction conditions: styrene (0.2 mmol), pentafluorobenzonitrile (1.5 equiv.), B_2pin_2 (1.5 equiv.), CuCl (10 mol%), ligand (10 mol%), NaO^{t}Bu (1.5 equiv.), THF (0.2 M), 60 ºC, 16 h. [b]stirred at room temperature (23 ºC).

3. General procedure

3.1 defluorinative arylboration procedure I.

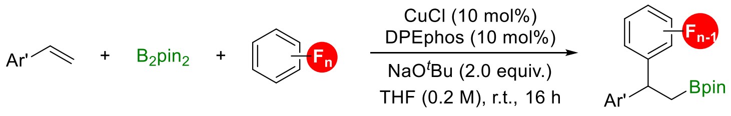

A 4 mL screw-cap vial was charged with CuCl (2.0 mg, 10 mol%), DPEphos (10.8 mg, 10 mol%), B_2pin_2 (76.2 mg, 1.5 equiv.), NaO^tBu (38.4 mg, 2.0 equiv.), and an oven-dried stir bar. The vial was closed with a Teflon septum and cap and connected to the atmosphere via a needle. After THF (1.0 mL, 0.2 M), olefins (0.2 mmol), and polyfluoroarene (1.5 equiv.) were added with a syringe under argon atmosphere, the vial was moved to an aluminum block. The reaction mixture was reacted at room temperature for 16 h. After the reaction was complete, the reaction was concentrated in vacuo. The crude product was purified by column chromatography on silica gel to afford the corresponding product.

3.2 defluorinative arylboration procedure II.

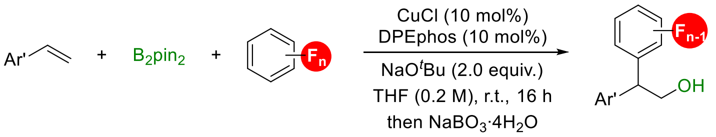

A 4 mL screw-cap vial was charged with CuCl (2.0 mg, 10 mol%), DPEphos (10.8 mg, 10 mol%), B_2pin_2 (76.2 mg, 1.5 equiv.), NaO^tBu (38.4 mg, 2.0 equiv.), and an oven-dried stir bar. The vial was closed with a Teflon septum and cap and connected to the atmosphere via a needle. After THF (1.0 mL, 0.2 M), olefins (0.2 mmol), and polyfluoroarene (1.5 equiv.) were added with a syringe under argon atmosphere, the vial was moved to an aluminum block. The reaction mixture was reacted at room temperature for 16 h. After the reaction was complete, the reaction was concentrated in vacuo. The crude product in THF (2.5 mL) and water (2.5 mL) was added $NaBO_3{\cdot}4H_2O$ (153.0 mg. 5 equiv). The reaction mixture was stirred vigorously for 30 min at room temperature. The reaction mixture was quenched with water and then extracted with ethyl acetate (5 mL). The combined organic layers were washed with brine (15 mL), dried over Na_2SO_4 and concentrated. The crude product was purified by column chromatography on silica gel to afford the corresponding product.

4. Spectroscopic Data of Products

4.1 Scope limitations.

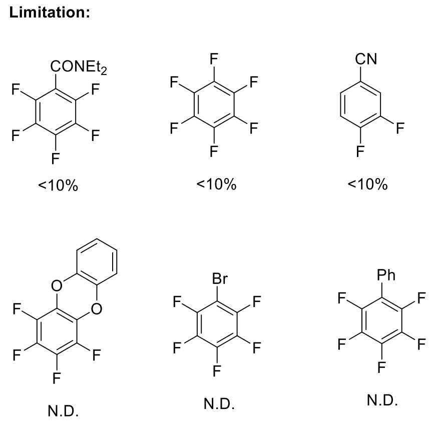

4.2 Spectroscopic data of products.

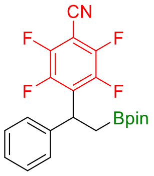

2,3,5,6-Tetrafluoro-4-(1-phenyl-2-(4,4,5,5-tetramethyl-1,3,2-dioxaborolan-2-yl)ethyl)benzonitrile (**3a**)

The title compound was prepared from styrene (23 uL, 0.2 mmol) and 2,3,4,5,6-pentafluorobenzonitrile (38 uL, 0.3 mmol) according to general defluorinative arylboration procedure I. The crude residue was purified by flash chromatography (pentane/EA = 20:1, R_f = 0.40) to give the product as a colorless oil (60.9 mg, 75%).

^{1}H NMR (300 MHz, CDCl$_3$) δ 7.32 – 7.16 (m, 4H), 7.16 – 7.09 (m, 1H), 4.68 (t, *J* = 8.7 Hz, 1H), 1.81 – 1.61 (m, 2H), 1.01 (s, 12H).

^{13}C NMR (75 MHz, CDCl$_3$) δ 147.0 (ddt, *J* = 261.2, 17.5, 3.5 Hz), 144.72 (dm, *J* = 248.1 Hz), 141.1, 132.3 (t, *J* = 15.8 Hz), 128.6, 127.4, 127.2, 107.5 (t, *J* = 3.7 Hz), 91.7 (tt, *J* = 17.2, 2.7 Hz), 83.5, 37.1, 24.5, 24.5, 14.8 (broad).

^{19}F NMR (282 MHz, CDCl$_3$) δ -133.03 – -133.55 (m), -138.59 (dt, *J* = 16.0, 8.0 Hz).

^{11}B NMR (96 MHz, CDCl$_3$) δ 33.0.

HRMS (EI): m/z calcd. for [M] $C_{21}H_{20}BF_4NO_2$ 405.15177, found 405.15171.

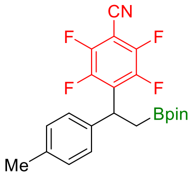

2,3,5,6-Tetrafluoro-4-(2-(4,4,5,5-tetramethyl-1,3,2-dioxaborolan-2-yl)-1-(*p*-tolyl)ethyl)benzonitrile **(3b)**

The title compound was prepared from 1-methyl-4-vinylbenzene (27 uL, 0.2 mmol) and 2,3,4,5,6-pentafluorobenzonitrile (38 uL, 0.3 mmol) according to general defluorinative arylboration procedure I. The crude residue was purified by flash chromatography (pentane/EA = 20:1, R_f = 0.40) to give the product as a colorless oil (43.0 mg, 51%).

^{1}H NMR (300 MHz, CDCl$_3$) δ 7.21 (d, *J* = 7.7 Hz, 2H), 7.10 (d, *J* = 8.5 Hz, 2H), 4.73 (t, *J* = 8.6 Hz, 1H), 2.31 (s, 3H), 1.87 – 1.66 (m, 2H), 1.10 (s, 12H).

^{13}C NMR (75 MHz, CDCl$_3$) δ 147.0 (dm, *J* = 261.2 Hz), 144.7 (dm, *J* = 248.1 Hz), 138.2, 137.0, 132.6 (t, *J* = 15.7 Hz), 129.3, 127.3, 107.7 (t, *J* = 3.7 Hz), 91.6, 83.5, 36.7, 24.6, 24.5, 21.0, 14.9 (broad).

^{19}F NMR (282 MHz, CDCl$_3$) δ -133.02 – -133.52 (m), -138.61 (td, *J* = 16.0, 6.9 Hz).

^{11}B NMR (96 MHz, CDCl$_3$) δ 32.9.

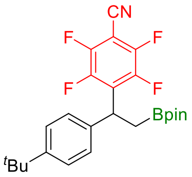

4-(1-(4-(*tert*-Butyl)phenyl)-2-(4,4,5,5-tetramethyl-1,3,2-dioxaborolan-2-yl)ethyl)-2,3,5,6-tetrafluorobenzonitrile (**3c**)

The title compound was prepared from 1-(*tert*-butyl)-4-vinylbenzene (37 uL, 0.2 mmol) and 2,3,4,5,6-pentafluorobenzonitrile (38 uL, 0.3 mmol) according to general defluorinative arylboration procedure I. The crude residue was purified by flash chromatography (pentane/EA = 20:1, R_f = 0.40) to give the product as a white solid (59.7 mg, 65%).

^{1}H NMR (300 MHz, CDCl$_3$) δ 7.36 – 7.28 (m, 2H), 7.28 – 7.21 (m, 2H), 4.73 (t, *J* = 8.8 Hz, 1H), 1.93 – 1.68 (m, 2H), 1.29 (s, 9H), 1.09 (d, *J* = 1.5 Hz, 12H).

^{13}C NMR (75 MHz, CDCl$_3$) δ 150.2, 147.0 (dm, *J* = 261.2 Hz), 144.8 (dm, *J* = 247.7 Hz), 138.1, 132.4 (t, *J* = 15.8 Hz), 127.1, 125.5, 107.7 (t, *J* = 3.9 Hz), 91.6, 83.5, 36.7, 34.4, 31.2, 24.5, 24.5, 14.9 (broad).

^{19}F NMR (282 MHz, CDCl$_3$) δ -133.34 (td, *J* = 16.4, 7.6 Hz), -138.47 (td, *J* = 16.4, 7.6 Hz).

^{11}B NMR (96 MHz, CDCl$_3$) δ 33.3.

HRMS (EI): m/z calcd. for [M] $C_{25}H_{28}BF_4NO_2$ 461.21437, found 461.21502.

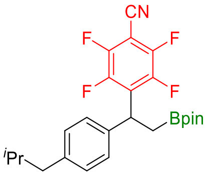

2,3,5,6-Tetrafluoro-4-(1-(4-isobutylphenyl)-2-(4,4,5,5-tetramethyl-1,3,2-dioxaborolan-2-yl)ethyl)benzonitrile (**3d**)

The title compound was prepared from 1-isobutyl-4-vinylbenzene (37 uL, 0.2 mmol) and 2,3,4,5,6-pentafluorobenzonitrile (38 uL, 0.3 mmol) according to general defluorinative arylboration procedure I. The crude residue was purified by flash chromatography (pentane/EA = 20:1, R_f = 0.40) to give the product as a colorless oil (81.0 mg, 88%).

^{1}H NMR (300 MHz, CDCl$_3$) δ 7.26 – 7.20 (m, 2H), 7.10 – 7.03 (m, 2H), 4.73 (t, *J* = 8.7 Hz, 1H), 2.43 (d, *J* = 7.2 Hz, 2H), 1.89 – 1.76 (m, 2H), 1.71 (dd, *J* = 15.9, 9.1 Hz, 1H), 1.10 (s, 6H), 1.09 (s, 6H), 0.88 (s, 3H), 0.86 (s, 3H).

^{13}C NMR (75 MHz, CDCl$_3$) δ 147.0 (dm, *J* = 261.2 Hz), 144.7 (dm, *J* = 247.7 Hz), 140.8, 138.4, 132.6 (t, *J* = 15.7 Hz), 129.3, 127.2 (t, *J* = 1.5 Hz), 107.7 (t, *J* = 3.7 Hz), 91.6, 83.5, 44.9, 36.8, 30.1, 24.5, 24.5, 22.2, 15.0 (broad).

^{19}F NMR (282 MHz, CDCl$_3$) δ -133.31 (td, *J* = 16.0, 6.9 Hz), -138.62 (td, *J* = 16.4, 7.2 Hz).

^{11}B NMR (96 MHz, CDCl$_3$) δ 33.3.

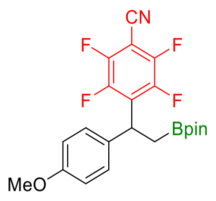

2,3,5,6-Tetrafluoro-4-(1-(4-methoxyphenyl)-2-(4,4,5,5-tetramethyl-1,3,2-dioxaborolan-2-yl)ethyl)benzonitrile (**3e**)

The title compound was prepared from 1-methoxy-4-vinylbenzene (27 uL, 0.2 mmol) and 2,3,4,5,6-pentafluorobenzonitrile (38 uL, 0.3 mmol) according to general defluorinative arylboration procedure I. The crude residue was purified by flash chromatography (pentane/EA = 20:1, R_f = 0.25) to give the product as a colorless oil (59.4 mg, 68%).

^{1}H NMR (300 MHz, $CDCl_3$) δ 7.30 – 7.21 (m, 2H), 6.87 – 6.79 (m, 2H), 4.71 (t, *J* = 8.7 Hz, 1H), 3.77 (s, 3H), 1.87 – 1.65 (m, 2H), 1.10 (s, 12H).

^{13}C NMR (75 MHz, $CDCl_3$) δ 158.7, 147.0 (dm, *J* = 261.2 Hz), 144.7 (dm, *J* = 247.7 Hz), 133.2, 132.7 (t, *J* = 15.7 Hz), 128.5, 114.0, 107.6 (t, *J* = 3.7 Hz), 91.8, 83.5, 55.2, 36.4, 24.5, 15.0 (broad).

^{19}F NMR (282 MHz, $CDCl_3$) δ -133.06 – -133.59 (m), -138.88 (td, *J* = 16.8, 7.6 Hz).

^{11}B NMR (96 MHz, $CDCl_3$) δ 33.3.

HRMS (EI): m/z calcd. for [M] $C_{22}H_{22}BF_4NO_3$ 435.16234, found 435.16259.

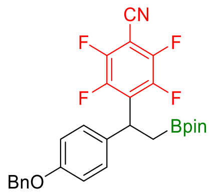

4-(1-(4-(Benzyloxy)phenyl)-2-(4,4,5,5-tetramethyl-1,3,2-dioxaborolan-2-yl)ethyl)-2,3,5,6-tetrafluorobenzonitrile (**3f**)

The title compound was prepared from 1-(benzyloxy)-4-vinylbenzene (42.0 mg, 0.2 mmol) and 2,3,4,5,6-pentafluorobenzonitrile (38 uL, 0.3 mmol) according to general defluorinative arylboration procedure I. The crude residue was purified by flash chromatography (pentane/EA = 20:1, R_f = 0.35) to give the product as a colorless oil (60.8 mg, 60%).

^{1}H NMR (300 MHz, $CDCl_3$) δ 7.44 – 7.30 (m, 5H), 7.26 (d, *J* = 8.0 Hz, 2H), 6.94 – 6.88 (m, 2H), 5.03 (s, 2H), 4.72 (t, *J* = 8.7 Hz, 1H), 1.76 (qd, *J* = 15.8, 8.7 Hz, 2H), 1.10 (s, 12H).

^{13}C NMR (75 MHz, $CDCl_3$) δ 157.9, 147.0 (dm, *J* = 261.2 Hz), 144.7 (d, *J* = 251.3 Hz), 136.8, 133.5, 132.6 (t, *J* = 15.5 Hz), 128.5, 127.9, 127.4, 114.9, 107.7 (t, *J* = 3.5Hz), 91.6, 83.5, 70.0, 36.4, 24.5, 15.1 (broad).

^{19}F NMR (282 MHz, $CDCl_3$) δ -132.99 – -133.41 (m), -138.83 (td, *J* = 16.4, 7.6 Hz).

^{11}B NMR (96 MHz, $CDCl_3$) δ 32.4.

HRMS (ESI-TOF): m/z calcd. for [M+Na$^+$] $C_{28}H_{26}BF_4NO_3$ 533.1875, found 533.1873.

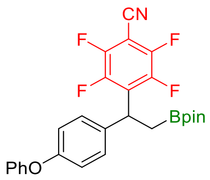

2,3,5,6-Tetrafluoro-4-(1-(4-phenoxyphenyl)-2-(4,4,5,5-tetramethyl-1,3,2-dioxaborolan-2-yl)ethyl)benzonitrile (**3g**)

The title compound was prepared from 1-phenoxy-4-vinylbenzene (37 uL, 0.2 mmol) and 2,3,4,5,6-pentafluorobenzonitrile (38 uL, 0.3 mmol) according to general defluorinative arylboration procedure I. The crude residue was purified by flash chromatography (pentane/EA = 20:1, R_f = 0.35) to give the product as a colorless oil (61.7 mg, 62%).

^{1}H NMR (300 MHz, CDCl$_3$) δ 7.36 – 7.27 (m, 4H), 7.13 – 7.06 (m, 1H), 7.01 – 6.90 (m, 4H), 4.75 (t, *J* = 8.5 Hz, 1H), 1.90 – 1.64 (m, 2H), 1.12 (s, 12H).

^{13}C NMR (75 MHz, CDCl$_3$) δ 156.9, 156.4, 147.0 (dm, *J* = 261.5 Hz), 144.7 (dm, *J* = 248.1 Hz), 135.8, 132.3 (t, *J* = 15.7 Hz), 129.7, 128.8, 123.4, 118.9, 118.8, 107.6 (t, *J* = 3.7 Hz), 91.6, 83.6, 36.5, 24.5, 15.1 (broad).

^{19}F NMR (282 MHz, CDCl$_3$) δ -133.07 (td, *J* = 16.0, 6.9 Hz), -138.73 (td, *J* = 16.4, 7.6 Hz).

^{11}B NMR (96 MHz, CDCl$_3$) δ 33.5.

HRMS (EI): m/z calcd. for [M] $C_{27}H_{24}BF_4NO_3$ 497.17799, found 497.17866.

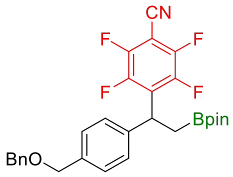

4-(1-(4-((Benzyloxy)methyl)phenyl)-2-(4,4,5,5-tetramethyl-1,3,2-dioxaborolan-2-yl)ethyl)-2,3,5,6-tetrafluorobenzonitrile (**3h**)

The title compound was prepared from 1-((benzyloxy)methyl)-4-vinylbenzene (44.8 mg, 0.2 mmol) and 2,3,4,5,6-pentafluorobenzonitrile (38 uL, 0.3 mmol) according to general defluorinative arylboration procedure I. The crude residue was purified by flash chromatography (pentane/EA = 20:1, R_f = 0.25) to give the product as a colorless oil (42.4 mg, 40%).

^{1}H NMR (300 MHz, CDCl$_3$) δ 7.35 (d, *J* = 4.5 Hz, 4H), 7.32 (s, 5H), 4.77 (t, *J* = 8.6 Hz, 1H), 4.54 (s, 2H), 4.52 (s, 2H), 1.92 – 1.67 (m, 2H), 1.11 (s, 12H).

^{13}C NMR (75 MHz, CDCl$_3$) δ 147.0 (dm, *J* = 261.2 Hz), 144.7 (dm, *J* = 248.1 Hz), 140.5, 138.1, 137.4, 132.3 (t, *J* = 15.7 Hz), 128.4, 128.1, 127.7, 127.6, 127.5, 107.6 (t, *J* = 3.7 Hz), 91.8, 83.5, 72.1, 71.6, 36.8, 24.5, 24.5, 14.9 (broad).

^{19}F NMR (282 MHz, CDCl$_3$) δ -133.15 (td, *J* = 16.4, 7.6 Hz), -138.50 (q, *J* = 9.2 Hz).

^{11}B NMR (96 MHz, CDCl$_3$) δ 34.1.

HRMS (ESI-TOF): m/z calcd. for [M+Na$^+$] $C_{29}H_{28}BF_4NO_3$ 547.2031, found 547.2037.

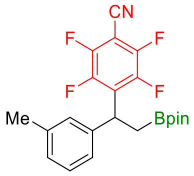

2,3,5,6-Tetrafluoro-4-(2-(4,4,5,5-tetramethyl-1,3,2-dioxaborolan-2-yl)-1-(m-tolyl)ethyl)benzonitrile (**3i**)

The title compound was prepared from 1-methyl-3-vinylbenzene (27 uL, 0.2 mmol) and 2,3,4,5,6-pentafluorobenzonitrile (38 uL, 0.3 mmol) according to general defluorinative arylboration procedure I. The crude residue was purified by flash chromatography (pentane/EA = 20:1, R_f = 0.40) to give the product as a colorless oil (68.3 mg, 82%).

^{1}H NMR (300 MHz, CDCl$_3$) δ 7.23 – 7.16 (m, 1H), 7.13 (d, *J* = 4.8 Hz, 2H), 7.04 (d, *J* = 6.4 Hz, 1H), 4.74 (t, *J* = 8.7 Hz, 1H), 2.32 (s, 3H), 1.77 (qd, *J* = 15.9, 8.6 Hz, 2H), 1.11 (s, 12H).

^{13}C NMR (75 MHz, CDCl$_3$) δ 147.0 (dm, *J* = 261.2 Hz), 144.7 (dm, *J* = 248.1 Hz), 141.0, 138.2, 132.4 (t, *J* = 15.7 Hz), 128.5, 128.2, 128.0, 124.4 (t, *J* = 1.7 Hz), 107.6 (t, *J* = 3.9 Hz), 91.7 (t, *J* = 17.2 Hz), 83.5, 37.0, 24.5, 24.5, 21.3, 14.9 (broad).

^{19}F NMR (282 MHz, CDCl$_3$) δ -133.30 (td, *J* = 16.4, 7.6 Hz), -138.51 (td, *J* = 16.4, 7.2 Hz).

^{11}B NMR (96 MHz, CDCl$_3$) δ 33.1.

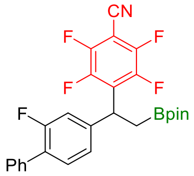

2,3,5,6-Tetrafluoro-4-(1-(2-fluoro-[1,1'-biphenyl]-4-yl)-2-(4,4,5,5-tetramethyl-1,3,2-dioxaborolan-2-yl)ethyl)benzonitrile (**3j**)

The title compound was prepared from 2-fluoro-4-vinyl-1,1'-biphenyl (39.6 mg, 0.2 mmol) and 2,3,4,5,6-pentafluorobenzonitrile (38 uL, 0.3 mmol) according to general defluorinative arylboration procedure I. The crude residue was purified by flash chromatography (pentane/EA = 20:1, R_f = 0.35) to give the product as a colorless oil (57.0 mg, 57%).

^{1}H NMR (300 MHz, CDCl$_3$) δ 7.51 (dt, *J* = 8.3, 1.6 Hz, 2H), 7.46 – 7.33 (m, 4H), 7.21 – 7.10 (m, 2H), 4.79 (t, *J* = 8.7 Hz, 1H), 1.96 – 1.65 (m, 2H), 1.13 (s, 12H).

^{13}C NMR (75 MHz, CDCl$_3$) δ 159.6 (d, *J* = 249.1 Hz), 142.5 (d, *J* = 7.1 Hz), 147.0 (dm, *J* = 261.5 Hz), 144.8 (dm, *J* = 248.4 Hz), 135.2, 135.2, 131.5 (t, *J* = 15.7 Hz), 130.8 (d, *J* = 4.0 Hz), 128.9 (d, *J* = 3.0 Hz), 128.4, 127.8, 123.4 (d, *J* = 3.4 Hz), 115.22 (d, *J* = 23.9 Hz), 107.5 (t, *J* = 3.6 Hz), 92.2, 83.7, 36.5, 24.6, 24.5, 14.8 (broad).

^{19}F NMR (282 MHz, CDCl$_3$) δ -117.14 (dd, *J* = 11.8, 8.0 Hz), -131.99 – -133.87 (m), -138.41 (td, *J* = 16.8, 7.6 Hz).

^{11}B NMR (96 MHz, CDCl$_3$) δ 33.6.

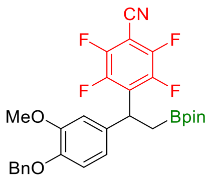

4-(1-(4-(Benzyloxy)-3-methoxyphenyl)-2-(4,4,5,5-tetramethyl-1,3,2-dioxaborolan-2-yl)ethyl)-2,3,5,6-tetrafluorobenzonitrile (**3k**)

The title compound was prepared from 1-(benzyloxy)-2-methoxy-4-vinylbenzene (48.0 mg, 0.2 mmol) and 2,3,4,5,6-pentafluorobenzonitrile (38 uL, 0.3 mmol) according to general defluorinative arylboration procedure I. The crude residue was purified by flash chromatography (pentane/EA = 10:1, R_f = 0.30) to give the product as a colorless oil (79.3 mg, 73%).

^{1}H NMR (300 MHz, CDCl$_3$) δ 7.44 – 7.38 (m, 2H), 7.37 – 7.27 (m, 3H), 6.89 (s, 1H), 6.85 – 6.76 (m, 2H), 5.11 (s, 2H), 4.69 (t, *J* = 8.7 Hz, 1H), 3.87 (s, 3H), 1.88 – 1.65 (m, 2H), 1.09 (s, 6H), 1.08 (s, 6H).

^{13}C NMR (75 MHz, CDCl$_3$) δ 149.5, 147.3, 147.0 (dm, *J* = 261.5 Hz), 144.6 (dm, *J* = 247.7 Hz), 137.0, 134.1, 132.4 (t, *J* = 15.7 Hz), 128.5, 127.8, 127.2, 119.7, 113.9, 111.3, 107.6 (t, *J* = 3.8 Hz), 91.6, 83.5, 70.9, 56.0, 36.7, 24.5, 24.5, 15.1 (broad).

^{19}F NMR (282 MHz, CDCl$_3$) δ -133.11 (td, *J* = 16.4, 7.6 Hz), -138.84 (td, *J* = 16.4, 7.2 Hz).

^{11}B NMR (96 MHz, CDCl$_3$) δ 33.5.

HRMS (ESI-TOF): m/z calcd. for [M+Na$^+$] $C_{29}H_{28}BF_4NO_4$ 563.1981, found 563.1997.

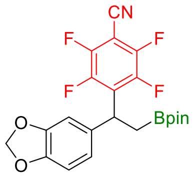

4-(1-(Benzo[d][1,3]dioxol-5-yl)-2-(4,4,5,5-tetramethyl-1,3,2-dioxaborolan-2-yl)ethyl)-2,3,5,6-tetrafluorobenzonitrile (**3l**)

The title compound was prepared from 5-vinylbenzo[d][1,3]dioxole (26 uL, 0.2 mmol) and 2,3,4,5,6-pentafluorobenzonitrile (38 uL, 0.3 mmol) according to general defluorinative arylboration procedure I. The crude residue was purified by flash chromatography (pentane/EA = 20:1, R_f = 0.25) to give the product as a colorless oil (65.2 mg, 73%).

^{1}H NMR (300 MHz, CDCl$_3$) δ 6.79 (d, *J* = 8.7 Hz, 2H), 6.71 (dd, *J* = 7.8, 0.7 Hz, 1H), 5.95 – 5.88 (m, 2H), 4.67 (t, *J* = 8.6 Hz, 1H), 1.81 – 1.63 (m, 2H), 1.11 (s, 12H).

^{13}C NMR (75 MHz, CDCl$_3$) δ 147.8, 147.0 (dm, *J* = 261.5 Hz), 146.6, 144.6 (dm, *J* = 248.1 Hz), 135.0, 132.3 (t, *J* = 15.7 Hz), 120.6 (t, *J* = 1.7 Hz), 108.2, 107.9 (t, *J* = 2.0 Hz), 107.6 (t, *J* = 3.7 Hz), 101.1, 91.7, 83.5, 36.8, 24.5, 24.5, 15.2 (broad).

^{19}F NMR (282 MHz, CDCl$_3$) δ -133.13 (td, *J* = 16.8, 7.6 Hz), -138.79 (td, *J* = 16.0, 6.9 Hz).

^{11}B NMR (96 MHz, CDCl$_3$) δ 33.2.

HRMS (EI): m/z calcd. for [M] $C_{22}H_{20}BF_4NO_4$ 449.14160, found 449.14240.

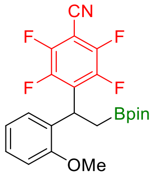

2,3,5,6-Tetrafluoro-4-(1-(2-methoxyphenyl)-2-(4,4,5,5-tetramethyl-1,3,2-dioxaborolan-2-yl)ethyl)benzonitrile (**3m**)

The title compound was prepared from 1-methoxy-2-vinylbenzene (27 uL, 0.2 mmol) and 2,3,4,5,6-pentafluorobenzonitrile (38 uL, 0.3 mmol) according to general defluorinative arylboration procedure I. The crude residue was purified by flash chromatography (pentane/EA = 20:1, R_f = 0.25) to give the product as a colorless oil (68.2 mg, 78%).

^{1}H NMR (300 MHz, $CDCl_3$) δ 7.48 (d, *J* = 8.4 Hz, 1H), 7.29 – 7.20 (m, 1H), 6.97 (td, *J* = 7.6, 1.2 Hz, 1H), 6.78 (dd, *J* = 8.3, 1.2 Hz, 1H), 4.93 (t, *J* = 8.7 Hz, 1H), 3.73 (s, 3H), 1.69 (d, *J* = 8.6 Hz, 2H), 1.13 (s, 6H), 1.11 (s, 6H).

^{13}C NMR (75 MHz, $CDCl_3$) δ 156.9, 146.7 (dm, *J* = 259.8 Hz), 145.1 (dm, *J* = 248.7 Hz), 132.4 (t, *J* = 15.3 Hz), 128.9, 128.4, 127.6 (t, *J* = 2.4 Hz), 120.3, 110.1, 107.9 (t, *J* = 3.9 Hz), 91.1, 83.4, 55.2, 31.0, 24.6, 24.5, 13.9 (broad).

^{19}F NMR (282 MHz, $CDCl_3$) δ -134.61 (td, *J* = 16.4, 7.6 Hz), -138.39 (dq, *J* = 16.8, 8.0 Hz).

^{11}B NMR (96 MHz, $CDCl_3$) δ 33.2.

HRMS (ESI-TOF): m/z calcd. for [M+Na$^+$] $C_{22}H_{22}BF_4NO_3$ 457.1562, found 457.1562.

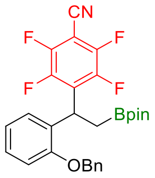

4-(1-(2-(Benzyloxy)phenyl)-2-(4,4,5,5-tetramethyl-1,3,2-dioxaborolan-2-yl)ethyl)-2,3,5,6-tetrafluorobenzonitrile (**3n**)

The title compound was prepared from 1-(benzyloxy)-2-vinylbenzene (42.0 mg, 0.2 mmol) and 2,3,4,5,6-pentafluorobenzonitrile (38 uL, 0.3 mmol) according to general defluorinative arylboration procedure I. The crude residue was purified by flash chromatography (pentane/EA = 20:1, R_f = 0.35) to give the product as a colorless oil (78.0 mg, 76%).

^{1}H NMR (300 MHz, $CDCl_3$) δ 7.54 (d, *J* = 7.7 Hz, 1H), 7.44 – 7.32 (m, 3H), 7.32 – 7.17 (m, 3H), 7.03 (td, *J* = 7.6, 1.3 Hz, 1H), 6.91 (dd, *J* = 8.3, 1.2 Hz, 1H), 5.10 – 4.84 (m, 3H), 1.69 (qd, *J* = 15.6, 8.6 Hz, 2H), 1.14 (d, *J* = 9.1 Hz, 12H).

^{13}C NMR (75 MHz, $CDCl_3$) δ 156.1, 146.6 (dm, *J* = 260.2Hz), 144.9 (dm, *J* = 248.7 Hz), 136.1, 132.8 (t, *J* = 15.1 Hz), 129.1, 128.5, 128.4, 128.3, 127.9, 127.8, 120.6, 111.2, 108.0 (t, *J* = 3.7 Hz), 90.9 (t, *J* = 17.2 Hz), 83.5, 70.2, 31.2, 24.6, 14.2 (broad).

^{19}F NMR (282 MHz, $CDCl_3$) δ -134.42 (td, *J* = 16.4, 7.6 Hz), -137.95 (td, *J* = 16.4, 7.6 Hz).

^{11}B NMR (96 MHz, $CDCl_3$) δ 33.1.

HRMS (ESI-TOF): m/z calcd. for [M+Na$^+$] $C_{28}H_{26}BF_4NO_3$ 533.1875, found 533.1873.

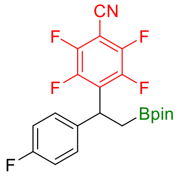

2,3,5,6-Tetrafluoro-4-(1-(4-fluorophenyl)-2-(4,4,5,5-tetramethyl-1,3,2-dioxaborolan-2-yl)ethyl)benzonitrile (**3o**)

The title compound was prepared from 1-fluoro-4-vinylbenzene (24 uL, 0.2 mmol) and 2,3,4,5,6-pentafluorobenzonitrile (38 uL, 0.3 mmol) according to general defluorinative arylboration procedure I. The crude residue was purified by flash chromatography (pentane/EA = 20:1, R_f = 0.40) to give the product as a colorless oil (43.5 mg, 52%).

^{1}H NMR (300 MHz, $CDCl_3$) δ 7.30 (dd, *J* = 8.2, 5.2 Hz, 2H), 7.03 – 6.94 (m, 2H), 4.74 (t, *J* = 8.7 Hz, 1H), 1.87 – 1.62 (m, 2H), 1.10 (s, 6H), 1.10 (s, 6H).

^{13}C NMR (75 MHz, $CDCl_3$) δ 161.9 (d, *J* = 246.3 Hz), 147.2 (dm, *J* = 262.1 Hz), 144.63 (dm, *J* = 254.2 Hz), 136.9 (d, *J* = 3.3 Hz), 132.0 (t, *J* = 15.5 Hz), 129.1 (dt, *J* = 8.0, 1.7 Hz), 115.48 (d, *J* = 21.3 Hz), 107.5 (t, *J* = 3.8 Hz), 92.0, 83.6, 36.4, 24.5, 14.1 (broad).

^{19}F NMR (282 MHz, $CDCl_3$) δ -114.74 – -115.27 (m), -132.85 – -133.11 (m), -138.85 (td, *J* = 16.3, 7.4 Hz).

^{11}B NMR (96 MHz, $CDCl_3$) δ 33.1.

HRMS (EI): m/z calcd. for [M] $C_{21}H_{19}BF_5NO_2$ 423.14235, found 423.14286.

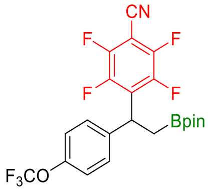

2,3,5,6-Tetrafluoro-4-(2-(4,4,5,5-tetramethyl-1,3,2-dioxaborolan-2-yl)-1-(4-(trifluoromethoxy)phenyl)ethyl)benzonitrile (**3p**)

The title compound was prepared from 1-(trifluoromethoxy)-4-vinylbenzene (34 uL, 0.2 mmol) and 2,3,4,5,6-pentafluorobenzonitrile (38 uL, 0.3 mmol) according to general defluorinative arylboration procedure I. The crude residue was purified by flash chromatography (pentane/EA = 20:1, R_f = 0.30) to give the product as a colorless oil (43.8 mg, 45%).

^{1}H NMR (300 MHz, $CDCl_3$) δ 7.36 (d, *J* = 8.2 Hz, 2H), 7.15 (dd, *J* = 8.8, 1.1 Hz, 2H), 4.76 (t, *J* = 8.6 Hz, 1H), 1.90 – 1.65 (m, 2H), 1.10 (s, 6H), 1.09 (s, 6H).

^{13}C NMR (75 MHz, $CDCl_3$) δ 148.3 (q, *J* = 3.3 Hz), 147.1 (dm, *J* = 261.5 Hz), 144.7 (d, *J* = 248.1 Hz), 139.8, 131.6 (t, *J* = 15.7 Hz), 129.0, 121.1, 120.4 (q, *J* = 257.5 Hz), 107.5 (t, *J* = 3.7 Hz), 92.2, 83.7, 36.5, 24.6, 24.5, 14.9 (broad).

^{19}F NMR (282 MHz, $CDCl_3$) δ -57.98, -132.78 (td, *J* = 16.8, 7.6 Hz), -138.69 (td, *J* = 16.0, 6.9 Hz).

^{11}B NMR (96 MHz, $CDCl_3$) δ 33.3.

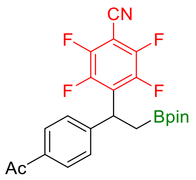

4-(1-(4-Acetylphenyl)-2-(4,4,5,5-tetramethyl-1,3,2-dioxaborolan-2-yl)ethyl)-2,3,5,6-tetrafluorobenzonitrile (**3q**)

The title compound was prepared from 1-(4-vinylphenyl)ethan-1-one (29.2 mg, 0.2 mmol) and 2,3,4,5,6-pentafluorobenzonitrile (38 uL, 0.3 mmol) according to general defluorinative arylboration procedure I. The crude residue was purified by flash chromatography (pentane/EA = 10:1, R_f = 0.25) to give the product as a colorless oil (53.3 mg, 60%).

^{1}H NMR (300 MHz, CDCl$_3$) δ 7.94 – 7.86 (m, 2H), 7.41 (d, *J* = 8.1 Hz, 2H), 4.81 (t, *J* = 8.5 Hz, 1H), 2.57 (s, 3H), 1.90 – 1.68 (m, 2H), 1.11 (s, 6H), 1.10 (s, 6H).

^{13}C NMR (75 MHz, CDCl$_3$) δ 197.4, 147.1 (dm, *J* = 262.0 Hz), 146.3, 144.7 (dm, *J* = 246.0 Hz), 136.1, 131.4 (t, *J* = 15.7 Hz), 128.7, 127.7, 107.4 (t, *J* = 3.5 Hz), 92.3, 83.7, 37.0, 26.6, 24.6, 24.5.

^{19}F NMR (282 MHz, CDCl$_3$) δ -132.58 – -132.86 (m), -138.41 (td, *J* = 16.0, 6.9 Hz).

^{11}B NMR (96 MHz, CDCl$_3$) δ 32.7.

HRMS (EI): m/z calcd. for [M] $C_{23}H_{22}BF_4NO_3$ 447.16234, found 447.16288.

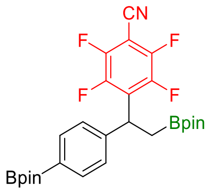

2,3,5,6-Tetrafluoro-4-(2-(4,4,5,5-tetramethyl-1,3,2-dioxaborolan-2-yl)-1-(4-(4,4,5,5-tetramethyl-1,3,2-dioxaborolan-2-yl)phenyl)ethyl)benzonitrile (**3r**)

The title compound was prepared from 4,4,5,5-tetramethyl-2-(4-vinylphenyl)-1,3,2-dioxaborolane (49 uL, 0.2 mmol) and 2,3,4,5,6-pentafluorobenzonitrile (38 uL, 0.3 mmol) according to general defluorinative arylboration procedure I. The crude residue was purified by flash chromatography (pentane/EA = 20:1, R_f = 0.20) to give the product as a colorless oil (76.1 mg, 72%).

^{1}H NMR (300 MHz, CDCl$_3$) δ 7.74 (dd, *J* = 8.2, 1.5 Hz, 2H), 7.33 (t, *J* = 8.4 Hz, 2H), 4.77 (t, *J* = 8.5 Hz, 1H), 1.88 – 1.68 (m, 2H), 1.32 (s, 12H), 1.10 (s, 12H).

^{13}C NMR (75 MHz, CDCl$_3$) δ 147.0 (dm, *J* = 261.5 Hz), 144.7 (dm, *J* = 249.1 Hz), 144.2, 135.1, 132.1 (t, *J* = 15.7 Hz), 126.7, 107.6 (t, *J* = 3.7 Hz), 91.8, 83.8, 83.6, 37.1, 24.8, 24.6, 24.5, 14.7 (broad).

^{19}F NMR (282 MHz, CDCl$_3$) δ -132.82 – -133.50 (m), -138.40 (td, *J* = 16.0, 6.9 Hz).

^{11}B NMR (96 MHz, CDCl$_3$) δ 30.7.

HRMS (ESI-TOF): m/z calcd. for [M+Na$^+$] $C_{27}H_{31}B_2F_4NO_4$ 552.2345, found 552.2350.

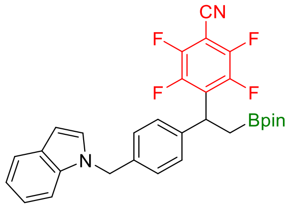

4-(1-(4-((1*H*-Indol-1-yl)methyl)phenyl)-2-(4,4,5,5-tetramethyl-1,3,2-dioxaborolan-2-yl)ethyl)-2,3,5,6-tetrafluorobenzonitrile (**3s**)

The title compound was prepared from 1-(4-vinylbenzyl)-1*H*-indole (46.6 mg, 0.2 mmol) and 2,3,4,5,6-pentafluorobenzonitrile (38 uL, 0.3 mmol) according to general defluorinative arylboration procedure I. The crude residue was purified by flash chromatography (pentane/EA = 10:1, R_f = 0.20) to give the product as a colorless oil (90.5 mg, 85%).

^{1}H NMR (300 MHz, $CDCl_3$) δ 7.64 (ddd, *J* = 7.3, 1.6, 0.8 Hz, 1H), 7.29 – 7.22 (m, 3H), 7.19 – 7.02 (m, 5H), 6.54 (dd, *J* = 3.2, 0.9 Hz, 1H), 5.29 (s, 2H), 4.72 (t, *J* = 8.7 Hz, 1H), 1.85 – 1.66 (m, 2H), 1.09 (s, 12H).

^{13}C NMR (75 MHz, $CDCl_3$) δ 146.9 (dm, *J* = 261.8 Hz), 144.7 (dm, *J* = 231.6 Hz), 140.6, 136.7, 136.2, 132.0 (t, *J* = 15.9 Hz), 128.7, 128.1, 127.8, 127.1, 121.6, 121.0, 119.5, 109.6, 107.6 (t, *J* = 3.8 Hz), 101.7, 92.4, 83.6, 49.7, 36.8, 24.5, 24.5.

^{19}F NMR (282 MHz, $CDCl_3$) δ -131.82 – -133.84 (m), -138.56 (td, *J* = 16.3, 7.4 Hz).

^{11}B NMR (96 MHz, $CDCl_3$) δ 33.6.

HRMS (ESI-TOF): m/z calcd. for $[M+Na^+]$ $C_{30}H_{27}BF_4N_2O_2$ 556.2030, found 556.2035.

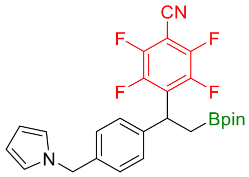

4-(1-(4-((1*H*-Pyrrol-1-yl)methyl)phenyl)-2-(4,4,5,5-tetramethyl-1,3,2-dioxaborolan-2-yl)ethyl)-2,3,5,6-tetrafluorobenzonitrile (**3t**)

The title compound was prepared from 1-(4-vinylbenzyl)-1*H*-pyrrole (36.6 mg, 0.2 mmol) and 2,3,4,5,6-pentafluorobenzonitrile (38 uL, 0.3 mmol) according to general defluorinative arylboration procedure I. The crude residue was purified by flash chromatography (pentane/EA = 10:1, R_f = 0.30) to give the product as a colorless oil (58.4 mg, 60%).

^{1}H NMR (300 MHz, $CDCl_3$) δ 7.29 (d, *J* = 7.9 Hz, 2H), 7.11 – 7.02 (m, 2H), 6.65 (t, *J* = 2.1 Hz, 2H), 6.17 (t, *J* = 2.1 Hz, 2H), 5.03 (s, 2H), 4.74 (t, *J* = 8.7 Hz, 1H), 1.86 – 1.69 (m, 2H), 1.10 (s, 12H).

^{13}C NMR (75 MHz, $CDCl_3$) δ 147.0 (dm, *J* = 261.2 Hz), 144.7 (dm, *J* = 247.7 Hz), 140.6, 137.3, 132.0 (t, *J* = 15.7 Hz), 127.8, 127.3, 121.0, 108.5, 107.6 (t, *J* = 3.7 Hz), 91.8, 83.6, 52.8, 36.8, 24.5, 24.5, 14.2 (broad).

^{19}F NMR (282 MHz, $CDCl_3$) δ -132.84 – -133.38 (m), -138.53 (q, *J* = 8.8 Hz).

^{11}B NMR (96 MHz, $CDCl_3$) δ 33.2.

HRMS (ESI-TOF): m/z calcd. for $[M+Na^+]$ $C_{26}H_{25}BF_4N_2O_2$ 506.1873, found 506.1878.

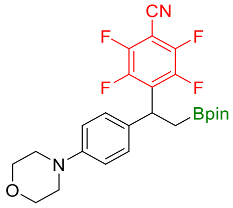

2,3,5,6-Tetrafluoro-4-(1-(4-morpholinophenyl)-2-(4,4,5,5-tetramethyl-1,3,2-dioxaborolan-2-yl)ethyl)benzonitrile (**3u**)

The title compound was prepared from 4-(4-vinylphenyl)morpholine (37.8 mg, 0.2 mmol) and 2,3,4,5,6-pentafluorobenzonitrile (38 uL, 0.3 mmol) according to general defluorinative arylboration procedure I. The crude residue was purified by flash chromatography (pentane/EA = 10:1, R_f = 0.25) to give the product as a colorless oil (58.6 mg, 58%).

^{1}H NMR (300 MHz, CDCl$_3$) δ 7.26 – 7.17 (m, 2H), 6.84 (d, *J* = 8.8 Hz, 2H), 4.69 (t, *J* = 8.6 Hz, 1H), 3.84 (dd, *J* = 5.6, 4.0 Hz, 4H), 3.12 (dd, *J* = 5.8, 4.0 Hz, 4H), 1.74 (dd, *J* = 8.8, 4.8 Hz, 2H), 1.10 (s, 12H).

^{13}C NMR (75 MHz, CDCl$_3$) δ 150.2, 147.0 (dm, *J* = 261.5 Hz), 144.7 (dm, *J* = 247.4 Hz), 132.7 (t, *J* = 15.7 Hz), 128.2, 115.6, 107.7, 91.3, 83.5, 66.8, 49.2, 36.3, 24.6, 24.5.

^{19}F NMR (282 MHz, CDCl$_3$) δ -133.30 (q, *J* = 8.8 Hz), -138.13 – -139.49 (m).

^{11}B NMR (96 MHz, CDCl$_3$) δ 32.5.

HRMS (ESI-TOF): m/z calcd. for [M+H$^+$] $C_{25}H_{27}BF_4N_2O_3$ 490.2165, found 490.2159.

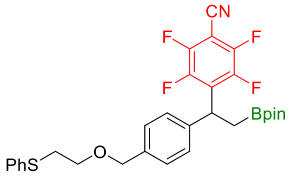

2,3,5,6-Tetrafluoro-4-(1-(4-((2-(phenylthio)ethoxy)methyl)phenyl)-2-(4,4,5,5-tetramethyl-1,3,2-dioxaborolan-2-yl)ethyl)benzonitrile (**3v**)

The title compound was prepared from phenyl(2-((4-vinylbenzyl)oxy)ethyl)sulfane (54.0 mg, 0.2 mmol) and 2,3,4,5,6-pentafluorobenzonitrile (38 uL, 0.3 mmol) according to general defluorinative arylboration procedure I. The crude residue was purified by flash chromatography (pentane/EA = 10:1, R_f = 0.30) to give the product as a colorless oil (86.9 mg, 76%).

^{1}H NMR (300 MHz, CDCl$_3$) δ 7.38 – 7.32 (m, 2H), 7.32 – 7.23 (m, 6H), 7.21 – 7.15 (m, 1H), 4.76 (t, *J* = 8.7 Hz, 1H), 4.49 (s, 2H), 3.65 (t, *J* = 6.9 Hz, 2H), 3.13 (t, *J* = 6.9 Hz, 2H), 1.87 – 1.69 (m, 2H), 1.11 (s, 12H).

^{13}C NMR (75 MHz, CDCl$_3$) δ 147.0 (dm, *J* = 260.5 Hz), 144.8 (dm, *J* = 248.5 Hz), 140.6, 137.2, 135.9, 132.2 (t, *J* = 15.7 Hz), 129.3, 128.9, 128.0, 127.5, 126.1, 107.6, 91.7, 83.6, 72.6, 68.8, 36.8, 33.3, 24.6, 24.5.

^{19}F NMR (282 MHz, CDCl$_3$) δ -132.98 – -133.31 (m), -138.51 (td, *J* = 16.3, 7.4 Hz).

^{11}B NMR (96 MHz, CDCl$_3$) δ 34.1.

HRMS (ESI-TOF): m/z calcd. for [M+Na$^+$] $C_{30}H_{30}BF_4NO_3S$ 593.1904, found 593.1915.

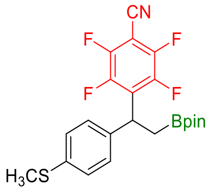

2,3,5,6-Tetrafluoro-4-(1-(4-(methylthio)phenyl)-2-(4,4,5,5-tetramethyl-1,3,2-dioxaborolan-2-yl)ethyl)benzonitrile (**3w**)

The title compound was prepared from methyl(4-vinylphenyl)sulfane (31 uL, 0.2 mmol) and 2,3,4,5,6-pentafluorobenzonitrile (38 uL, 0.3 mmol) according to general defluorinative arylboration procedure I. The crude residue was purified by flash chromatography (pentane/EA = 20:1, R_f = 0.20) to give the product as a colorless oil (52.2 mg, 58%).

^{1}H NMR (300 MHz, $CDCl_3$) δ 7.30 – 7.21 (m, 2H), 7.21 – 7.15 (m, 2H), 4.71 (t, *J* = 8.5 Hz, 1H), 2.45 (s, 3H), 1.85 – 1.65 (m, 2H), 1.10 (s, 12H).

^{13}C NMR (75 MHz, $CDCl_3$) δ 147.0 (dm, *J* = 261.5 Hz), 144.7 (dm, *J* = 248.1 Hz), 137.9, 137.6, 132.2 (t, *J* = 15.7 Hz), 127.9, 126.7, 107.6 (t, *J* = 3.5 Hz), 91.8, 83.6, 36.6, 24.5, 15.7, 14.8 (broad).

^{19}F NMR (282 MHz, $CDCl_3$) δ -132.94 – -133.29 (m), -138.34 – -138.90 (m).

^{11}B NMR (96 MHz, $CDCl_3$) δ 33.4.

HRMS (EI): m/z calcd. for [M] $C_{22}H_{22}BF_4NO_2S$ 451.13949, found 451.13978.

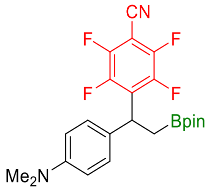

4-(1-(4-(Dimethylamino)phenyl)-2-(4,4,5,5-tetramethyl-1,3,2-dioxaborolan-2-yl)ethyl)-2,3,5,6-tetrafluorobenzonitrile (**3x**)

The title compound was prepared from *N*,*N*-dimethyl-4-vinylaniline (31 uL, 0.2 mmol) and 2,3,4,5,6-pentafluorobenzonitrile (38 uL, 0.3 mmol) according to general defluorinative arylboration procedure I. The crude residue was purified by flash chromatography (pentane/EA = 20:1, R_f = 0.35) to give the product as a colorless oil (40.1 mg, 45%).

^{1}H NMR (300 MHz, $CDCl_3$) δ 7.23 – 7.15 (m, 2H), 6.67 (d, *J* = 9.1 Hz, 2H), 4.67 (t, *J* = 8.7 Hz, 1H), 2.91 (s, 6H), 1.75 (dq, *J* = 9.1, 1.4 Hz, 2H), 1.11 (s, 6H), 1.10 (s, 6H).

^{13}C NMR (75 MHz, $CDCl_3$) δ 149.6, 146.9 (dm, *J* = 261.0 Hz), 144.5 (dm, *J* = 241.2 Hz), 133.1 (t, *J* = 15.7 Hz), 128.1, 112.6, 107.8 (t, *J* = 3.7 Hz), 91.2, 83.4, 40.6, 36.3, 24.6, 24.5.

^{19}F NMR (282 MHz, $CDCl_3$) δ -133.51 (td, *J* = 16.3, 7.7 Hz), -138.91 (td, *J* = 16.3, 7.4 Hz).

^{11}B NMR (96 MHz, $CDCl_3$) δ 33.3.

HRMS (ESI-TOF): m/z calcd. for $[M+H^+]$ $C_{23}H_{25}BF_4N_2O_2$ 448.2060, found 448.2060.

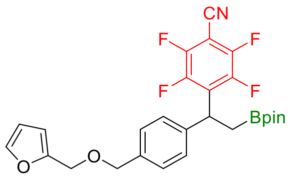

2,3,5,6-Tetrafluoro-4-(1-(4-((furan-2-ylmethoxy)methyl)phenyl)-2-(4,4,5,5-tetramethyl-1,3,2-dioxaborolan-2-yl)ethyl)benzonitrile (**3y**)

The title compound was prepared from 2-(((4-vinylbenzyl)oxy)methyl)furan (42.8 mg, 0.2 mmol) and 2,3,4,5,6-pentafluorobenzonitrile (38 uL, 0.3 mmol) according to general defluorinative arylboration procedure I. The crude residue was purified by flash chromatography (pentane/EA = 20:1, R_f = 0.20) to give the product as a colorless oil (56.9 mg, 55%).

^{1}H NMR (300 MHz, CDCl$_3$) δ 7.41 (dd, *J* = 1.8, 0.9 Hz, 1H), 7.29 (s, 4H), 6.39 – 6.26 (m, 2H), 4.76 (t, *J* = 8.7 Hz, 1H), 4.50 (s, 2H), 4.46 (d, *J* = 0.5 Hz, 2H), 1.87 – 1.68 (m, 2H), 1.10 (s, 12H).

^{13}C NMR (75 MHz, CDCl$_3$) δ 151.6, 146.9 (dm, *J* = 261.2 Hz), 144.9 (dm, *J* = 247.5 Hz), 142.8, 140.6, 137.1, 132.3 (t, *J* = 15.8 Hz), 128.2, 127.5, 110.2, 109.4, 107.6 (t, *J* = 3.7 Hz), 92.0, 83.6, 71.4, 63.9, 36.8, 24.6, 24.5.

^{19}F NMR (282 MHz, CDCl$_3$) δ -133.16 (td, *J* = 16.4, 7.6 Hz), -138.52 (td, *J* = 16.4, 7.3 Hz).

^{11}B NMR (96 MHz, CDCl$_3$) δ 33.5.

HRMS (ESI-TOF): m/z calcd. for [M+Na$^+$] $C_{27}H_{26}BF_4NO_4$ 537.1824, found 537.1840.

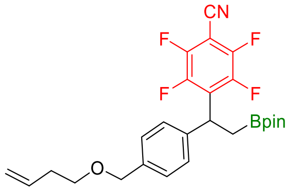

4-(1-(4-((But-3-en-1-yloxy)methyl)phenyl)-2-(4,4,5,5-tetramethyl-1,3,2-dioxaborolan-2-yl)ethyl)-2,3,5,6-tetrafluorobenzonitrile (**3z**)

The title compound was prepared from 1-((but-3-en-1-yloxy)methyl)-4-vinylbenzene (38.0 mg, 0.2 mmol) and 2,3,4,5,6-pentafluorobenzonitrile (38 uL, 0.3 mmol) according to general defluorinative arylboration procedure I. The crude residue was purified by flash chromatography (pentane/EA = 20:1, R_f = 0.30) to give the product as a colorless oil (62.4 mg, 64%).

^{1}H NMR (300 MHz, CDCl$_3$) δ 7.48 – 7.08 (m, 5H), 5.84 (ddt, *J* = 17.0, 10.2, 6.7 Hz, 1H), 5.19 – 5.01 (m, 2H), 4.78 (t, *J* = 8.6 Hz, 1H), 4.50 (s, 2H), 3.52 (t, *J* = 6.7 Hz, 2H), 2.43 – 2.31 (m, 2H), 1.89 – 1.74 (m, 2H), 1.12 (s, 12H).

^{13}C NMR (75 MHz, CDCl$_3$) δ 147.0 (dm, *J* = 261.5 Hz), 144.7 (dm, *J* = 248.1 Hz), 140.4, 137.6, 135.1, 132.3 (t, *J* = 15.8 Hz), 127.9, 127.4, 116.3, 107.6 (t, *J* = 3.7 Hz), 91.7, 72.4, 69.6, 36.8, 34.1, 24.5, 24.5, 14.8 (broad).

^{19}F NMR (282 MHz, CDCl$_3$) δ -133.01 – -133.45 (m), -138.53 (td, *J* = 16.4, 7.6 Hz).

^{11}B NMR (96 MHz, CDCl$_3$) δ 33.0.

HRMS (ESI-TOF): m/z calcd. for [M+Na$^+$] $C_{26}H_{28}BF_4NO_3$ 511.2032, found 511.2043.

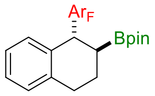

trans-2,3,5,6-Tetrafluoro-4-(2-(4,4,5,5-tetramethyl-1,3,2-dioxaborolan-2-yl)-1,2,3,4-tetrahydronaphthalen-1-yl)benzonitrile (**3aa**)

The title compound was prepared from 1,2-dihydronaphthalene (26 uL, 0.2 mmol) and 2,3,4,5,6-pentafluorobenzonitrile (38 uL, 0.3 mmol) according to general defluorinative arylboration procedure I. The crude residue was purified by flash chromatography (pentane/EA = 20:1, R_f = 0.40) to give the product as a colorless oil (28.1 mg, 33%).

^{1}H NMR (300 MHz, $CDCl_3$) δ 7.13 (d, *J* = 3.6 Hz, 2H), 7.02 (dt, *J* = 8.7, 4.3 Hz, 1H), 6.63 (d, *J* = 7.5 Hz, 1H), 4.68 (d, *J* = 10.7 Hz, 1H, *trans*), 3.00 – 2.81 (m, 2H), 2.22 – 2.08 (m, 1H), 1.84 – 1.67 (m, 2H), 1.17 (s, 6H), 1.14 (s, 6H).

^{13}C NMR (75 MHz, $CDCl_3$) δ 146.9 (dm, *J* = 261.5 Hz), 145.0 (dm, *J* = 252.8 Hz), 137.1, 135.8, 133.2 (t, *J* = 15.5 Hz), 129.5, 127.4, 126.5, 126.2, 107.8, 91.1, 83.6, 37.5, 30.1, 24.8, 24.7, 24.4, 14.1 (broad).

^{19}F NMR (282 MHz, $CDCl_3$) δ -133.56 (td, *J* = 16.4, 7.2 Hz), -137.02 – -137.63 (m).

^{11}B NMR (96 MHz, $CDCl_3$) δ 33.5.

HRMS (EI): m/z calcd. for [M] $C_{23}H_{22}BF_4NO_2$ 431.16742, found 431.16777.

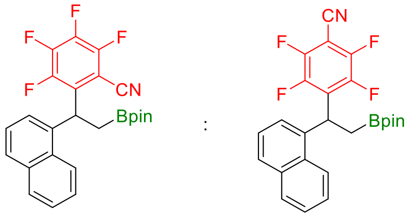

***o*-3bb** : ***p*-3bb** = 72:28

2,3,4,5-Tetrafluoro-6-(1-(naphthalen-1-yl)-2-(4,4,5,5-tetramethyl-1,3,2-dioxaborolan-2-yl)ethyl)benzonitrile (*o*-**3bb**)

The title compound was prepared from 1-vinylnaphthalene (30 uL, 0.2 mmol) and 2,3,4,5,6-pentafluorobenzonitrile (38 uL, 0.3 mmol) according to general defluorinative arylboration procedure I. The crude residue was purified by flash chromatography (pentane/EA = 20:1, R_f = 0.35) to give the product as a colorless oil (30.1 mg, 33%).

^{1}H NMR (300 MHz, $CDCl_3$, mixture of ***o*-3bb** and ***p*-3bb,** the ratio of the isomers is 72:28**)** δ 8.00 (d, *J* = 6.3 Hz, 1H), 7.89 – 7.71 (m, 3H), 7.57 – 7.43 (m, 3H), 5.42 (t, *J* = 8.1 Hz, 1H), 1.94 (ddd, *J* = 22.9, 15.0, 7.8 Hz, 2H), 1.14 (s, 6H), 1.10 (s, 6H).

^{13}C NMR (75 MHz, $CDCl_3$, major isomer ***o*-3bb)** δ 135.8, 133.9, 131.4, 129.2, 128.5, 126.6, 125.5, 125.0, 122.6, 110.7, 83.6, 37.1, 24.6, 24.6, 14.1 (broad).

^{19}F NMR (282 MHz, $CDCl_3$, isomer ***o*-3bb)** δ -130.70 (ddd, *J* = 20.6, 11.1, 6.9 Hz), -135.81 – -136.35 (m), -144.95 (td, *J* = 21.0, 6.9 Hz), -155.22 (td, *J* = 20.6, 4.6 Hz).

^{19}F NMR (282 MHz, $CDCl_3$, isomer ***p*-3bb)** δ -132.86 – -133.28 (m), -137.64 (td, *J* = 16.4, 7.6 Hz).

^{11}B NMR (96 MHz, $CDCl_3$) δ 33.5.

HRMS (ESI-TOF): m/z calcd. for [M+H$^+$] $C_{25}H_{22}BF_4NO_2$ 455.1794, found 455.1797.

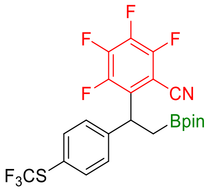

2,3,4,5-Tetrafluoro-6-(2-(4,4,5,5-tetramethyl-1,3,2-dioxaborolan-2-yl)-1-(4-((trifluoromethyl)thio)phenyl)ethyl) benzonitrile (**3cc**)

The title compound was prepared from (trifluoromethyl)(4-vinylphenyl)sulfane (37 uL, 0.2 mmol) and 2,3,4,5,6-pentafluorobenzonitrile (38 uL, 0.3 mmol) according to general defluorinative arylboration procedure I. The crude residue was purified by flash chromatography (pentane/EA = 20:1, R_f = 0.35) to give the product as a white solid (54.6 mg, 54%).

^{1}H NMR (300 MHz, $CDCl_3$) δ 7.60 (d, *J* = 8.3 Hz, 2H), 7.43 (d, *J* = 8.2 Hz, 2H), 4.75 (t, *J* = 8.5 Hz, 1H), 1.82 (qdd, *J* = 16.0, 8.5, 1.5 Hz, 2H), 1.10 (d, *J* = 1.7 Hz, 12H).

^{13}C NMR (75 MHz, $CDCl_3$) δ 144.3, 136.5, 129.5 (q, *J* = 308.0 Hz), 128.8 (d, *J* = 2.0 Hz), 123.3 (q, *J* = 2.0 Hz), 111.0 (m), 98.3, 83.7, 41.6, 24.5, 14.9 (broad).

^{19}F NMR (282 MHz, $CDCl_3$) δ -42.78, -130.47 (ddd, *J* = 21.4, 11.4, 7.2 Hz), -136.60 (ddd, *J* = 21.4, 11.4, 5.3 Hz), -144.32 (td, *J* = 20.6, 6.9 Hz), -154.44 (td, *J* = 20.2, 5.0 Hz).

^{11}B NMR (96 MHz, $CDCl_3$) δ 33.3.

HRMS (EI): m/z calcd. for [M] $C_{22}H_{19}BF_7NO_2S$ 505.11123, found 505.11174.

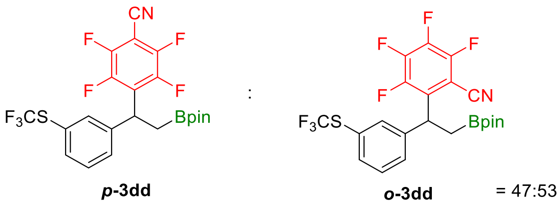

The title compound was prepared from (trifluoromethyl)(3-vinylphenyl)sulfane (36 uL, 0.2 mmol) and 2,3,4,5,6-pentafluorobenzonitrile (38 uL, 0.3 mmol) according to general defluorinative arylboration procedure I. The crude residue was purified by flash chromatography (pentane/EA = 20:1, R_f = 0.35) to give the product as a colorless oil (38.1 mg, 38%).

^{1}H NMR (300 MHz, $CDCl_3$, mixture of ***p*-3dd** and ***o*-3dd**, ratio of two isomers is 47:53**)** δ 7.65 (d, *J* = 8.5 Hz, 1H), 7.54 (d, *J* = 7.6 Hz, 1H), 7.51 – 7.42 (m, 1H), 7.37 (t, *J* = 7.7 Hz, 1H), 4.77 (q, *J* = 8.0 Hz, 1H), 1.94 – 1.67 (m, 2H), 1.13 – 1.07 (m, 12H).

^{13}C NMR (75 MHz, $CDCl_3$) δ 142.8, 135.3, 135.1, 130.2, 129.8, 129.5 (q, *J* = 308.3 Hz), 124.9, 83.7, 41.5, 24.6, 24.5.

^{19}F NMR (282 MHz, $CDCl_3$, isomer ***o*-3dd)** δ -42.60 (d, *J* = 25.9 Hz), -130.53 (ddd, *J* = 21.4, 11.4, 6.9 Hz), -136.73 (ddd, *J* = 20.6, 11.4, 5.3 Hz), -144.36 (td, *J* = 20.2, 7.2 Hz), -154.46 (td, *J* = 20.6, 4.6 Hz).

^{19}F NMR (282 MHz, $CDCl_3$, isomer ***p*-3dd)** δ -42.60 (d, *J* = 25.9 Hz), -132.63 (td, *J* = 16.8, 7.6 Hz), -138.55 (td, *J* = 16.0, 6.9 Hz).

^{11}B NMR (96 MHz, $CDCl_3$) δ 33.2.

HRMS (EI): m/z calcd. for [M] $C_{22}H_{19}BF_7NO_2S$ 505.11123, found 505.11178.

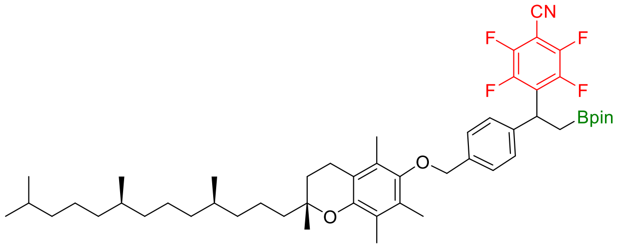

2,3,5,6-Tetrafluoro-4-(2-(4,4,5,5-tetramethyl-1,3,2-dioxaborolan-2-yl)-1-(4-((((*R*)-2,5,7,8-tetramethyl-2-((4*R*,8*R*)-4,8,12-trimethyltridecyl)chroman-6-yl)oxy)methyl)phenyl)ethyl)benzonitrile (**4a**)

The title compound was prepared from (*R*)-2,5,7,8-tetramethyl-2-((4*R*,8*R*)-4,8,12-trimethyltridecyl)-6-((4-vinylbenzyl)oxy)chromane (109.2 mg, 0.2 mmol) and 2,3,4,5,6-pentafluorobenzonitrile (38 uL, 0.3 mmol) according to general defluorinative arylboration procedure I. The crude residue was purified by flash chromatography (pentane/EA = 20:1, R_f = 0.30) to give the product as a colorless oil (109.7 mg, 65%).

^{1}H NMR (300 MHz, CDCl$_3$) δ 7.46 (d, *J* = 8.4 Hz, 2H), 7.38 (d, *J* = 8.1 Hz, 2H), 4.82 (t, *J* = 8.6 Hz, 1H), 4.68 (s, 2H), 2.60 (t, *J* = 6.7 Hz, 2H), 2.21 (s, 3H), 2.16 (s, 3H), 2.12 (s, 3H), 1.92 – 1.73 (m, 4H), 1.56 (dq, *J* = 13.1, 6.6 Hz, 3H), 1.47 – 1.24 (m, 15H), 1.14 (d, *J* = 2.1 Hz, 18H), 0.92 – 0.85 (m, 12H).

^{13}C NMR (75 MHz, CDCl$_3$) δ 148.0, 147.9, 147.1 (dm, *J* = 262.1 Hz), 144.7 (d, *J* = 246.5 Hz), 140.7, 137.2, 132.3 (t, *J* = 15.6 Hz), 128.0, 127.8, 127.5, 125.8, 122.9, 117.6, 107.5 (t, *J* = 3.3 Hz), 91.8, 83.5, 74.8, 74.1, 40.0, 40.0, 39.3, 37.6, 37.4, 37.4, 37.3, 36.9, 32.8, 32.7, 31.3, 27.9, 24.8, 24.6, 24.5, 24.4, 23.8, 22.7, 22.6, 21.0, 20.6, 19.7, 19.7, 19.6, 14.9 (broad), 12.8, 11.9, 11.8.

^{19}F NMR (282 MHz, CDCl$_3$) δ -133.20 (td, *J* = 16.6, 7.4 Hz), -138.46 (td, *J* = 16.3, 7.4 Hz).

^{11}B NMR (96 MHz, CDCl$_3$) δ 32.0.

HRMS (EI): m/z calcd. for [M] $C_{51}H_{70}BF_4NO_4$ 847.53285, found 847.53079.

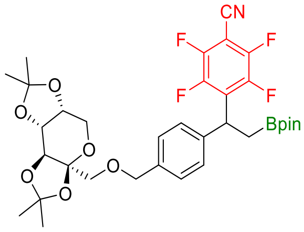

2,3,5,6-Tetrafluoro-4-(2-(4,4,5,5-tetramethyl-1,3,2-dioxaborolan-2-yl)-1-(4-(((((*3aS*,*5aR*,*8aR*,*8bS*)-2,2,7,7-tetramethyltetrahydro-*3aH*-bis([1,3]dioxolo)[4,5-b:4',5'-d]pyran-3a-yl)methoxy)methyl)phenyl)ethyl)benzonitrile (**4b**)

The title compound was prepared from (*3aS*,*5aR*,*8aR*,*8bS*)-2,2,7,7-tetramethyl-3a-(((4-vinylbenzyl)oxy)methyl)tetrahydro-5H-bis([1,3]dioxolo)[4,5-b:4',5'-d]pyran (131.6 mg, 0.3 mmol) and 2,3,4,5,6-pentafluorobenzonitrile (67 uL, 0.53 mmol) according to general defluorinative arylboration procedure I. The crude residue was purified by flash chromatography (pentane/EA = 10:1, R_f = 0.30) to give the product as a colorless oil (189.9 mg, 80%).

^{1}H NMR (300 MHz, CDCl$_3$) δ 7.26 (d, *J* = 1.6 Hz, 4H), 4.73 (t, *J* = 8.6 Hz, 1H), 4.66 – 4.48 (m, 3H), 4.40 (dd, *J* = 2.6, 1.7 Hz, 1H), 4.21 (dd, *J* = 7.9, 1.1 Hz, 1H), 3.89 (dd, *J* = 12.9, 1.9 Hz, 1H), 3.70 (dd, *J* = 12.9, 0.9 Hz, 1H), 3.61 – 3.50 (m, 2H), 1.75 (ddt, *J* = 24.9, 15.9, 8.6 Hz, 2H), 1.54 – 1.50 (m, 3H), 1.41 – 1.36 (m, 6H), 1.31 (s, 3H), 1.08 (s, 12H).

^{13}C NMR (75 MHz, CDCl$_3$) δ 147.0 (dm, *J* = 261.2 Hz), 144.7 (dm, *J* = 248.1 Hz), 140.3, 140.3, 137.2, 132.2 (t, *J* = 15.5 Hz), 127.8, 127.7, 127.3, 107.5 (t, *J* = 3.7 Hz), 102.6, 91.7 (t, *J* = 17.2 Hz), 83.5, 73.1, 71.5, 70.9, 70.1, 70.0, 60.9, 36.8, 26.5, 25.7, 25.3, 24.5, 24.5, 23.9, 14.8 (broad).

^{19}F NMR (282 MHz, CDCl$_3$) δ -131.05 – -135.12 (m), -138.60 (ddd, *J* = 20.6, 10.7, 6.9 Hz).

^{11}B NMR (96 MHz, CDCl$_3$) δ 30.1.

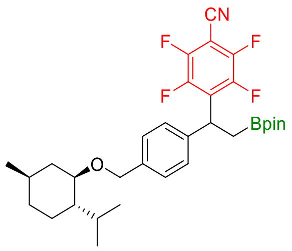

2,3,5,6-Tetrafluoro-4-(1-(4-((((*1R*,*2S*,*5R*)-2-isopropyl-5-methylcyclohexyl)oxy)methyl)phenyl)-2-(4,4,5,5-tetramethyl-1,3,2-dioxaborolan-2-yl)ethyl)benzonitrile (**4c**)

The title compound was prepared from 1-((((*1R*,*2S*,*5R*)-2-isopropyl-5-methylcyclohexyl)oxy)methyl)-4-vinylbenzene (54.4 mg, 0.2 mmol) and 2,3,4,5,6-pentafluorobenzonitrile (38 uL, 0.3 mmol) according to general defluorinative arylboration procedure I. The crude residue was purified by flash chromatography (pentane/EA = 20:1, R_f = 0.30) to give the product as a colorless oil (55.2 mg, 48%).

^{1}H NMR (300 MHz, CDCl$_3$) δ 7.27 (d, *J* = 6.6 Hz, 4H), 4.75 (t, *J* = 8.6 Hz, 1H), 4.61 (dd, *J* = 11.5, 1.1 Hz, 1H), 4.34 (d, *J* = 11.5 Hz, 1H), 3.20 – 3.06 (m, 1H), 2.31 – 2.11 (m, 2H), 1.77 (ddd, *J* = 7.9, 3.9, 2.6 Hz, 2H), 1.62 (d, *J* = 3.9 Hz, 2H), 1.28 – 1.26 (m, 1H), 1.10 (s, 12H), 0.98 – 0.82 (m, 10H), 0.68 (dd, *J* = 7.0, 3.0 Hz, 3H).

^{13}C NMR (75 MHz, CDCl$_3$) δ 147.0 (dm, *J* = 261.5 Hz), 144.7 (dm, *J* = 248.4 Hz), 140.3, 138.3, 132.4 (t, *J* = 15.7 Hz), 128.2, 127.3, 107.6 (t, *J* = 3.7 Hz), 91.7, 83.5, 78.9, 69.9, 48.2, 40.3, 34.5, 31.5, 25.5, 24.6, 24.5, 23.2, 22.3, 21.0, 16.0, 14.7 (broad).

^{19}F NMR (282 MHz, CDCl$_3$) δ -133.27 (td, *J* = 16.4, 7.6 Hz), -138.51 (dt, *J* = 15.3, 9.5 Hz).

^{11}B NMR (96 MHz, CDCl$_3$) δ 33.4.

HRMS (ESI-TOF): m/z calcd. for [M+Na$^+$] $C_{32}H_{40}BF_4NO_3$ 595.2971, found 595.2971.

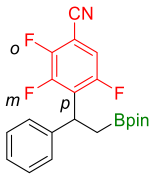

p:*m*:*o* = 38:32:30 (**5a**)

The title compound was prepared from styrene (23 uL, 0.2 mmol) and 2,3,4,5-tetrafluorobenzonitrile (36 uL, 0.3 mmol) according to general defluorinative arylboration procedure I. The crude residue was purified by flash chromatography (pentane/EA = 20:1, R_f = 0.35) to give the product as a colorless oil (34.9 mg, 45%).

^{1}H NMR (300 MHz, CDCl$_3$, mixture of **5a)** δ 7.46 – 6.83 (m, 6H), 4.78 (t, *J* = 8.5 Hz, 1H), 1.84 (ddd, *J* = 8.7, 5.5, 1.6 Hz, 2H), 1.10 (s, 12H).

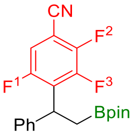

2,3,5-Trifluoro-4-(1-phenyl-2-(4,4,5,5-tetramethyl-1,3,2-dioxaborolan-2-yl)ethyl)benzonitrile (*p*-**5a**)

^{19}F NMR (282 MHz, CDCl$_3$) δ -113.91 (ddd, *J* = 13.7, 9.2, 5.0 Hz, F^1), -131.82 (dd, *J* = 20.2, 5.7 Hz, F^2), -134.75 (ddd, *J* = 19.1, 14.1, 4.6 Hz, F^3).

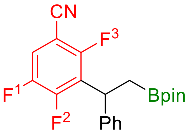

2,4,5-Trifluoro-3-(1-phenyl-2-(4,4,5,5-tetramethyl-1,3,2-dioxaborolan-2-yl)ethyl)benzonitrile (*m*-**5a**)

^{19}F NMR (282 MHz, CDCl$_3$) δ -107.00 (dt, *J* = 14.5, 7.2 Hz, F^3), -122.37 (ddd, *J* = 21.4, 9.9, 6.9 Hz, F^1), -140.40 (ddd, *J* = 19.8, 13.7, 6.1 Hz, F^2).

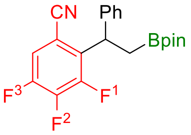

3,4,5-Trifluoro-2-(1-phenyl-2-(4,4,5,5-tetramethyl-1,3,2-dioxaborolan-2-yl)ethyl)benzonitrile (*o*-**5a**)

^{19}F NMR (282 MHz, CDCl$_3$) δ -130.84 (dd, *J* = 20.2, 8.8 Hz, F^1), -134.03 (dt, *J* = 21.4, 9.2 Hz, F^3), -149.55 (td, *J* = 20.2, 6.9 Hz, F^2).
HRMS (EI): m/z calcd. for [M] $C_{21}H_{21}BF_3NO_2$ 387.16120, found 387.16171.

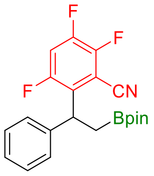

2,3,5-Trifluoro-6-(1-phenyl-2-(4,4,5,5-tetramethyl-1,3,2-dioxaborolan-2-yl)ethyl)benzonitrile (**5b**)

The title compound was prepared from styrene (23 uL, 0.2 mmol) and 2,3,5,6-tetrafluorobenzonitrile (52.5 mg, 0.3 mmol) according to general defluorinative arylboration procedure I with Xantphos (11.6 mg, 10 mol%) as ligand and n-heptane as solvent, stirred at 60 ºC for 16 h. The crude residue was purified by flash chromatography (pentane/EA = 20:1, R_f = 0.35) to give the product as a colorless oil (20.8 mg, 27%).

^{1}H NMR (300 MHz, CDCl$_3$) δ 7.37 (d, *J* = 8.2 Hz, 2H), 7.33 – 7.26 (m, 2H), 7.24 – 7.17 (m, 1H), 7.09 (td, *J* = 9.8, 6.7 Hz, 1H), 4.79 – 4.65 (m, 1H), 1.82 (dd, *J* = 8.4, 1.7 Hz, 2H), 1.11 (s, 6H), 1.10 (s, 6H).

^{13}C NMR (75 MHz, CDCl$_3$) δ 141.9, 134.0, 128.5, 127.5 (d, *J* = 2.0 Hz), 127.0, 111.7 (m), 111.1 (dd, *J* = 29.1, 20.4 Hz), 83.5, 41.6, 24.6, 24.5.

^{19}F NMR (282 MHz, CDCl$_3$) δ -111.56 (ddd, *J* = 15.3, 9.9, 5.3 Hz), -133.63 (ddd, *J* = 21.4, 9.9, 5.0 Hz), -134.70 (ddd, *J* = 21.4, 14.5, 6.9 Hz).

^{11}B NMR (96 MHz, CDCl$_3$) δ 33.7.

HRMS (EI): m/z calcd. for [M] $C_{21}H_{21}BF_3NO_2$ 387.16120, found 387.16118.

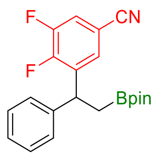

3,4-Difluoro-5-(1-phenyl-2-(4,4,5,5-tetramethyl-1,3,2-dioxaborolan-2-yl)ethyl)benzonitrile (**5c**)

The title compound was prepared from styrene (23 uL, 0.2 mmol) and 3,4,5-trifluorobenzonitrile (23 uL, 0.3 mmol) according to general defluorinative arylboration procedure I. The crude residue was purified by flash chromatography (pentane/EA = 20:1, R_f = 0.35) to give the product as a colorless oil (40.9 mg, 56%).

^{1}H NMR (300 MHz, $CDCl_3$) δ 7.46 – 7.35 (m, 3H), 7.34 – 7.27 (m, 2H), 7.25 – 7.17 (m, 1H), 7.08 (td, *J* = 8.9, 7.4 Hz, 1H), 4.82 (t, *J* = 8.5 Hz, 1H), 1.86 (dd, *J* = 8.5, 1.7 Hz, 2H), 1.09 (s, 12H).

^{13}C NMR (75 MHz, $CDCl_3$) δ 153.7 (dd, *J* = 258.5, 13.8 Hz), 149.6 (d, *J* = 252.1, 13.5 Hz), 142.1, 140.2 (d, *J* = 12.1 Hz), 129.6 (dd, *J* = 7.7, 4.4 Hz), 128.4, 127.6 (d, *J* = 2.4 Hz), 126.9, 117.1 (m), 115.6 (d, *J* = 19.2 Hz), 109.5 (dd, *J* = 5.9, 3.7 Hz), 83.4, 42.1 (d, *J* = 2.0 Hz), 24.5, 24.5.

^{19}F NMR (282 MHz, $CDCl_3$) δ -127.27 (ddd, *J* = 20.6, 9.2, 4.6 Hz), -135.29 (dd, *J* = 20.6, 7.6 Hz).

^{11}B NMR (96 MHz, $CDCl_3$) δ 33.4.

HRMS (ESI-TOF): m/z calcd. for [M+Na$^+$] $C_{21}H_{22}BF_2NO_2$ 391.1645, found 391.1651.

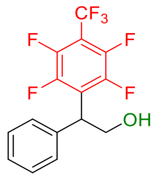

2-Phenyl-2-(2,3,5,6-tetrafluoro-4-(trifluoromethyl)phenyl)ethan-1-ol (**5e**)

The title compound was prepared from styrene (23 uL, 0.2 mmol) and 1,2,3,4,5-pentafluoro-6-(trifluoromethyl)benzene (42 uL, 0.3 mmol) according to general defluorinative arylboration procedure II. The crude residue was purified by flash chromatography (pentane/EA = 5:1, R_f = 0.40) to give the product as a colorless oil (25.8 mg, 38%).

^{1}H NMR (300 MHz, $CDCl_3$) δ 7.40 – 7.35 (m, 1H), 7.35 – 7.26 (m, 4H), 4.70 (t, *J* = 8.0 Hz, 1H), 4.37 (t, *J* = 1.4 Hz, 1H), 4.35 (t, *J* = 1.4 Hz, 1H), 1.78 (s, 1H).

^{13}C NMR (75 MHz, $CDCl_3$) δ 145.5 (dm, *J* = 240.7 Hz), 144.2 (dm, *J* = 244.4 Hz), 137.3, 129.1, 127.9, 125.1 (t, *J* = 16.2 Hz), 120.8 (q, *J* = 274.3 Hz), 63.4 (t, *J* = 4.0 Hz), 45.0 (t, *J* = 1.7 Hz).

^{19}F NMR (282 MHz, $CDCl_3$) δ -56.38 (t, *J* = 21.4 Hz), -139.90 (td, *J* = 15.6, 6.5 Hz), -140.26 – -140.74 (m).

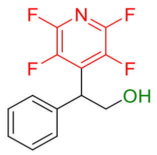

2,3,5,6-Tetrafluoro-4-(1-phenyl-2-(4,4,5,5-tetramethyl-1,3,2-dioxaborolan-2-yl)ethyl)pyridine (**5f**)

The title compound was prepared from styrene (23 uL, 0.2 mmol) and perfluoropyridine (33 uL, 0.3 mmol) according to general defluorinative arylboration procedure II. The crude residue was purified by flash chromatography (pentane/EA = 5:1, R_f = 0.40) to give the product as a colorless oil (28.0 mg, 52%).

^{1}H NMR (300 MHz, $CDCl_3$) δ 7.40 – 7.35 (m, 1H), 7.35 – 7.27 (m, 4H), 4.78 – 4.64 (m, 1H), 4.46 – 4.28 (m, 2H), 1.83 (s, 1H).

^{13}C NMR (75 MHz, $CDCl_3$) δ 140.4 (dm, *J* = 252.1 Hz), 140.5 (dm, *J* = 246.5 Hz), 138.7, 134.3, 129.2, 128.1, 128.0 (t, *J* = 1.4 Hz), 63.2 (t, *J* = 3.7 Hz), 45.5.

^{19}F NMR (282 MHz, $CDCl_3$) δ -90.28 – -92.16 (m), -142.27 – -144.37 (m).

HRMS (EI): m/z calcd. for [M] $C_{13}H_9F_4NO$ 271.06148, found 271.06134.

5. Derivatization of the B–C bond.

4.1 Deborylative oxidation.

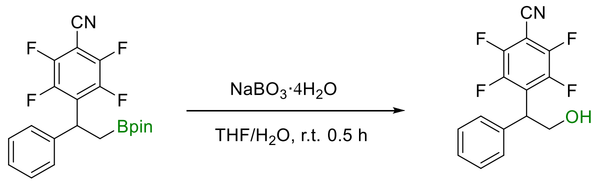

2,3,5,6-Tetrafluoro-4-(2-hydroxy-1-phenylethyl)benzonitrile (**6a**)

The title compound was synthesized according to the following procedure: To the boration product **3a** (80.1 mg, 0.2 mmol) in THF (2.5 mL) and water (2.5 mL) was added $NaBO_3·4H_2O$ (153.0 mg. 5 equiv). The reaction mixture was stirred vigorously for 30 min at room temperature. The reaction mixture was quenched with water and then extracted with ethyl acetate (5 mL). The combined organic layers were washed with brine (15 mL), dried over Na_2SO_4 and concentrated. The crude product was purified by column chromatography on silica gel to afford the corresponding product **6a** as colorless oil (47.0 mg, 80%).

^{1}H NMR (300 MHz, CDCl$_3$) δ 7.39 – 7.26 (m, 5H), 4.71 (t, *J* = 8.0 Hz, 1H), 4.43 – 4.28 (m, 2H), 2.04 (s, 1H).

^{13}C NMR (75 MHz, CDCl$_3$) δ 147.2 (ddt, *J* = 262.2, 17.8, 4.0 Hz), 145.2 (dm, *J* = 247.7 Hz), 137.0, 129.2, 128.1, 127.9 (t, *J* = 1.5 Hz), 107.5 (t, *J* = 3.7 Hz), 92.7, 63.2, 45.3 (t, *J* = 1.9 Hz).

^{19}F NMR (282 MHz, CDCl$_3$) δ -132.25 – -132.65 (m), -138.51 (td, *J* = 16.4, 7.2 Hz).

HRMS (EI): m/z calcd. for [M] $C_{15}H_8F_4O_2$ 296.04549, found 296.04629.

4.2 BF_3K preparation.

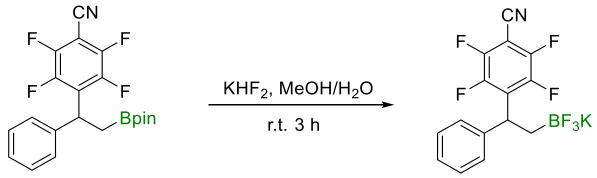

2,3,5,6-tetrafluoro-4-(1-phenyl-2-(trifluoro-l4-boraneyl)ethyl)benzonitrile, potassium salt (**6b**)

The title compound was synthesized according to the following procedure: To the boration product **3a** (81.0 mg, 0.2 mmol) in MeOH (5.0 mL) and H_2O (0.4 mL) was added KHF_2 (140.0 mg, 4.5 M). The reaction mixture was stirred vigorously for 3 h at room temperature. The resulting slurry was stirred concentrated, then placed under high vacuum. The dried solids were triturated with hot acetone and filtered to remove inorganic salts. The resulting filtrate was concentrated to a minimal volume and wash with n-pentane/EA (90:10, 5 x 2 mL) was added to afford **6b** as a white solid (64.2 mg, 83%).

^{1}H NMR (300 MHz, DMSO) δ 7.32 – 7.23 (m, 4H), 7.20 – 7.08 (m, 1H), 4.51 (dd, *J* = 11.6, 4.5 Hz, 1H).

^{13}C NMR (75 MHz, DMSO) δ 144.3, 135.0 (t, *J* = 16.6 Hz), 128.2, 127.4, 126.1, 108.4, 38.1.

^{19}F NMR (282 MHz, DMSO) δ -136.13 – -136.59 (m), -137.15, -139.51 (dd, *J* = 20.2, 9.5 Hz).

^{11}B NMR (96 MHz, DMSO) δ 3.8.

4.3 Deborylative bromination.

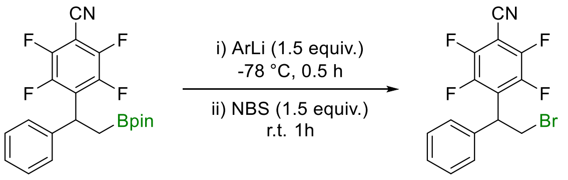

3a 6c

The title compound was synthesized according to the following procedure:[1] To a solution of 1-bromo-3,5-bis(trifluoromethyl)benzene (44.0 mg, 1.50 equiv) in THF (2 mL) at –78 °C was added *n*-BuLi (1.6 M in hexane; 96 µL, 1.50 equiv) dropwise. The mixture was stirred at –78 °C for 1 h, at which point a solution of **3a** (40.5 mg, 0.1 mmol) in THF (1.0 mL) was added. The mixture was stirred at –78 °C for 30 min. *N*-bromosuccinimide (26.7 mg, 1.50 equiv) was added as a solid to it and stirred at this temperature for another 5 minutes, and then allowed to warm to room temperature and stirred for 1 hour. Then the reaction was quenched with a saturated aqueous sodium thiosulfate solution (2.0 mL). The resulting mixture was diluted with water (5.0 mL) and ethyl acetate (5.0 mL). The aqueous layer was extracted with ethyl acetate. The combined organic layers were dried over Na_2SO_4, filtered and concentrated. The crude product was purified by flash column chromatography (Pentane:EtOAc = 50:1, Rf = 0.3) to obtain the desired product **6c** (16.0 mg, 45%) as a colorless oil.

^{1}H NMR (300 MHz, CDCl$_3$) δ 7.41 – 7.27 (m, 5H), 4.88 (dd, *J* = 11.1, 6.0 Hz, 1H), 4.21 (ddt, *J* = 11.2, 10.4, 1.0 Hz, 1H), 3.96 (ddt, *J* = 10.4, 6.1, 1.1 Hz, 1H).

^{13}C NMR (75 MHz, CDCl$_3$) δ 137.5, 129.4, 128.6, 127.4 (t, *J* = 2.0 Hz), 107.3, 45.5, 31.2.

^{19}F NMR (282 MHz, CDCl$_3$) δ -131.63 – -131.98 (m), -138.85 (td, *J* = 16.4, 7.2 Hz).

HRMS (EI): m/z calcd. for [M] $C_{15}H_8BrF_4$ 356.97708, found 356.97646.

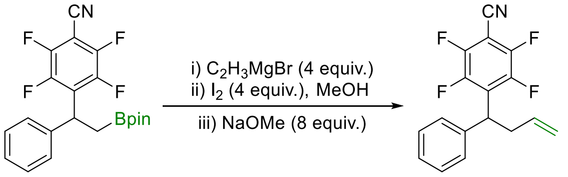

3a 6d

The title compound was synthesized according to the following procedure:[2] to an oven-dried round bottom flask containing a stirring bar was added a solution of **3a** (60.7 mg, 0.15 mmol) in THF (2 mL) and subsequently vinylmagnesium chloride (1.6 M, 0.38 mL, 4.0 equiv.) was added dropwise. The mixture was stirred as room temperature for 30 min. To the above solution at -78 °C I_2 (151 mg, 2.0 equiv.) in methanol (3.0 mL) was added dropwise. The reaction mixture was allowed to stir 30 min at the same temperature followed by dropwise addition of a solution of NaOMe (66 mg, 8.0 equiv) in methanol (3 mL). After warming to room temperature, the mixture was stirred for another 1.5 h, diluted with EtOAc (10 mL) and washed sequentially with 10% aqueous solution of $Na_2S_2O_3$ (5 mL). Then, the mixture was extracted with EtOAc (10 mL), dried over Na_2SO_4, filtered and concentrated under reduced pressure. The crude product was purified by column chromatography on silica gel to afford the corresponding product **6d** as colorless oil (35.2 mg, 77%).

^{1}H NMR (300 MHz, CDCl$_3$) δ 7.38 – 7.24 (m, 5H), 5.83 – 5.64 (m, 1H), 5.18 – 4.95 (m, 2H), 4.59 (t, *J* = 8.3 Hz, 1H), 3.01 (ddt, *J* = 8.3, 7.0, 1.3 Hz, 2H).

^{13}C NMR (75 MHz, CDCl$_3$) δ 147.13 (dm, *J* = 261.9 Hz), 144.8 (dm, *J* = 247.7 Hz), 139.4, 134.7, 129.9 (t, *J* = 15.8 Hz), 128.9, 127.6 (t, *J* = 1.7 Hz), 127.6, 118.0, 107.5 (t, *J* = 3.7 Hz), 92.4, 41.7 (t, *J* = 1.7 Hz), 36.1 (t, *J* = 3.2 Hz).

^{19}F NMR (282 MHz, CDCl$_3$) δ -132.40 – -132.70 (m), -138.55 (td, *J* = 16.0, 6.9 Hz).

HRMS (EI): m/z calcd. for [M] $C_{17}H_{11}NF_4$ 305.08221, found 305.08199.

6. X-ray crystal structure analysis of 3c’

Data were collected on a Bruker Kappa APEX II Duo diffractometer. The structure was solved by direct methods (SHELXS-97: Sheldrick, G. M. *Acta Cryst.* **2008**, *A64*, 112.) and refined by full-matrix least-squares procedures on F^2 (SHELXL-2018: Sheldrick, G. M. *Acta Cryst.* **2015**, *C71*, 3.). XP (Bruker AXS) was used for graphical representation. Crystal data of **3c’**: $C_{18}H_{17}BrF_3NO$, M = 400.23, monoclinic, space group $P2_1/c$, a = 4.8463(2), b = 20.8699(10), c = 18.5686(10) Å, b = 93.7087(19)°, V = 1874.13(16) Å^3, T = 150(2) K, Z = 4, 27878 reflections measured, 5064 independent reflections (R_{int} = 0.0292), final R values ($I > 2\sigma(I)$): R_1 = 0.0343, wR_2 = 0.0766, final R values (all data): R_1 = 0.0499, wR_2 = 0.0830, 209 parameters.

CCDC 2203611 contains the supplementary crystallographic data for this paper. These data are provided free of charge by the joint Cambridge Crystallographic Data Centre and Fachinformationszentrum Karlsruhe Access Structures service www.ccdc.cam.ac.uk/structures.

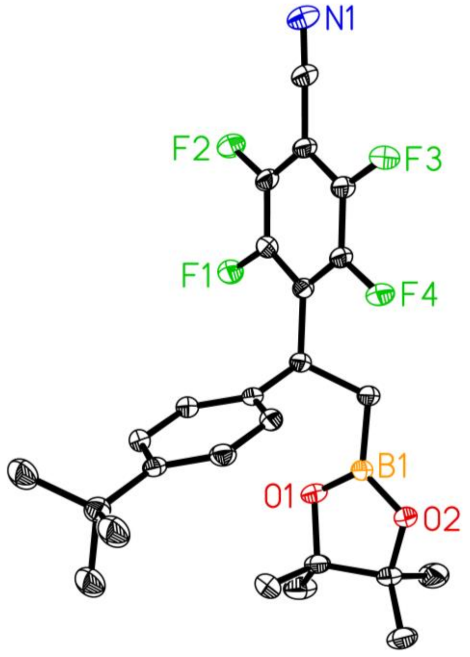

Displacement ellipsoid plot of **3c’** (30% probability level, without H and the second orientation of the disordered ^{t}Bu group)

7. References

[1] Y. M. Xi, J. F. Hartwig, *J. Am. Chem. Soc.* **2016**, *138*, 6703.

[2] S. Z. Sun, R. Martin, *Angew. Chem. Int. Ed.* **2018**, *57*, 3622.

8. NMR Spectra of the Products

NMR Spectra of **3a**

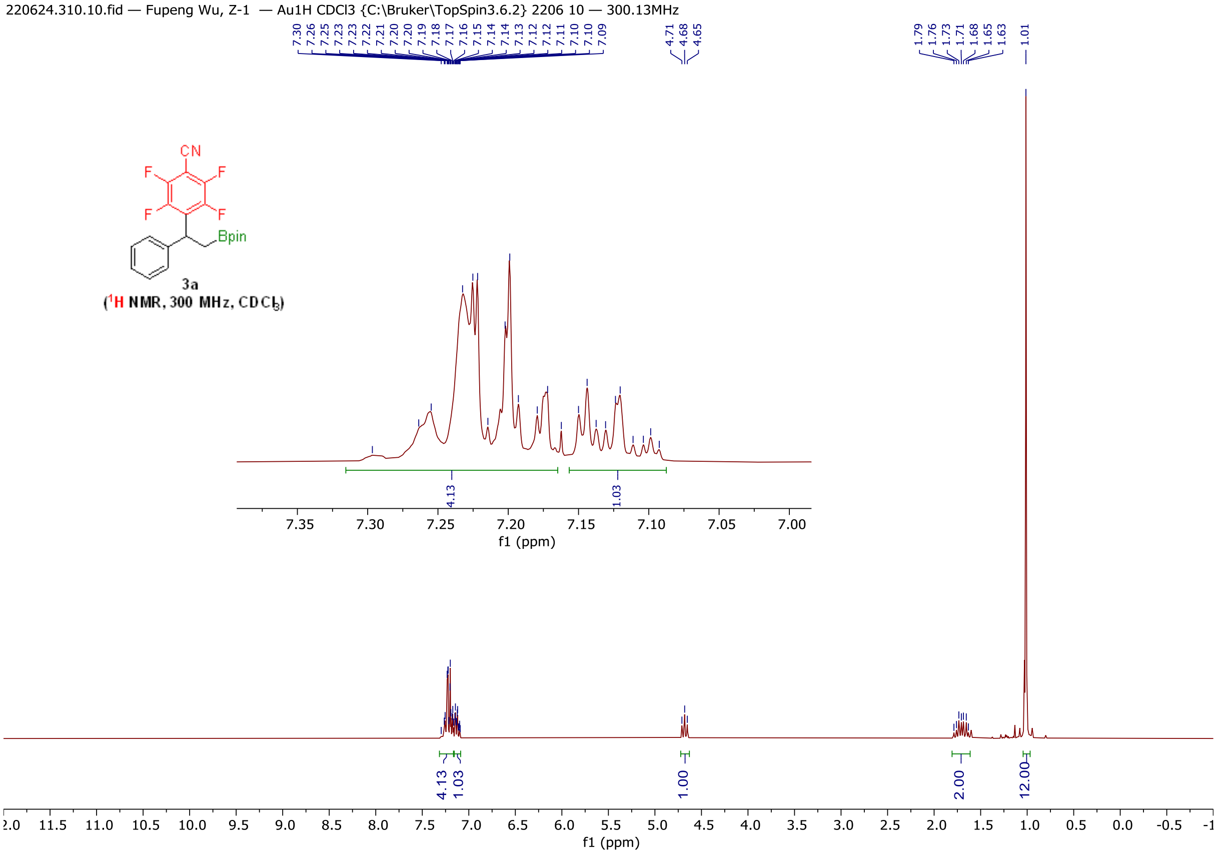

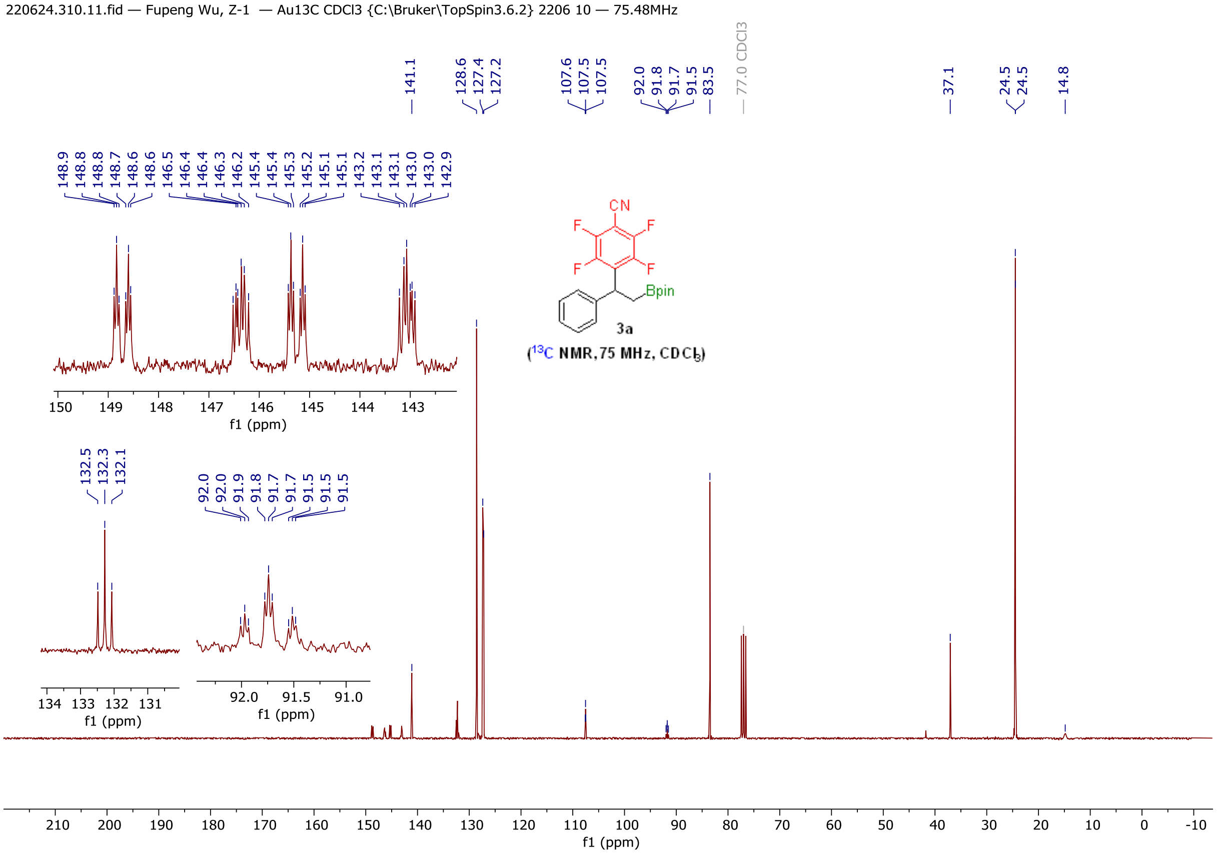

220624.310.12.fid — Fupeng Wu, Z-1 — Au19F CDCl3 {C:\Bruker\TopSpin3.6.2} 2206 10 — 282.39MHz

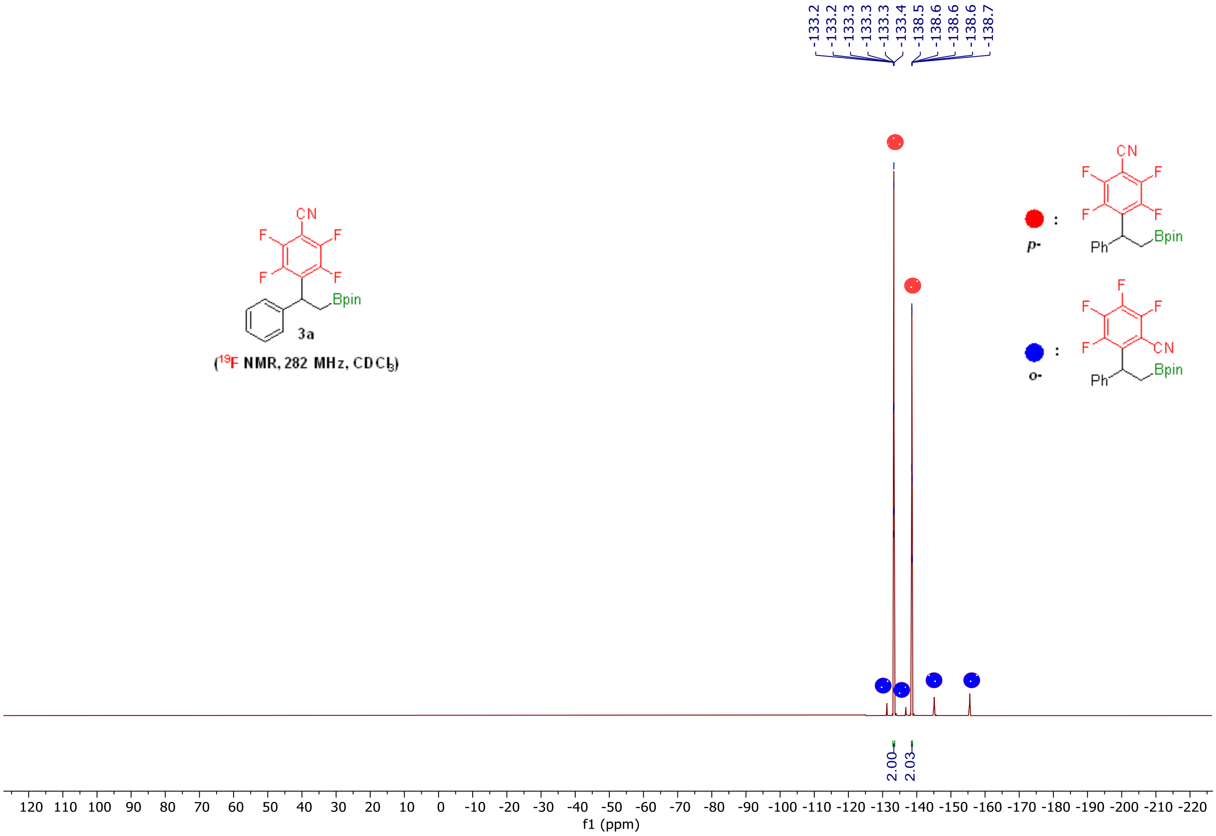

220624.310.13.fid — Fupeng Wu, Z-1 — Au11B CDCl3 {C:\Bruker\TopSpin3.6.2} 2206 10 — 96.29MHz

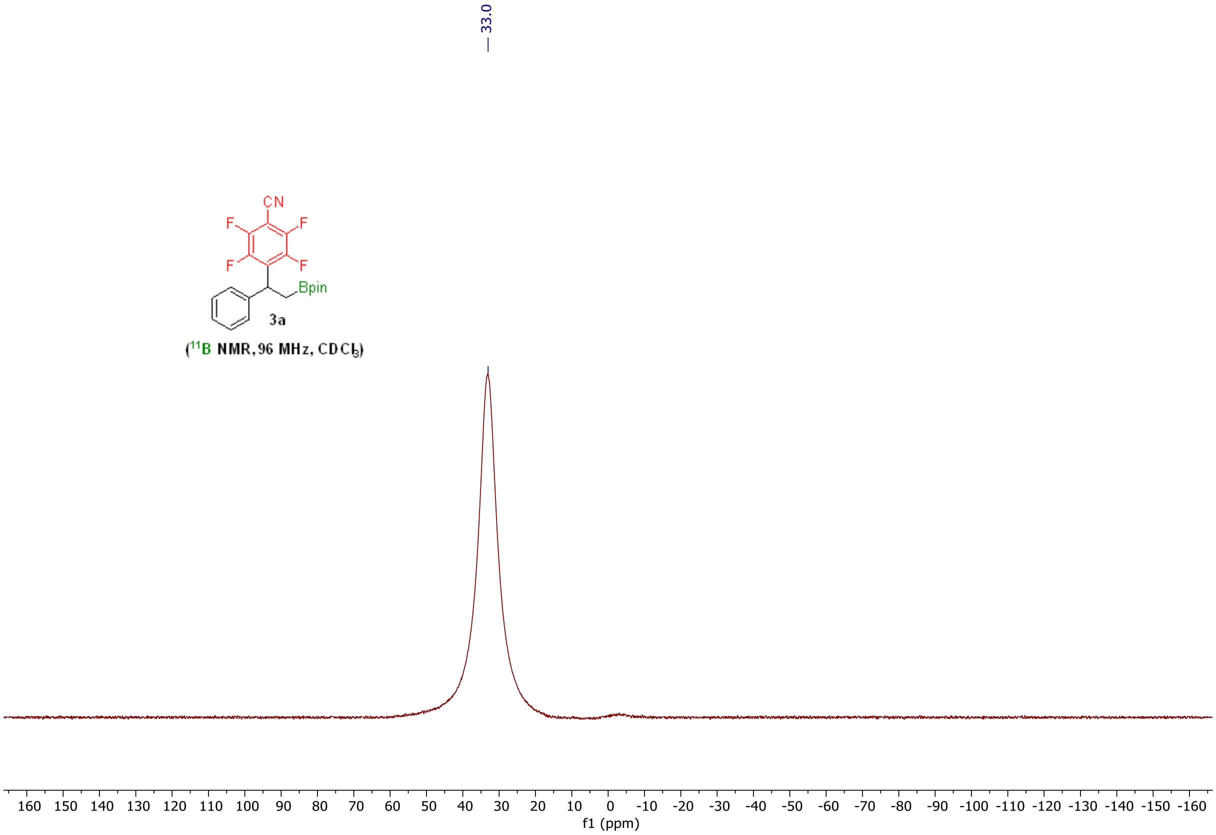

NMR Spectra of **3b**

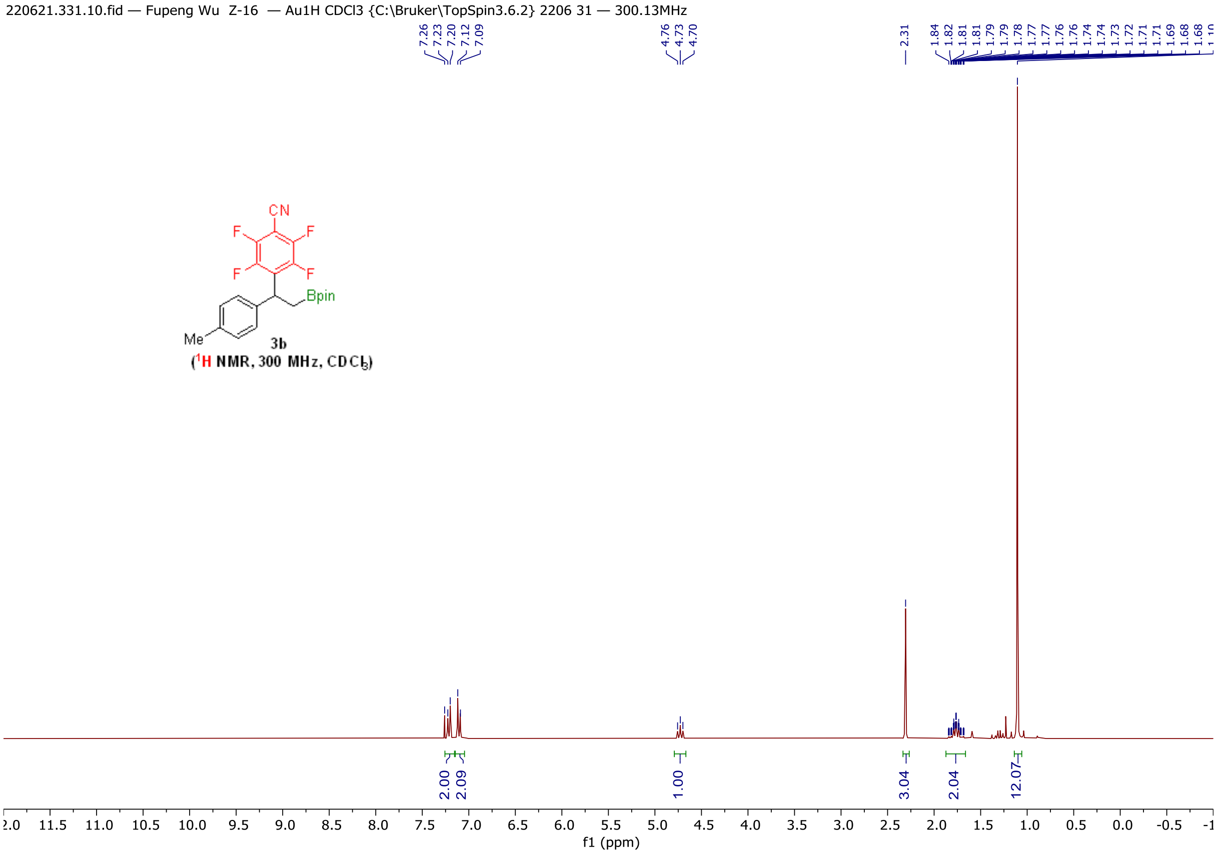

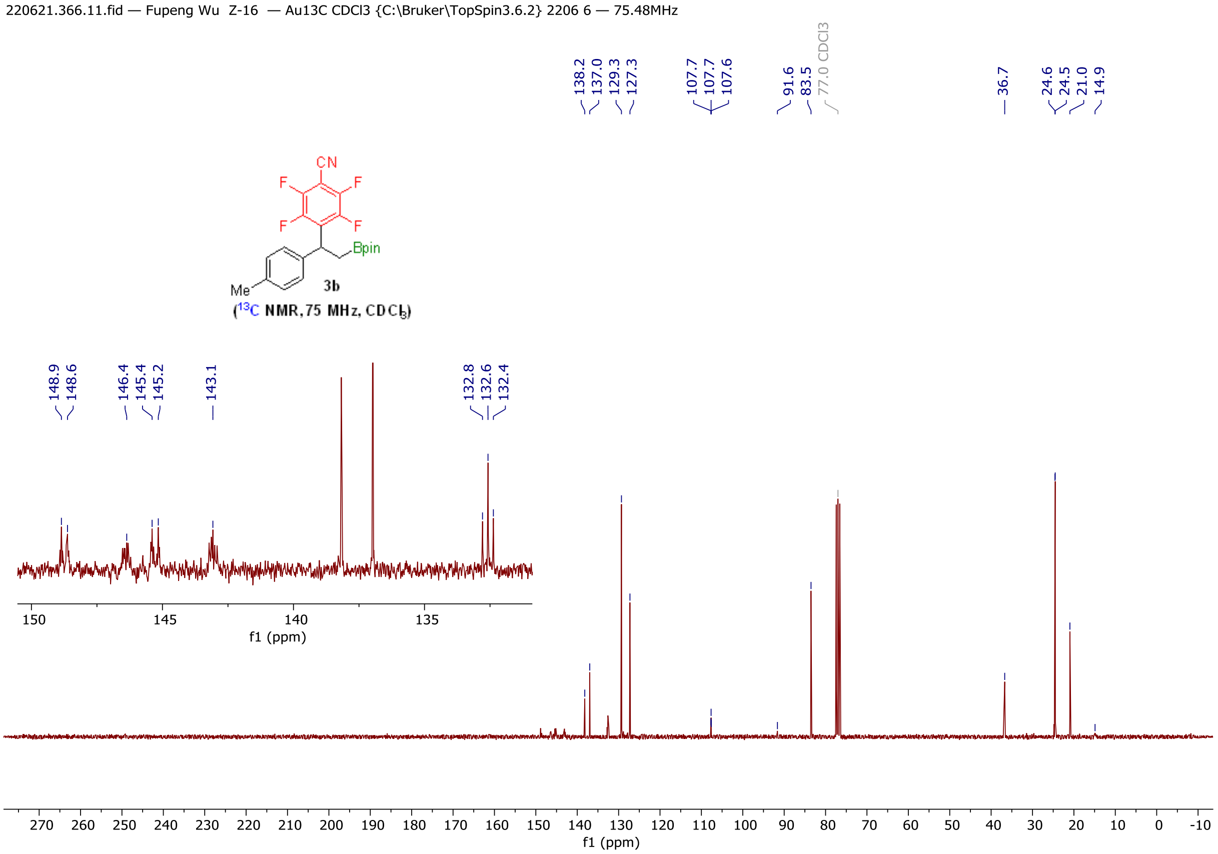

220621.366.12.fid — Fupeng Wu Z-16 — Au19F CDCl3 {C:\Bruker\TopSpin3.6.2} 2206 6 — 282.39MHz

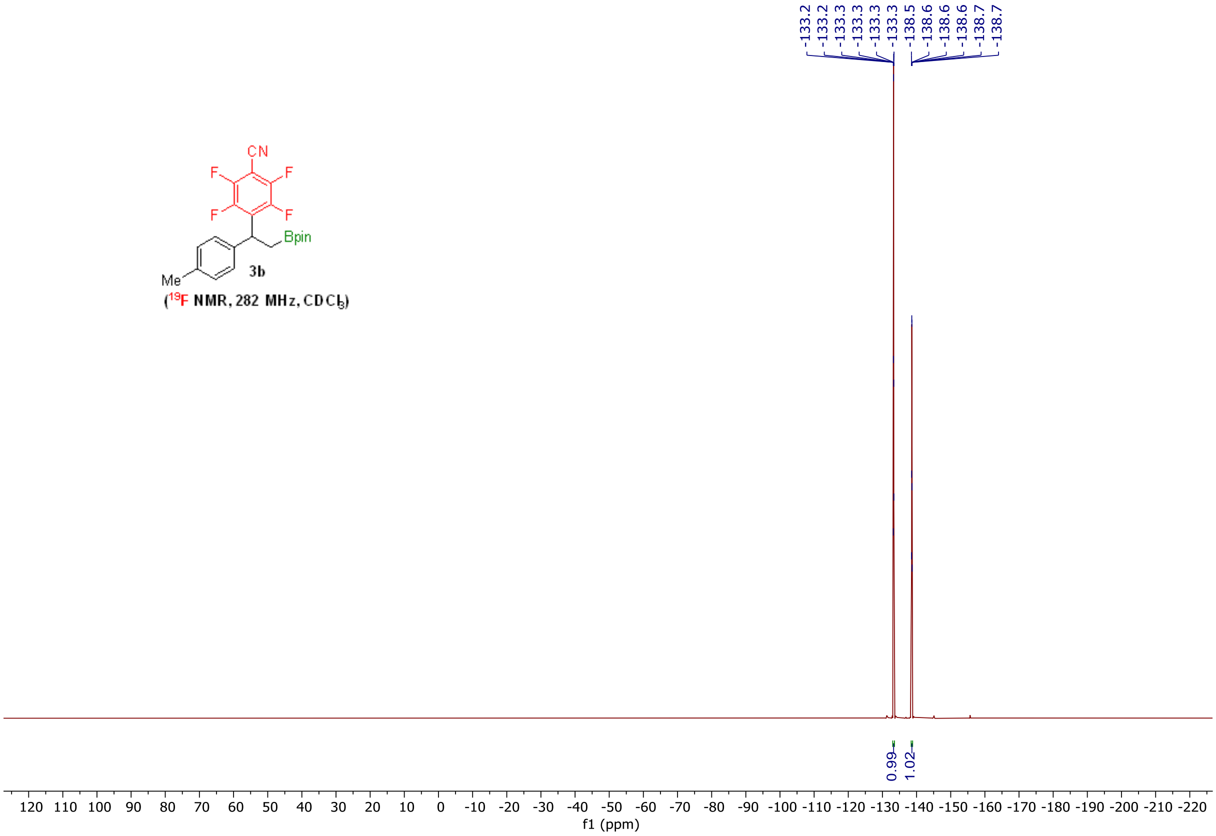

220621.366.10.fid — Fupeng Wu Z-16 — Au11B CDCl3 {C:\Bruker\TopSpin3.6.2} 2206 6 — 96.29MHz

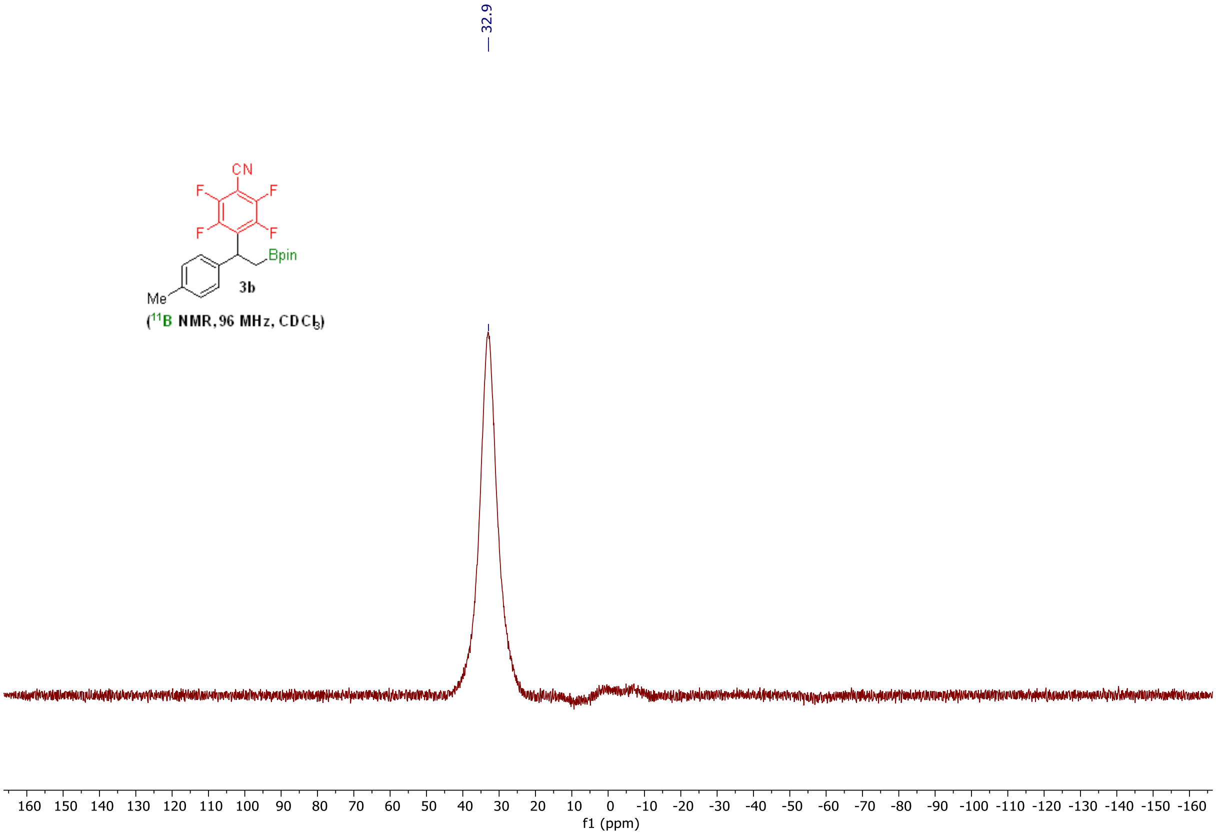

NMR Spectra of **3c**

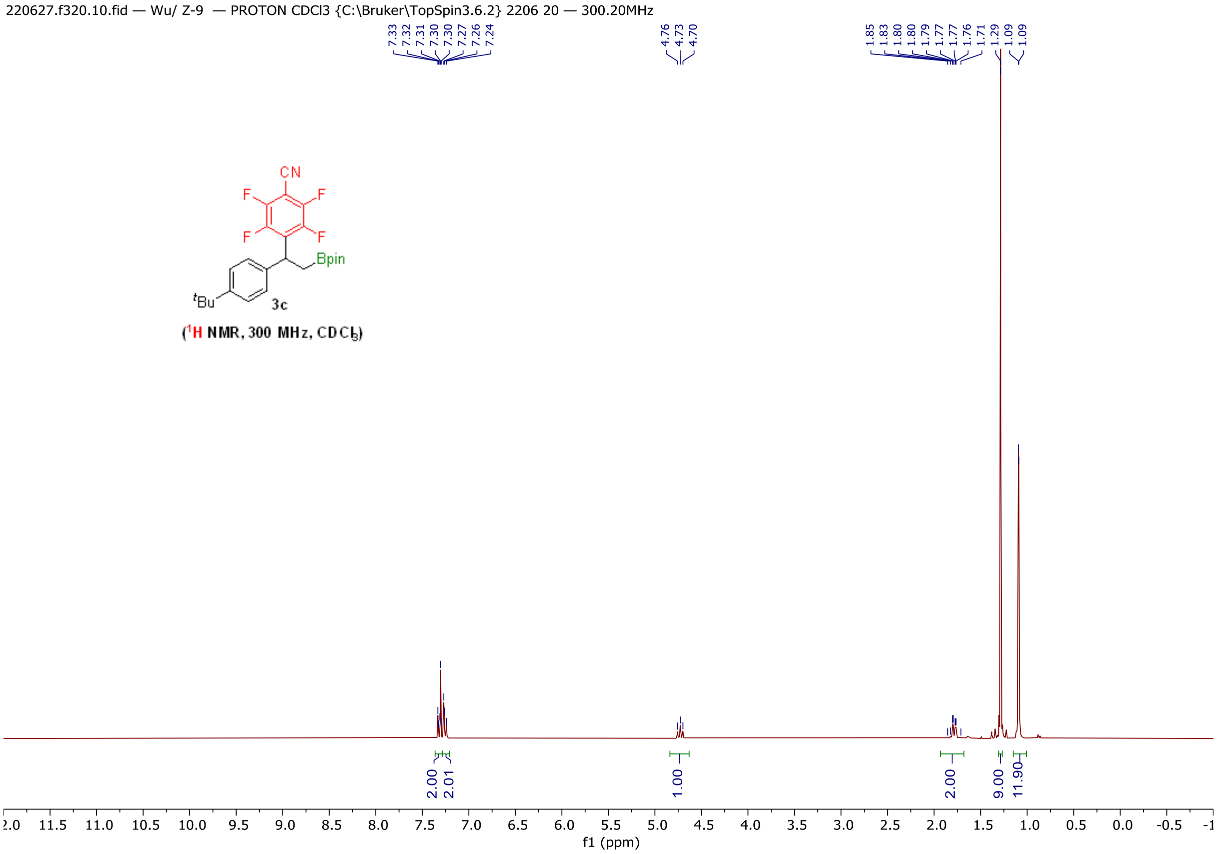

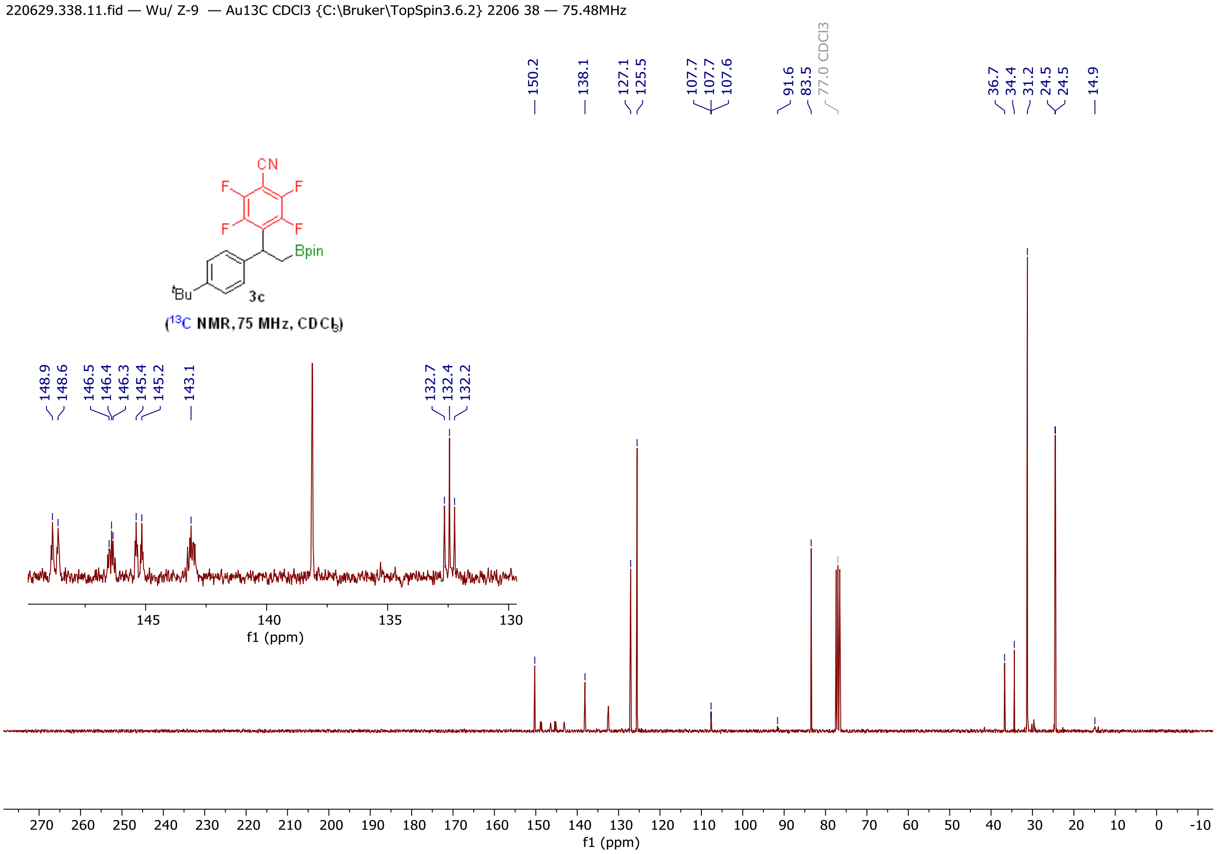

220629.338.12.fid — Wu/ Z-9 — Au19F CDCl3 {C:\Bruker\TopSpin3.6.2} 2206 38 — 282.39MHz

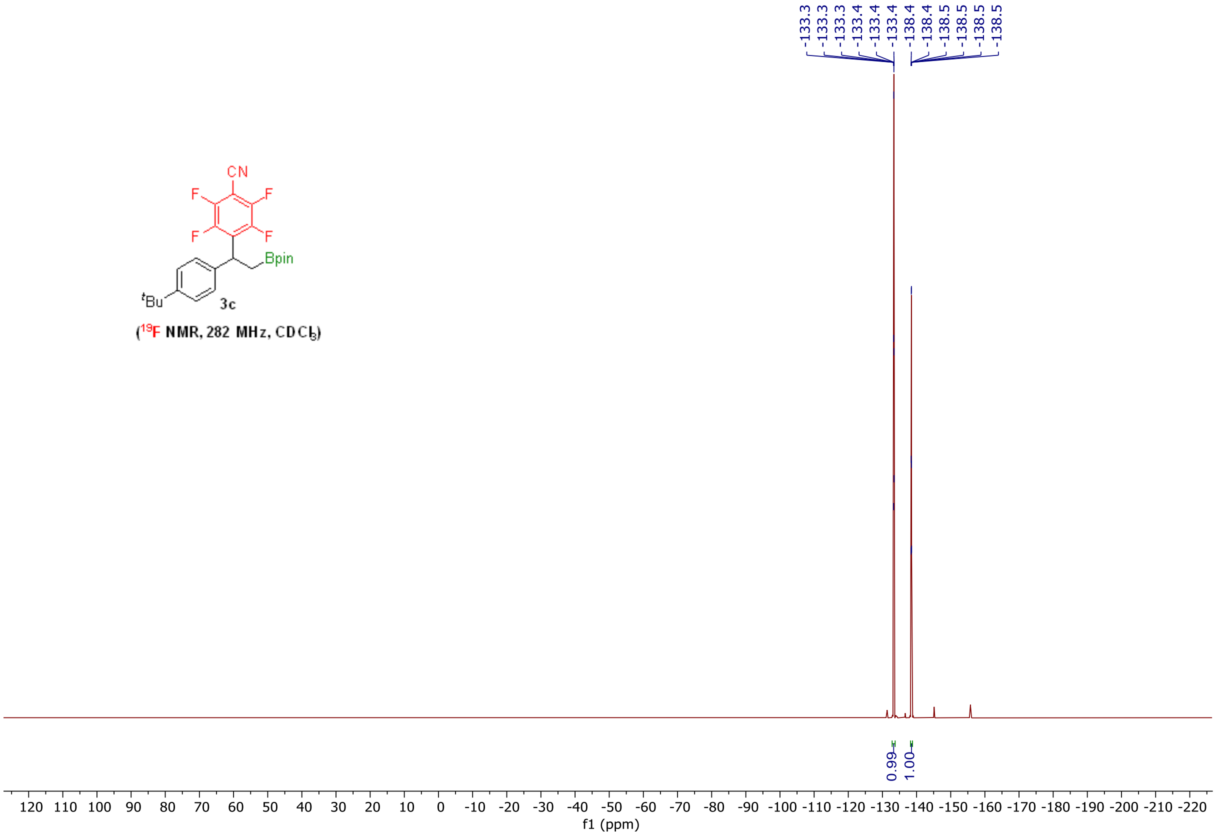

220629.338.13.fid — Wu/ Z-9 — Au11B CDCl3 {C:\Bruker\TopSpin3.6.2} 2206 38 — 96.29MHz

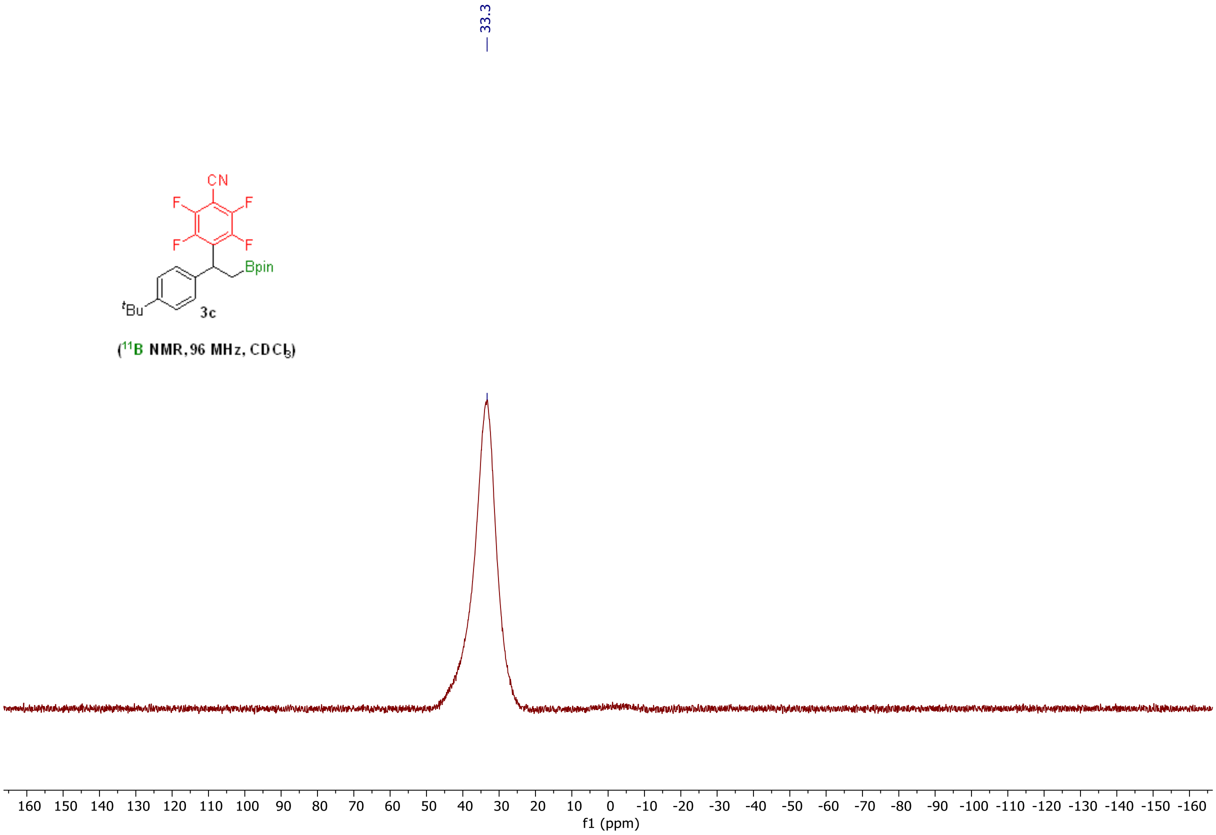

NMR Spectra of **3d**

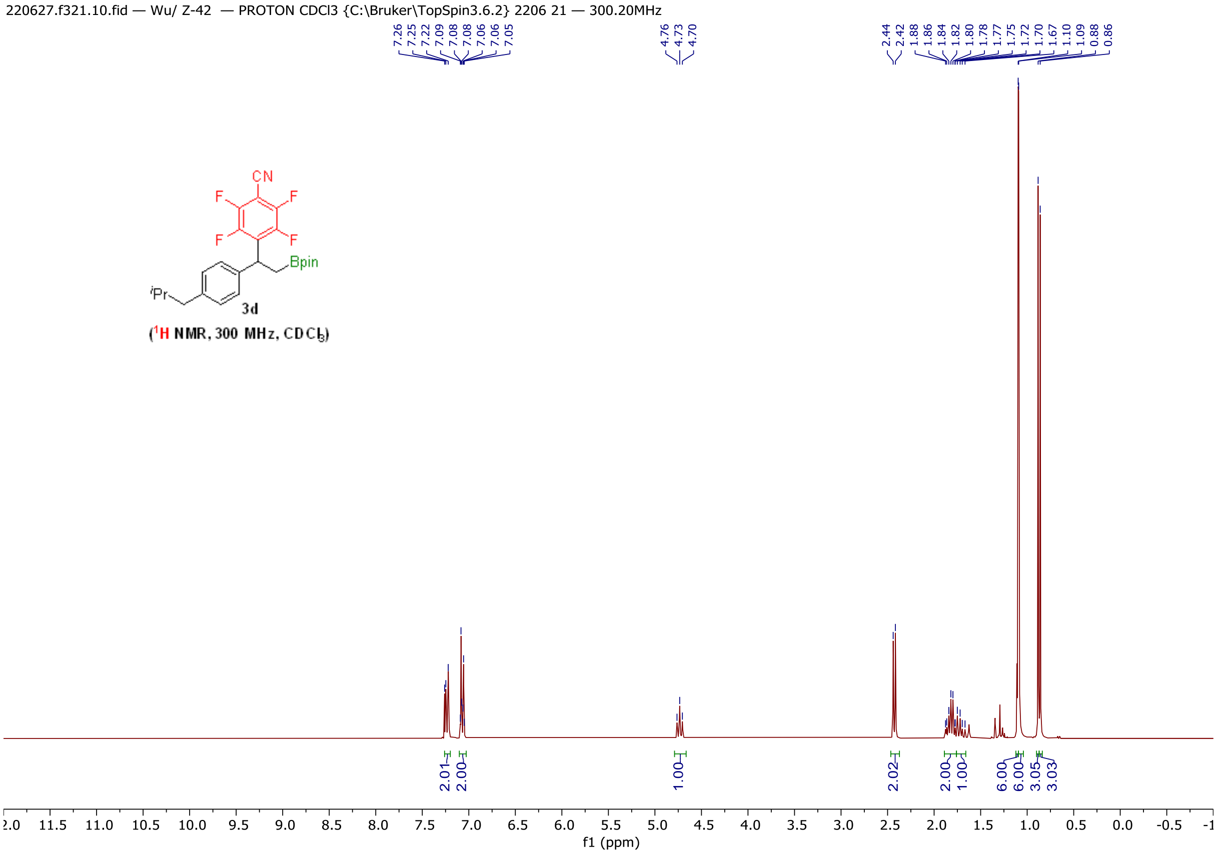

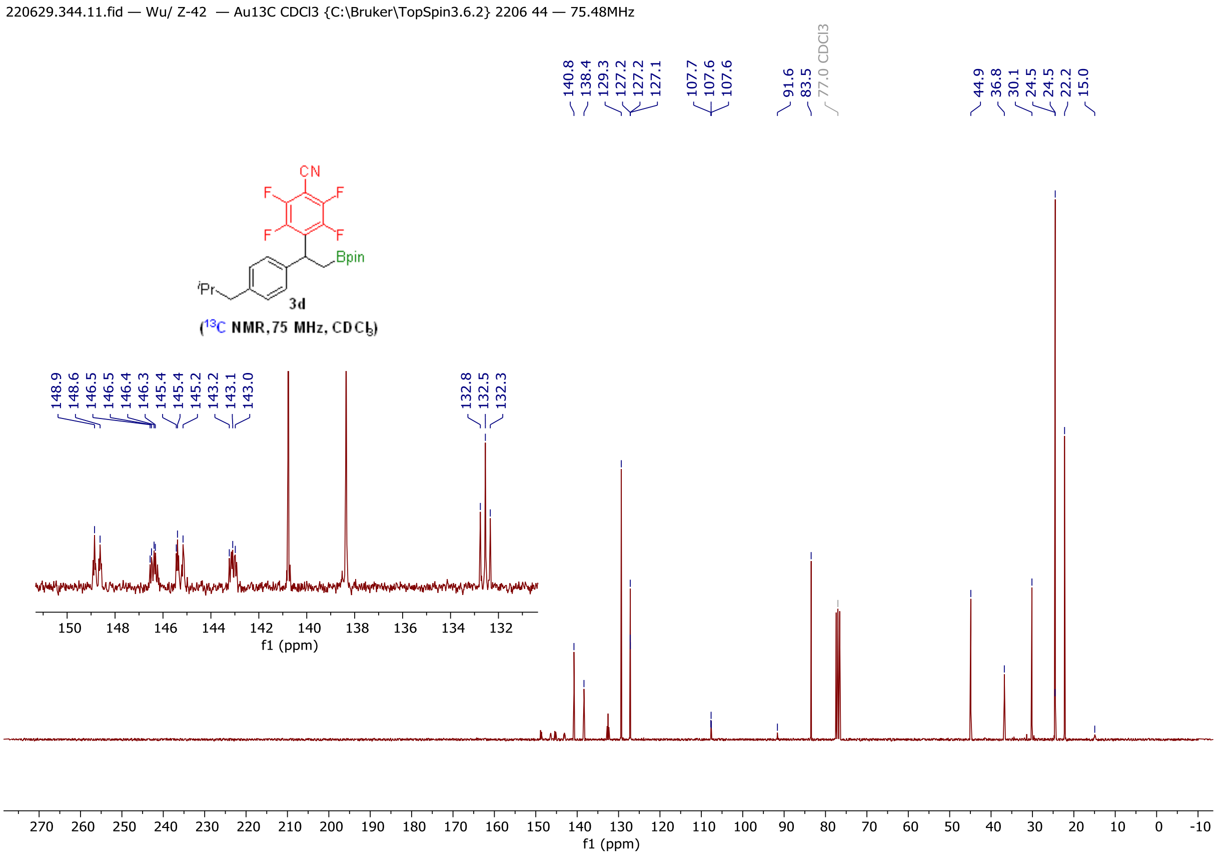

220629.344.12.fid — Wu/ Z-42 — Au19F CDCl3 {C:\Bruker\TopSpin3.6.2} 2206 44 — 282.39MHz

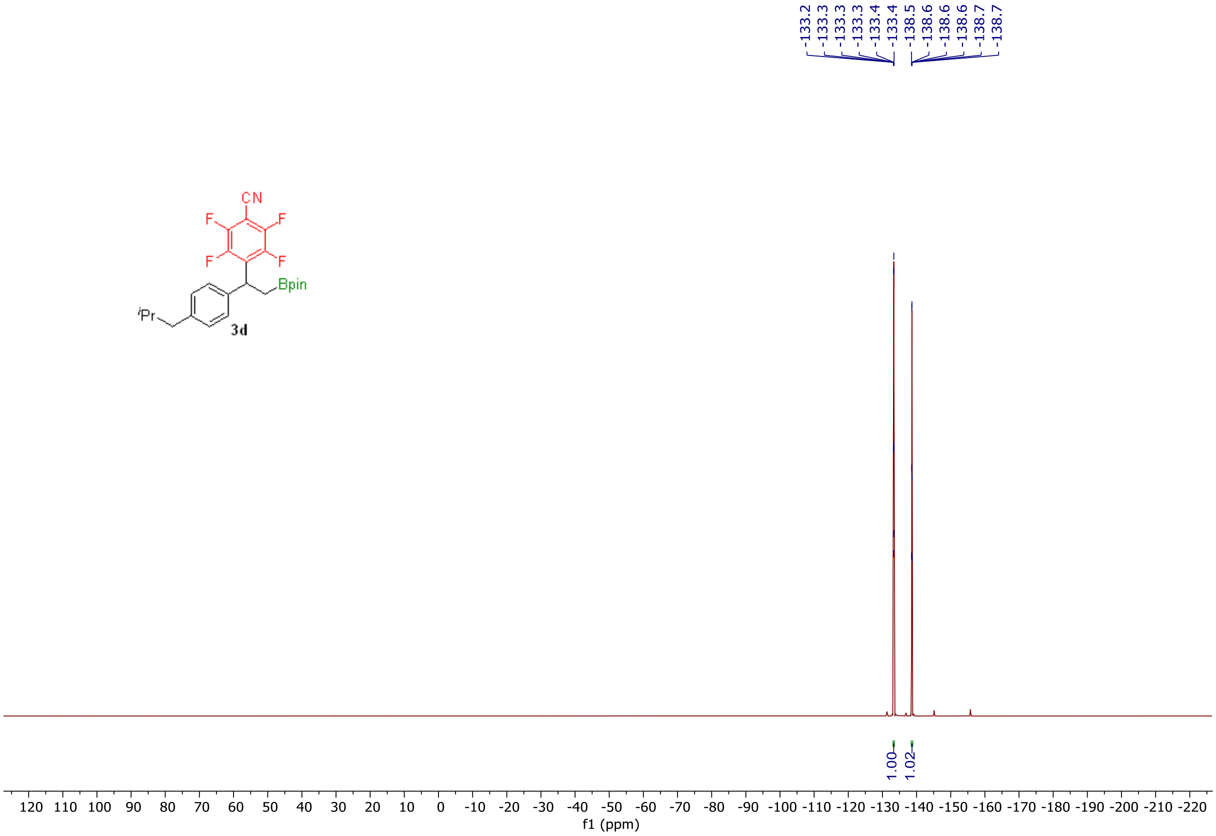

220629.344.13.fid — Wu/ Z-42 — Au11B CDCl3 {C:\Bruker\TopSpin3.6.2} 2206 44 — 96.29MHz

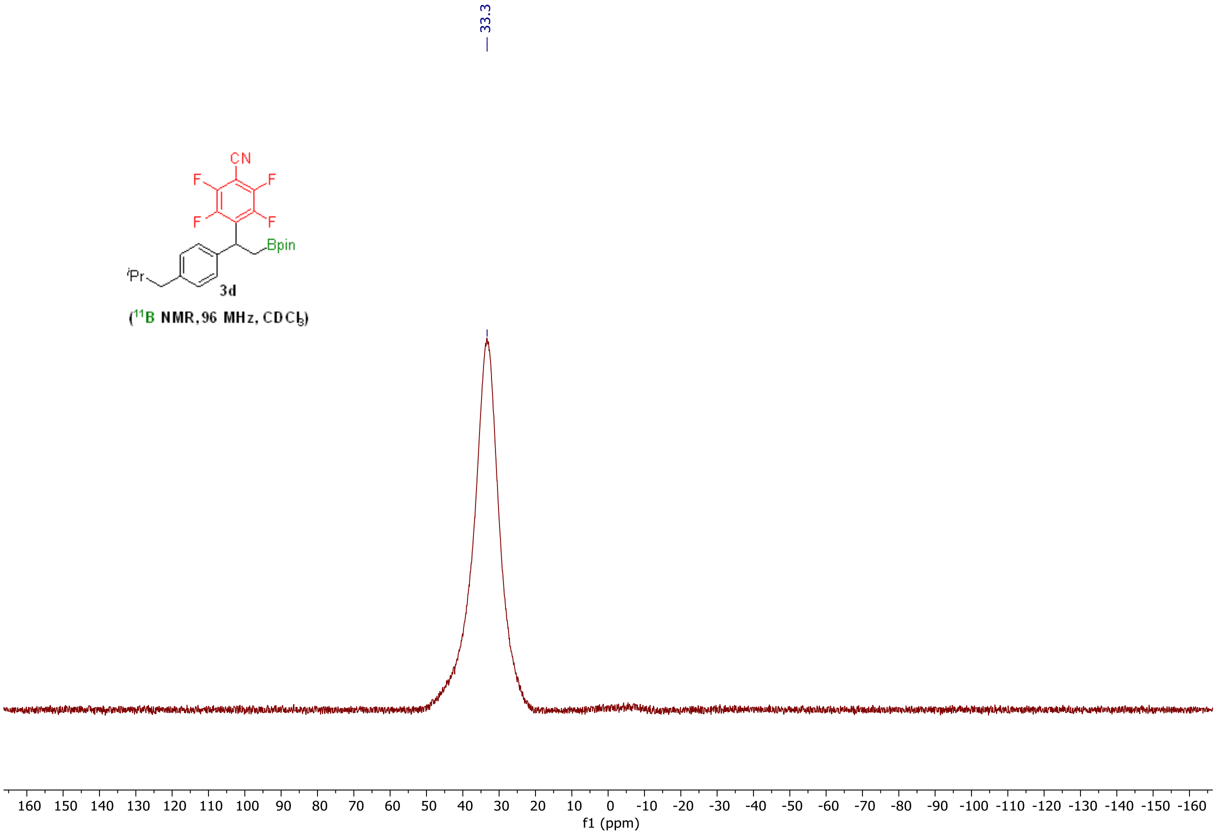

NMR Spectra of **3e**

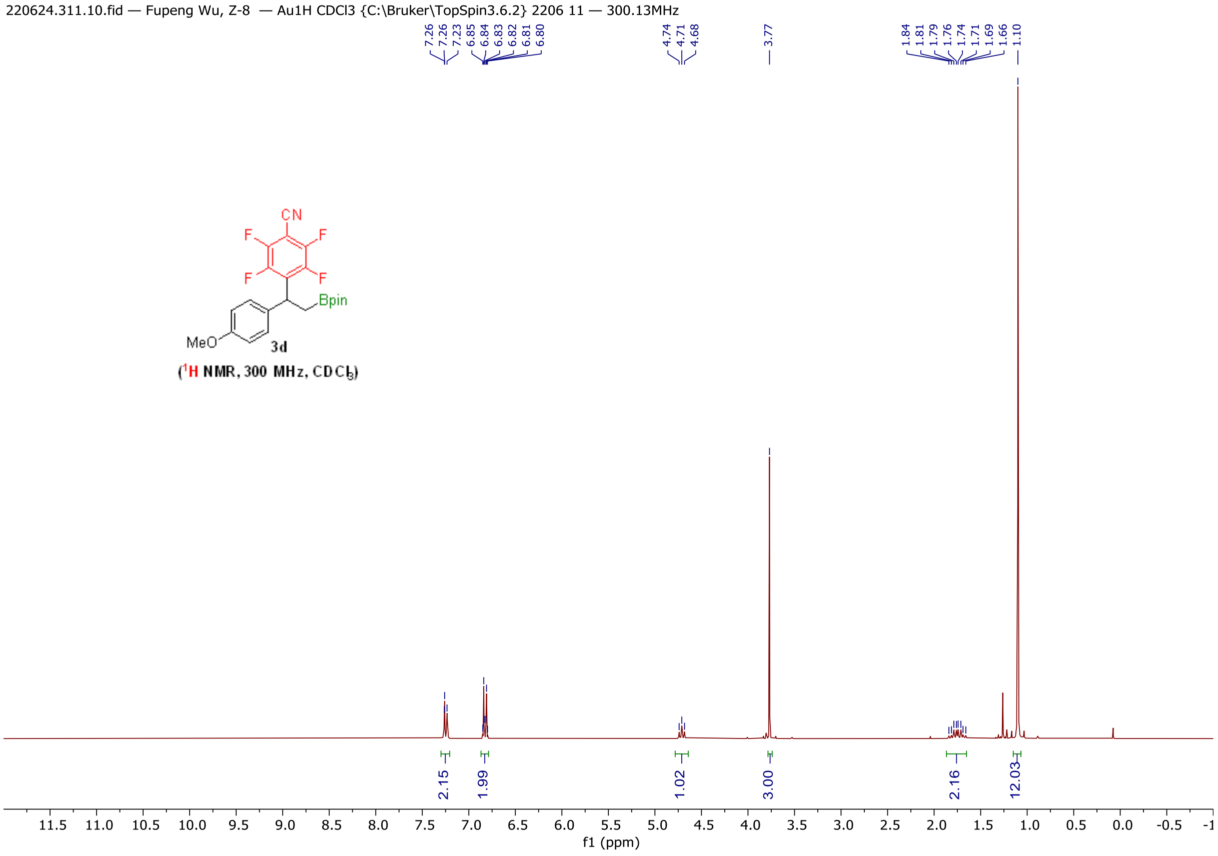

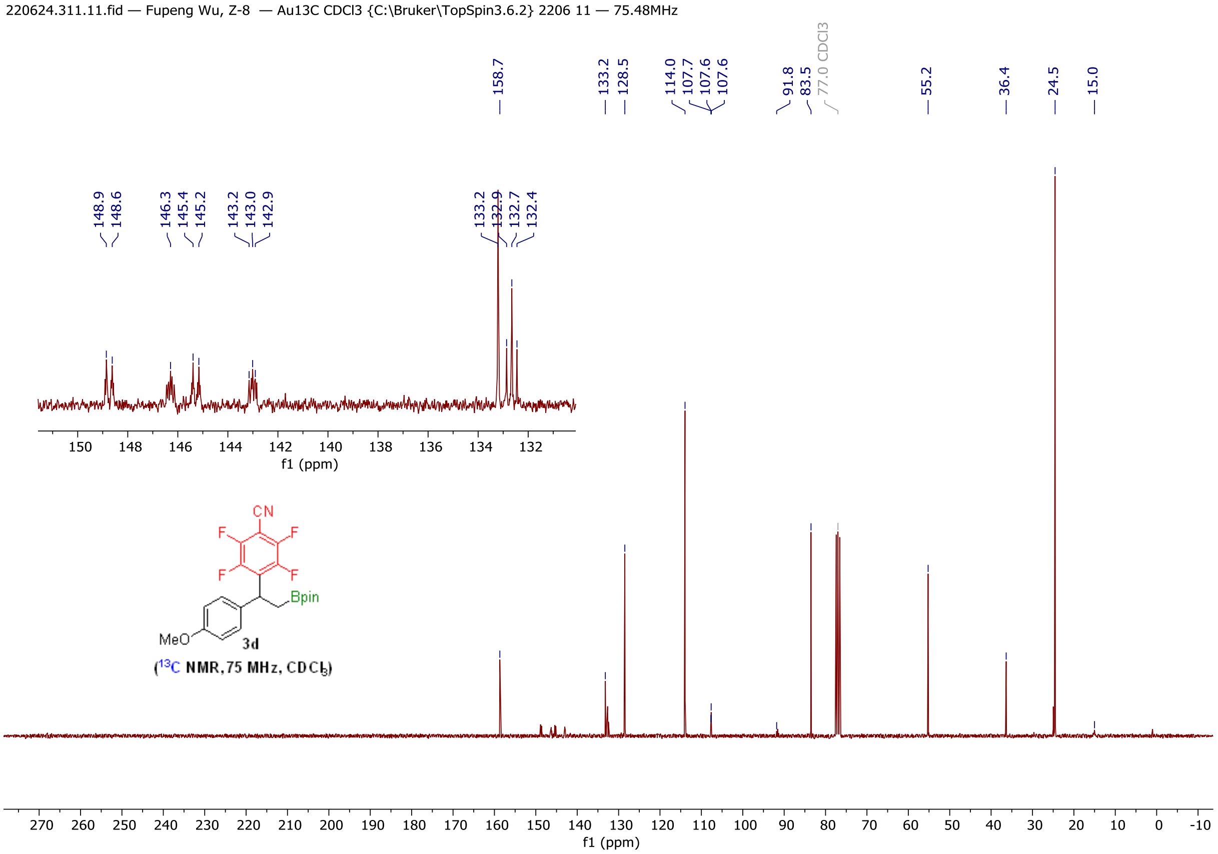

220624.311.12.fid — Fupeng Wu, Z-8 — Au19F CDCl3 {C:\Bruker\TopSpin3.6.2} 2206 11 — 282.39MHz

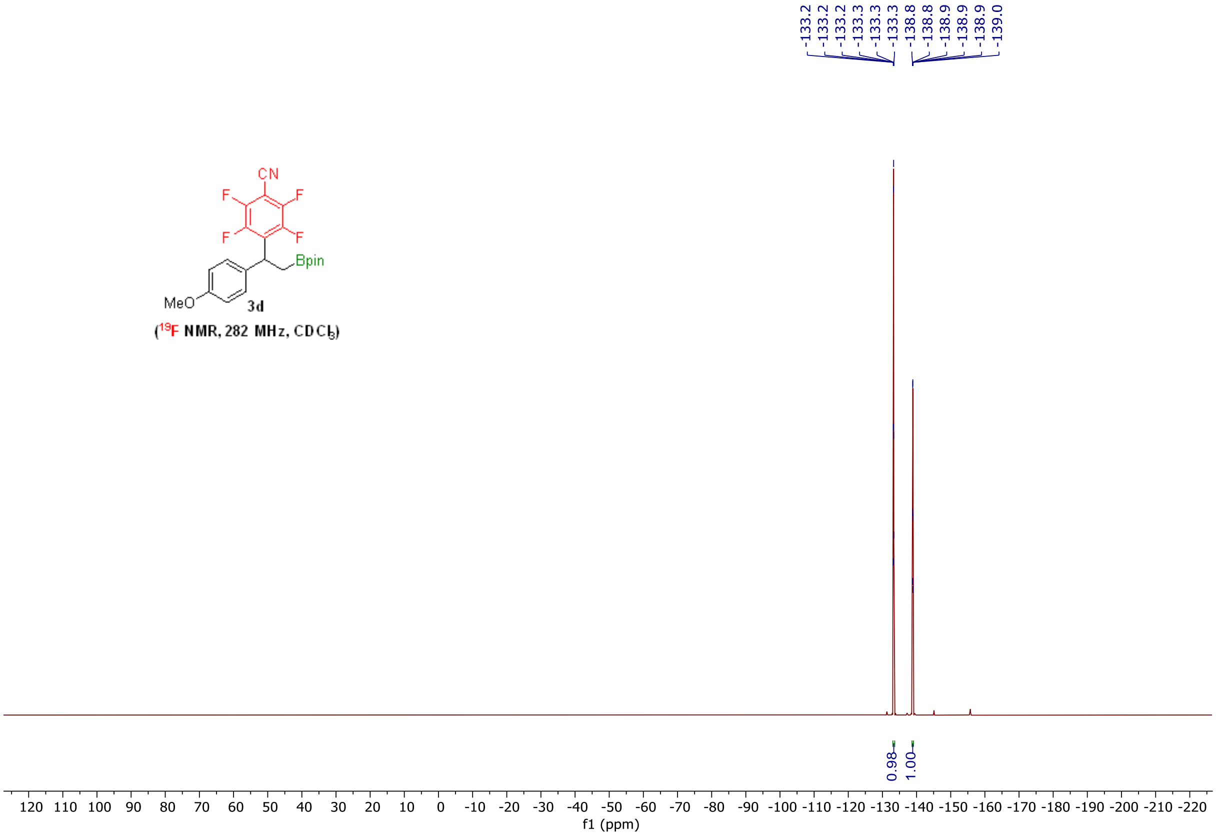

220624.311.13.fid — Fupeng Wu, Z-8 — Au11B CDCl3 {C:\Bruker\TopSpin3.6.2} 2206 11 — 96.29MHz

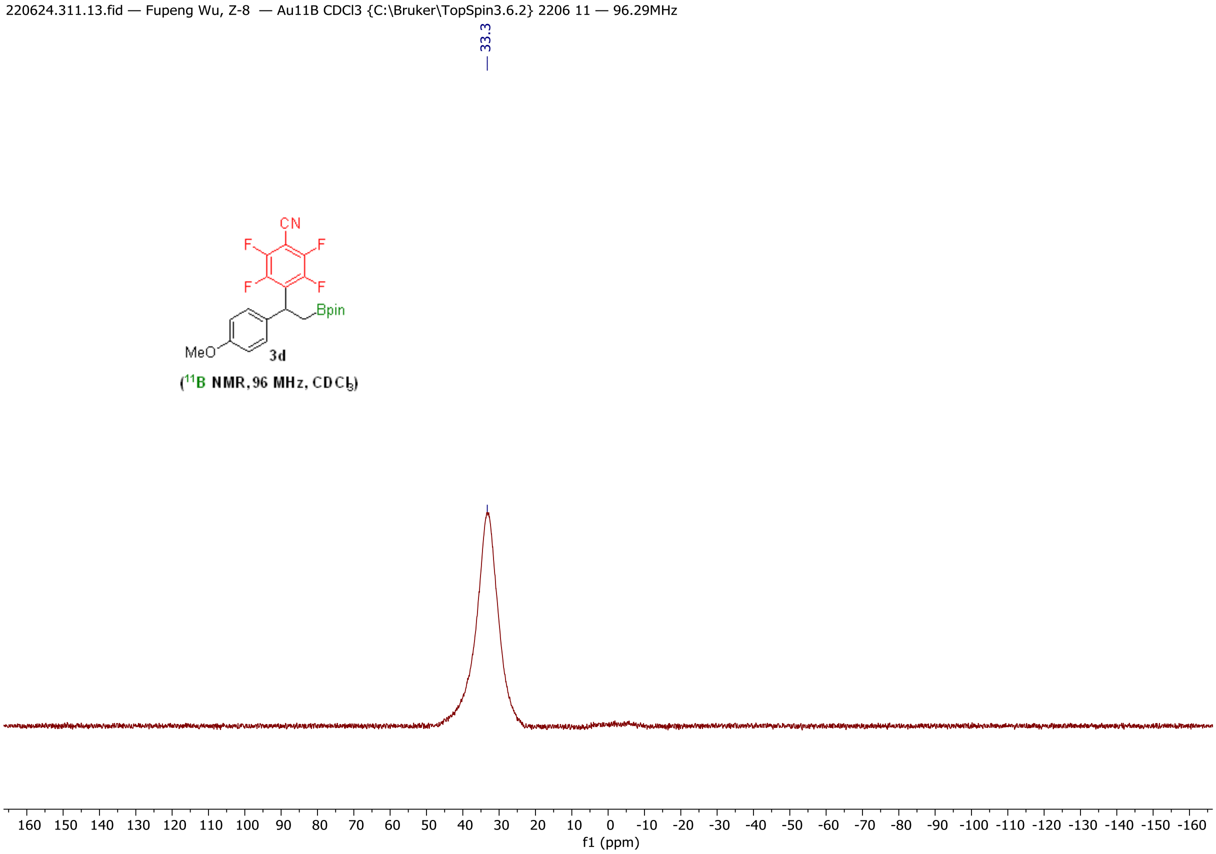

NMR Spectra of **3f**

220621.329.10.fid — Fupeng Wu Z-13 — Au1H CDCl3 {C:\Bruker\TopSpin3.6.2} 2206 29 — 300.13MHz

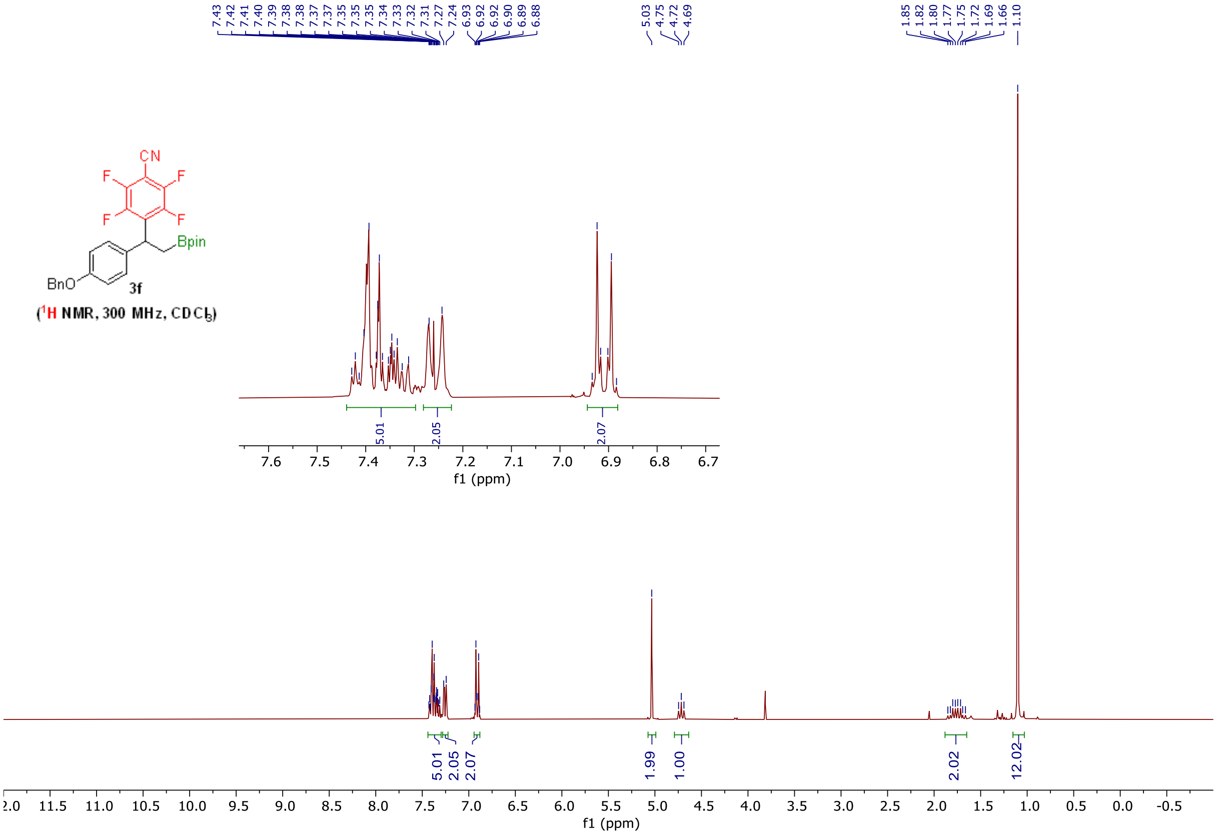

220621.368.11.fid — Fupeng Wu Z-13 — Au13C CDCl3 {C:\Bruker\TopSpin3.6.2} 2206 8 — 75.48MHz

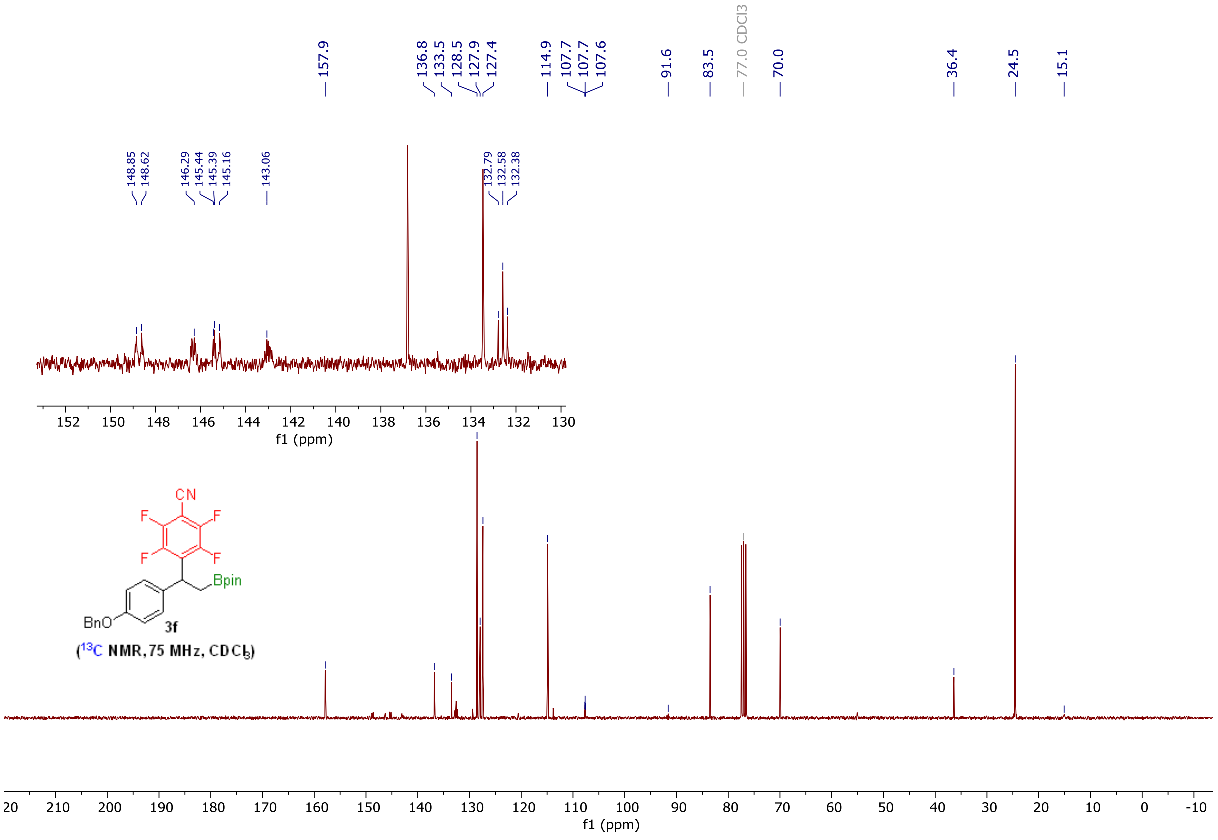

220621.368.12.fid — Fupeng Wu Z-13 — Au19F CDCl3 {C:\Bruker\TopSpin3.6.2} 2206 8 — 282.39MHz

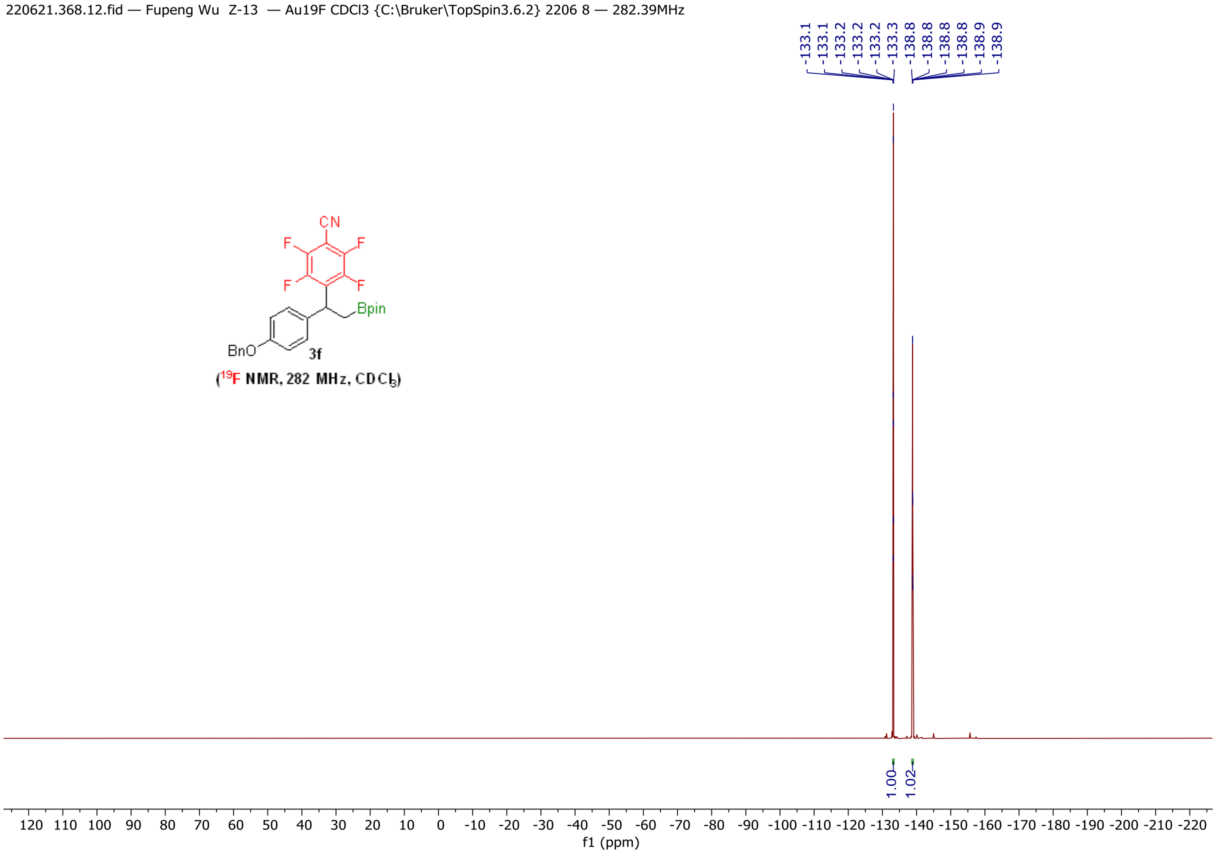

220621.368.10.fid — Fupeng Wu Z-13 — Au11B CDCl3 {C:\Bruker\TopSpin3.6.2} 2206 8 — 96.29MHz

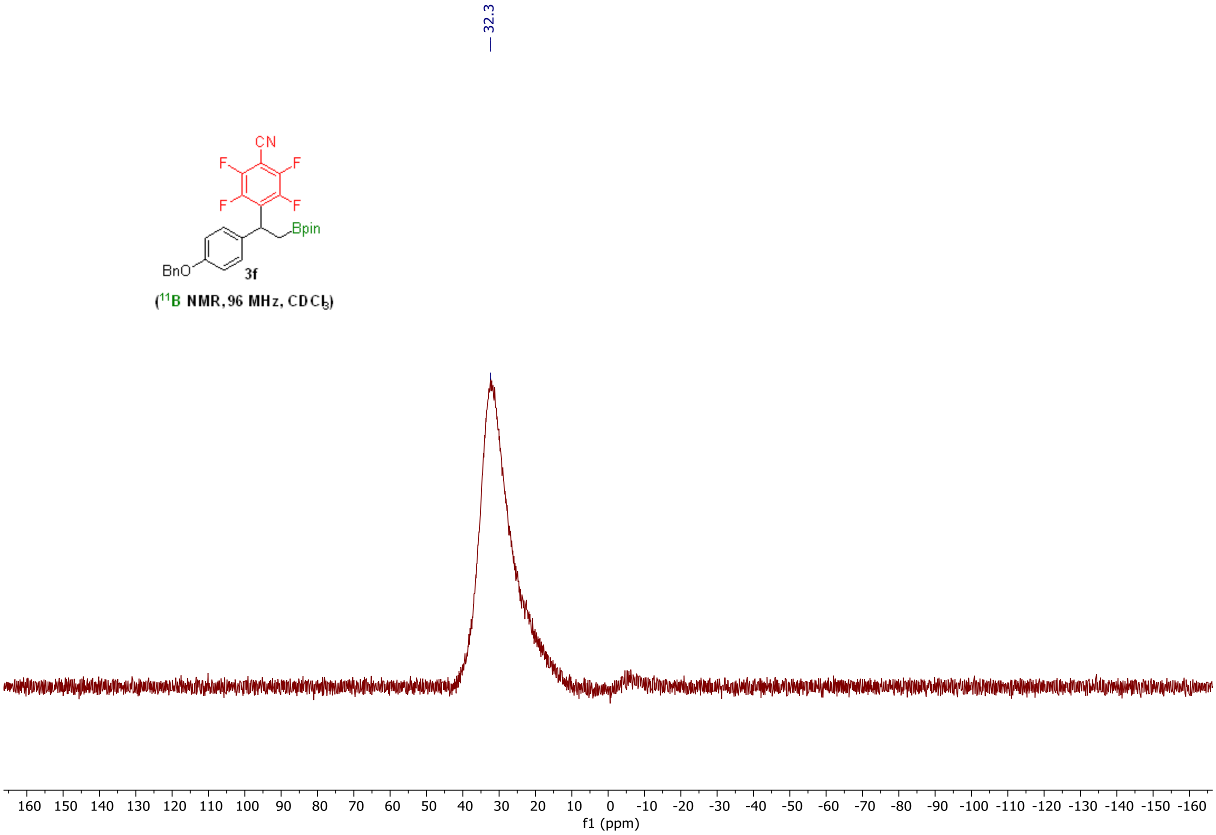

NMR Spectra of **3g**

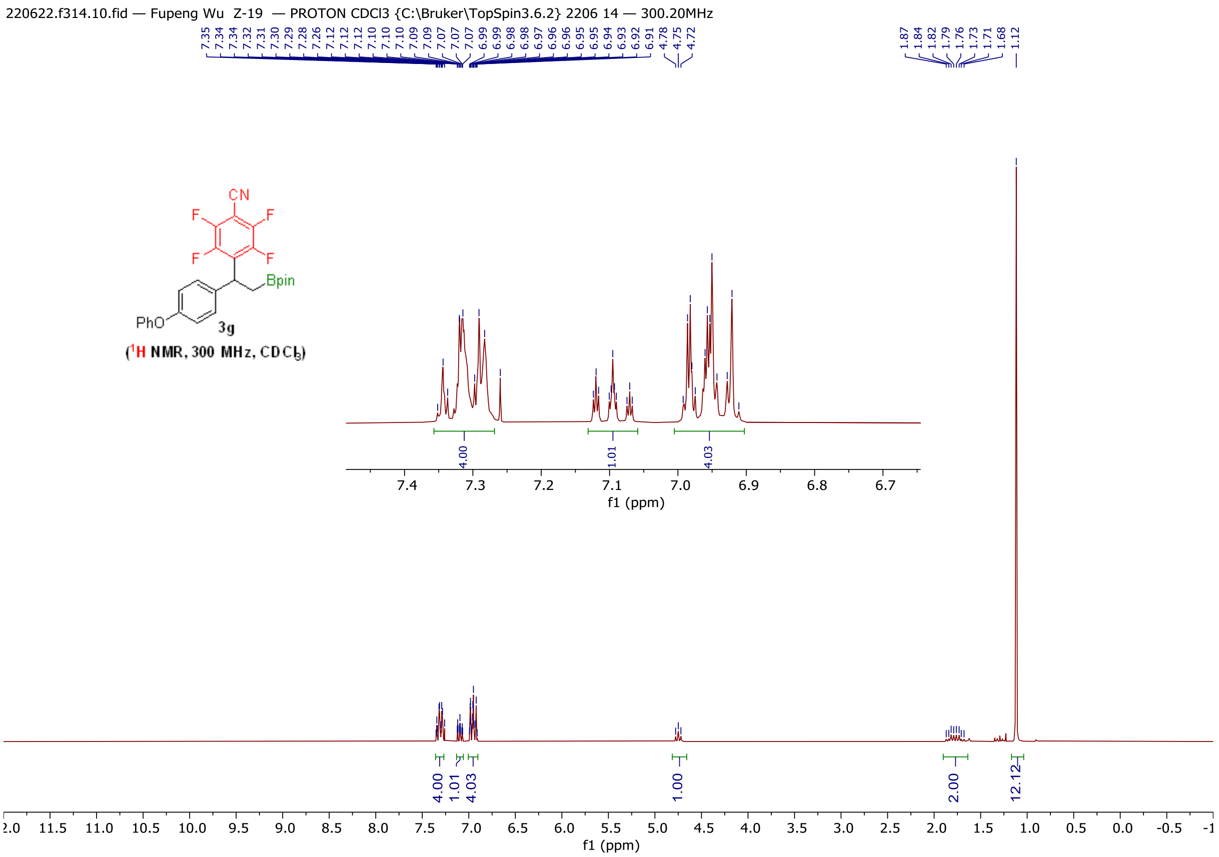

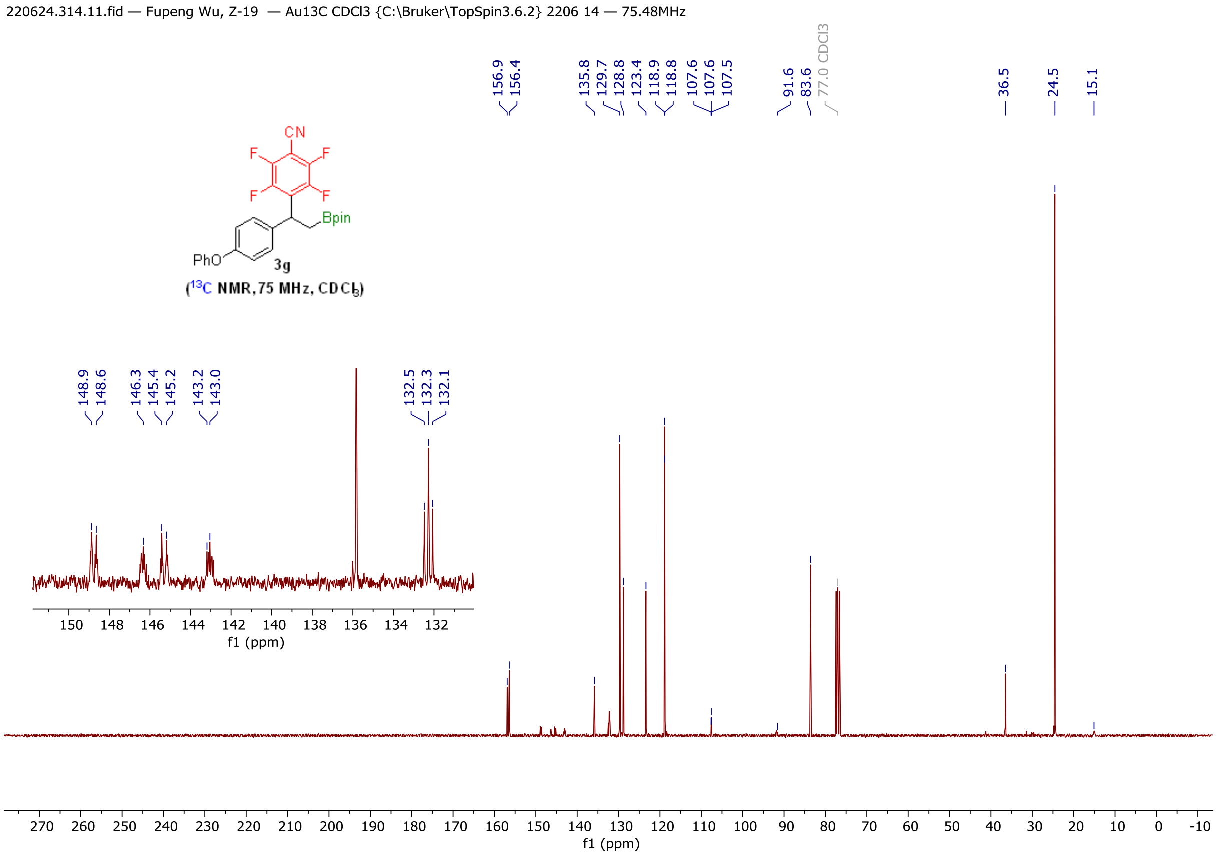

220624.314.12.fid — Fupeng Wu, Z-19 — Au19F CDCl3 {C:\Bruker\TopSpin3.6.2} 2206 14 — 282.39MHz

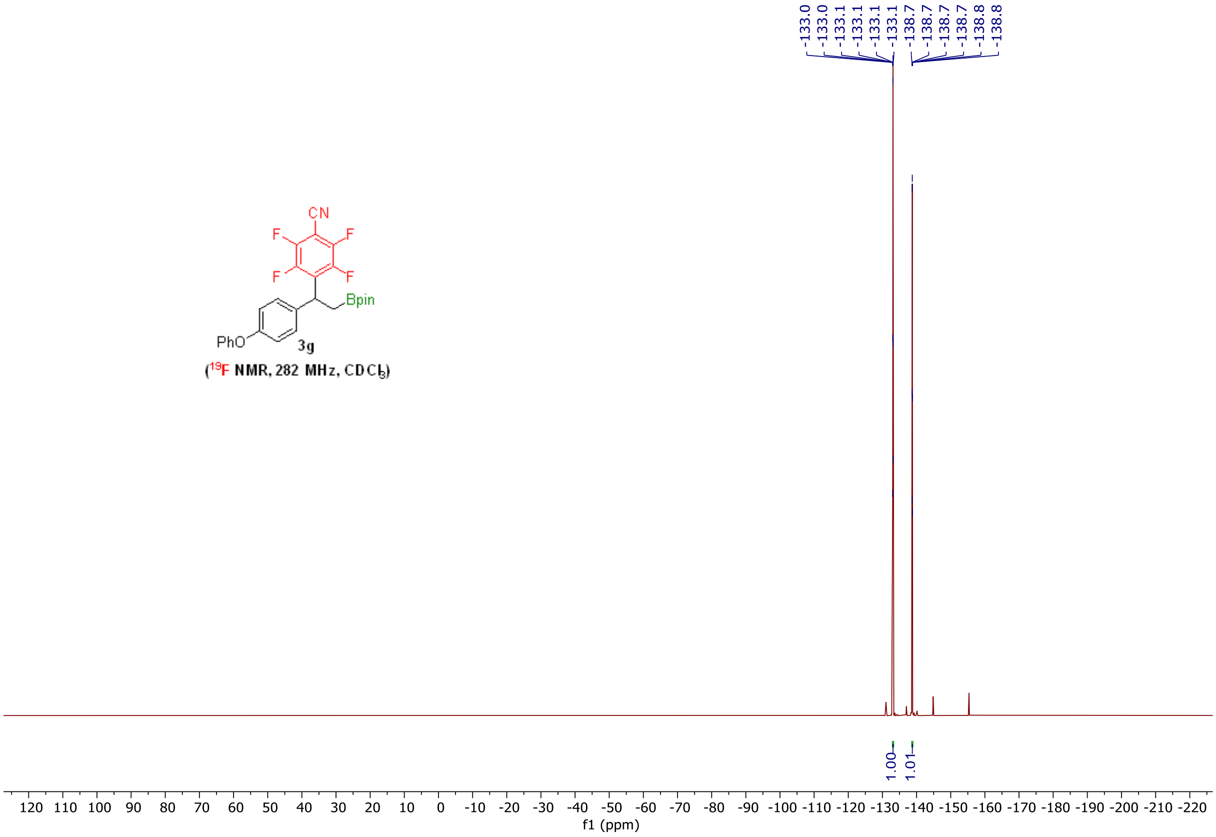

220624.314.13.fid — Fupeng Wu, Z-19 — Au11B CDCl3 {C:\Bruker\TopSpin3.6.2} 2206 14 — 96.29MHz

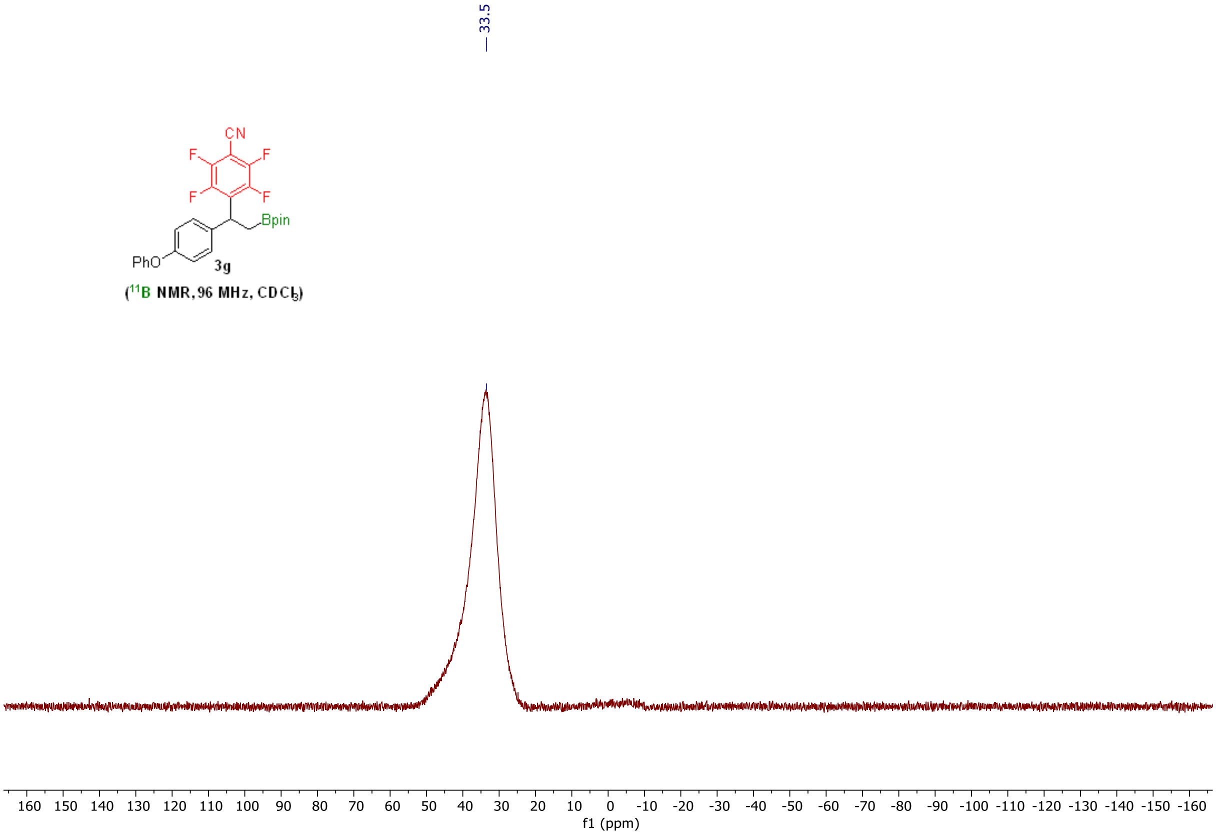

NMR Spectra of **3h**

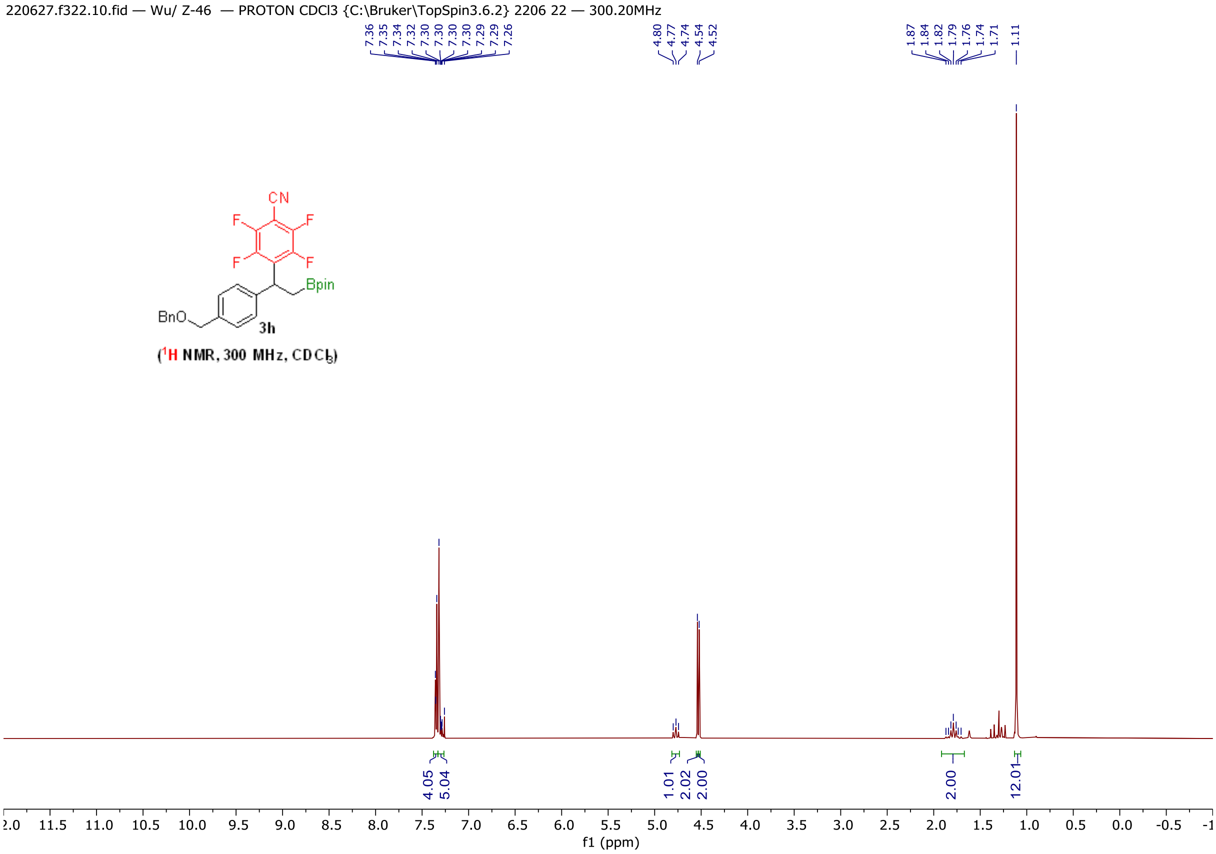

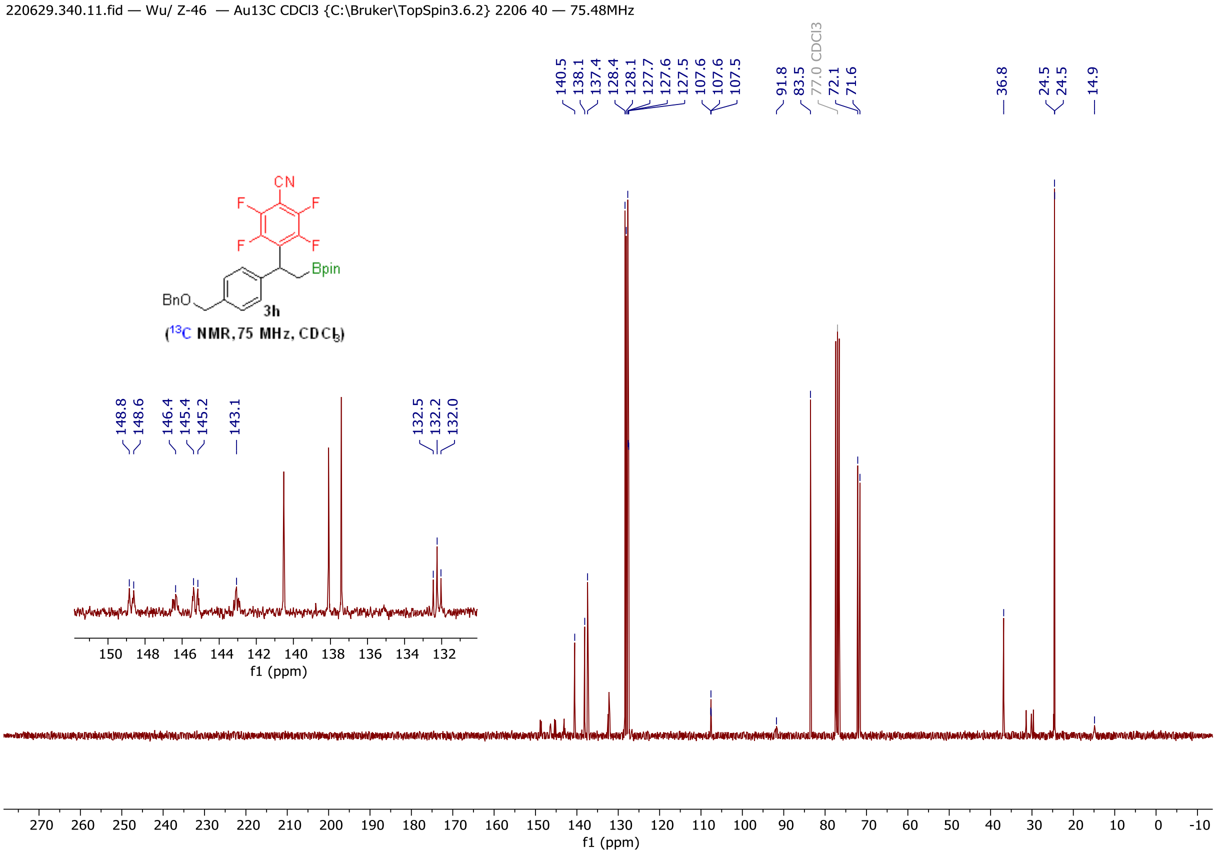

220629.340.12.fid — Wu/ Z-46 — Au19F CDCl3 {C:\Bruker\TopSpin3.6.2} 2206 40 — 282.39MHz

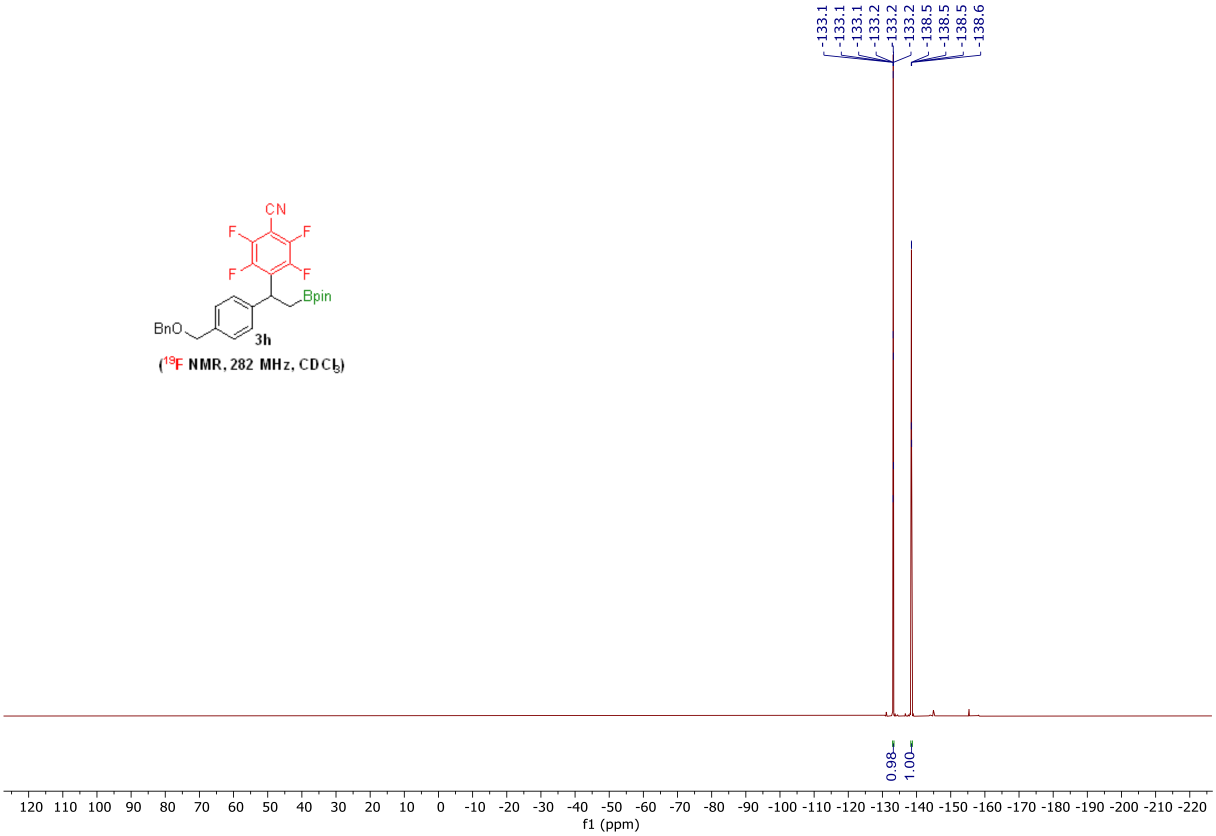

220629.340.13.fid — Wu/ Z-46 — Au11B CDCl3 {C:\Bruker\TopSpin3.6.2} 2206 40 — 96.29MHz

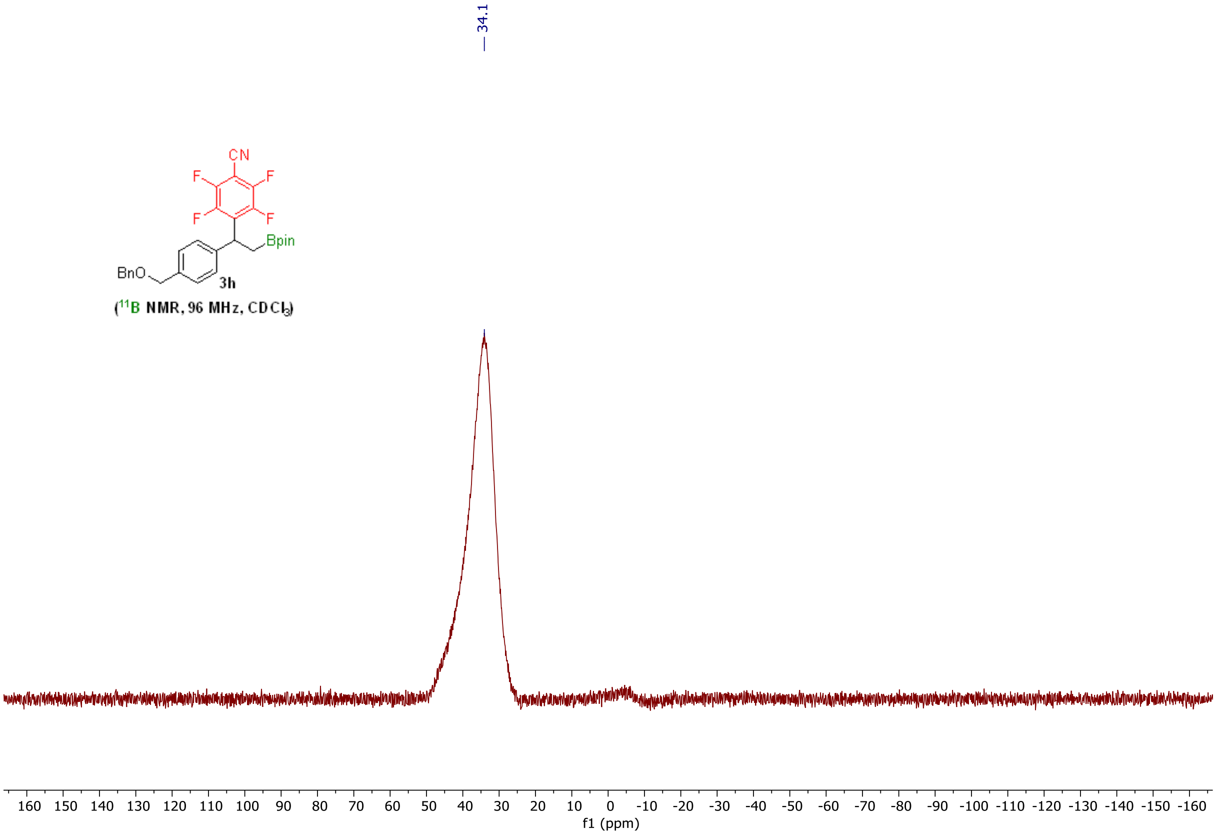

NMR Spectra of **3i**

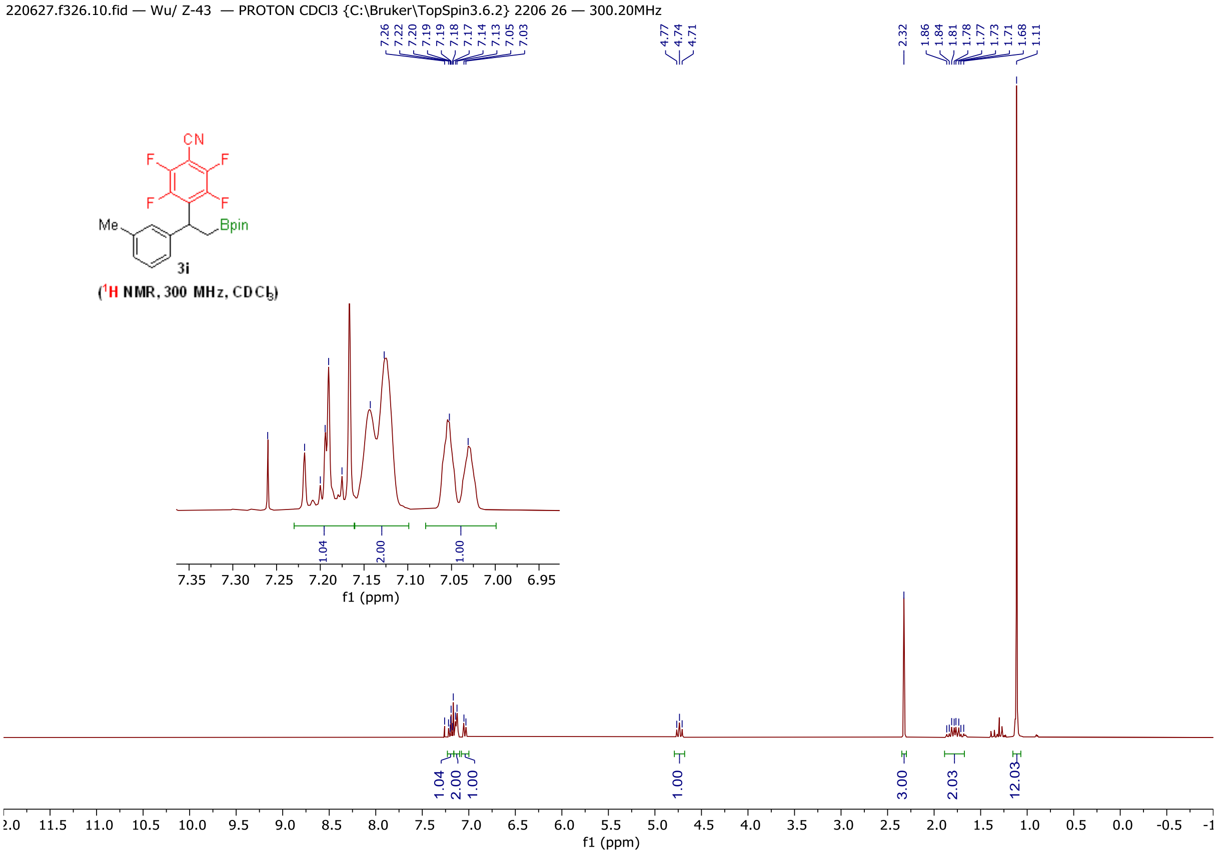

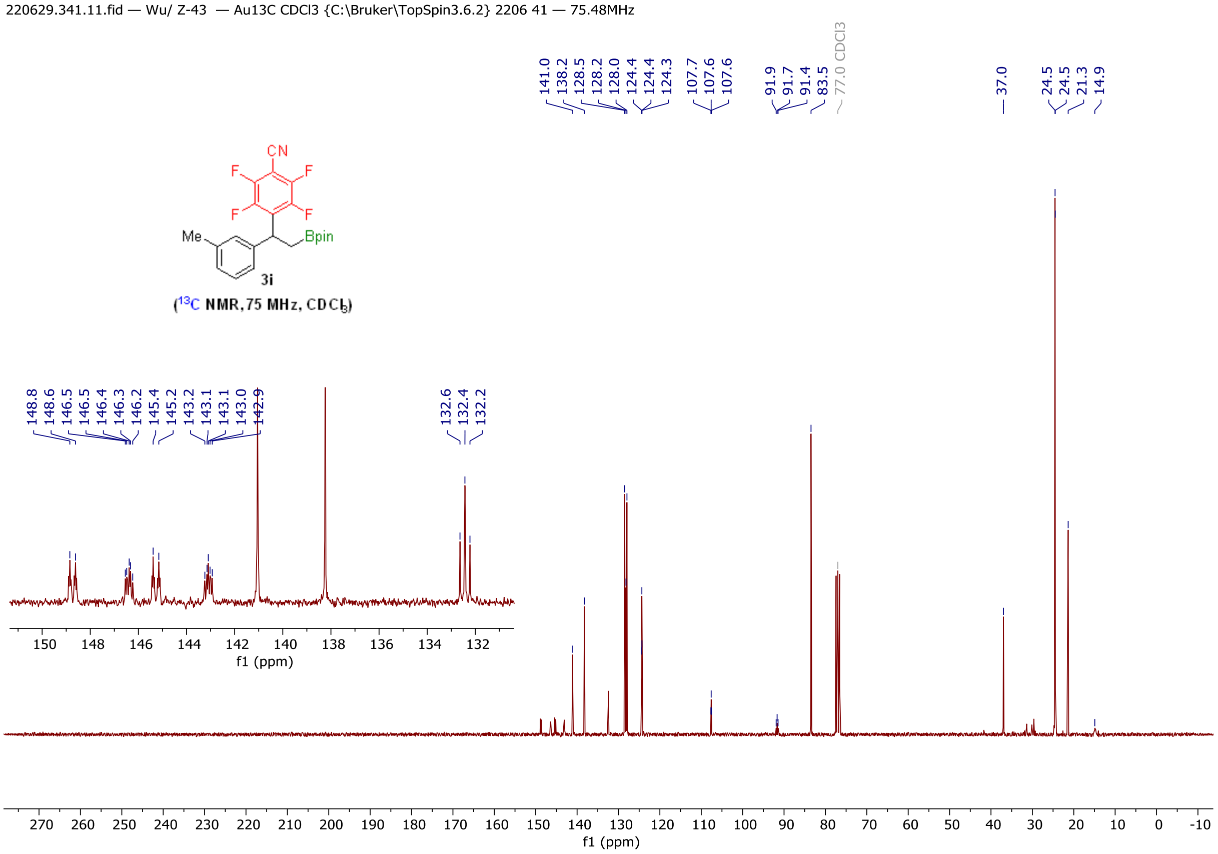

220629.341.12.fid — Wu/ Z-43 — Au19F CDCl3 {C:\Bruker\TopSpin3.6.2} 2206 41 — 282.39MHz

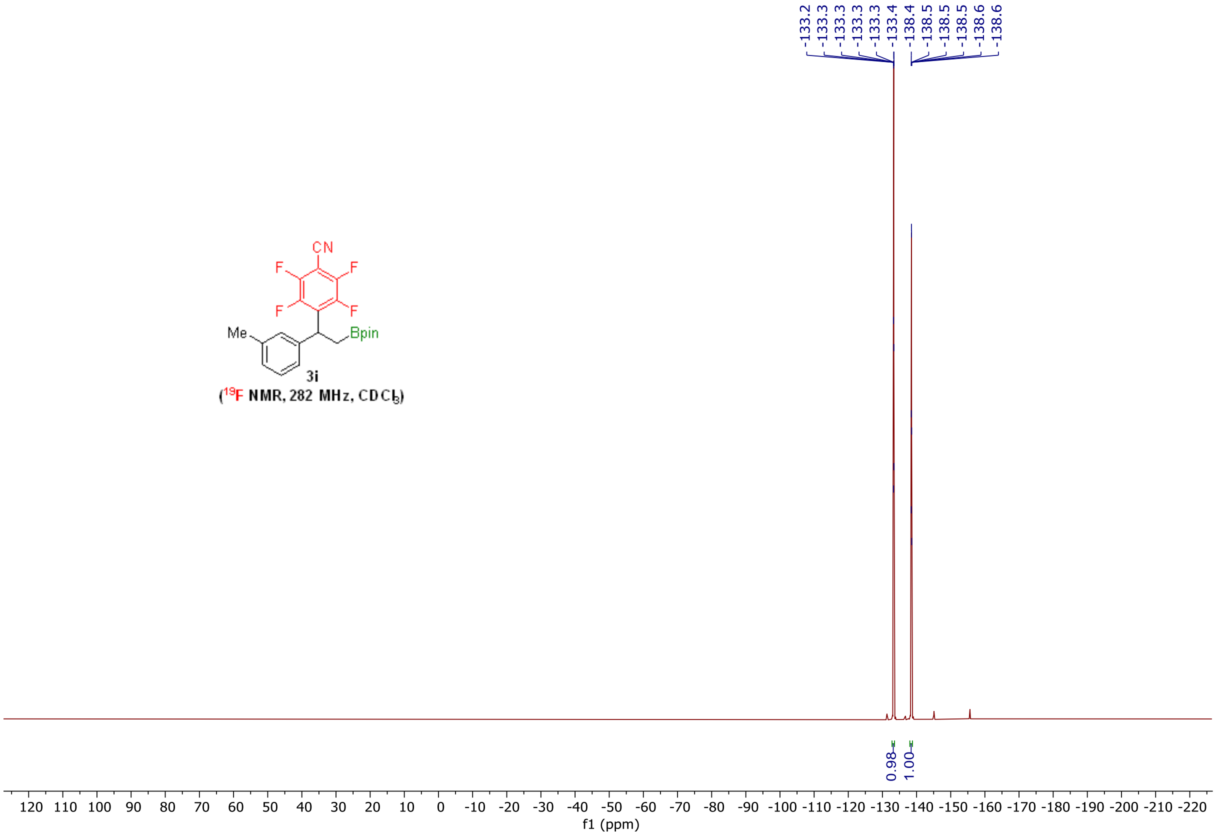

220629.341.13.fid — Wu/ Z-43 — Au11B CDCl3 {C:\Bruker\TopSpin3.6.2} 2206 41 — 96.29MHz

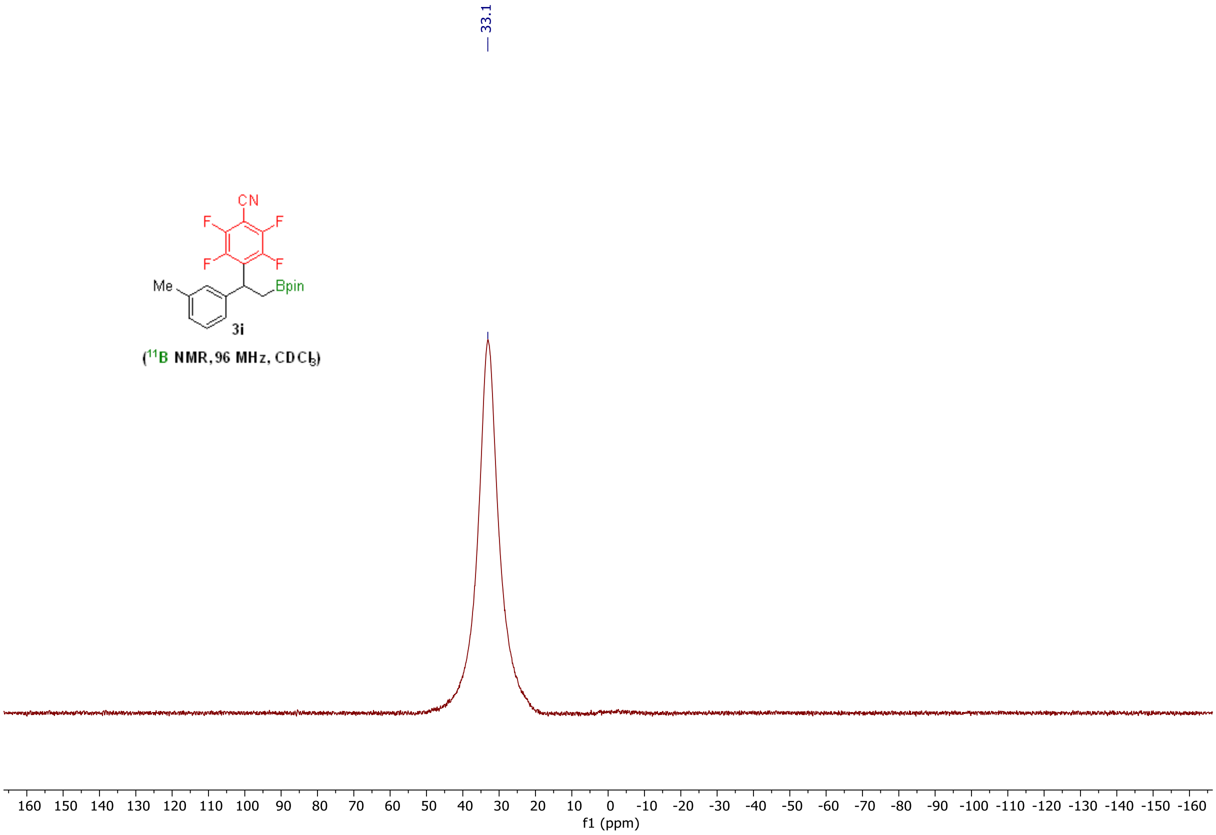

NMR Spectra of **3j**

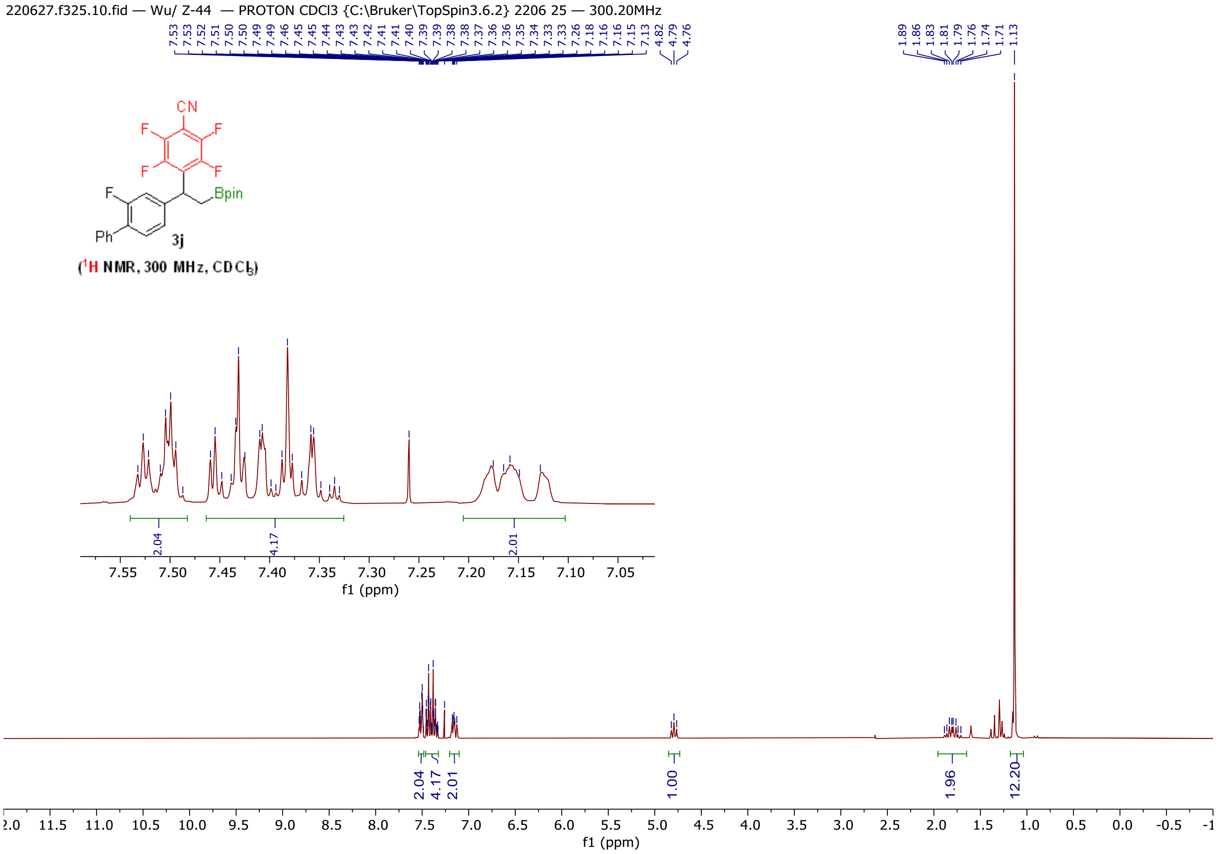

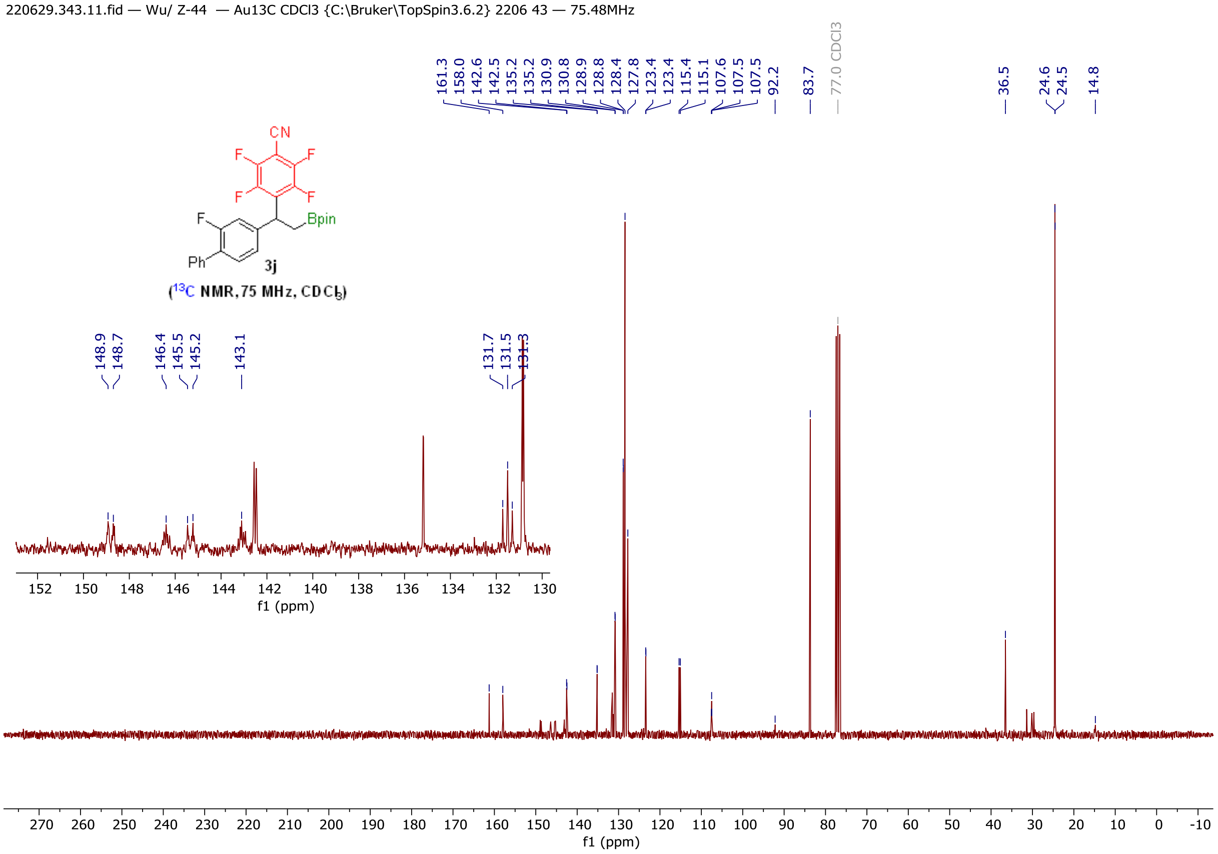

220629.343.12.fid — Wu/ Z-44 — Au19F CDCl3 {C:\Bruker\TopSpin3.6.2} 2206 43 — 282.39MHz

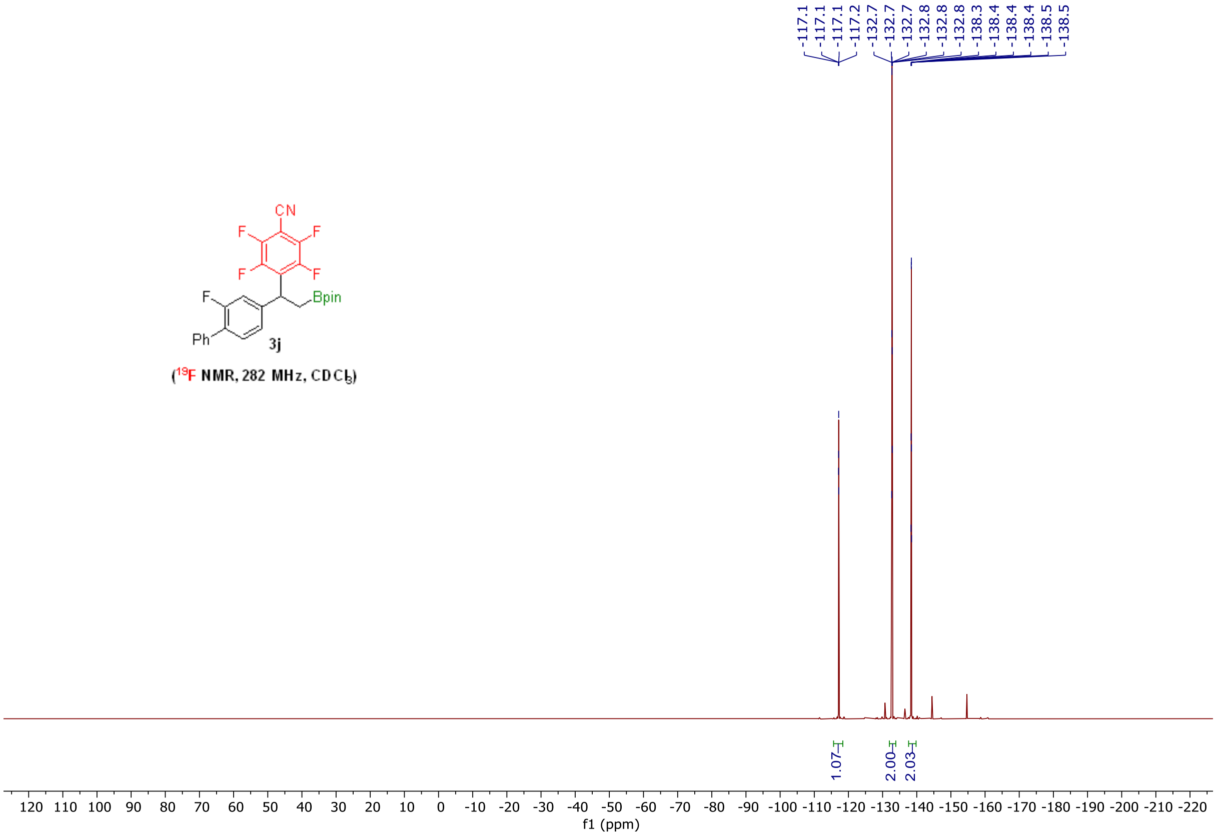

220629.343.13.fid — Wu/ Z-44 — Au11B CDCl3 {C:\Bruker\TopSpin3.6.2} 2206 43 — 96.29MHz

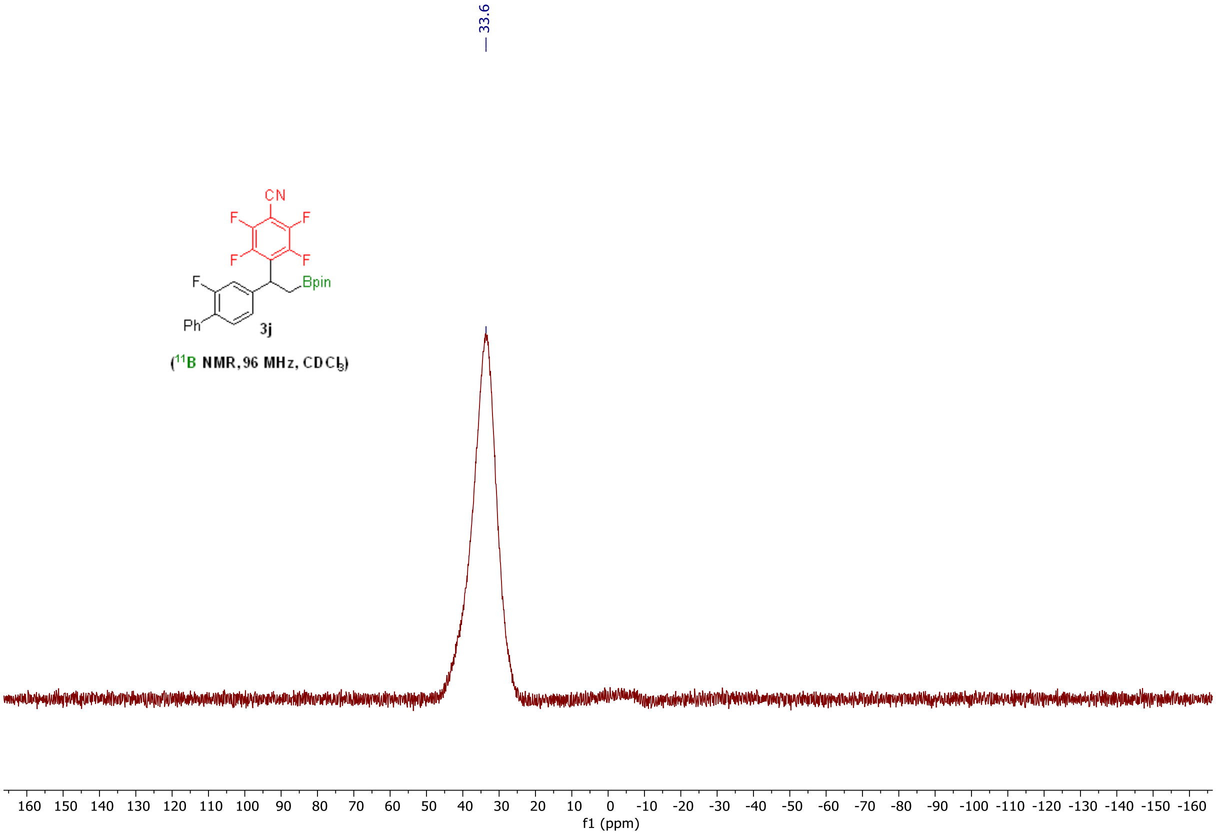

NMR Spectra of **3k**

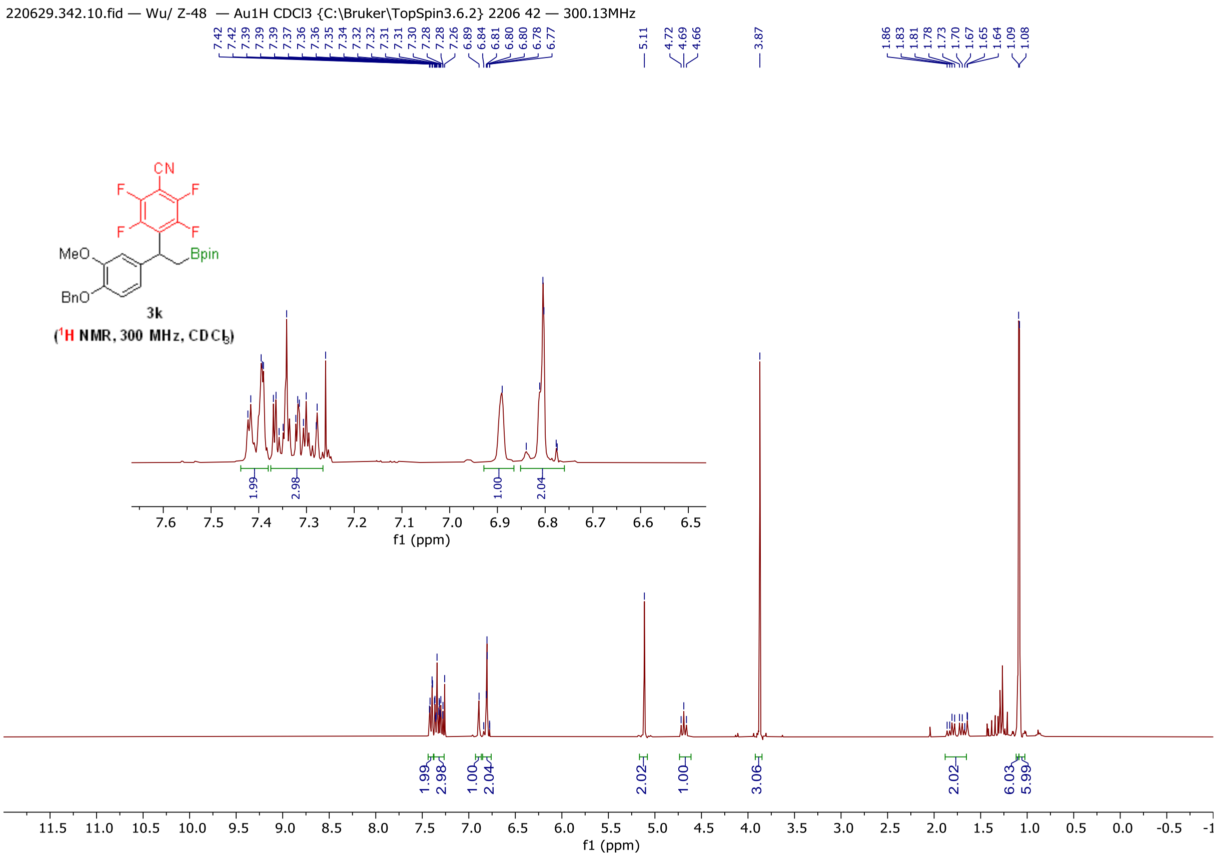

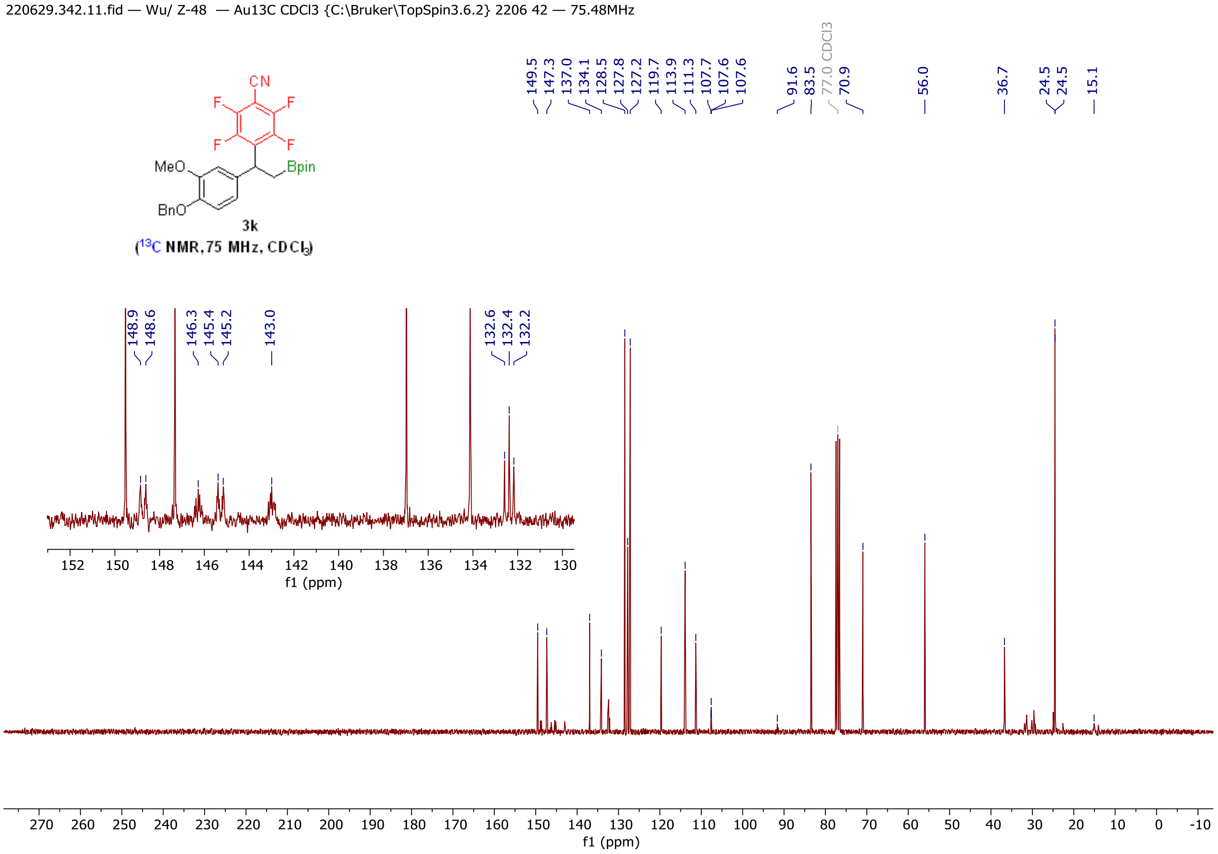

220629.342.12.fid — Wu/ Z-48 — Au19F CDCl3 {C:\Bruker\TopSpin3.6.2} 2206 42 — 282.39MHz

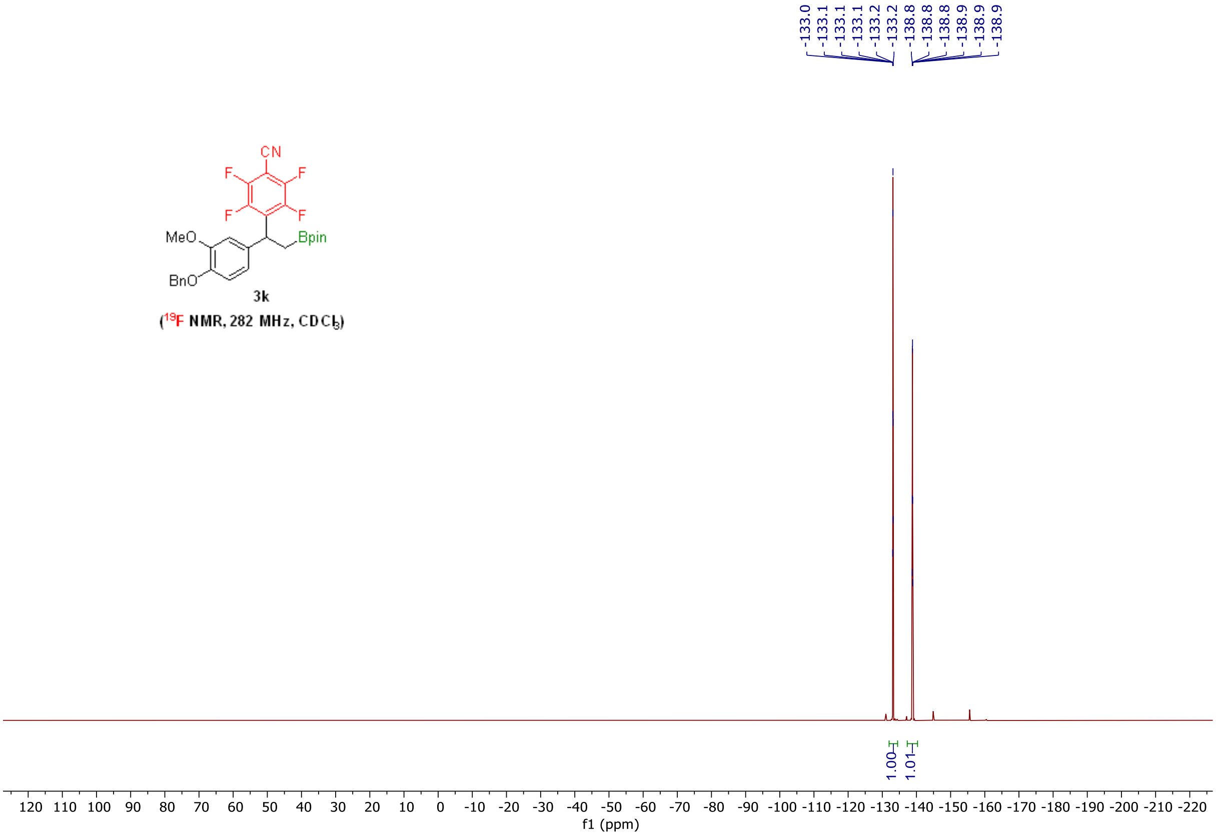

220629.342.13.fid — Wu/ Z-48 — Au11B CDCl3 {C:\Bruker\TopSpin3.6.2} 2206 42 — 96.29MHz

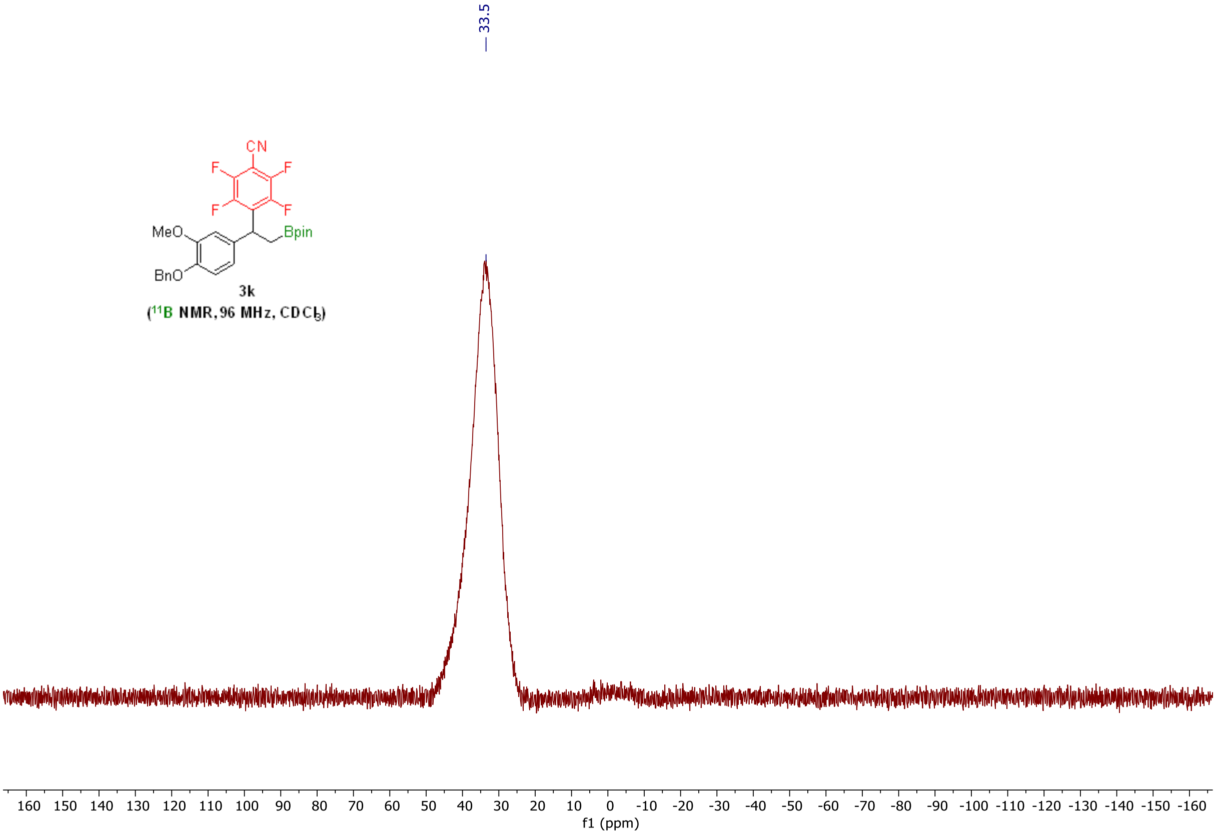

NMR Spectra of **3l**

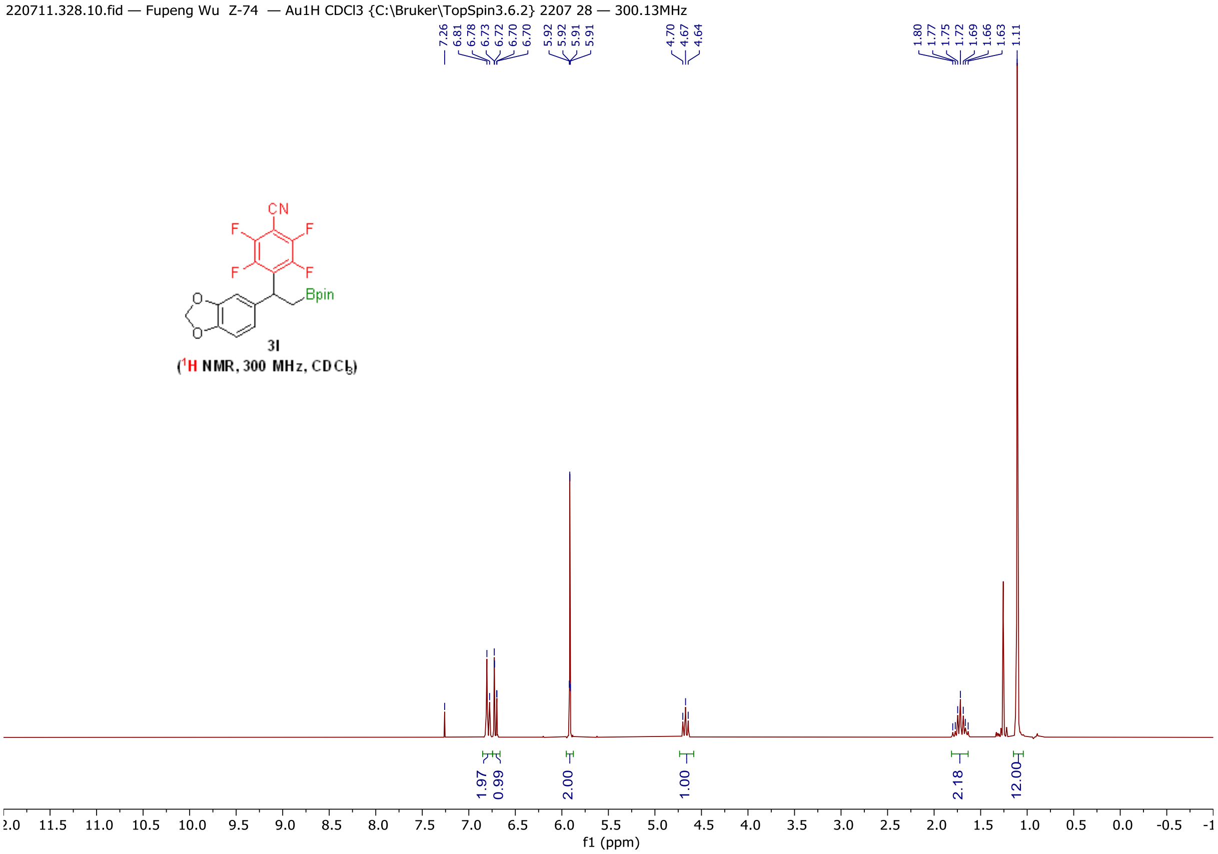

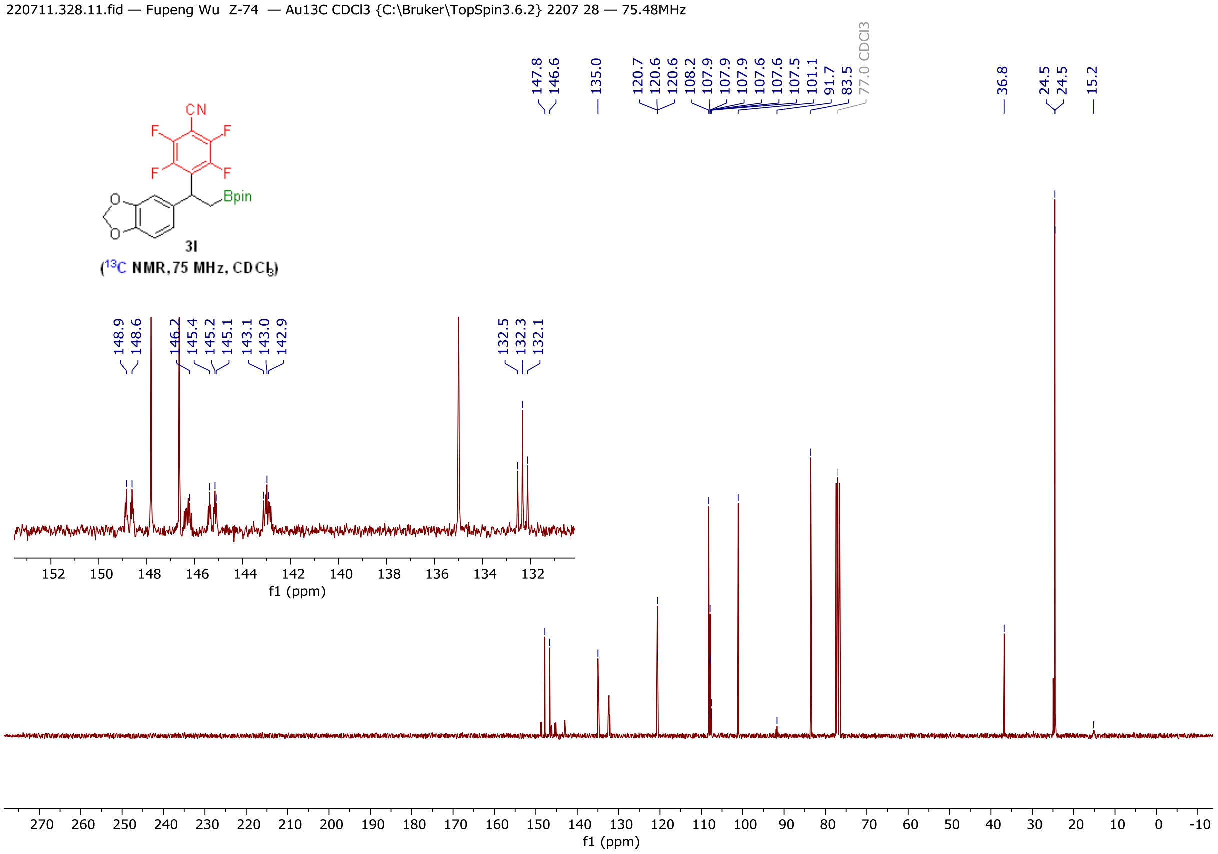

220711.328.12.fid — Fupeng Wu Z-74 — Au19F CDCl3 {C:\Bruker\TopSpin3.6.2} 2207 28 — 282.39MHz

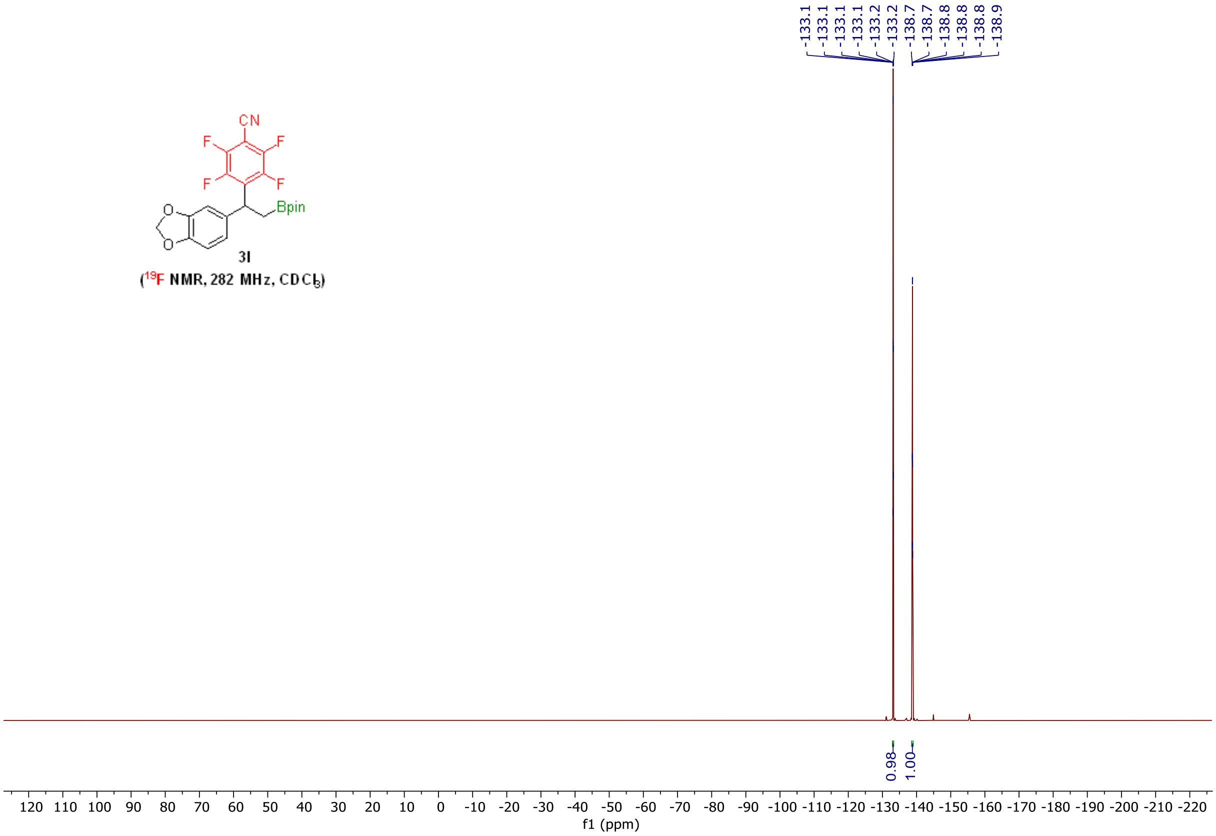

220711.328.13.fid — Fupeng Wu Z-74 — Au11B CDCl3 {C:\Bruker\TopSpin3.6.2} 2207 28 — 96.29MHz

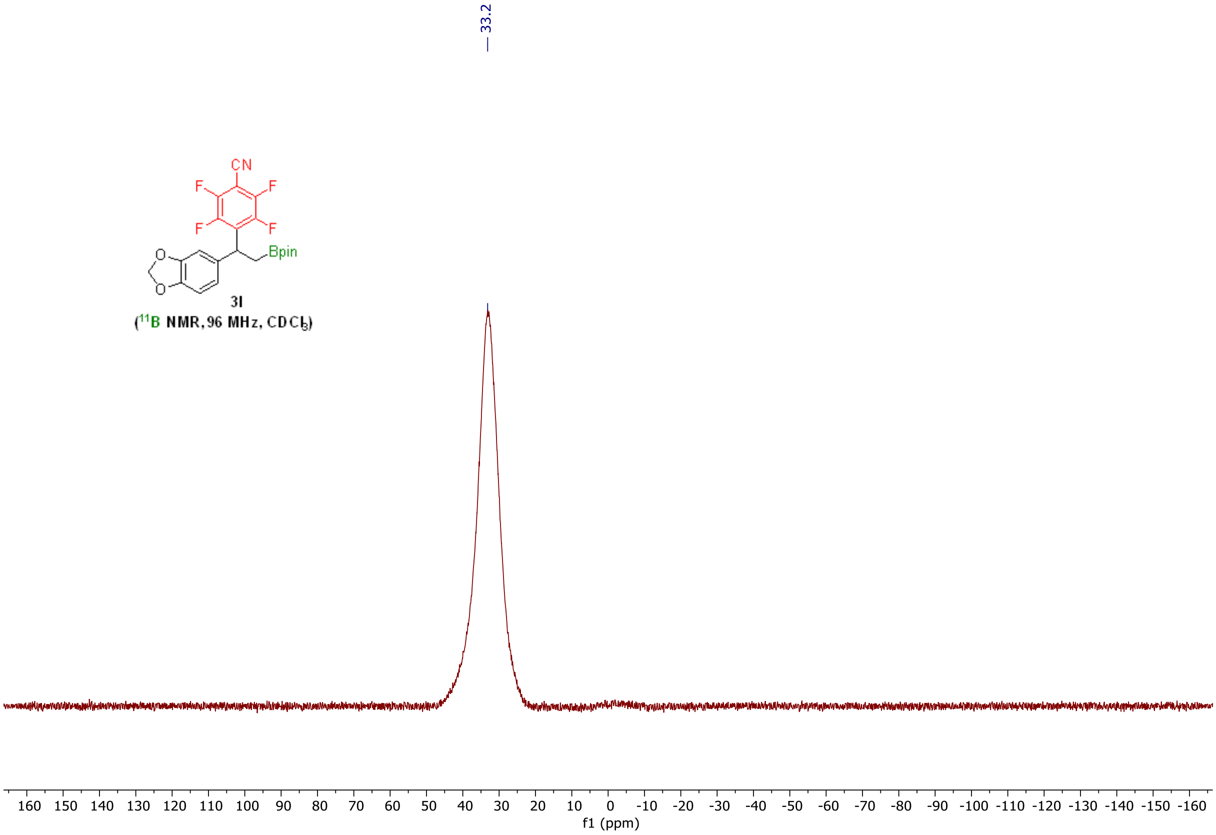

NMR Spectra of **3m**

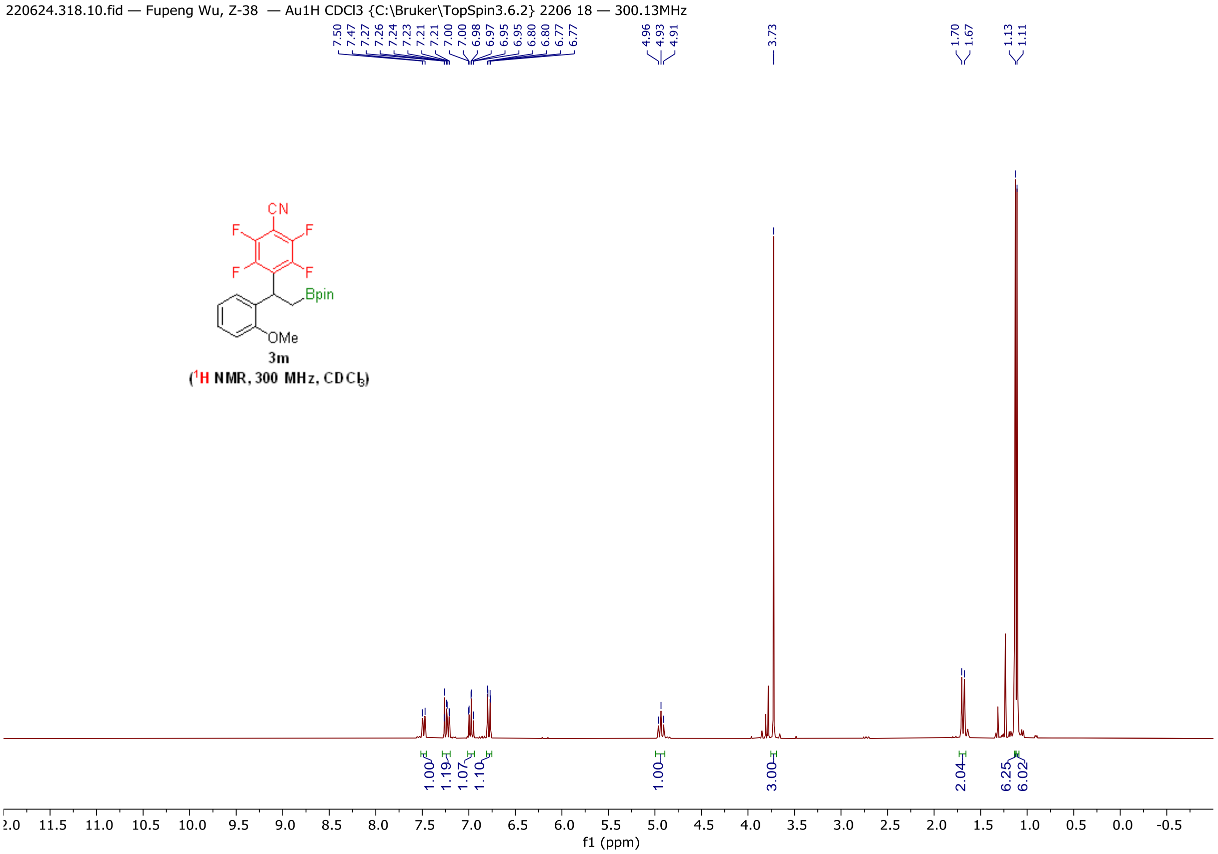

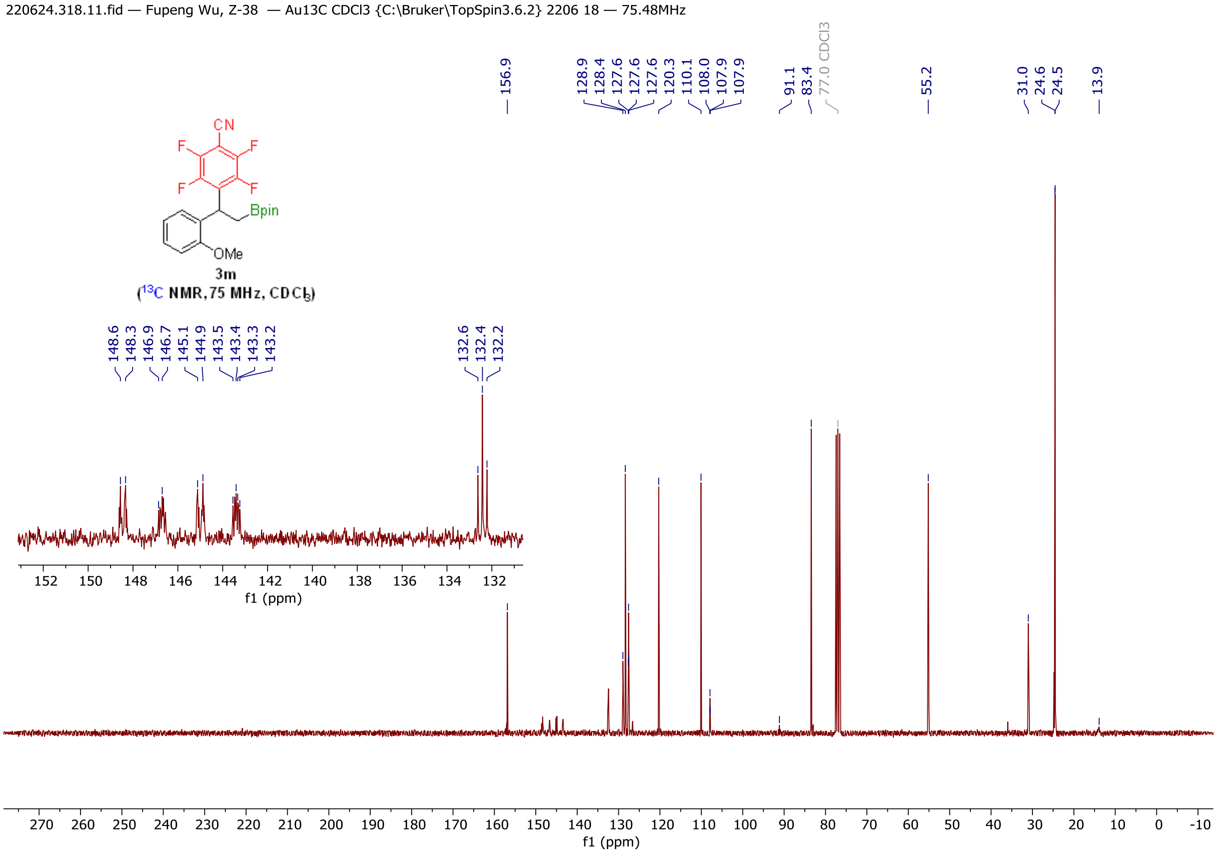

220624.318.12.fid — Fupeng Wu, Z-38 — Au19F CDCl3 {C:\Bruker\TopSpin3.6.2} 2206 18 — 282.39MHz

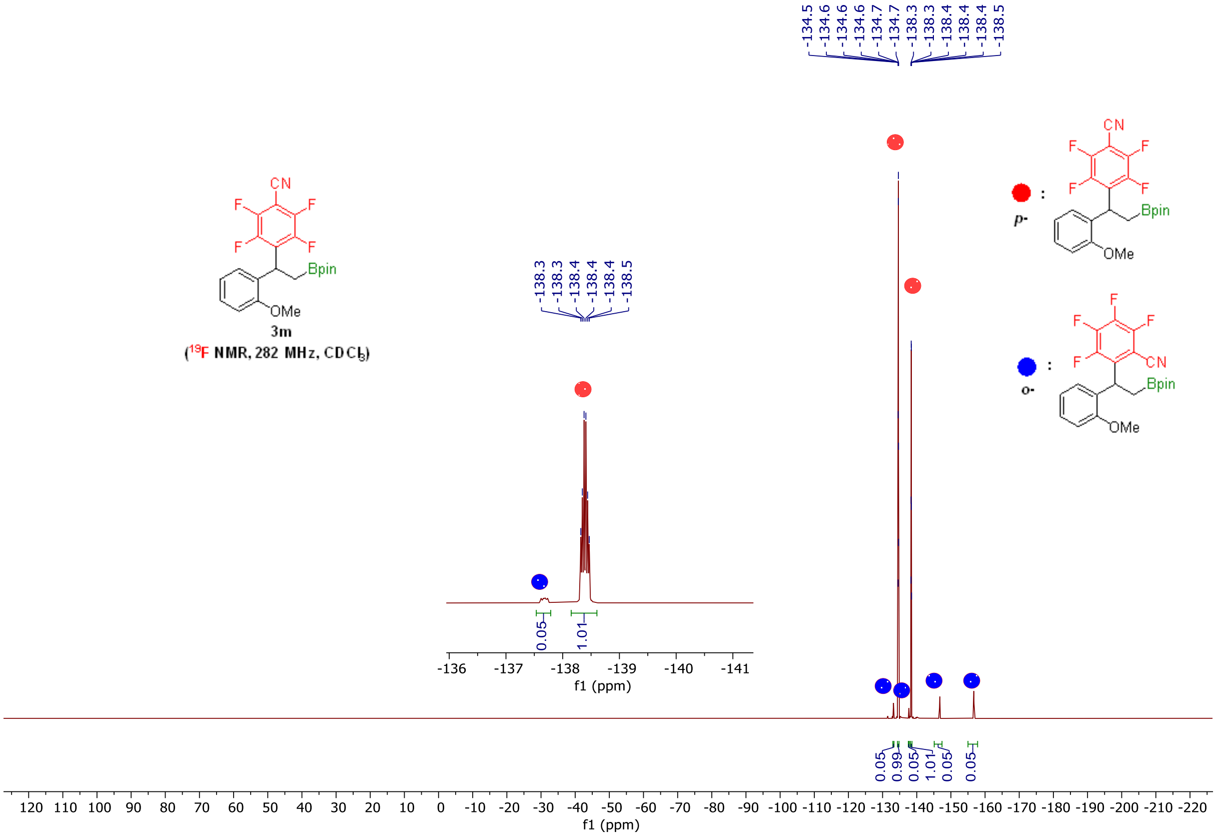

220624.318.13.fid — Fupeng Wu, Z-38 — Au11B CDCl3 {C:\Bruker\TopSpin3.6.2} 2206 18 — 96.29MHz

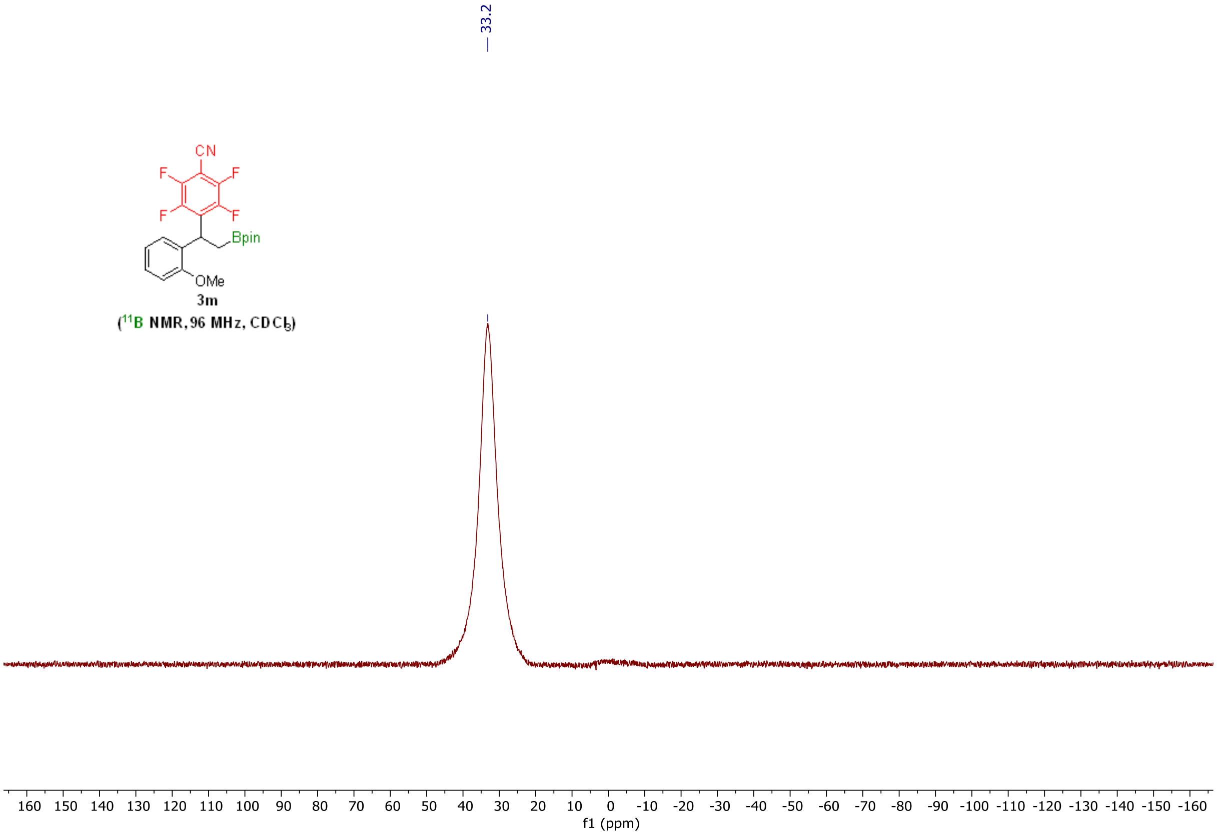

NMR Spectra of **3n**

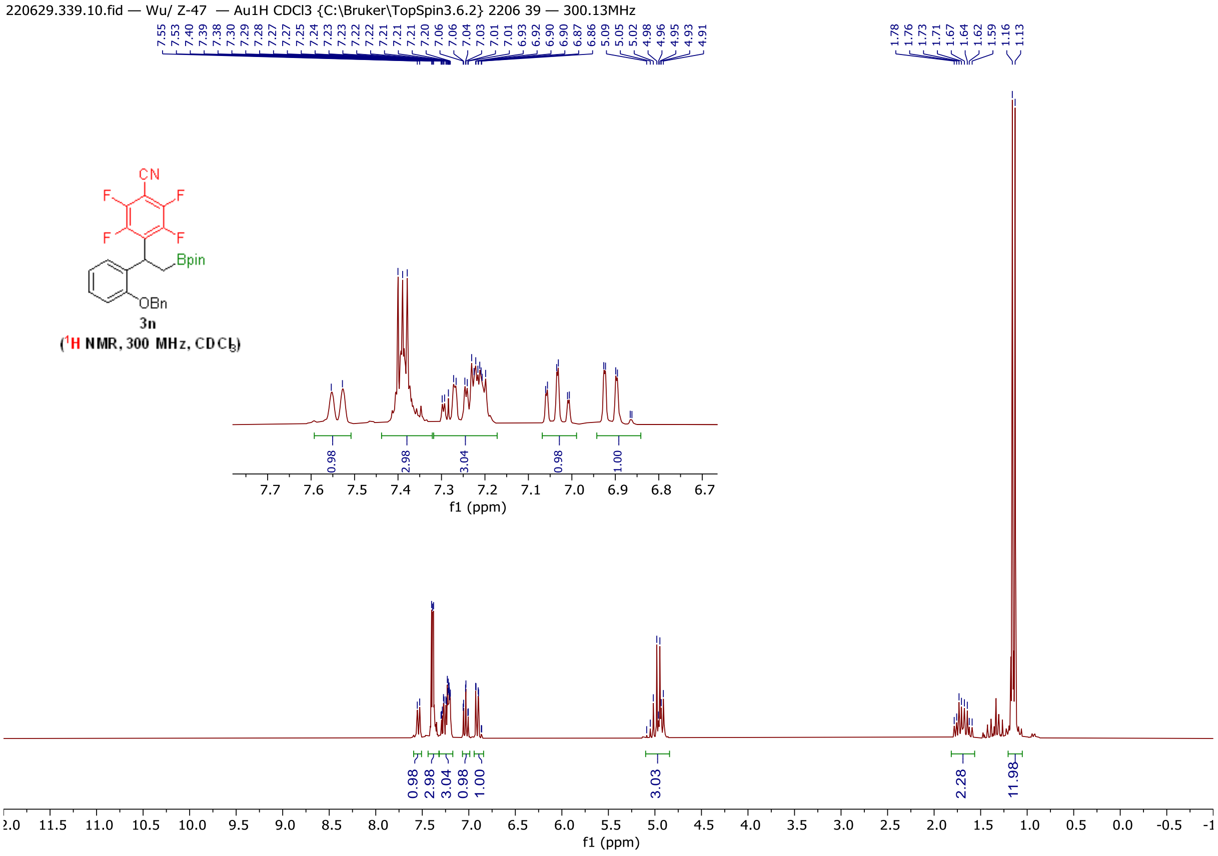

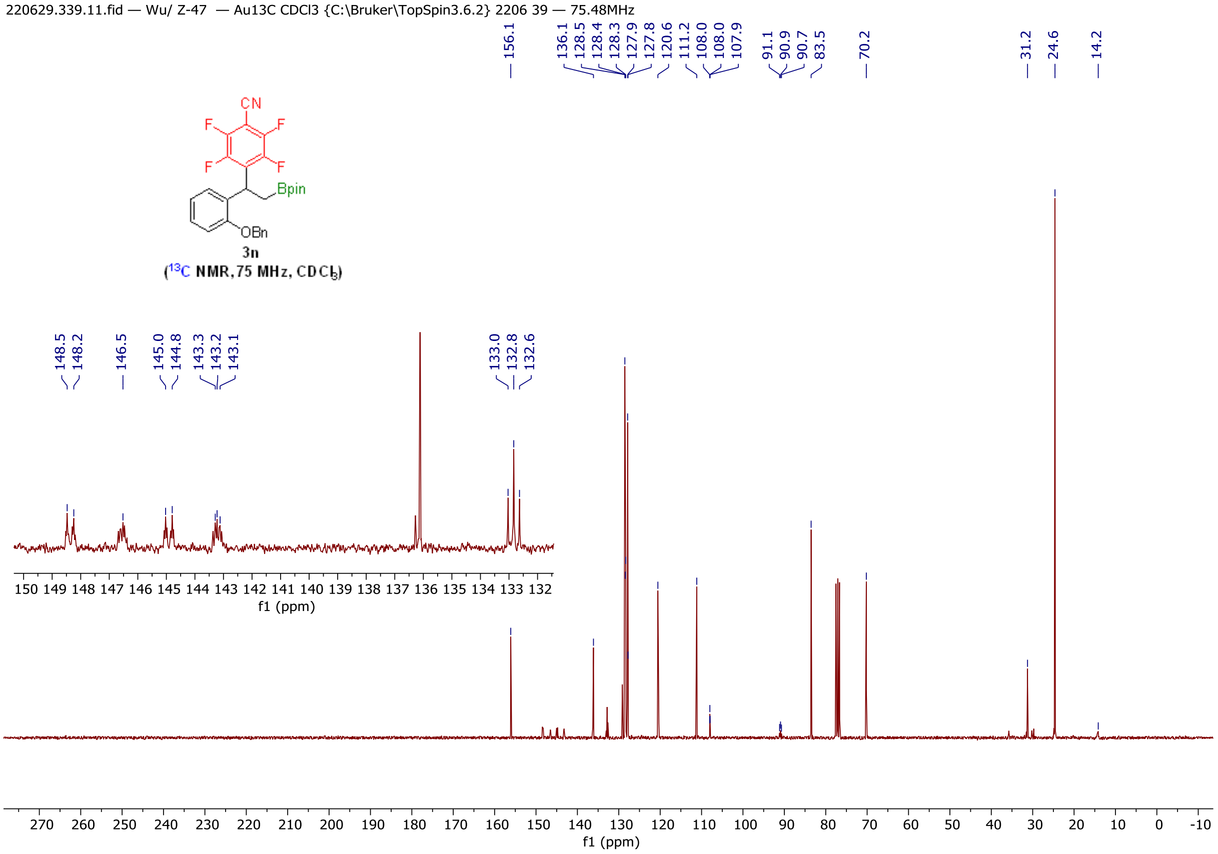

220629.339.12.fid — Wu/ Z-47 — Au19F CDCl3 {C:\Bruker\TopSpin3.6.2} 2206 39 — 282.39MHz

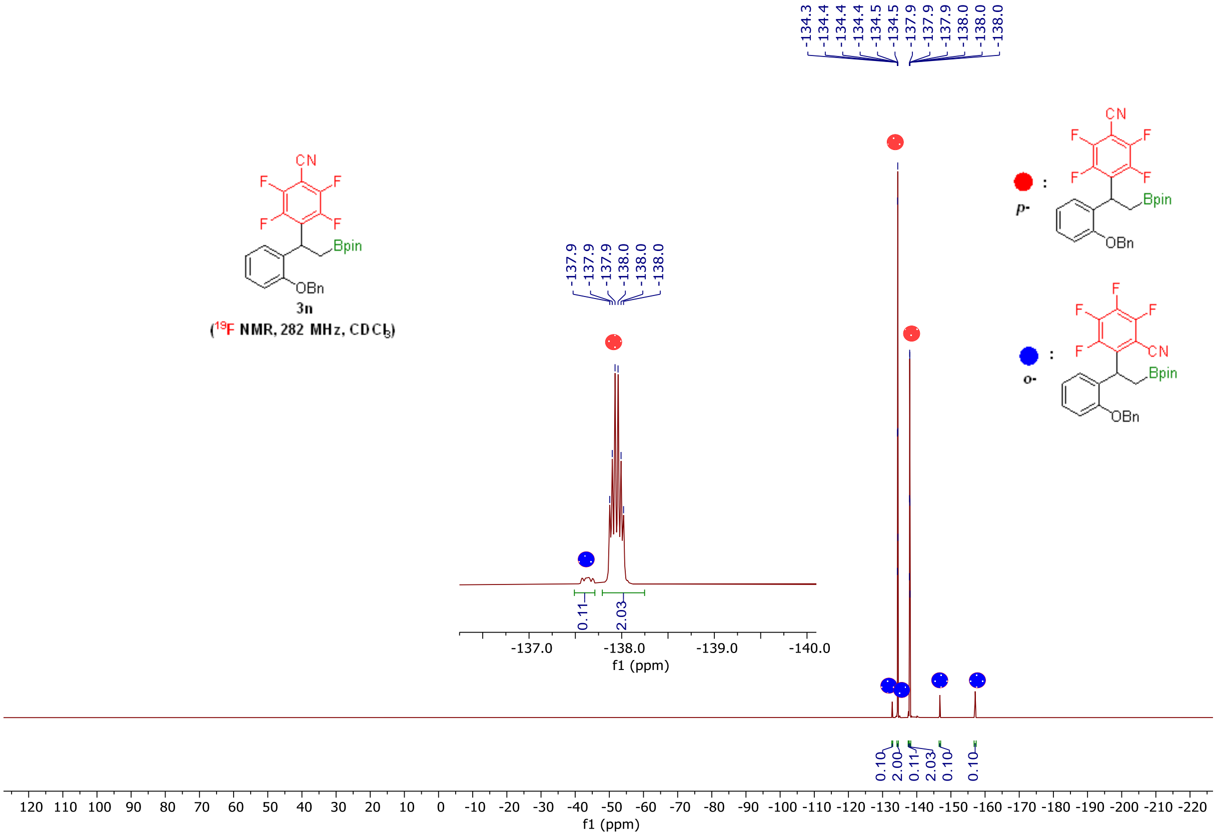

220629.339.13.fid — Wu/ Z-47 — Au11B CDCl3 {C:\Bruker\TopSpin3.6.2} 2206 39 — 96.29MHz

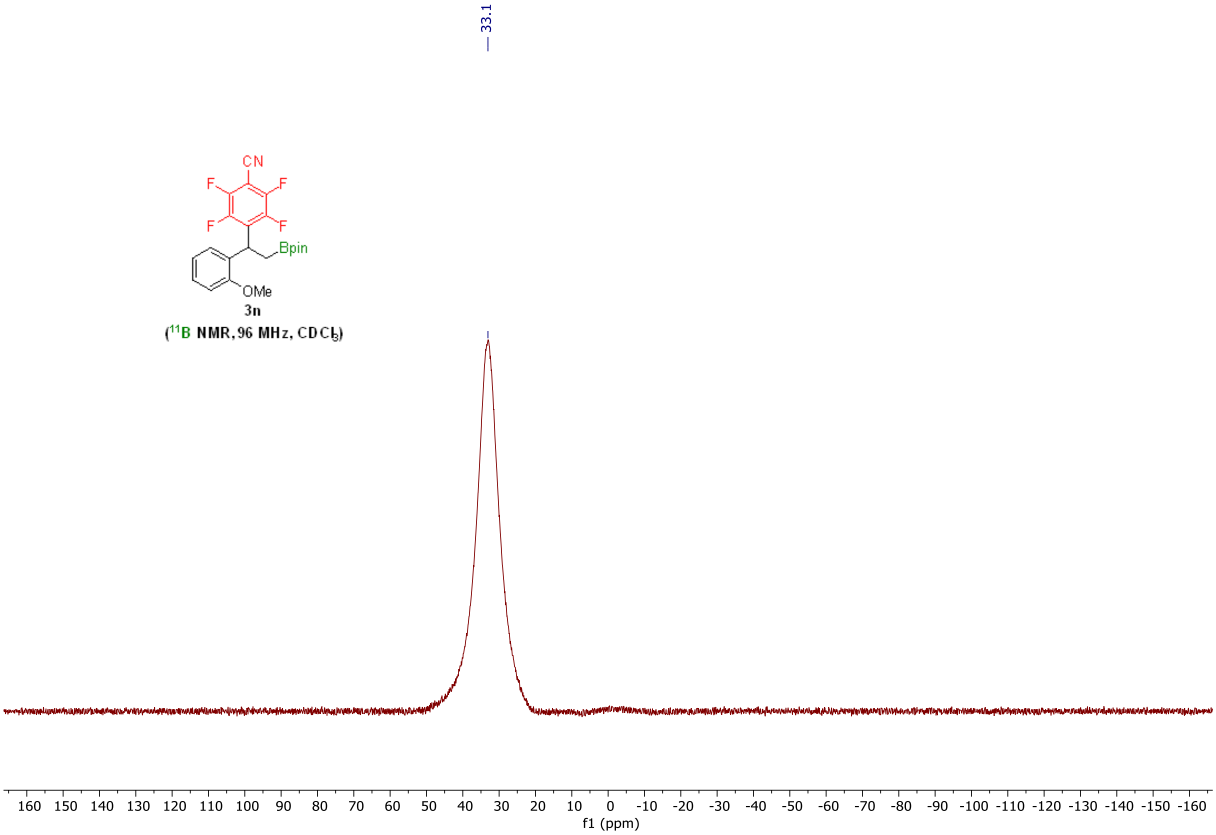

NMR Spectra of **3o**

220628.f339.10.fid — Wu/ Z-7 — PROTON CDCl3 {C:\Bruker\TopSpin3.6.2} 2206 39 — 300.20MHz

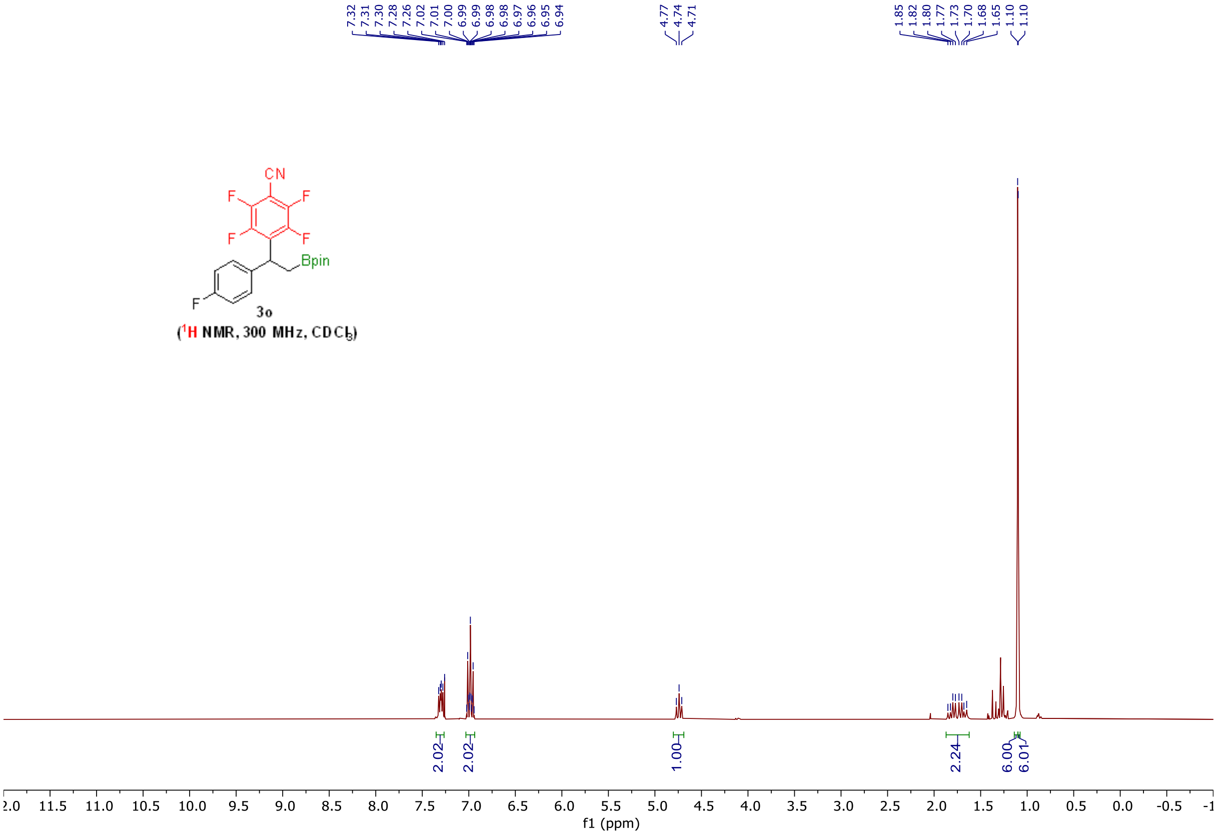

220628.f339.11.fid — Wu/ Z-7 — C13CPD CDCl3 {C:\Bruker\TopSpin3.6.2} 2206 39 — 75.49MHz

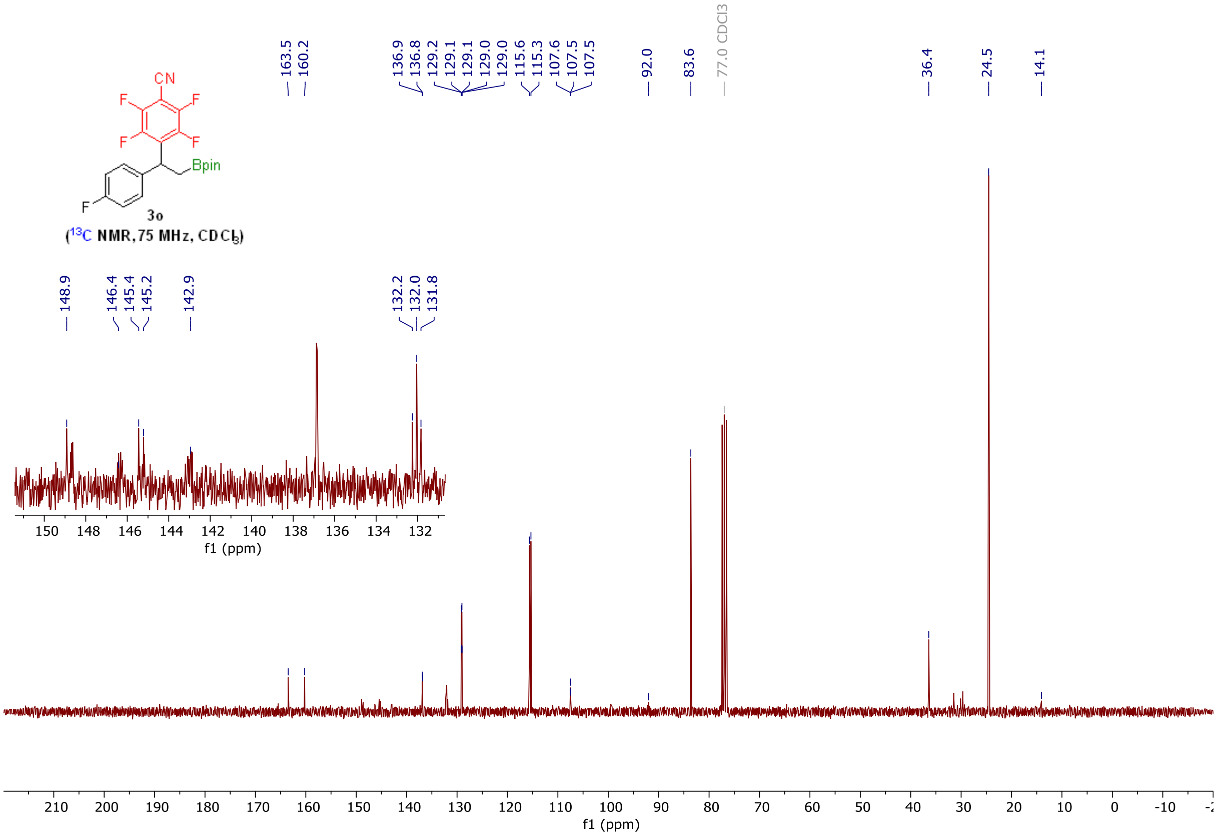

220628.f339.12.fid — Wu/ Z-7 — F19 CDCl3 {C:\Bruker\TopSpin3.6.2} 2206 39 — 282.44MHz

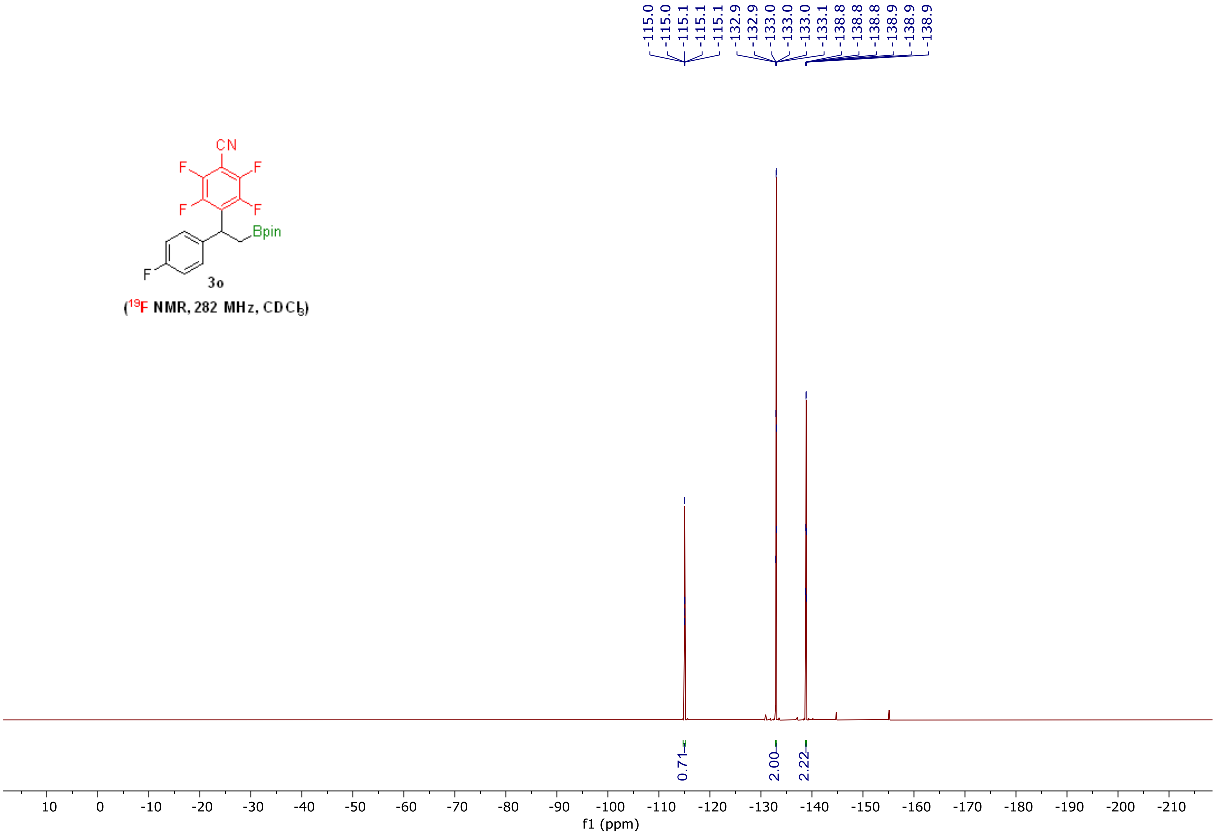

220628.f339.13.fid — Wu/ Z-7 — 11B CDCl3 {C:\Bruker\TopSpin3.6.2} 2206 39 — 96.32MHz

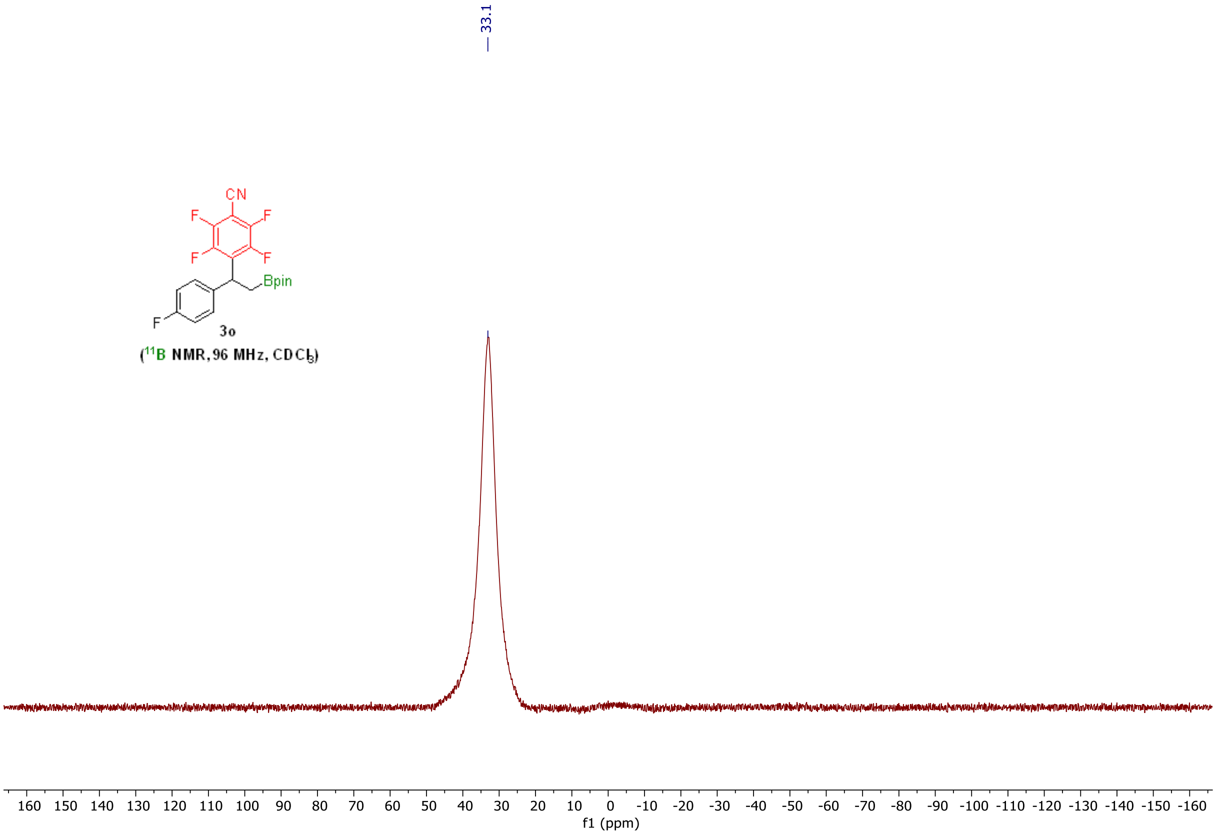

NMR Spectra of **3p**

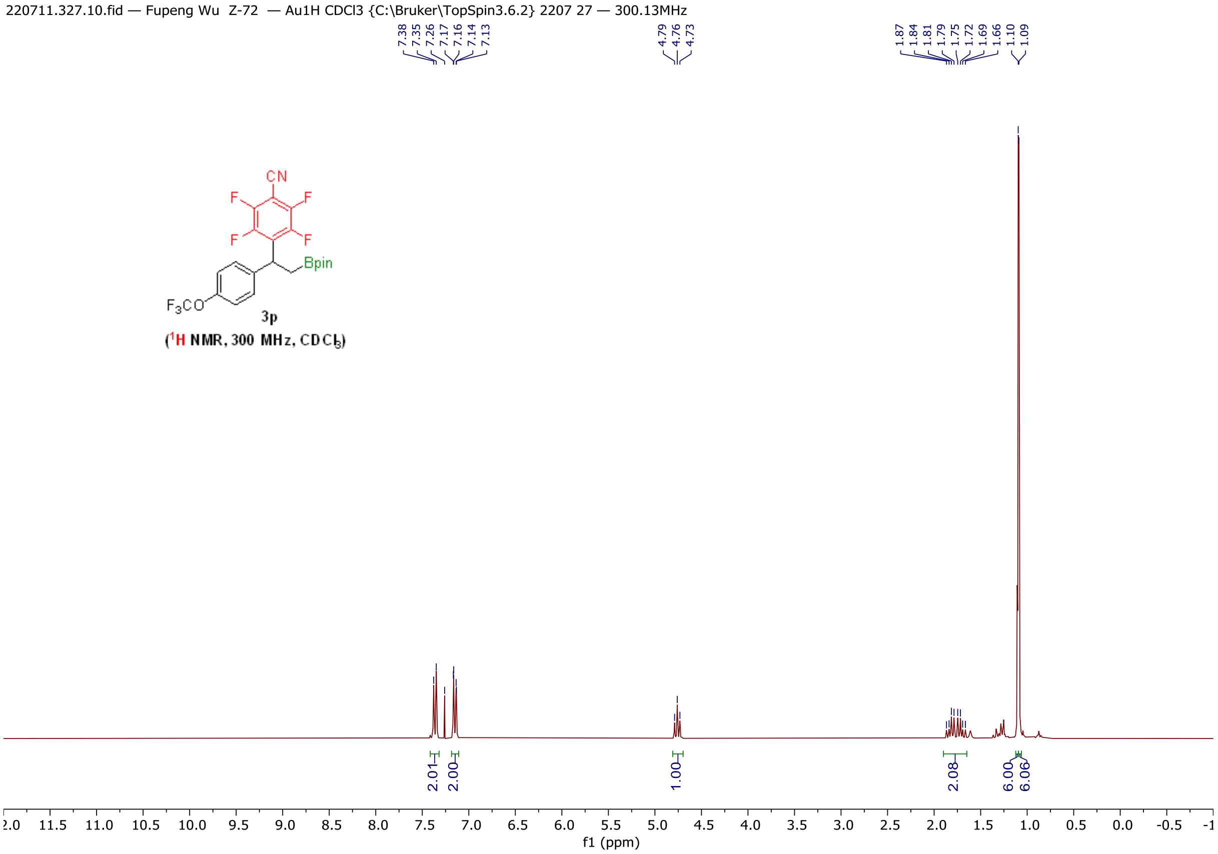

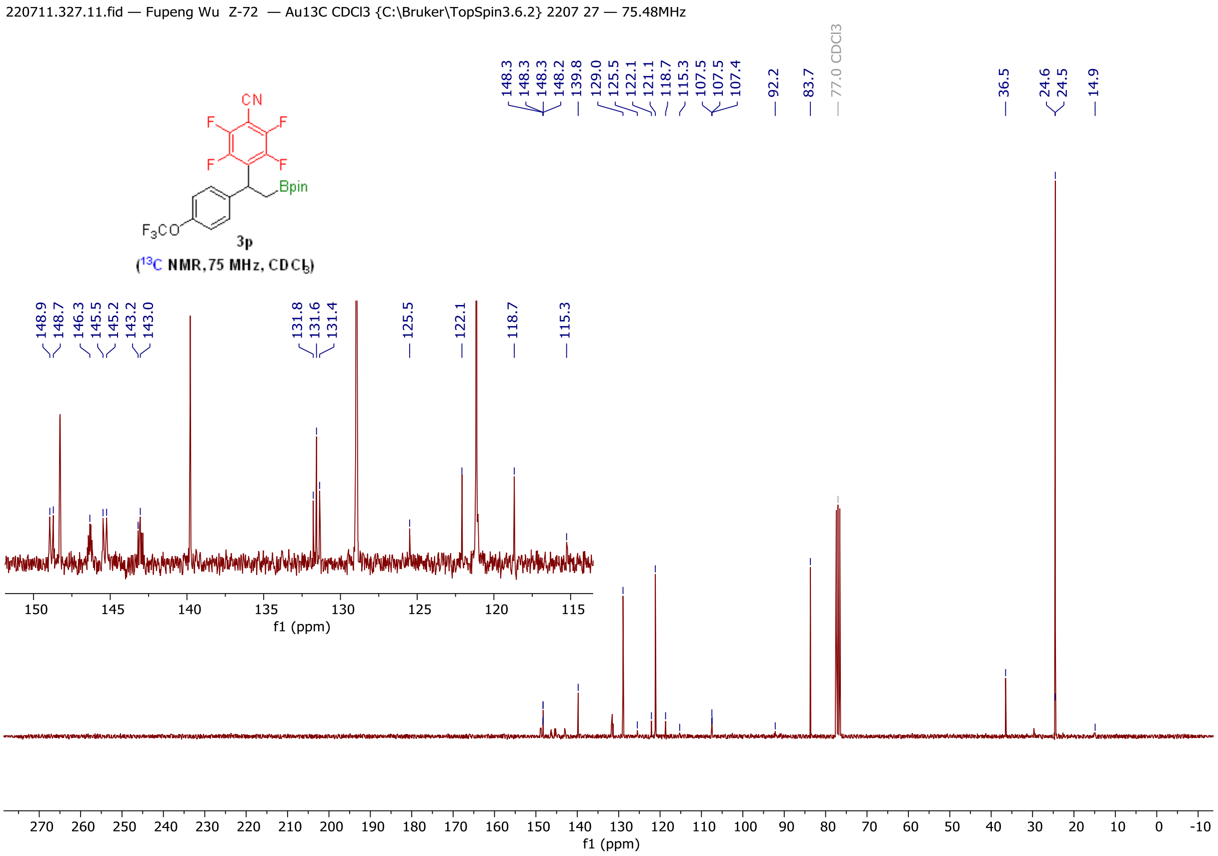

220711.327.12.fid — Fupeng Wu Z-72 — Au19F CDCl3 {C:\Bruker\TopSpin3.6.2} 2207 27 — 282.39MHz

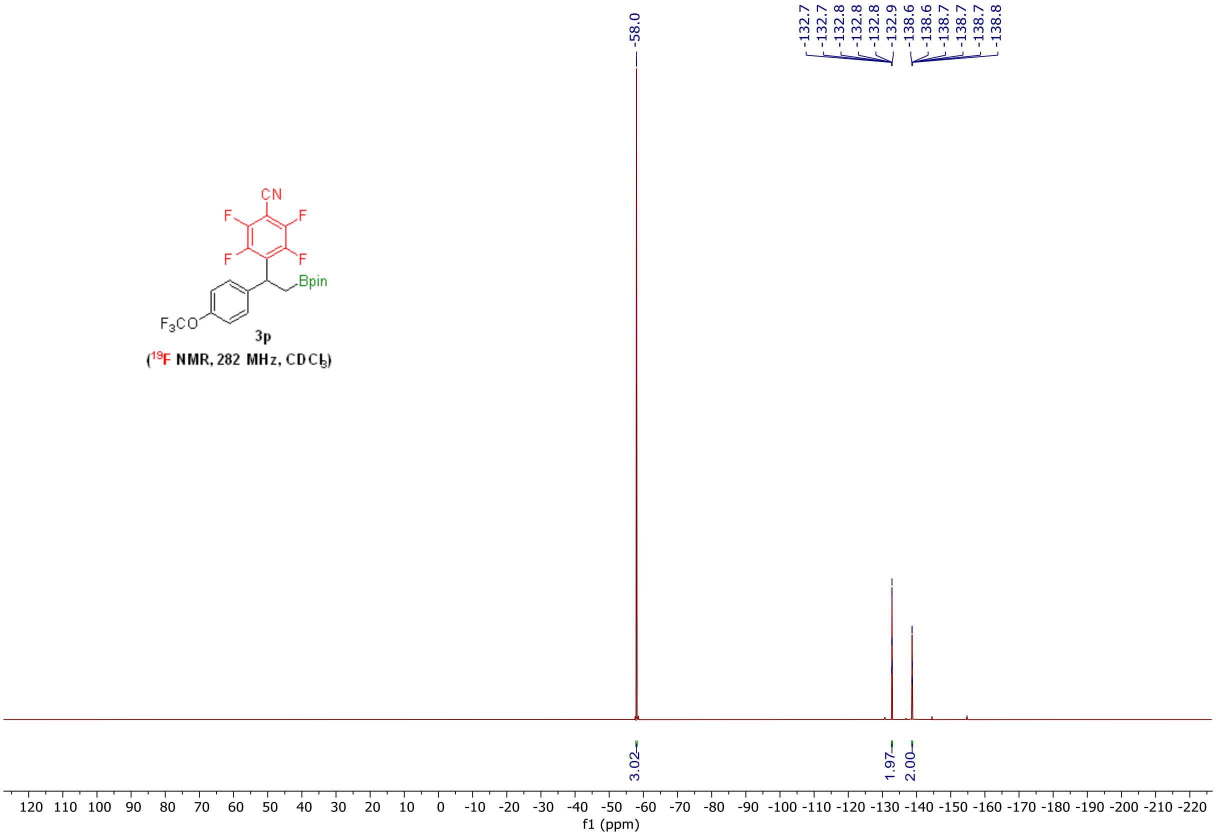

220711.327.13.fid — Fupeng Wu Z-72 — Au11B CDCl3 {C:\Bruker\TopSpin3.6.2} 2207 27 — 96.29MHz

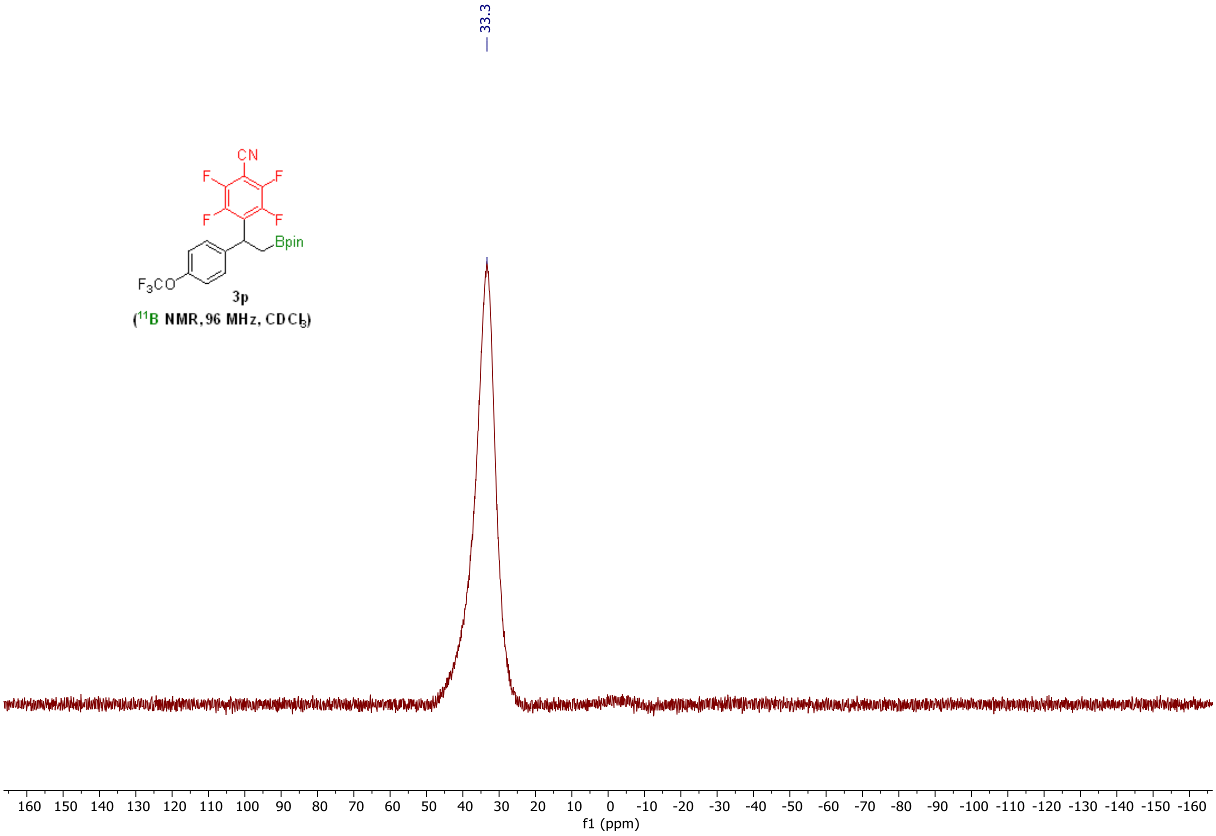

NMR Spectra of **3q**

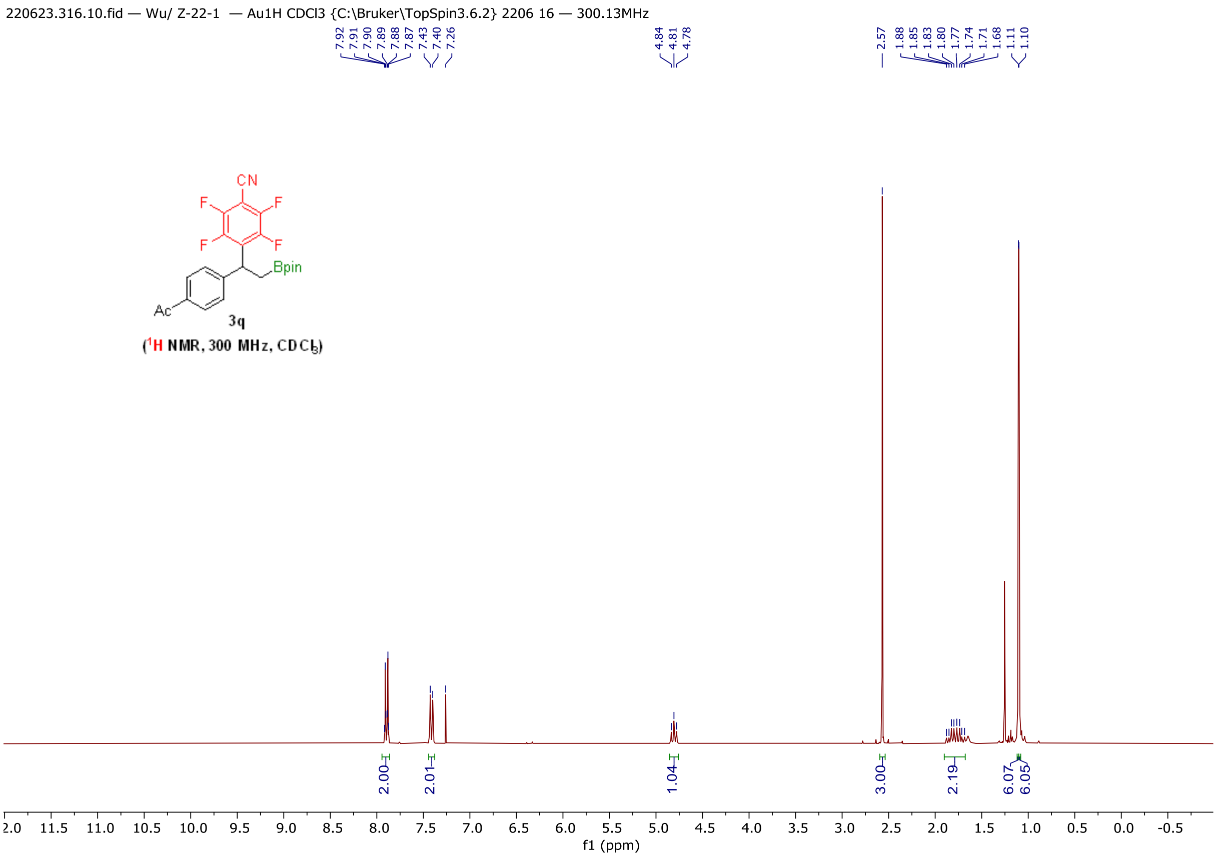

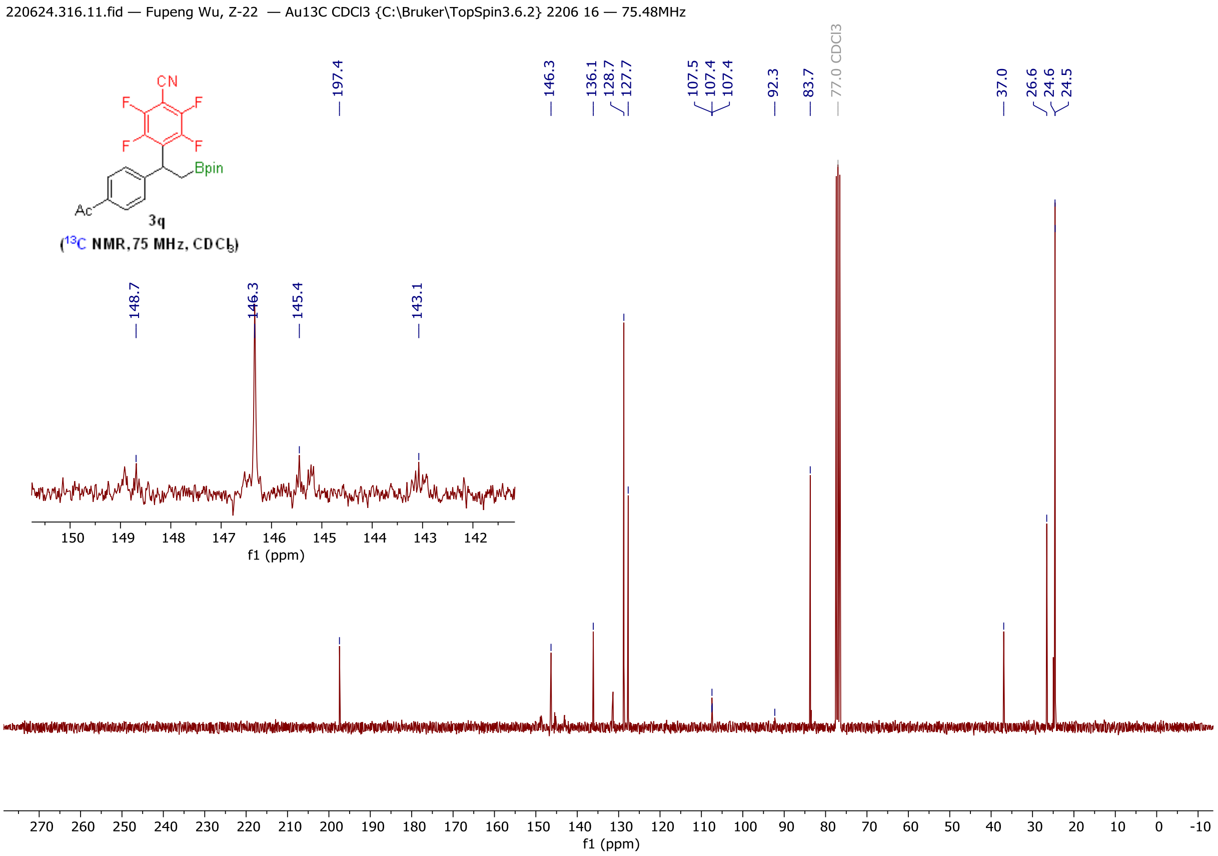

220624.316.12.fid — Fupeng Wu, Z-22 — Au19F CDCl3 {C:\Bruker\TopSpin3.6.2} 2206 16 — 282.39MHz

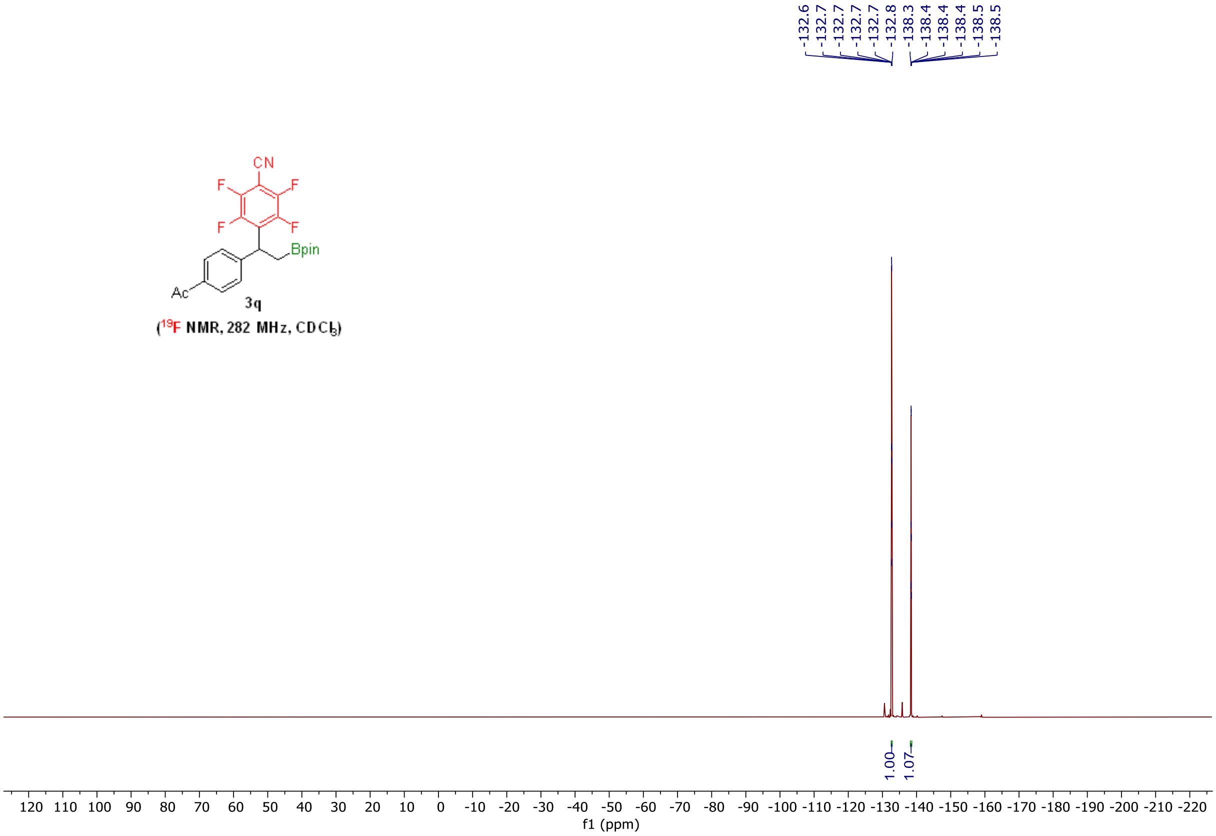

220624.316.13.fid — Fupeng Wu, Z-22 — Au11B CDCl3 {C:\Bruker\TopSpin3.6.2} 2206 16 — 96.29MHz

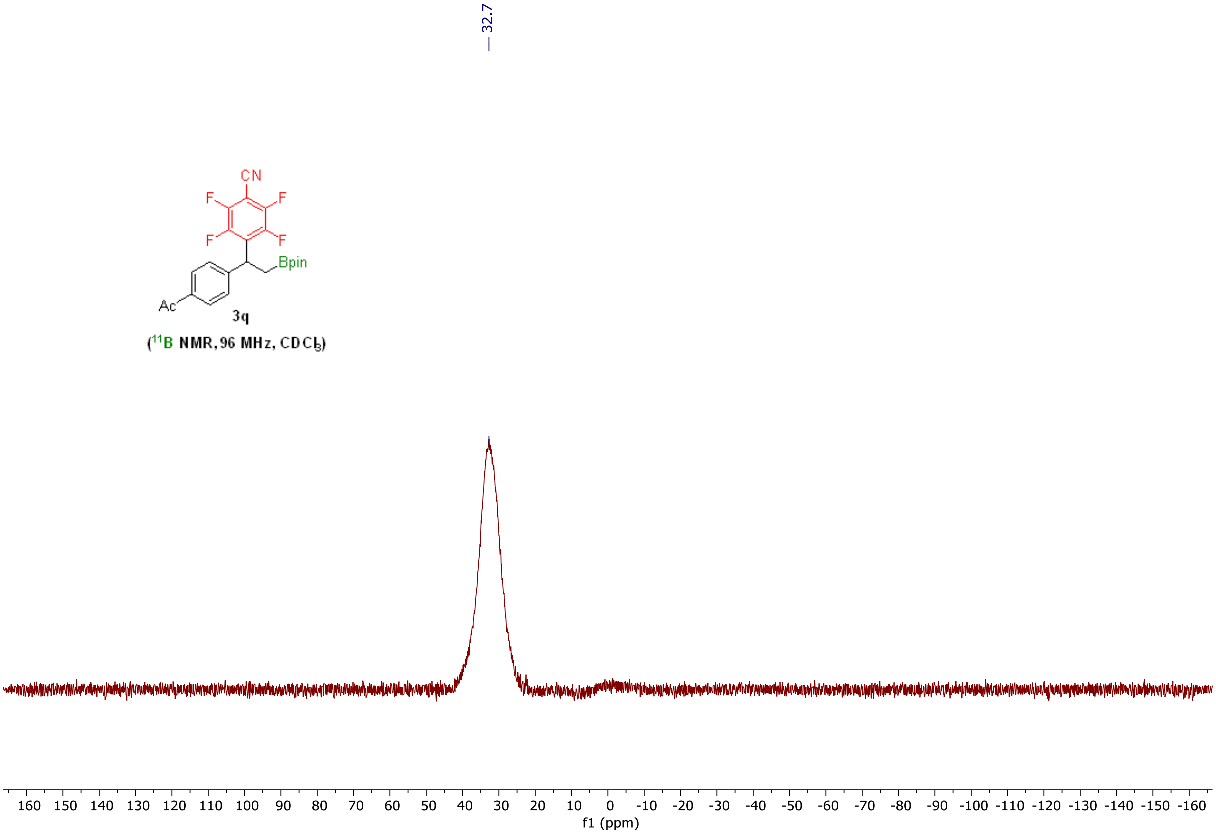

NMR Spectra of **3r**

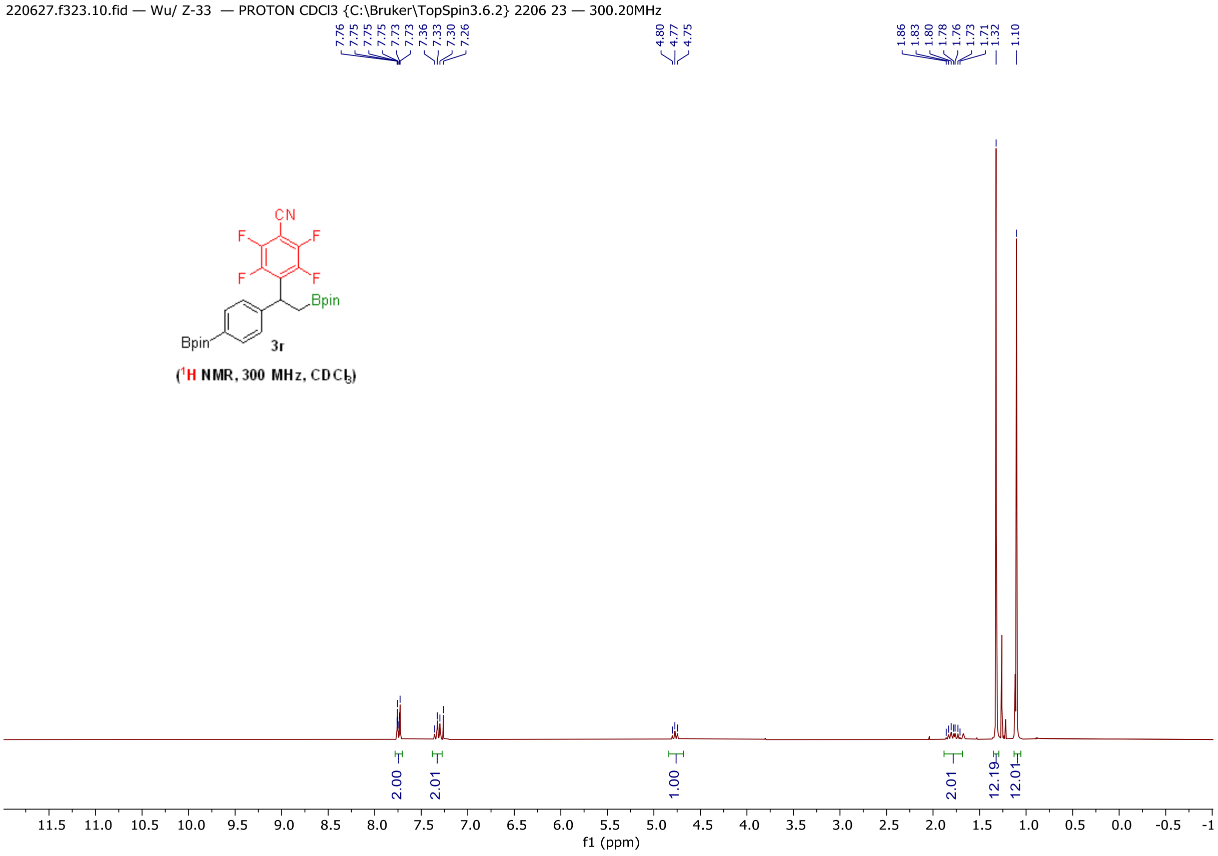

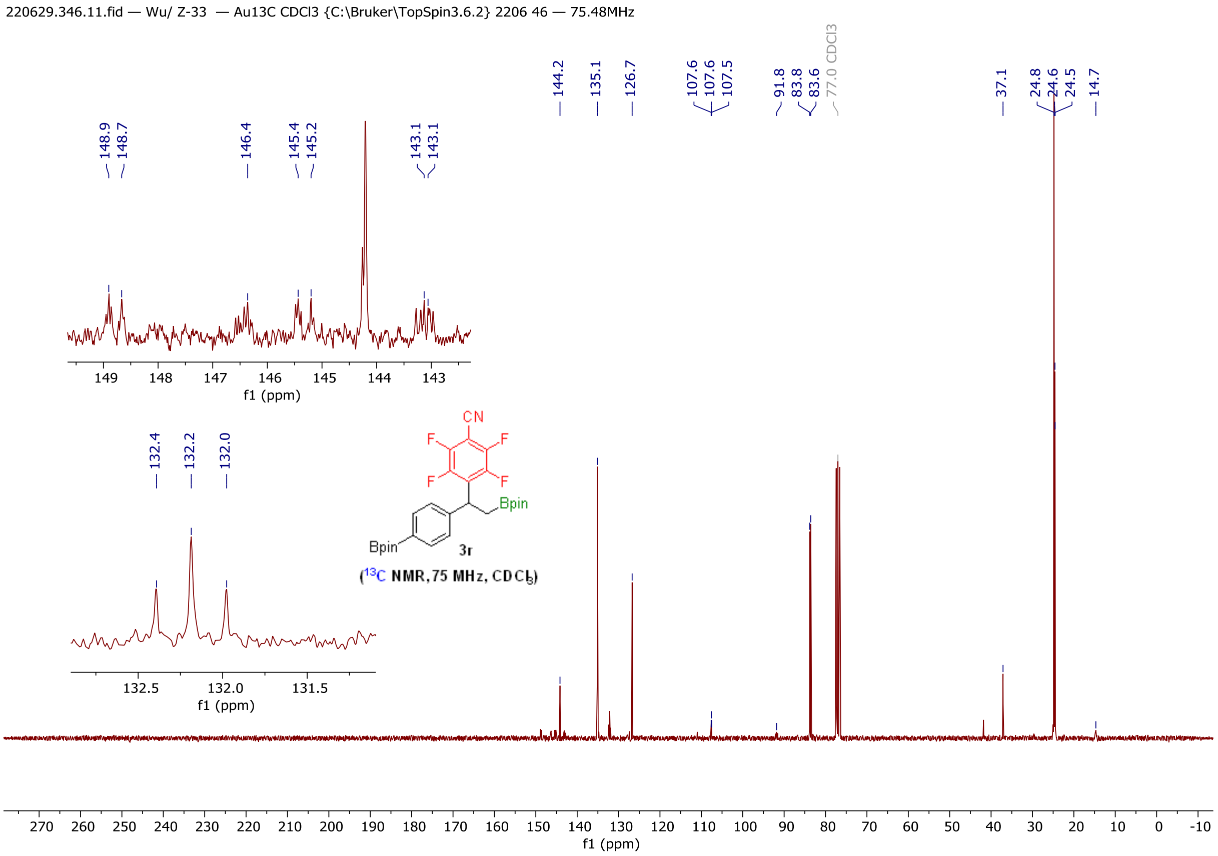

220629.346.12.fid — Wu/ Z-33 — Au19F CDCl3 {C:\Bruker\TopSpin3.6.2} 2206 46 — 282.39MHz

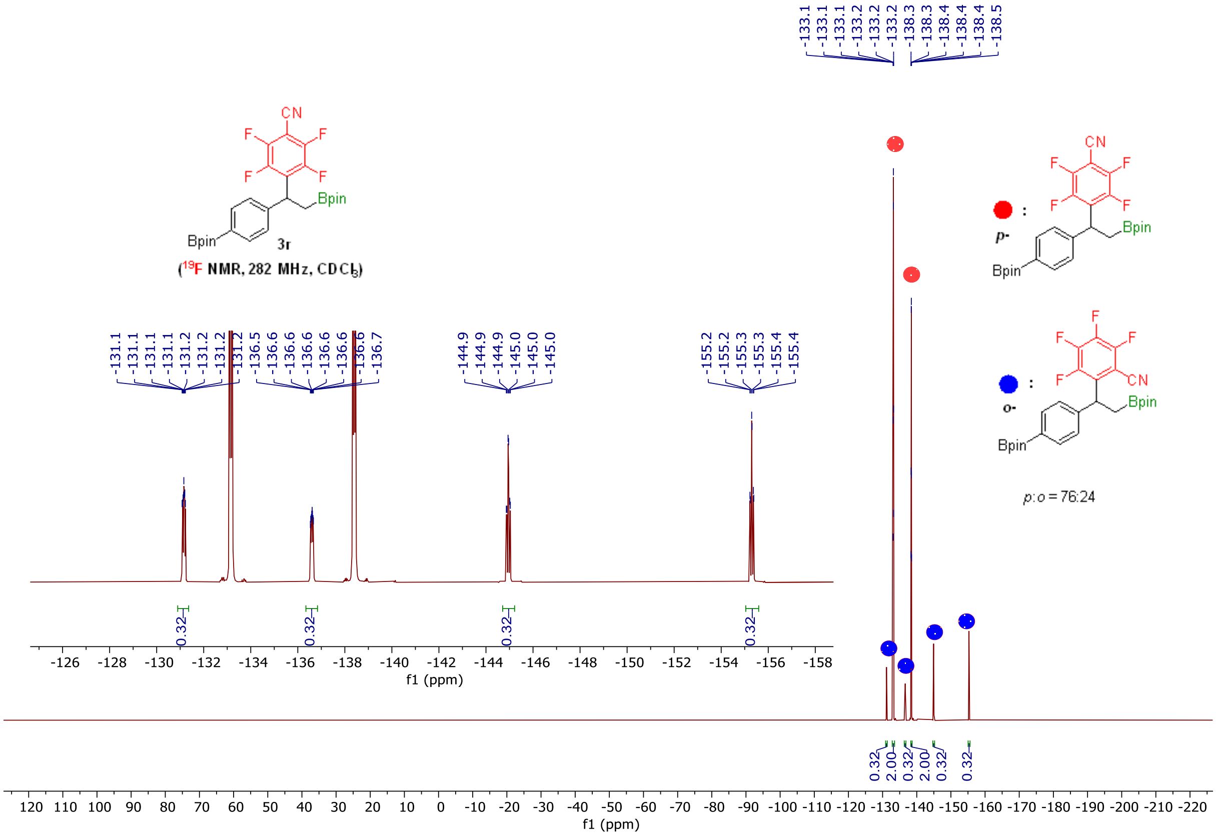

220629.346.13.fid — Wu/ Z-33 — Au11B CDCl3 {C:\Bruker\TopSpin3.6.2} 2206 46 — 96.29MHz

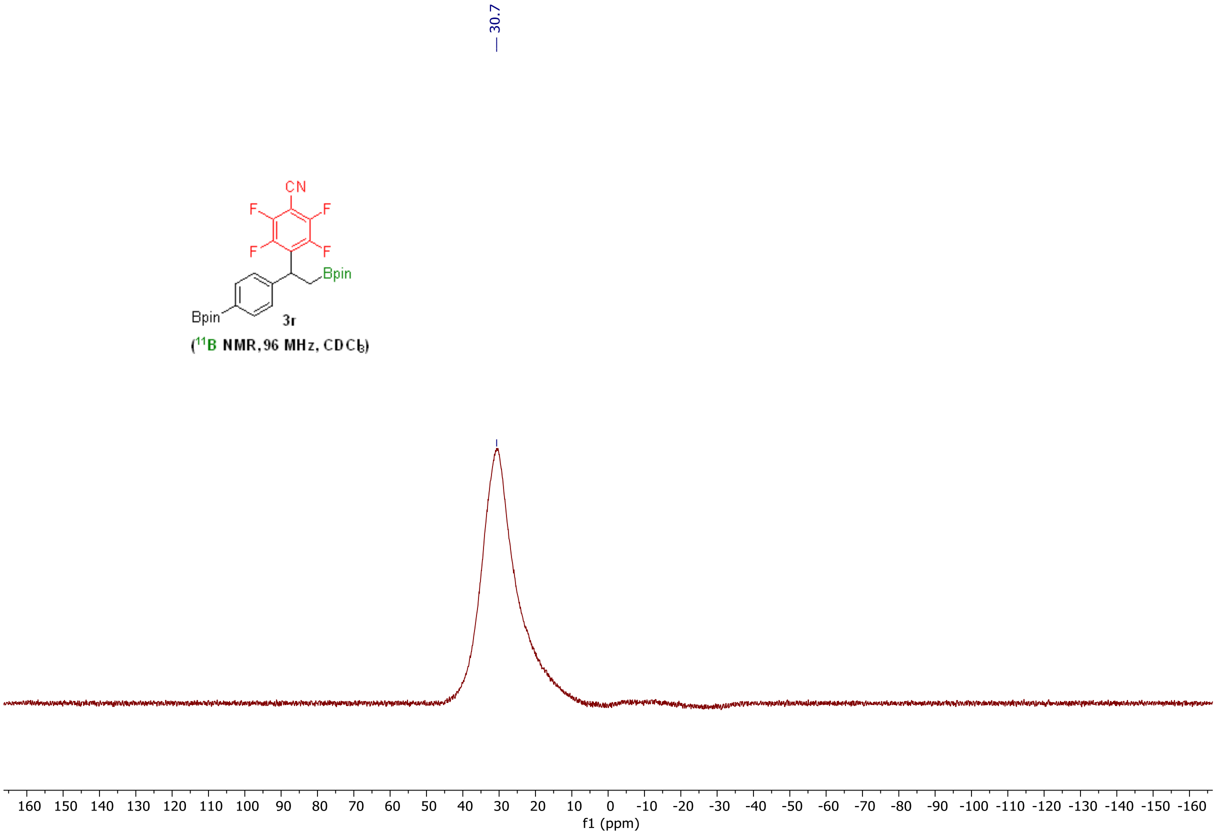

NMR Spectra of **3s**

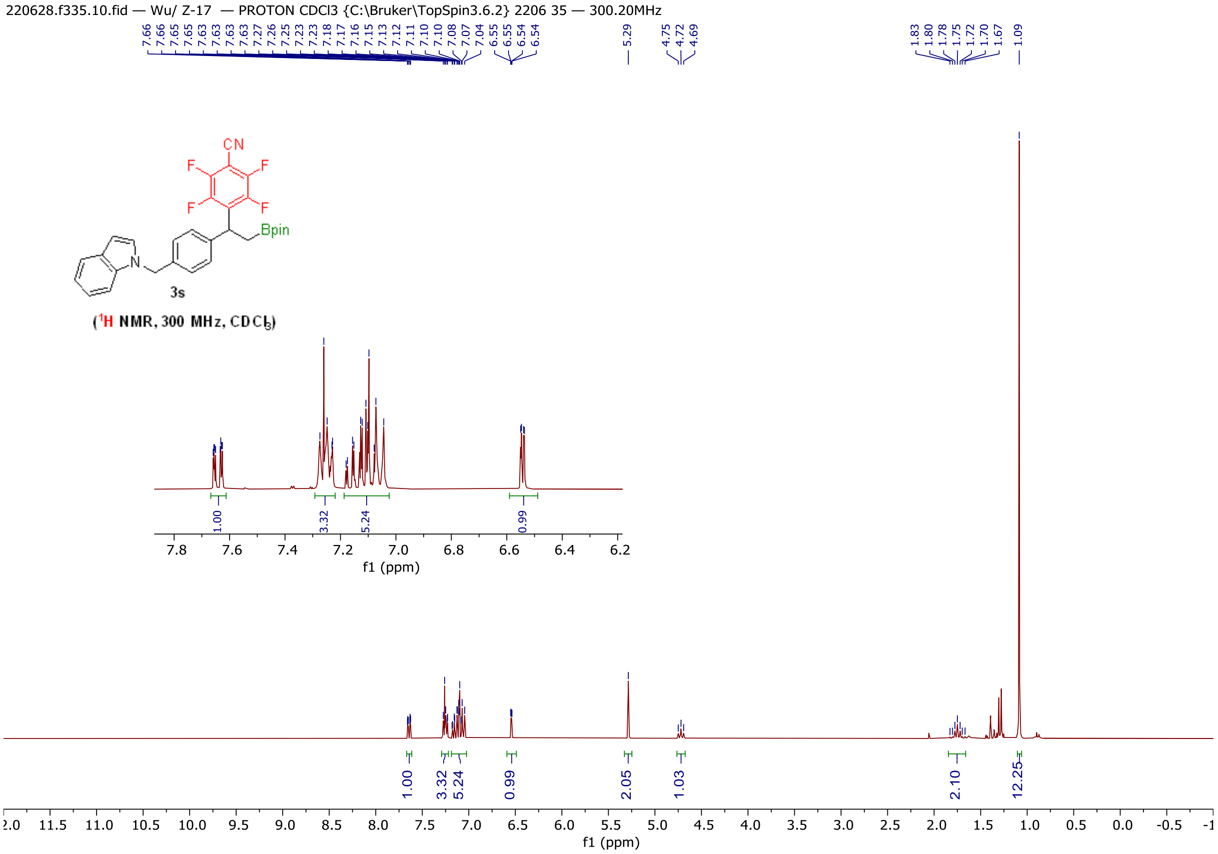

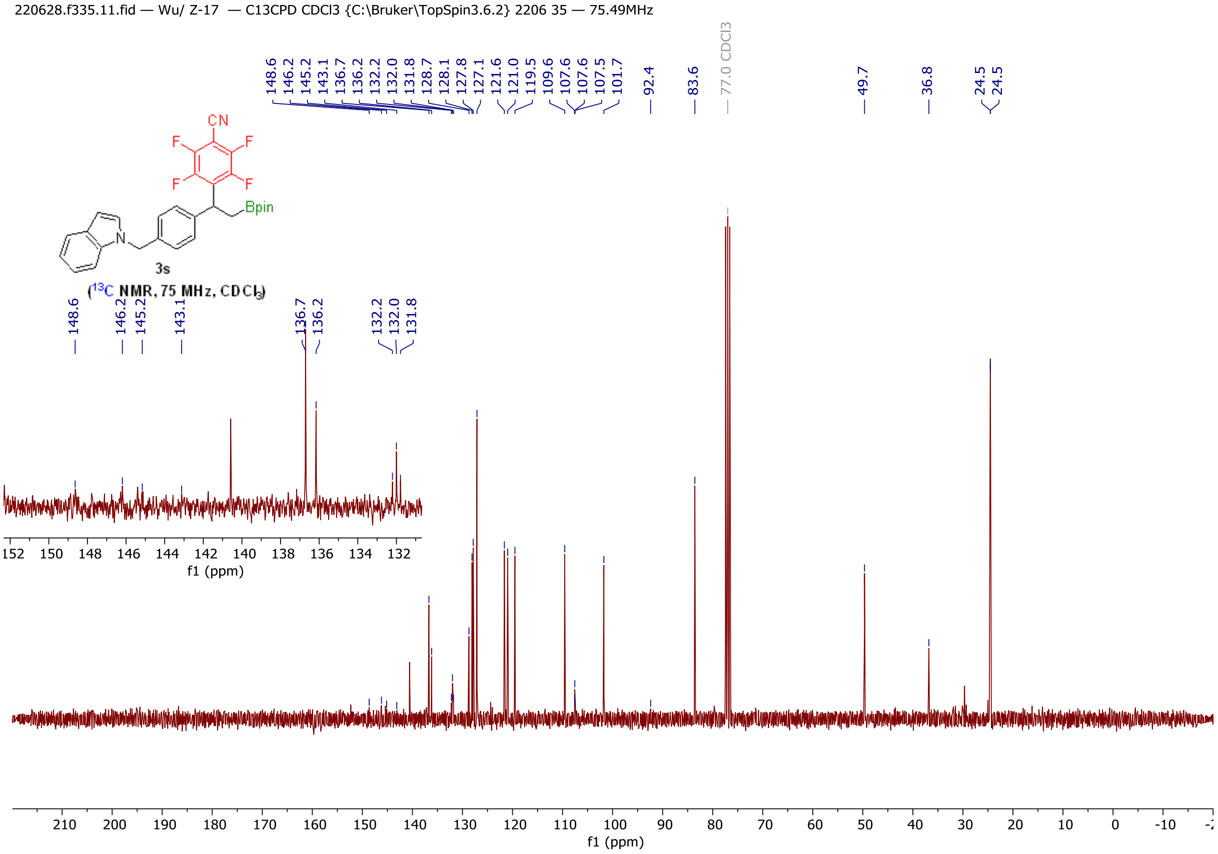

220628.f335.12.fid — Wu/ Z-17 — F19 CDCl3 {C:\Bruker\TopSpin3.6.2} 2206 35 — 282.44MHz

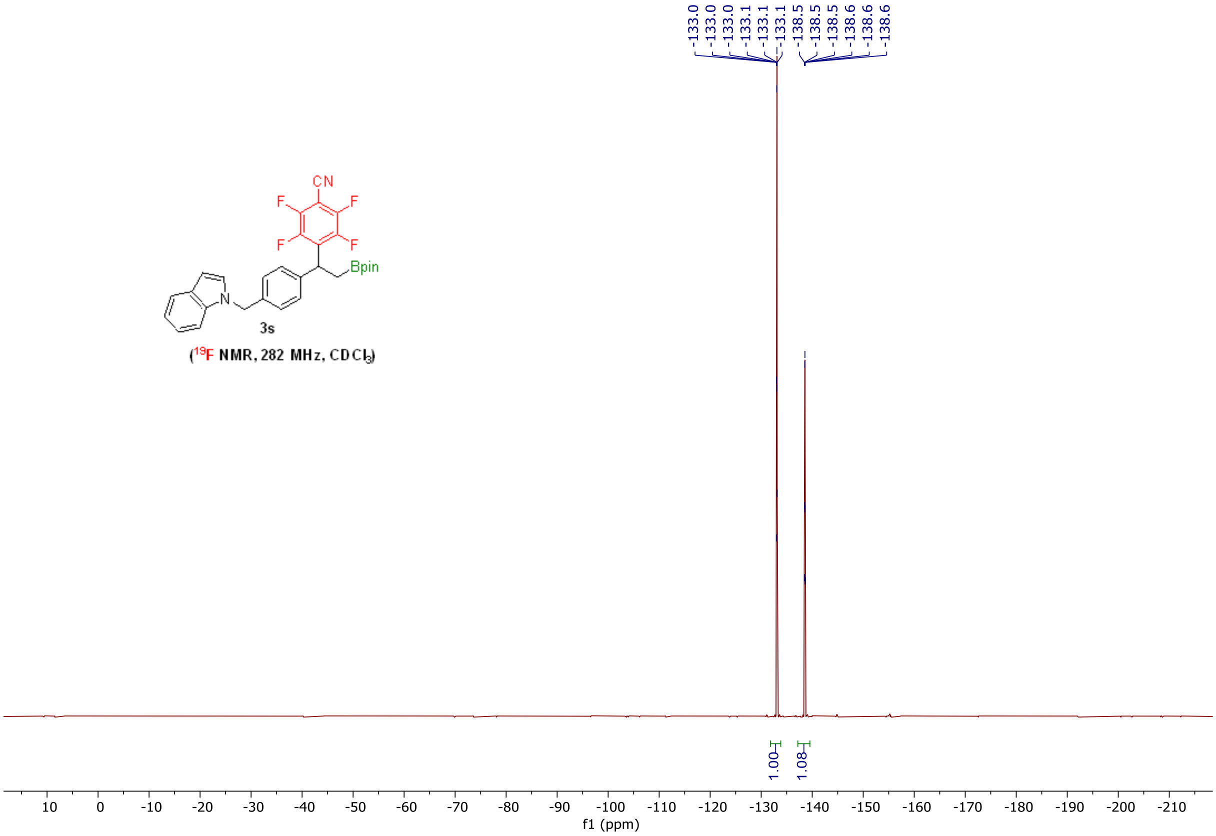

220628.f335.13.fid — Wu/ Z-17 — 11B CDCl3 {C:\Bruker\TopSpin3.6.2} 2206 35 — 96.32MHz

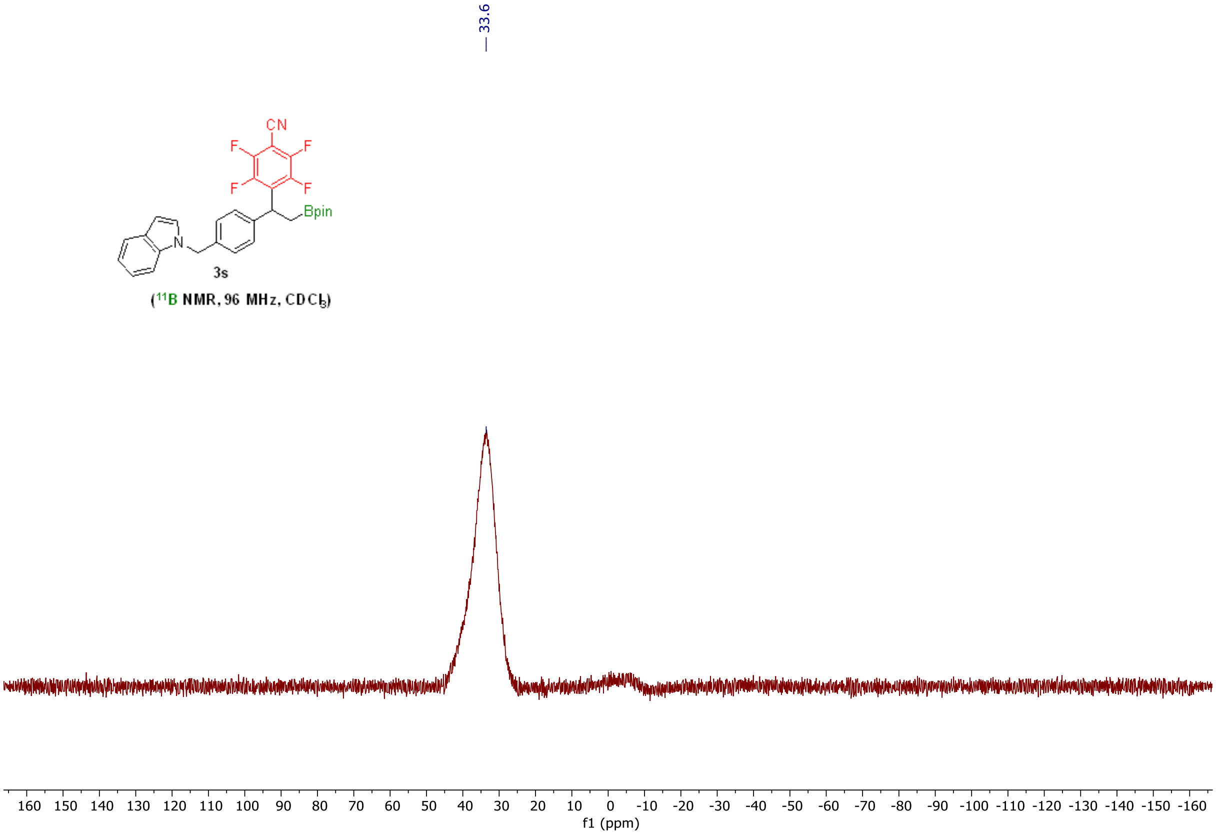

NMR Spectra of **3t**

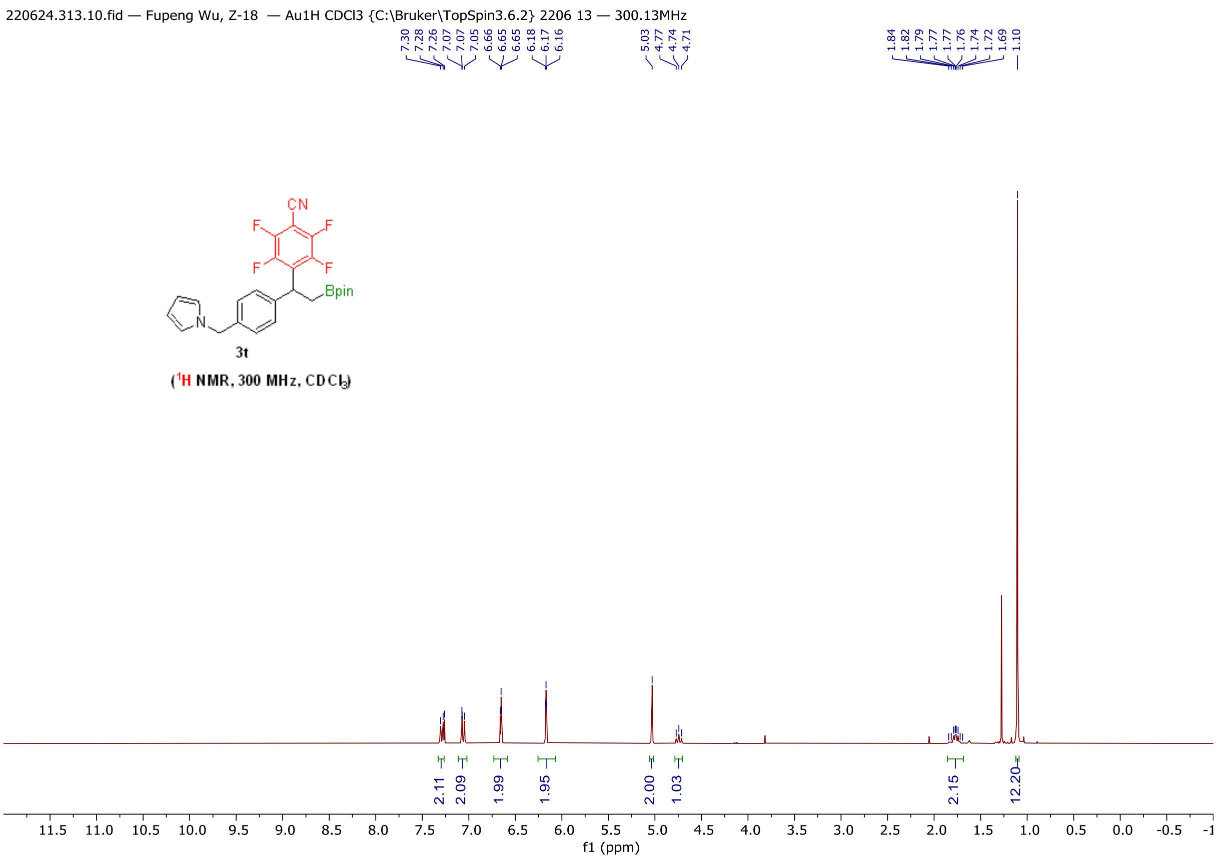

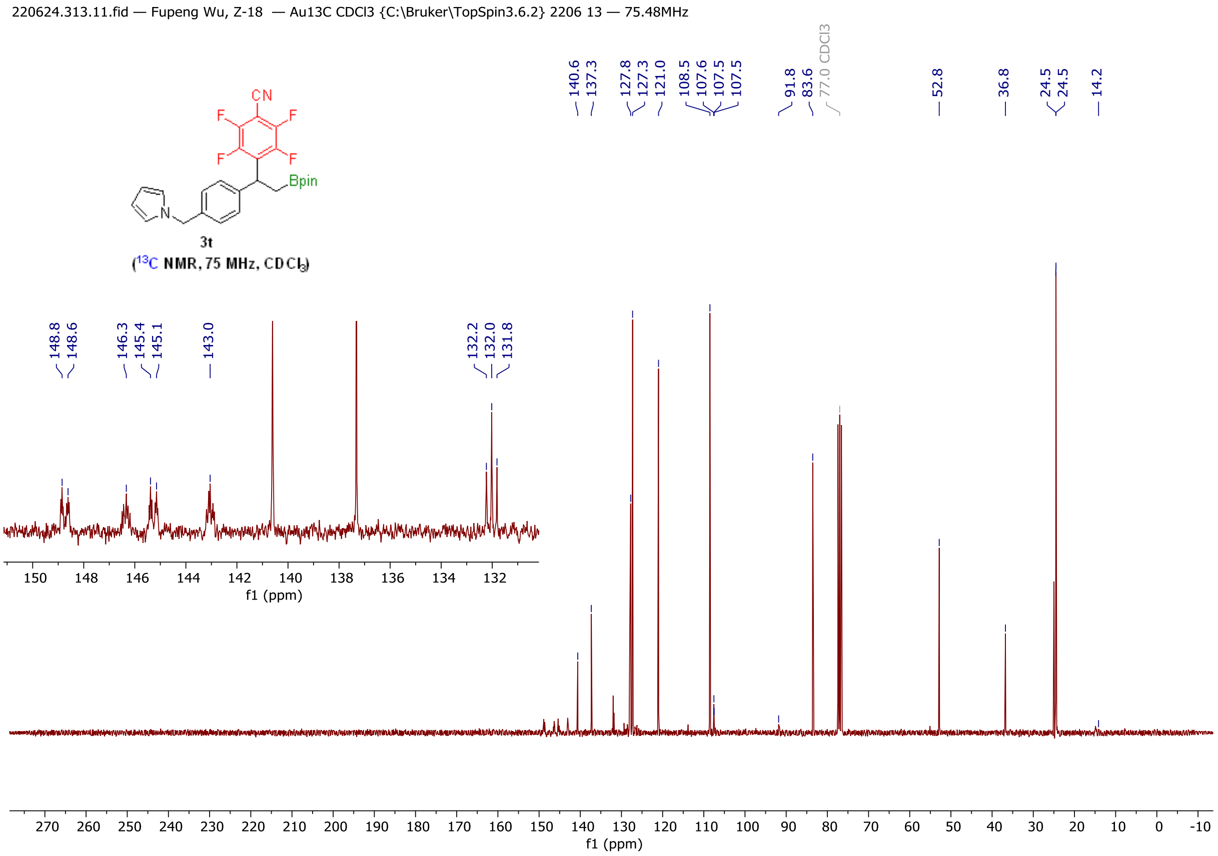

220624.313.12.fid — Fupeng Wu, Z-18 — Au19F CDCl3 {C:\Bruker\TopSpin3.6.2} 2206 13 — 282.39MHz

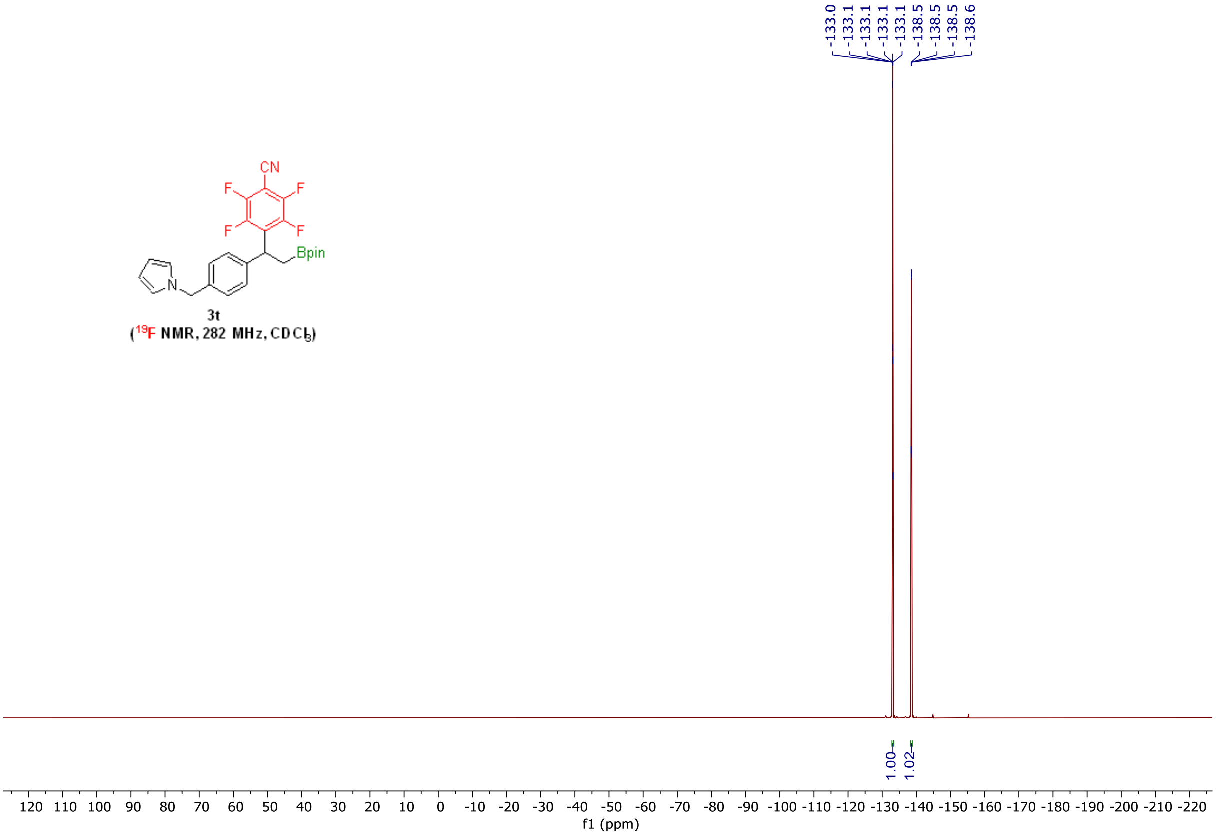

220624.313.13.fid — Fupeng Wu, Z-18 — Au11B CDCl3 {C:\Bruker\TopSpin3.6.2} 2206 13 — 96.29MHz

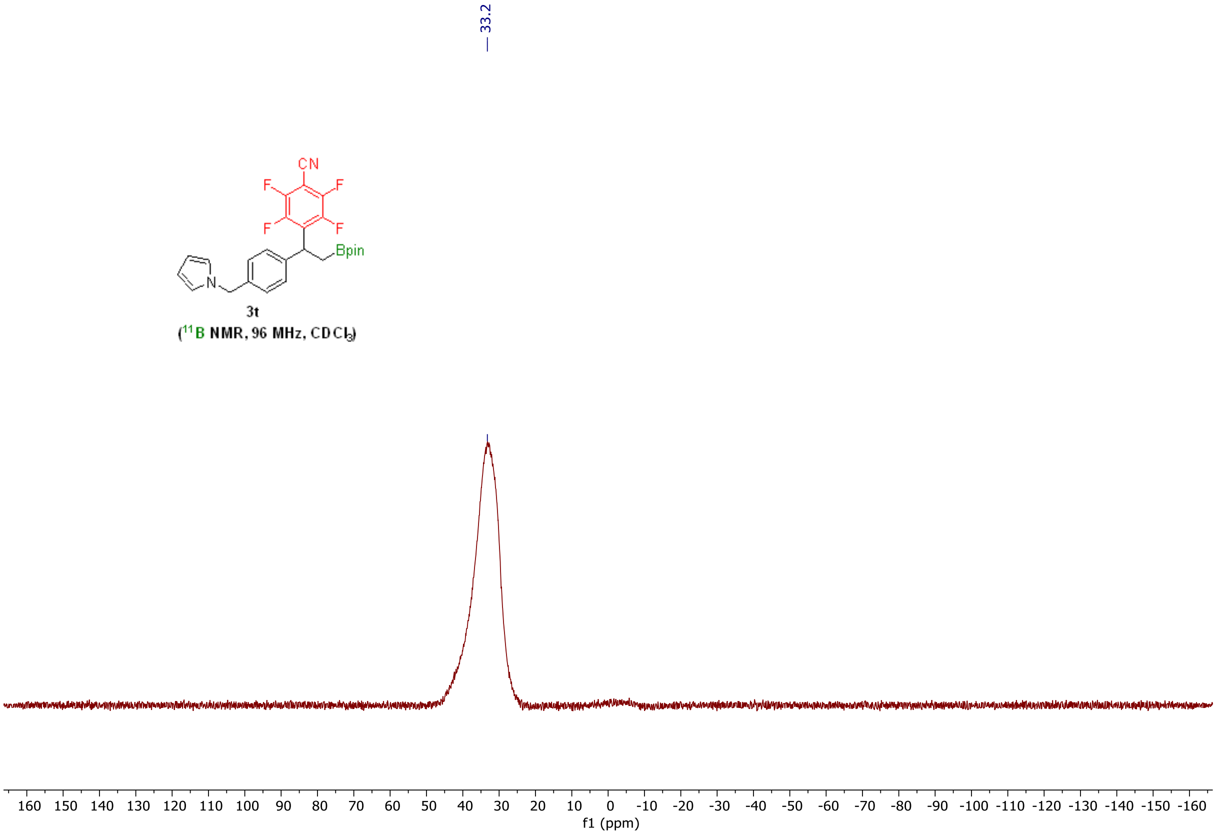

NMR Spectra of **3u**

220628.f337.10.fid — Wu/ Z-10 — PROTON CDCl3 {C:\Bruker\TopSpin3.6.2} 2206 37 — 300.20MHz

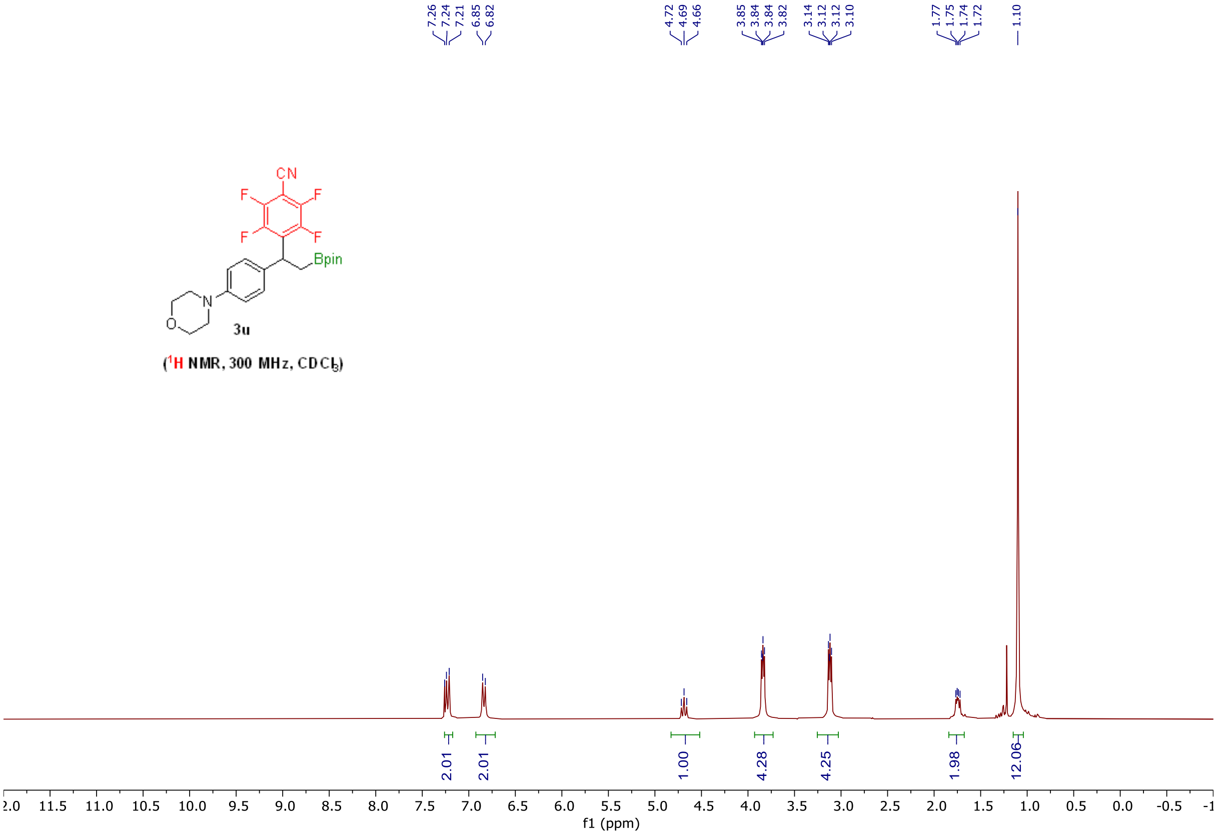

220628.f337.11.fid — Wu/ Z-10 — C13CPD CDCl3 {C:\Bruker\TopSpin3.6.2} 2206 37 — 75.49MHz

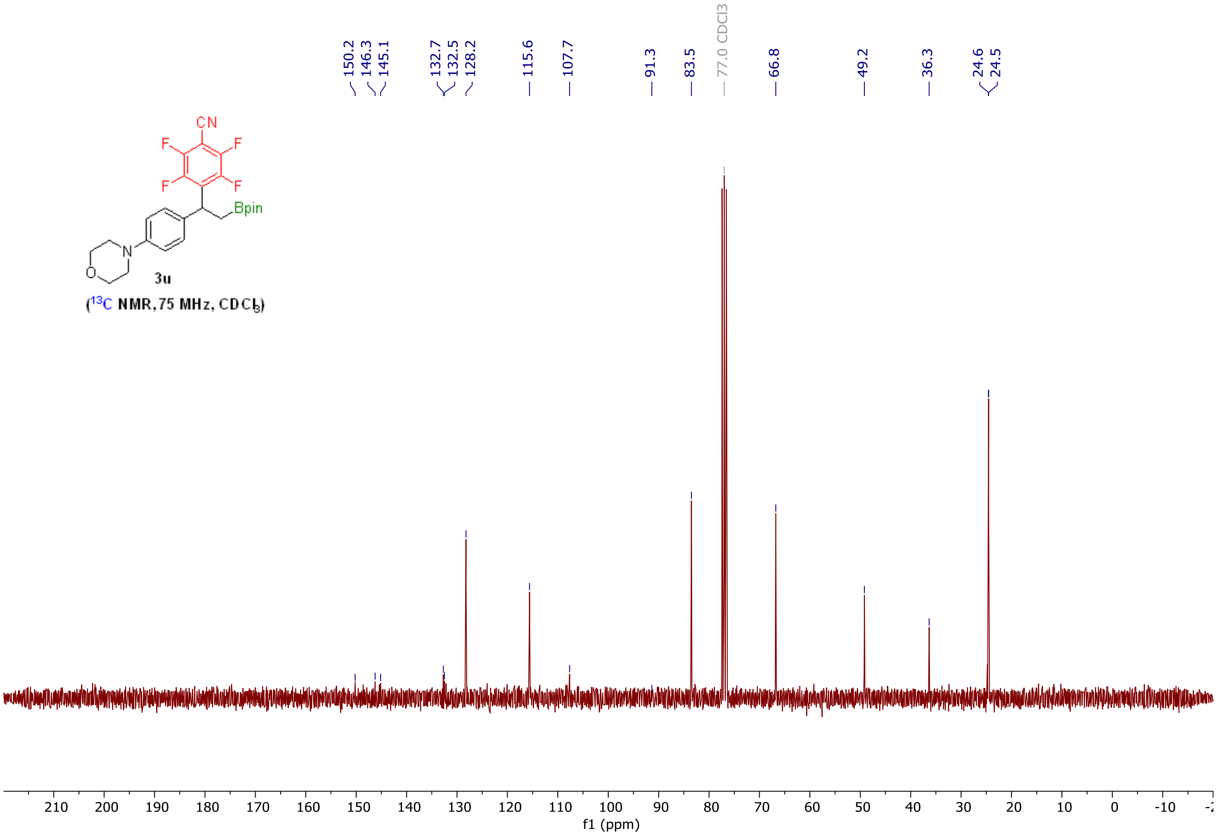

220621.363.12.fid — Fupeng Wu Z-10 — Au19F CDCl3 {C:\Bruker\TopSpin3.6.2} 2206 3 — 282.39MHz

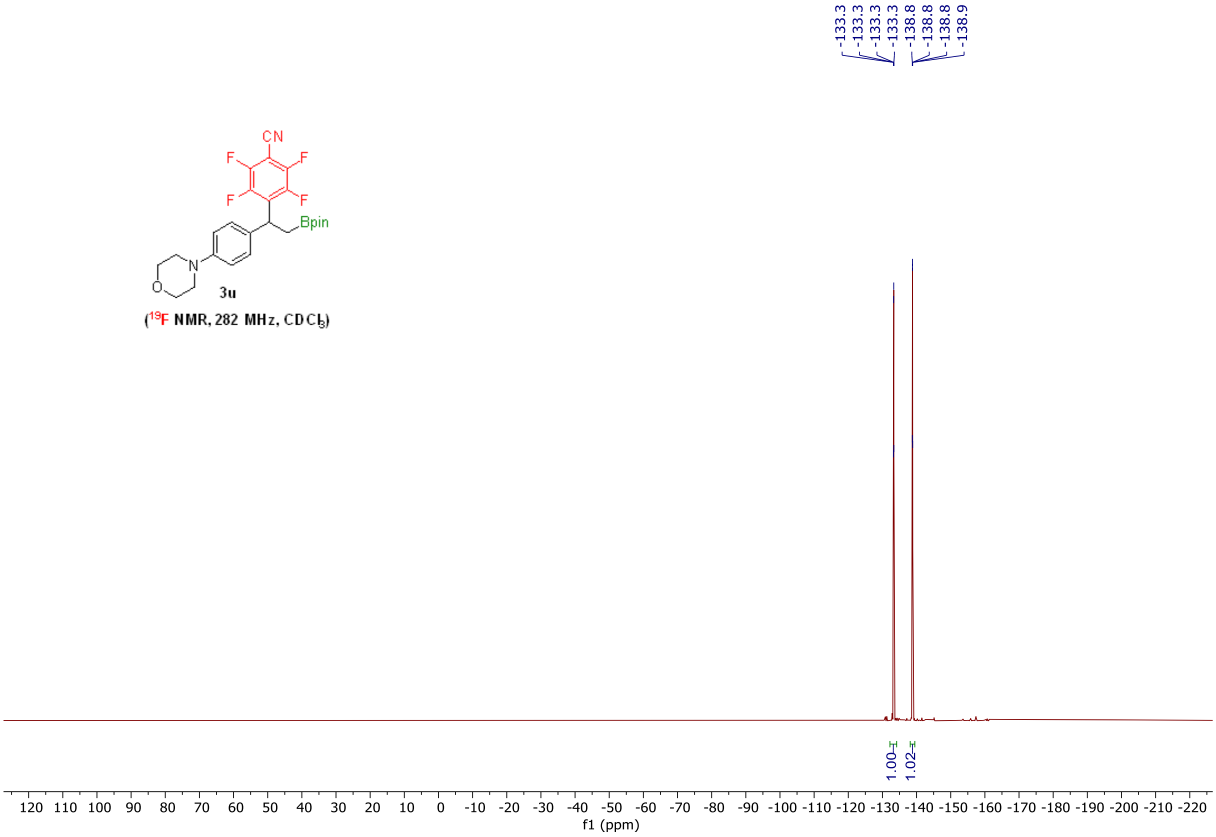

220628.f337.13.fid — Wu/ Z-10 — 11B CDCl3 {C:\Bruker\TopSpin3.6.2} 2206 37 — 96.32MHz

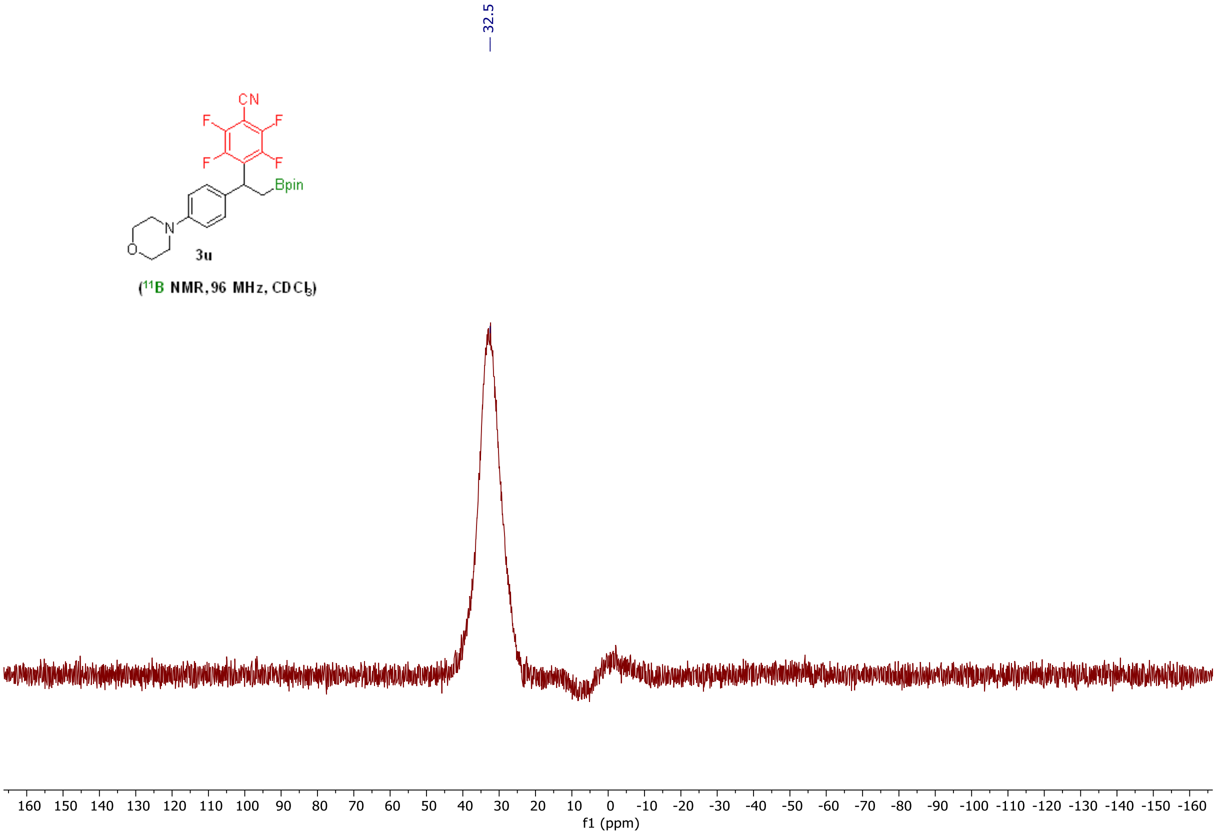

NMR Spectra of **3v**

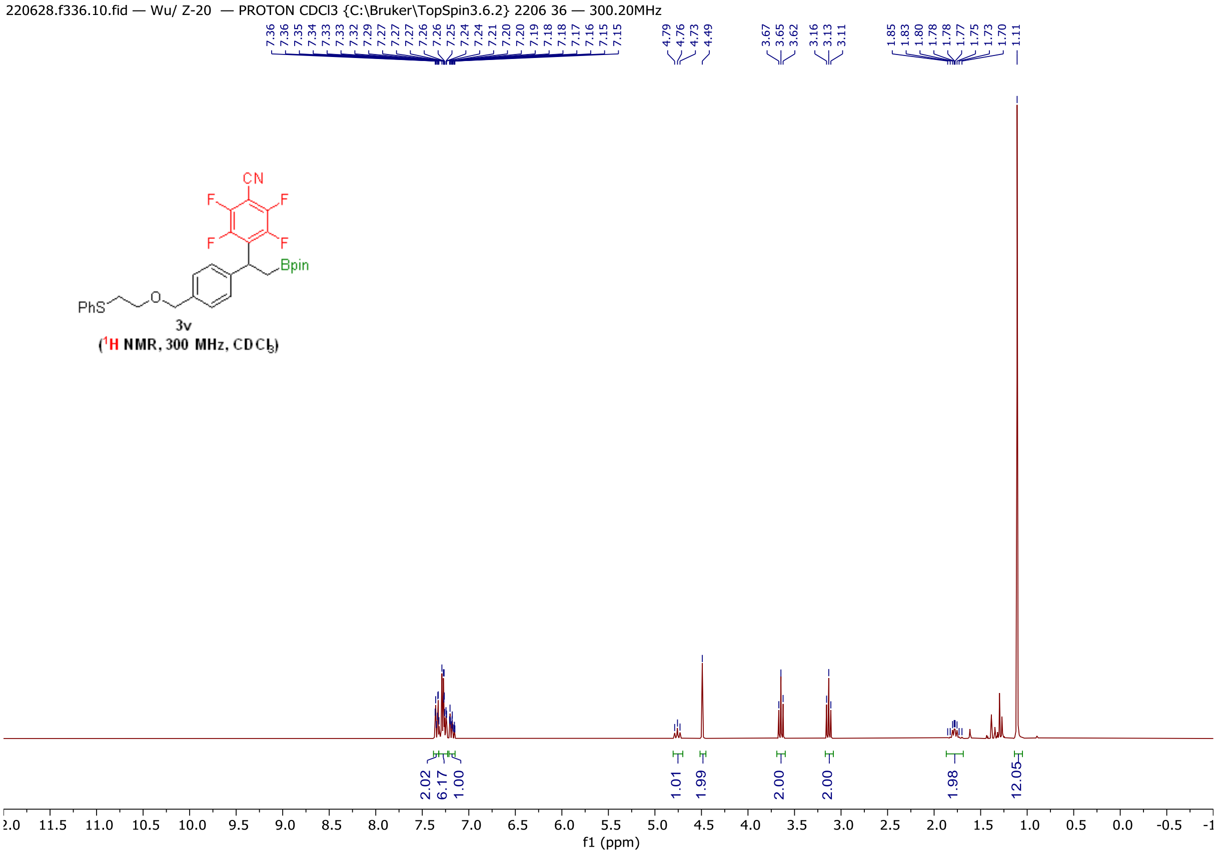

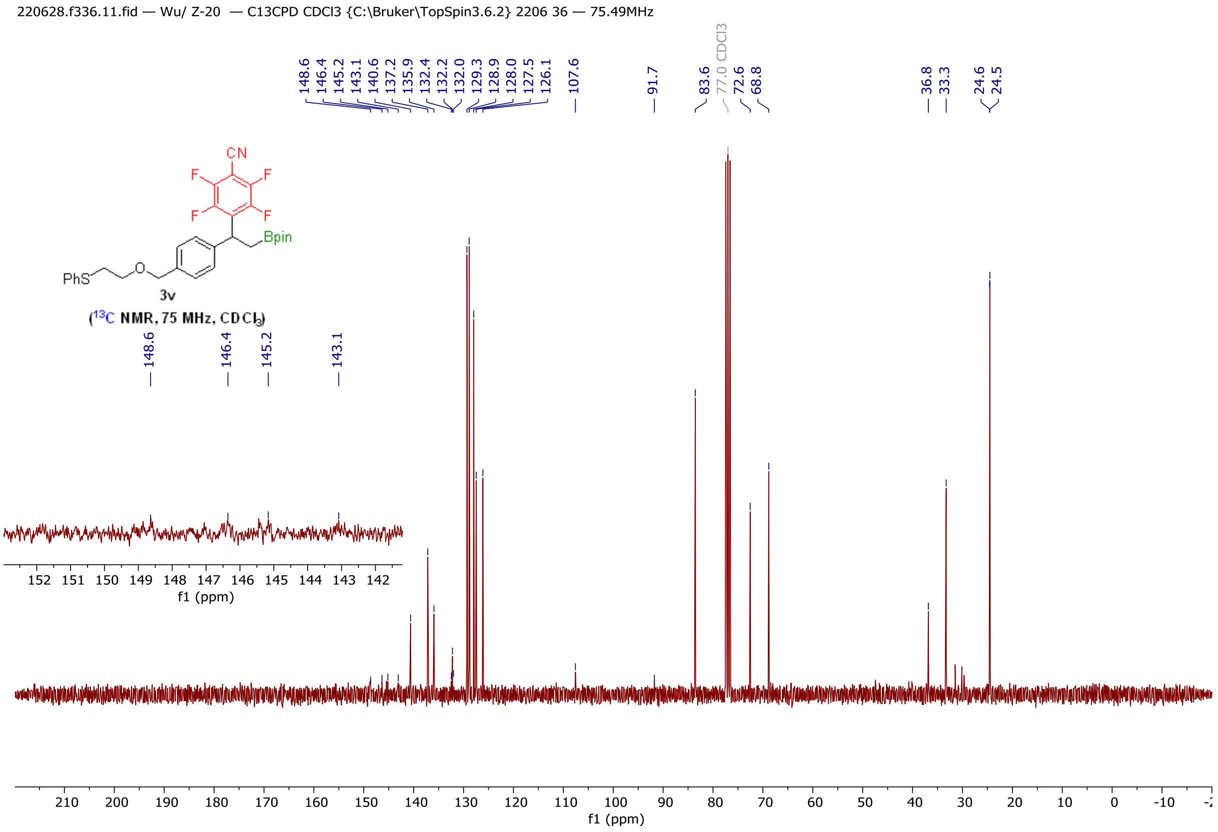

220628.f336.12.fid — Wu/ Z-20 — F19 CDCl3 {C:\Bruker\TopSpin3.6.2} 2206 36 — 282.44MHz

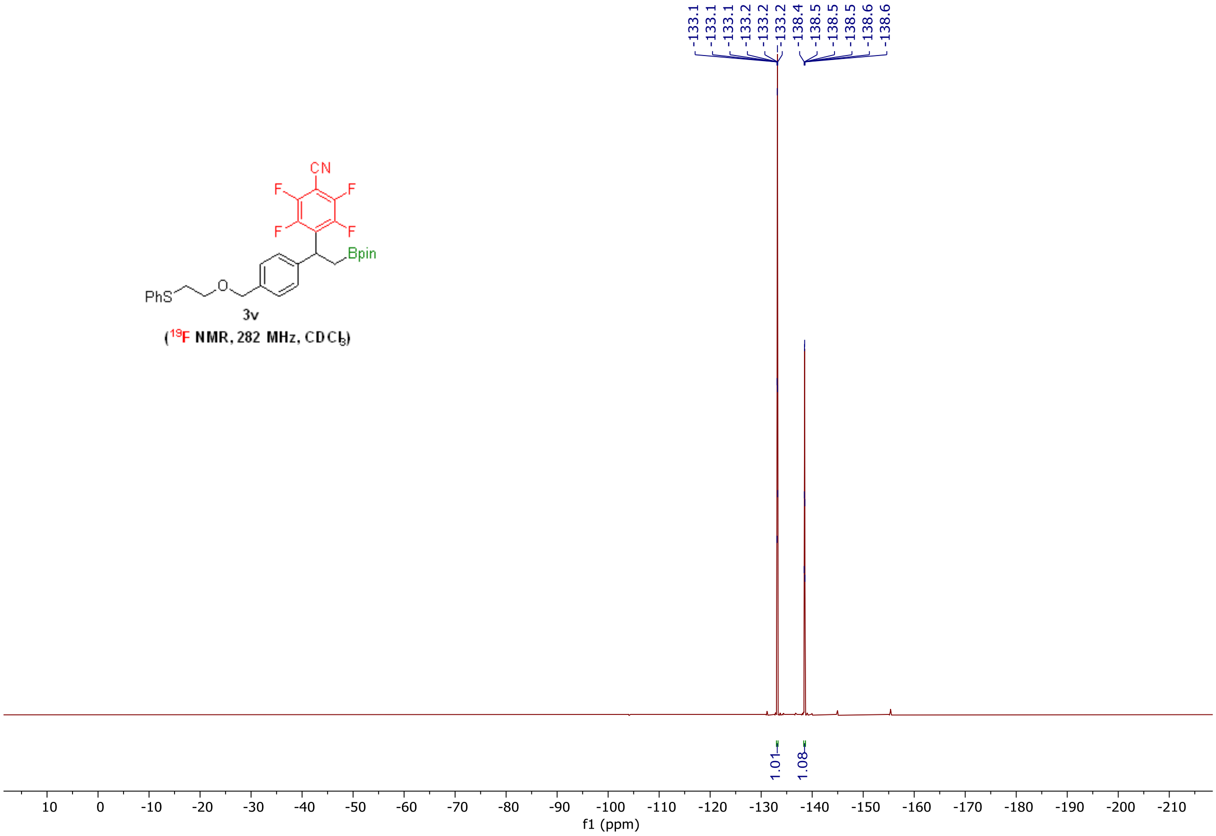

220628.f336.13.fid — Wu/ Z-20 — 11B CDCl3 {C:\Bruker\TopSpin3.6.2} 2206 36 — 96.32MHz

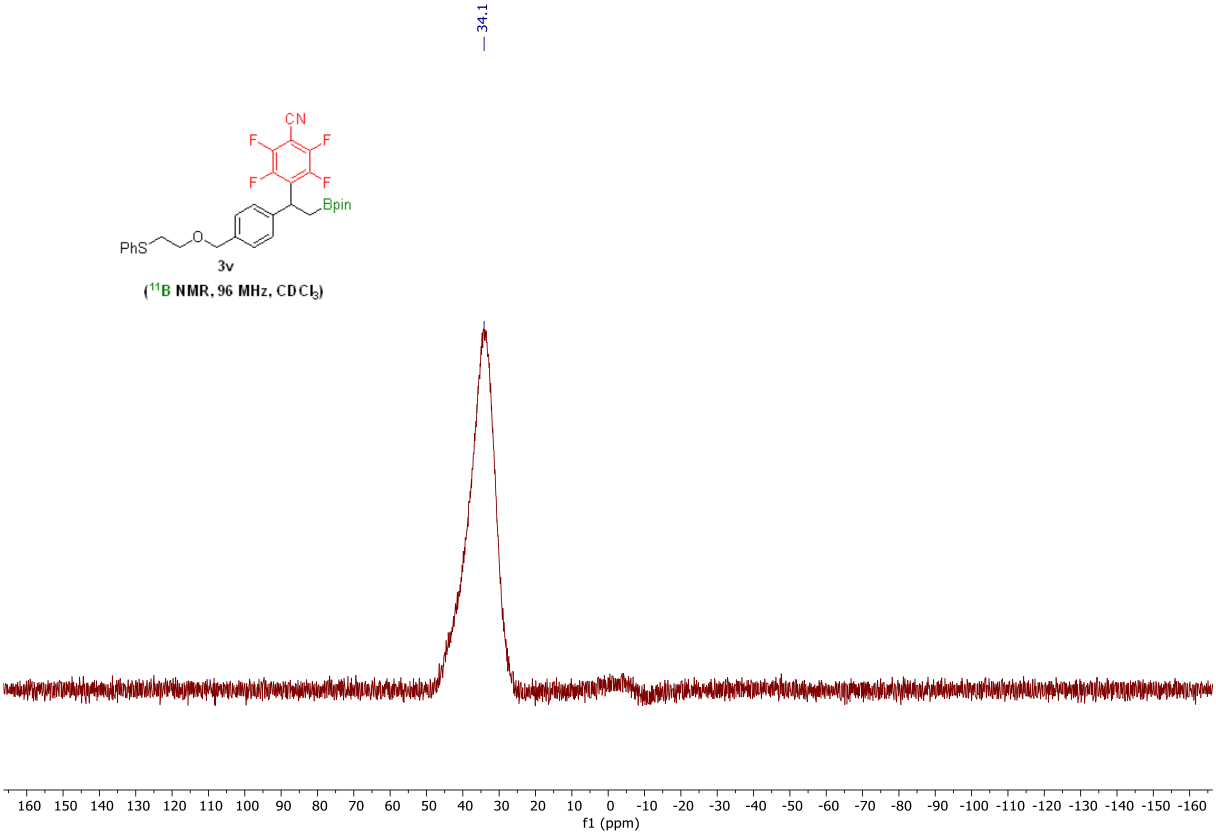

NMR Spectra of **3w**

220623.317.10.fid — Wu/ Z-14-1 — Au1H CDCl3 {C:\Bruker\TopSpin3.6.2} 2206 17 — 300.13MHz

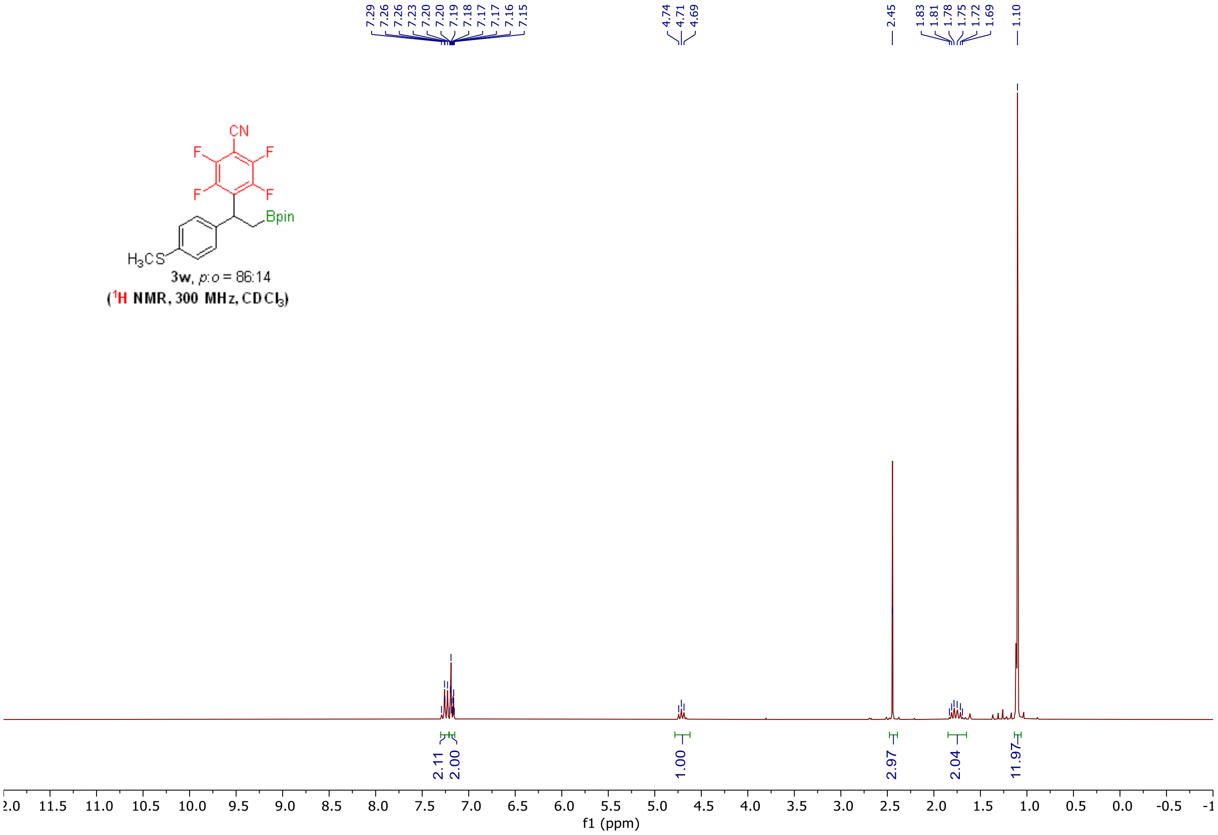

220624.312.11.fid — Fupeng Wu, Z-14 — Au13C CDCl3 {C:\Bruker\TopSpin3.6.2} 2206 12 — 75.48MHz

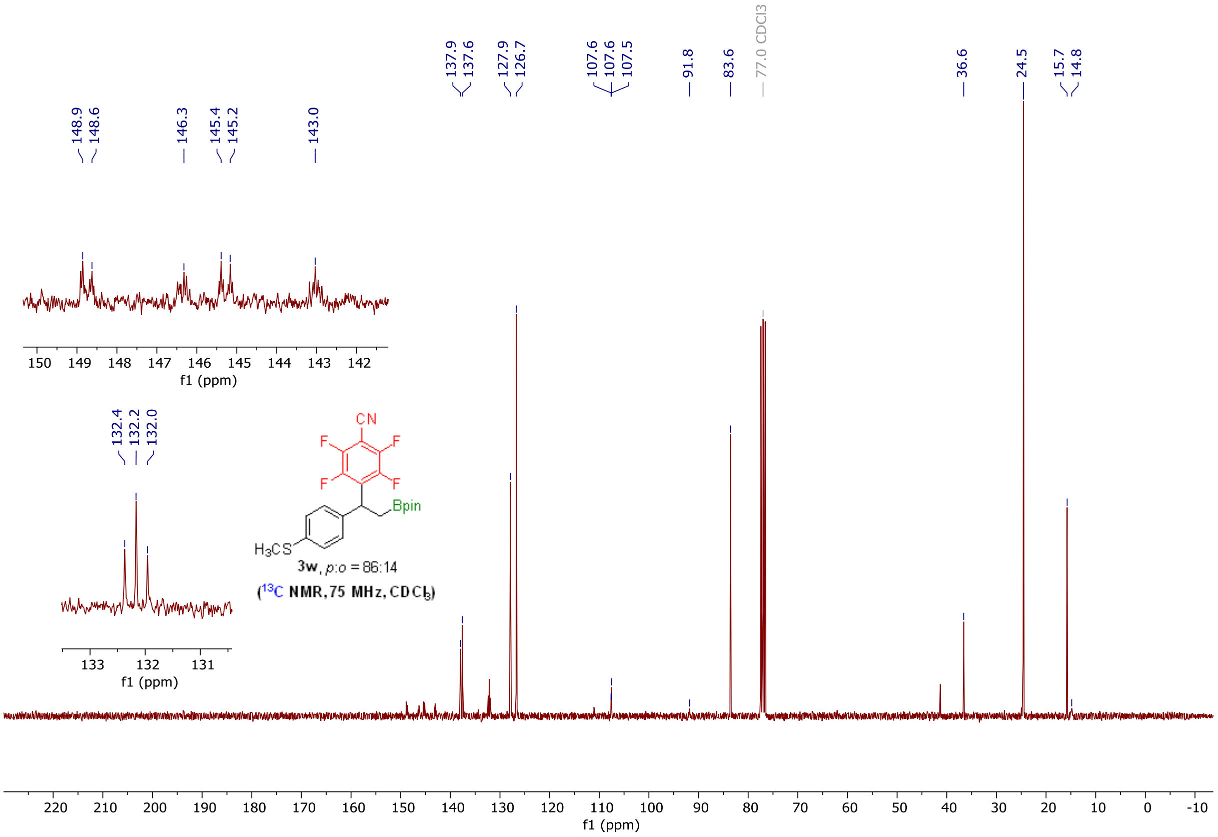

220624.312.12.fid — Fupeng Wu, Z-14 — Au19F CDCl3 {C:\Bruker\TopSpin3.6.2} 2206 12 — 282.39MHz

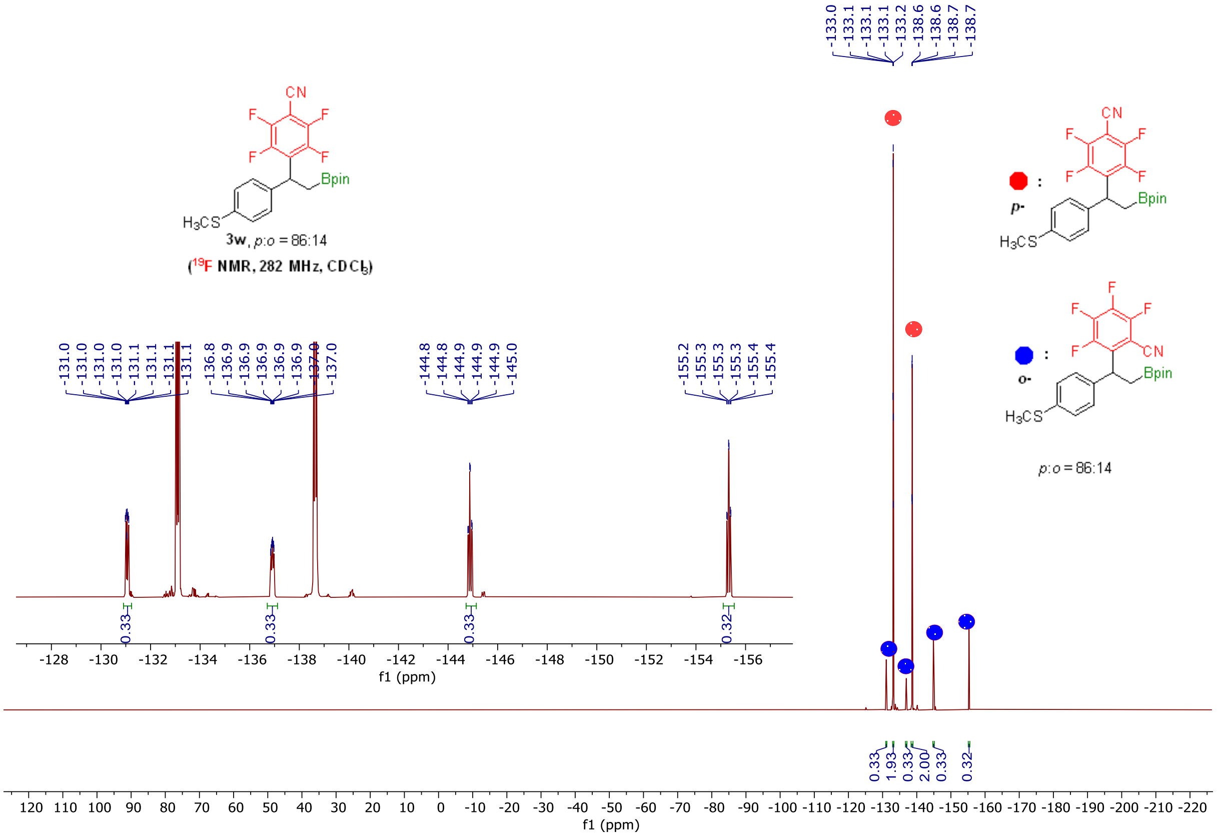

220624.312.13.fid — Fupeng Wu, Z-14 — Au11B CDCl3 {C:\Bruker\TopSpin3.6.2} 2206 12 — 96.29MHz

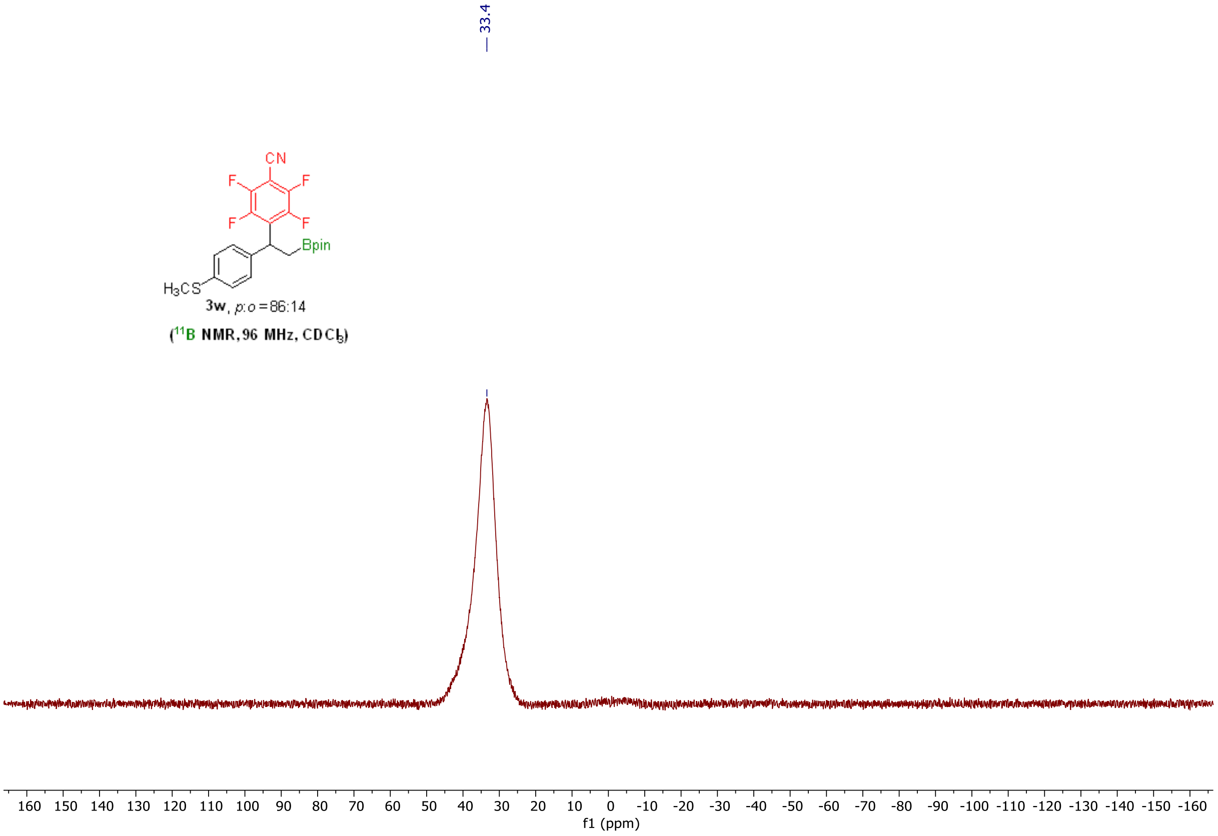

NMR Spectra of **3x**

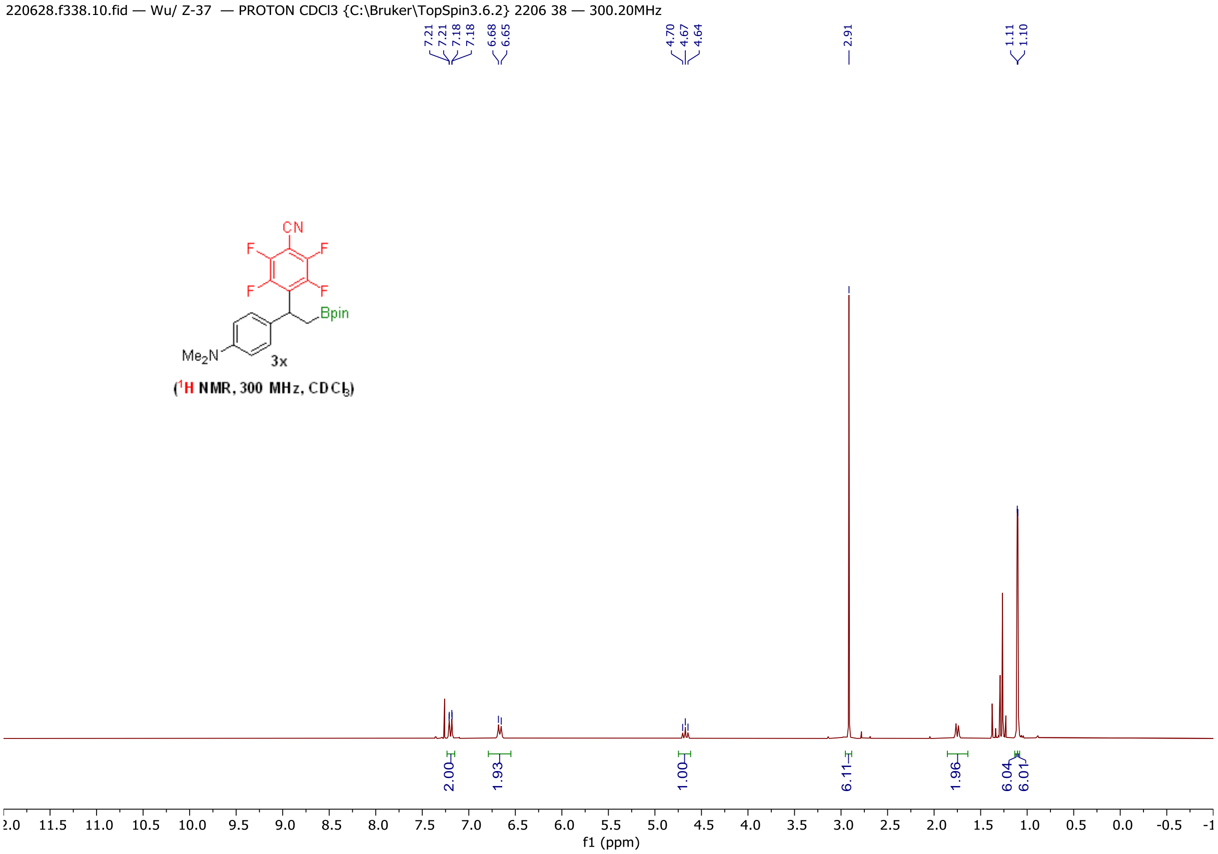

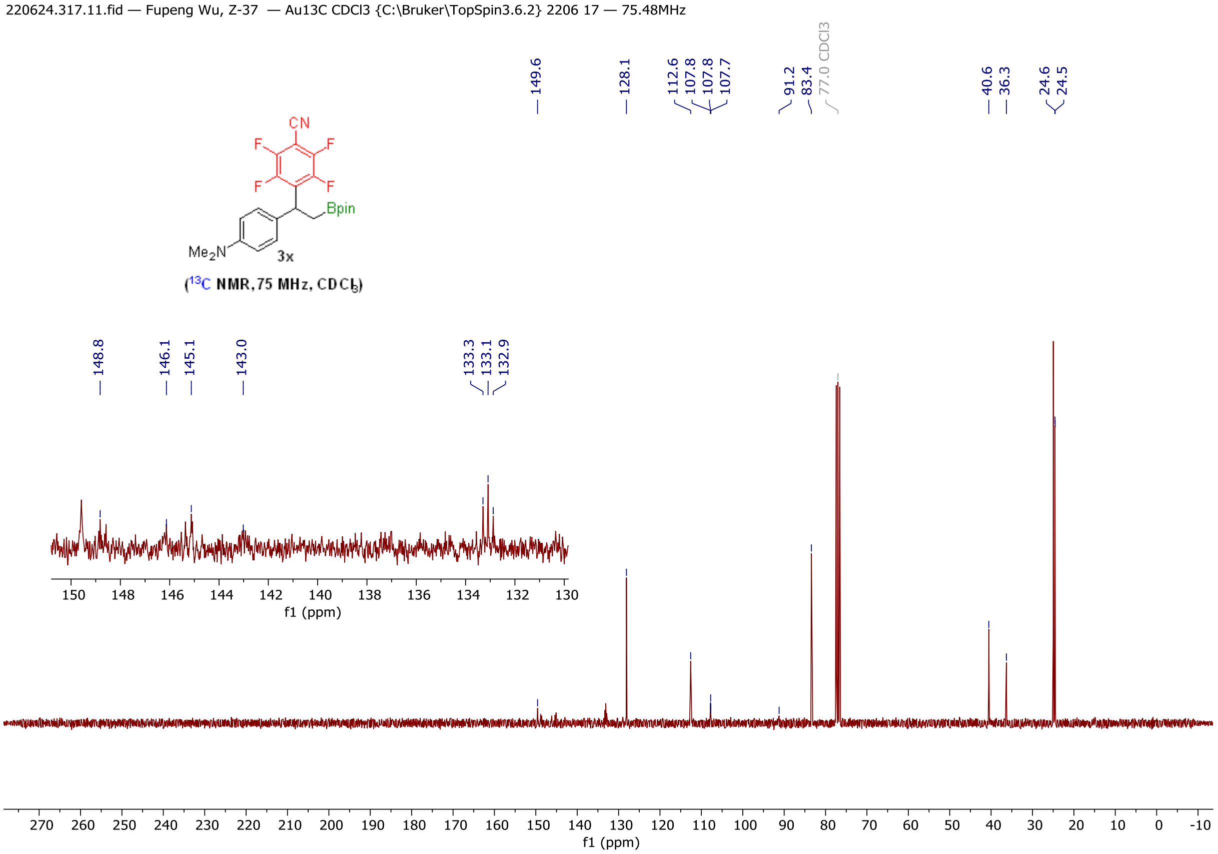

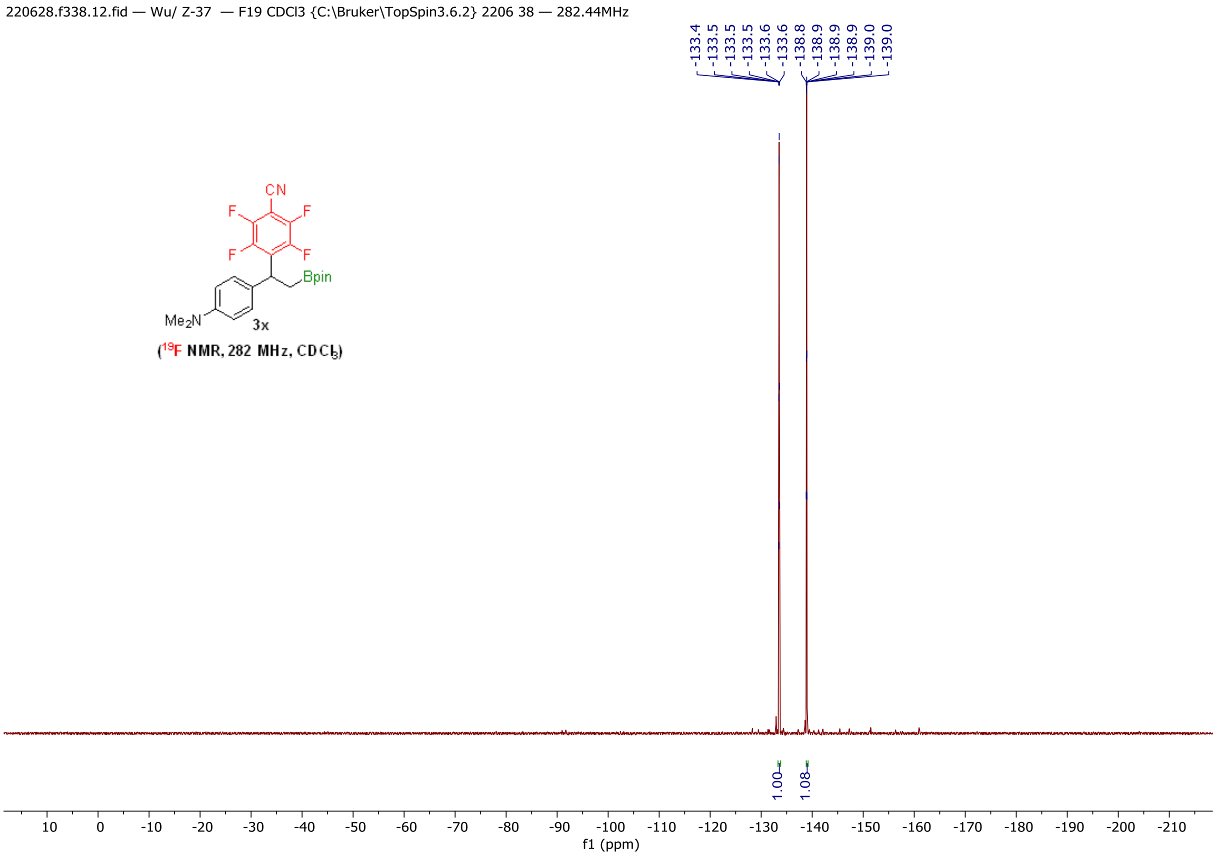
220628.f338.12.fid — Wu/ Z-37 — F19 CDCl3 {C:\Bruker\TopSpin3.6.2} 2206 38 — 282.44MHz
-133.4
-133.5
-133.5
-133.5
-133.6
-133.6
-138.8
-138.9
-138.9
-138.9
-139.0
-139.0
CN
F
F
F
F
Bpin
Me2N
3x
(19F NMR, 282 MHz, CDCl3)
1.00
1.08
10 0 -10 -20 -30 -40 -50 -60 -70 -80 -90 -100 -110 -120 -130 -140 -150 -160 -170 -180 -190 -200 -210
f1 (ppm)

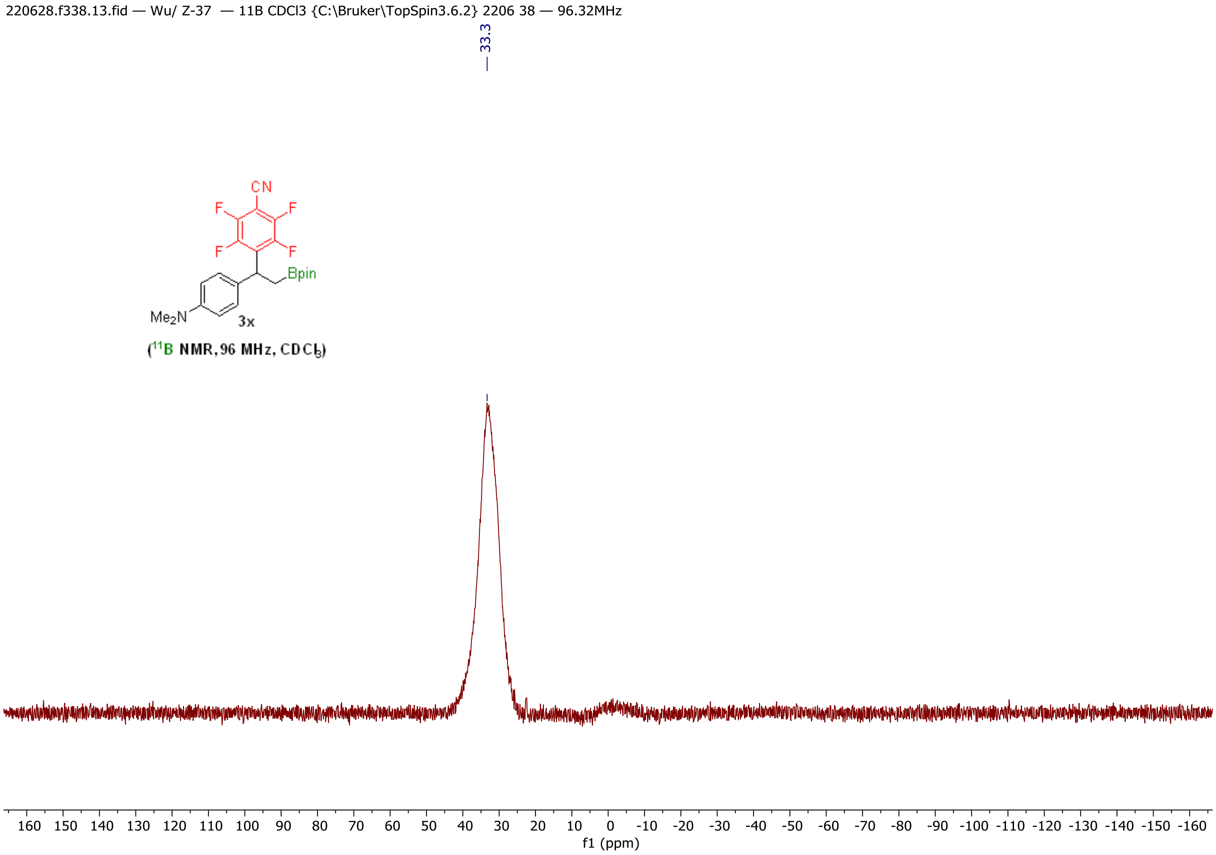
220628.f338.13.fid — Wu/ Z-37 — 11B CDCl3 {C:\Bruker\TopSpin3.6.2} 2206 38 — 96.32MHz
33.3
CN
F
F
F
F
Bpin
Me2N
3x
(11B NMR, 96 MHz, CDCl3)
160 150 140 130 120 110 100 90 80 70 60 50 40 30 20 10 0 -10 -20 -30 -40 -50 -60 -70 -80 -90 -100 -110 -120 -130 -140 -150 -160
f1 (ppm)

NMR Spectra of **3y**

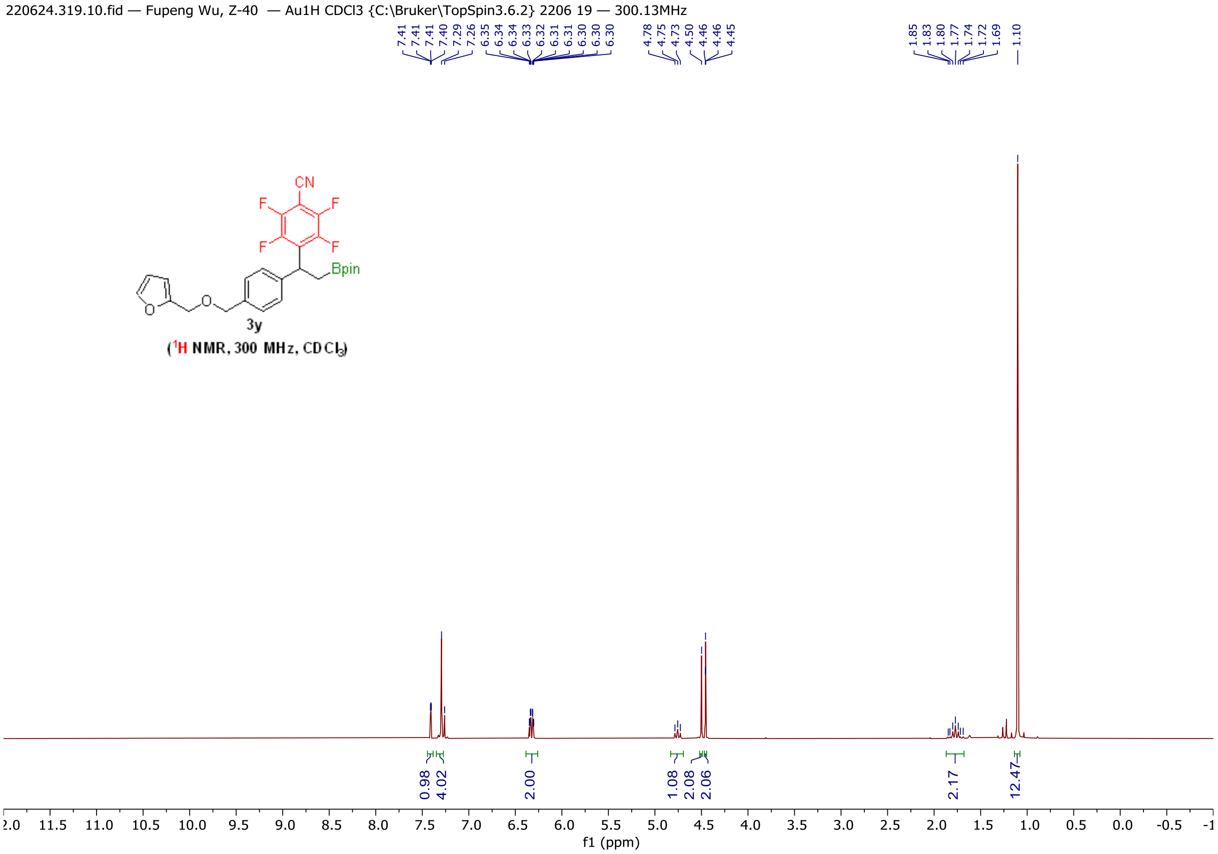

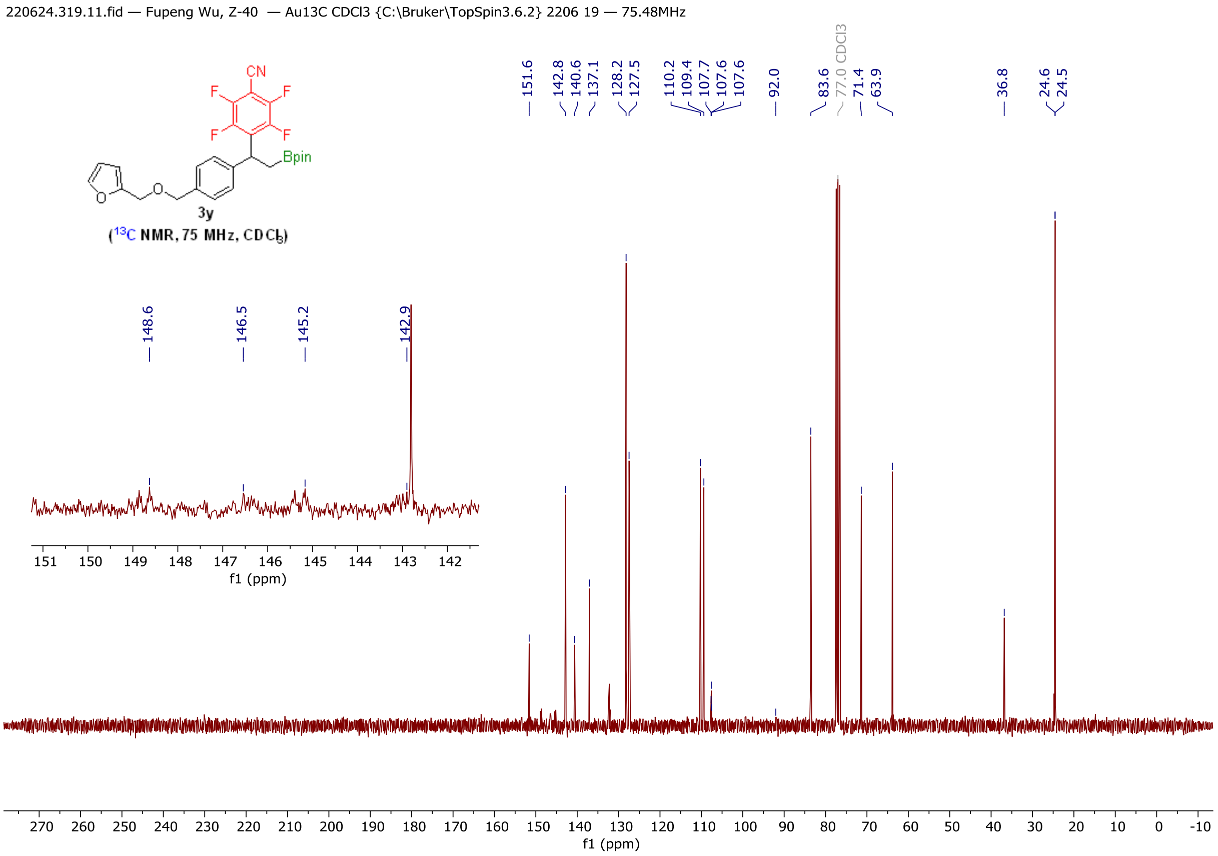

220624.319.12.fid — Fupeng Wu, Z-40 — Au19F CDCl3 {C:\Bruker\TopSpin3.6.2} 2206 19 — 282.39MHz

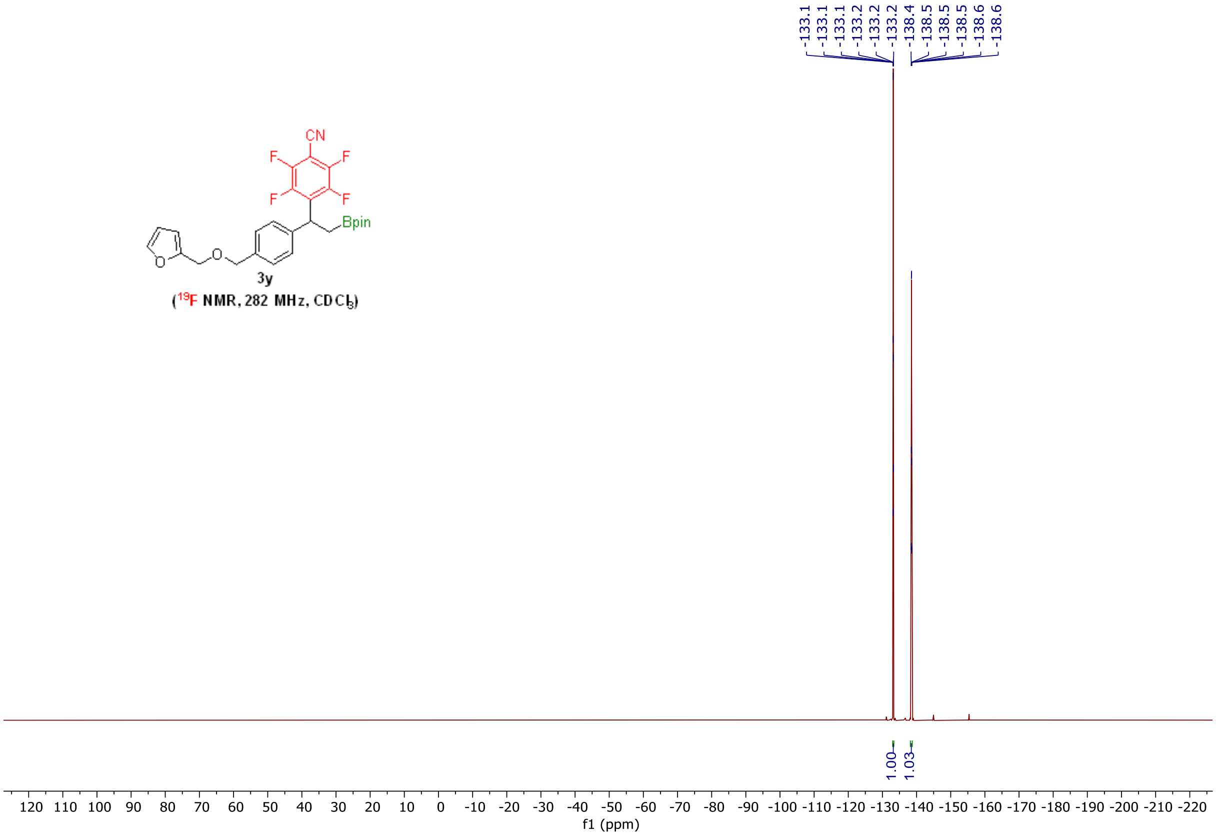

220624.319.13.fid — Fupeng Wu, Z-40 — Au11B CDCl3 {C:\Bruker\TopSpin3.6.2} 2206 19 — 96.29MHz

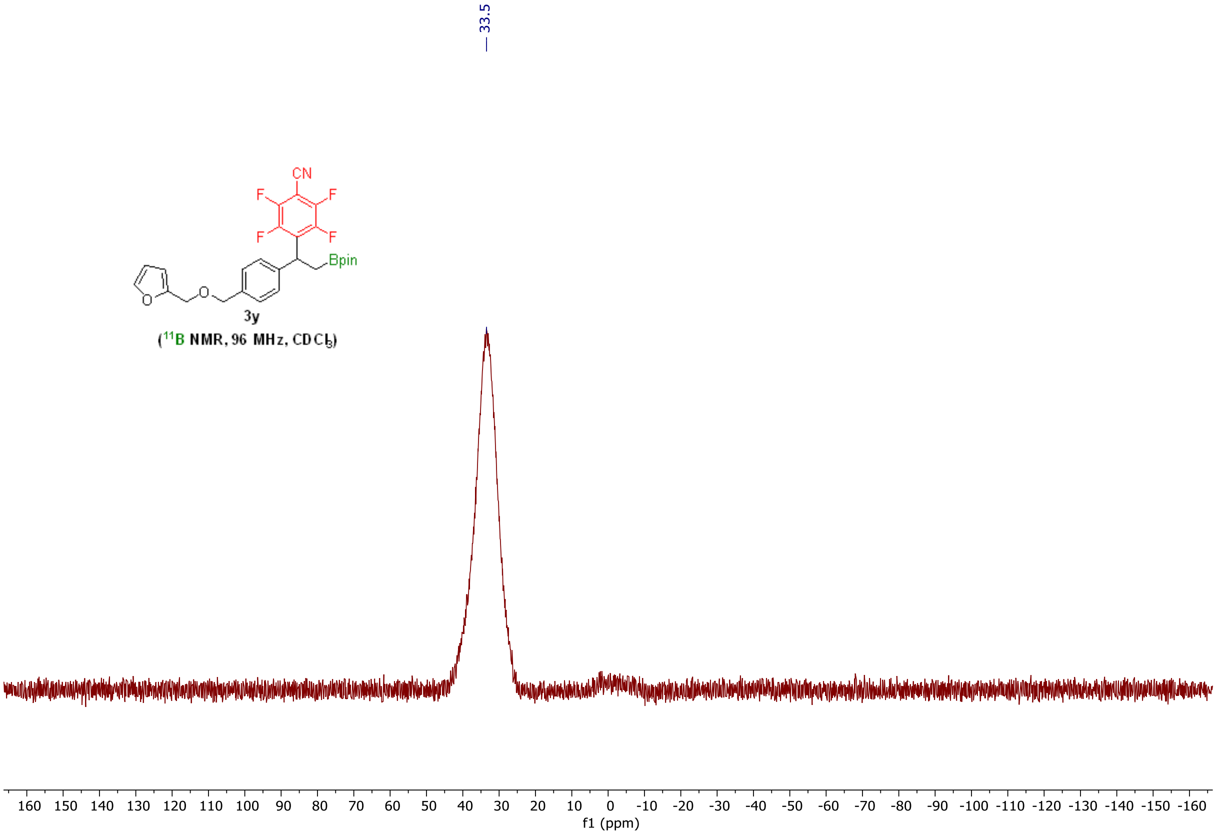

NMR Spectra of **3z**

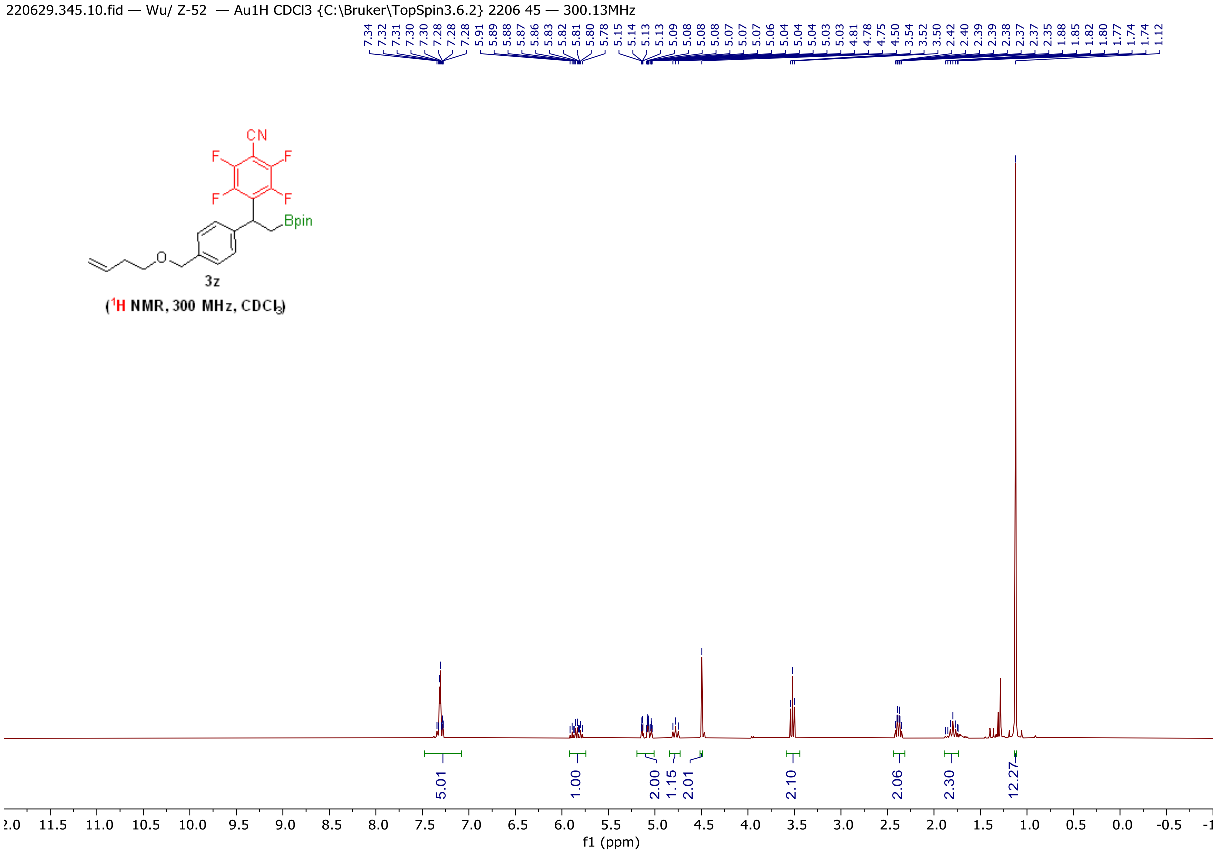

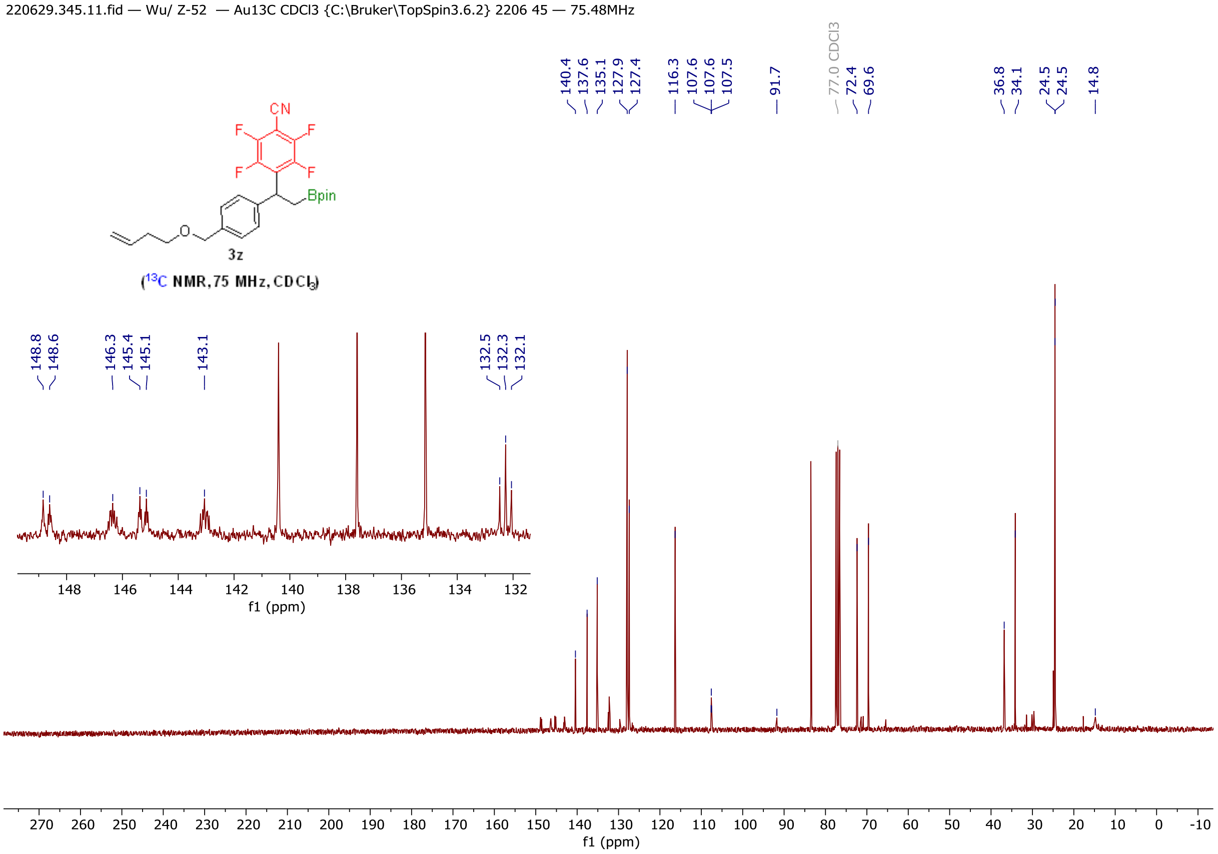

220629.345.12.fid — Wu/ Z-52 — Au19F CDCl3 {C:\Bruker\TopSpin3.6.2} 2206 45 — 282.39MHz

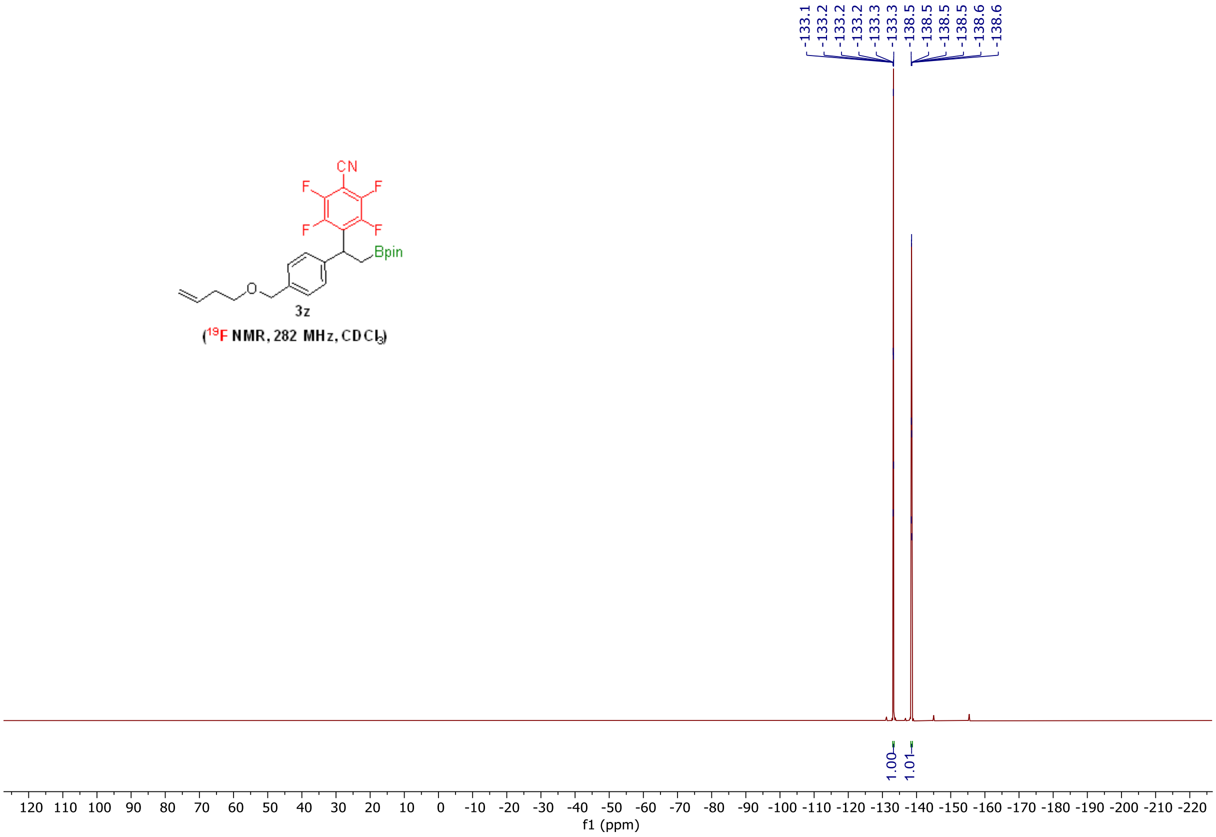

220629.345.13.fid — Wu/ Z-52 — Au11B CDCl3 {C:\Bruker\TopSpin3.6.2} 2206 45 — 96.29MHz

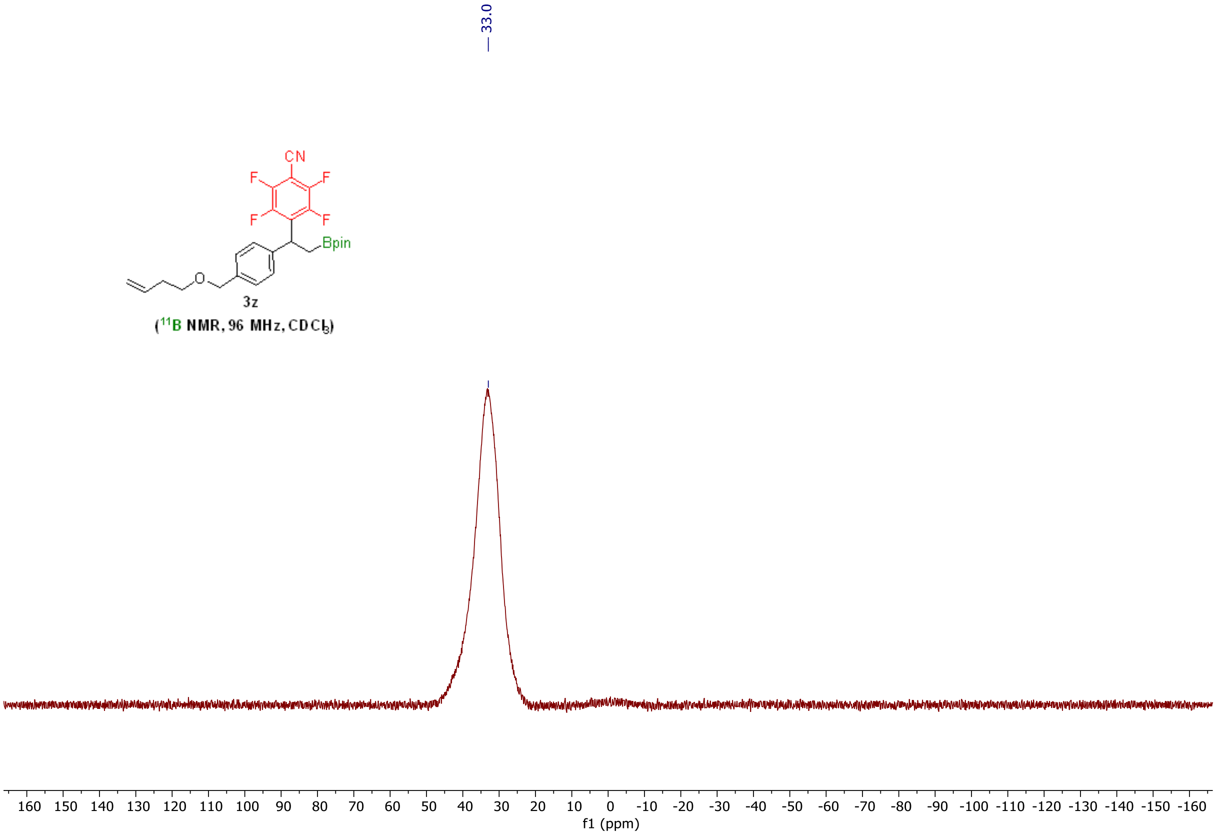

NMR Spectra of **3aa**

220707.314.10.fid — Fupeng Wu Z-65 — Au1H CDCl3 {C:\Bruker\TopSpin3.6.2} 2207 14 — 300.13MHz

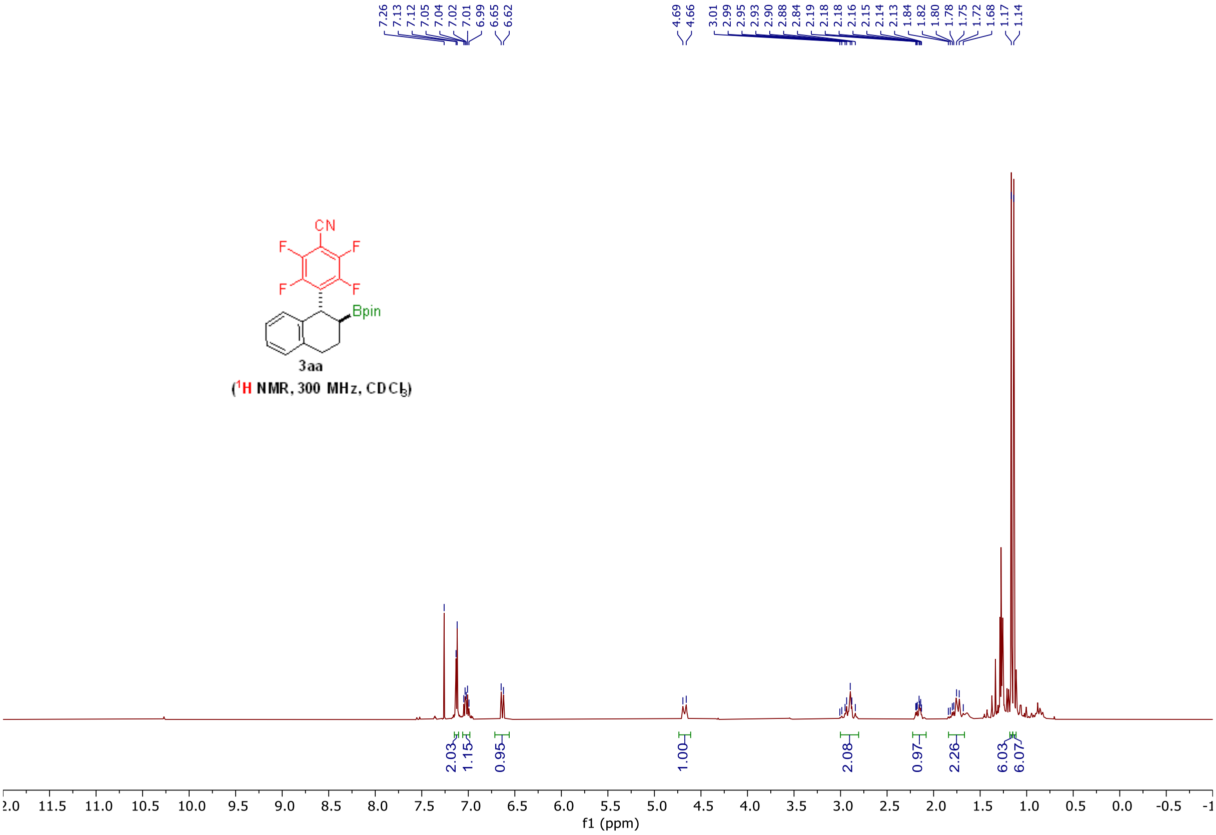

220707.314.11.fid — Fupeng Wu Z-65 — Au13C CDCl3 {C:\Bruker\TopSpin3.6.2} 2207 14 — 75.48MHz

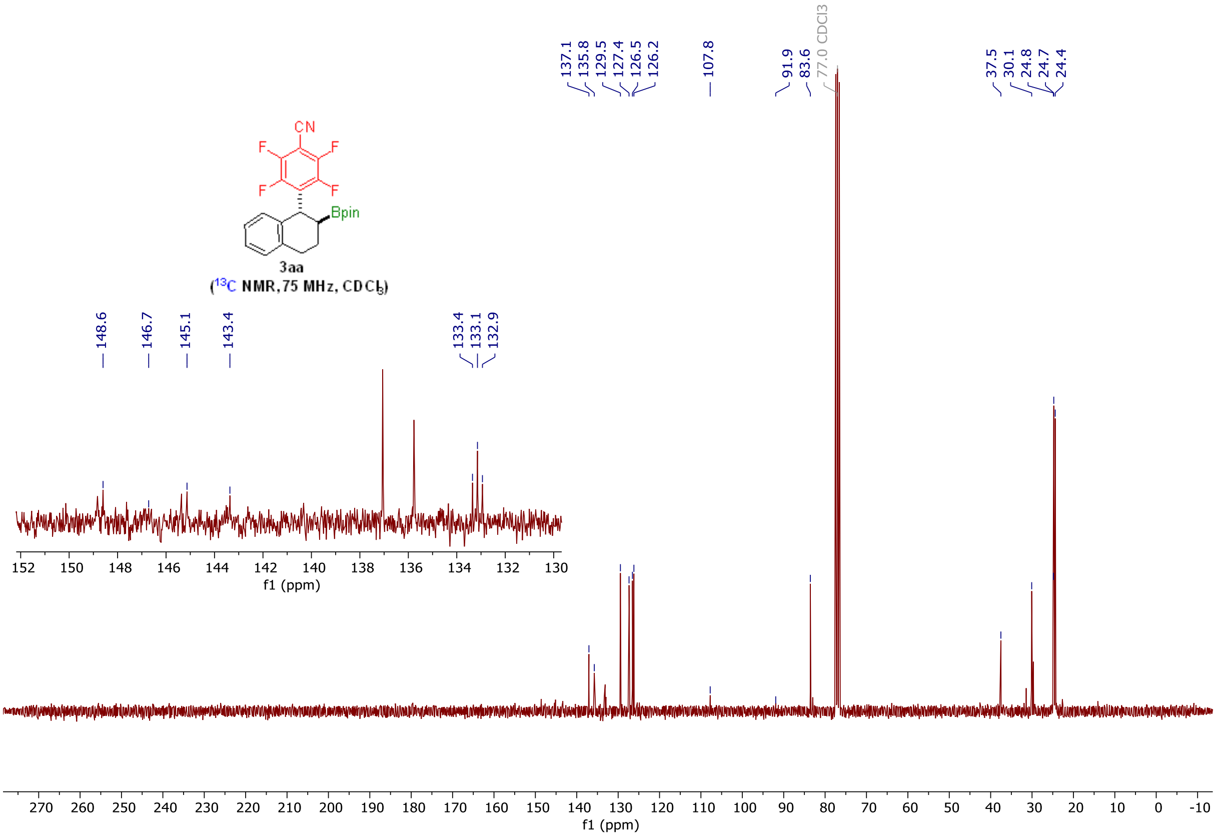

220707.314.12.fid — Fupeng Wu Z-65 — Au19F CDCl3 {C:\Bruker\TopSpin3.6.2} 2207 14 — 282.39MHz

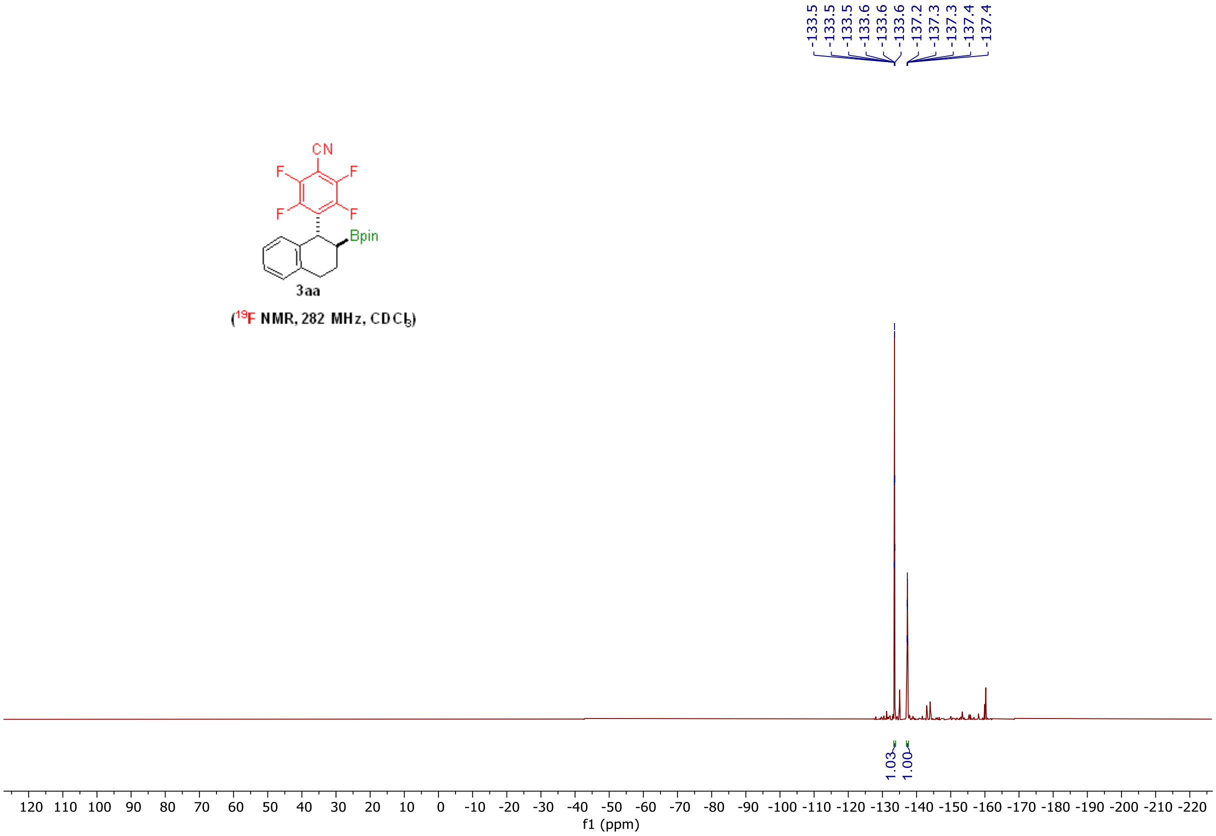

220707.314.13.fid — Fupeng Wu Z-65 — Au11B CDCl3 {C:\Bruker\TopSpin3.6.2} 2207 14 — 96.29MHz

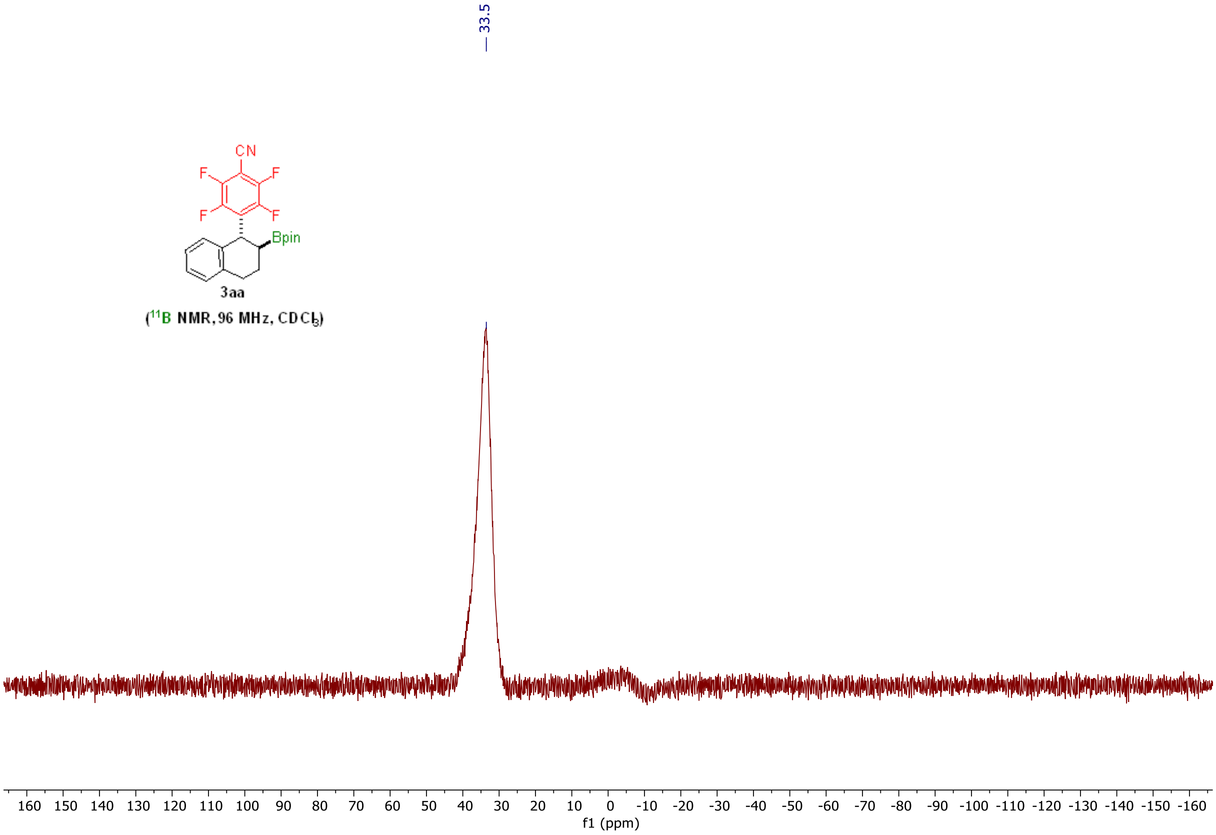

NMR Spectra of **3bb**

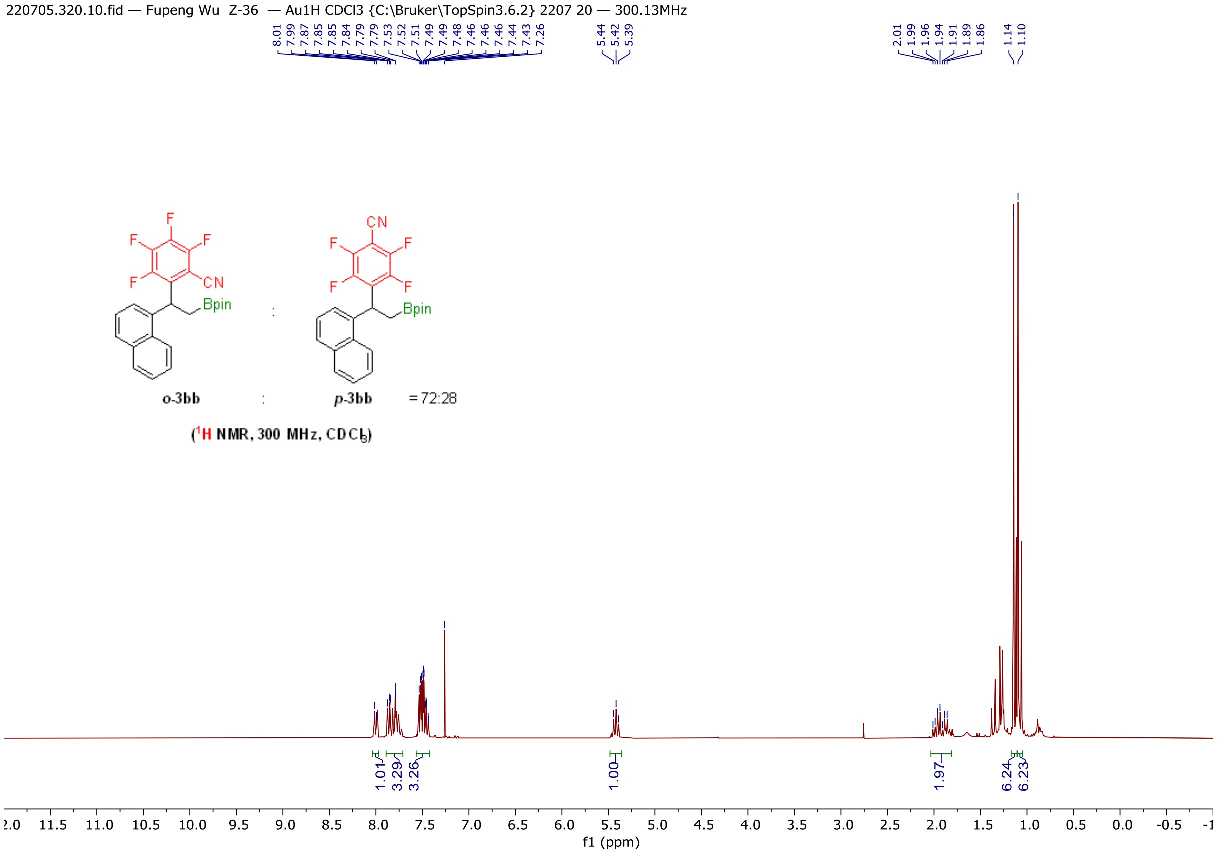

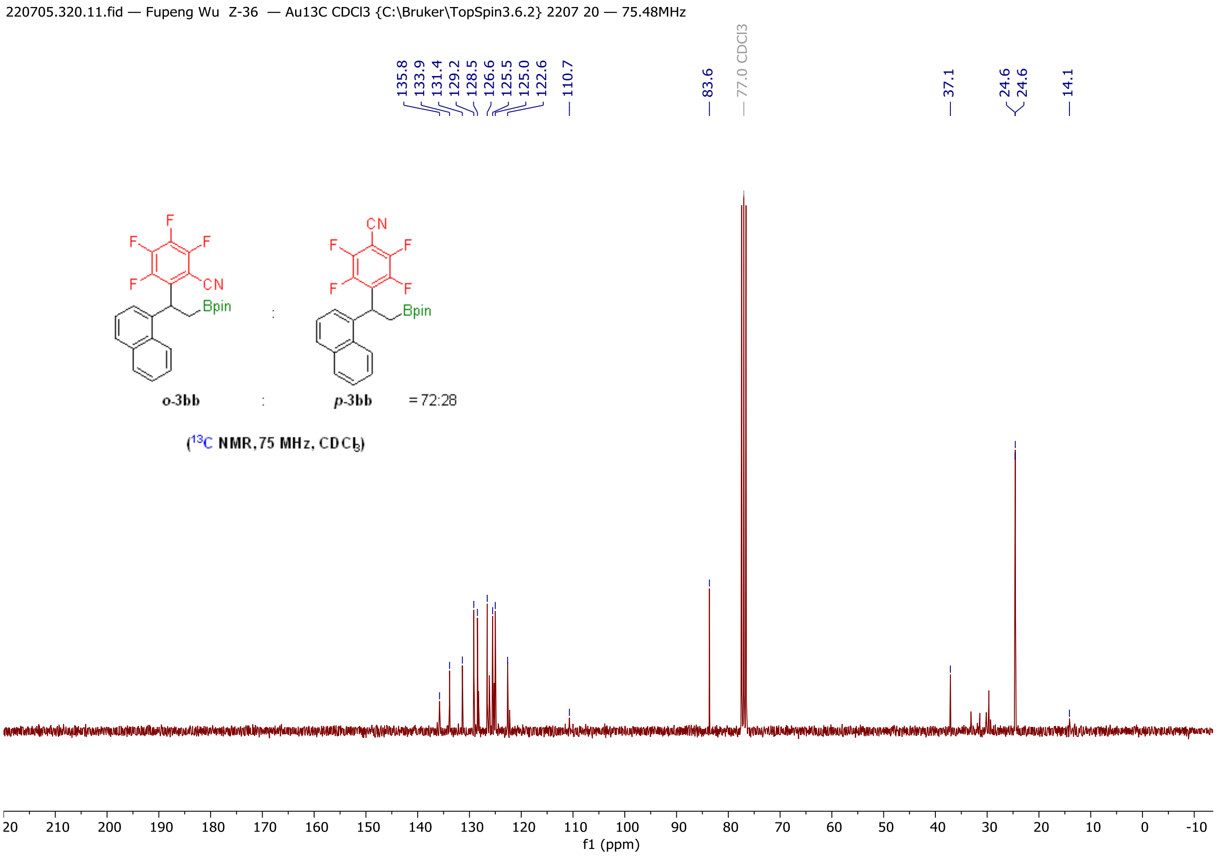

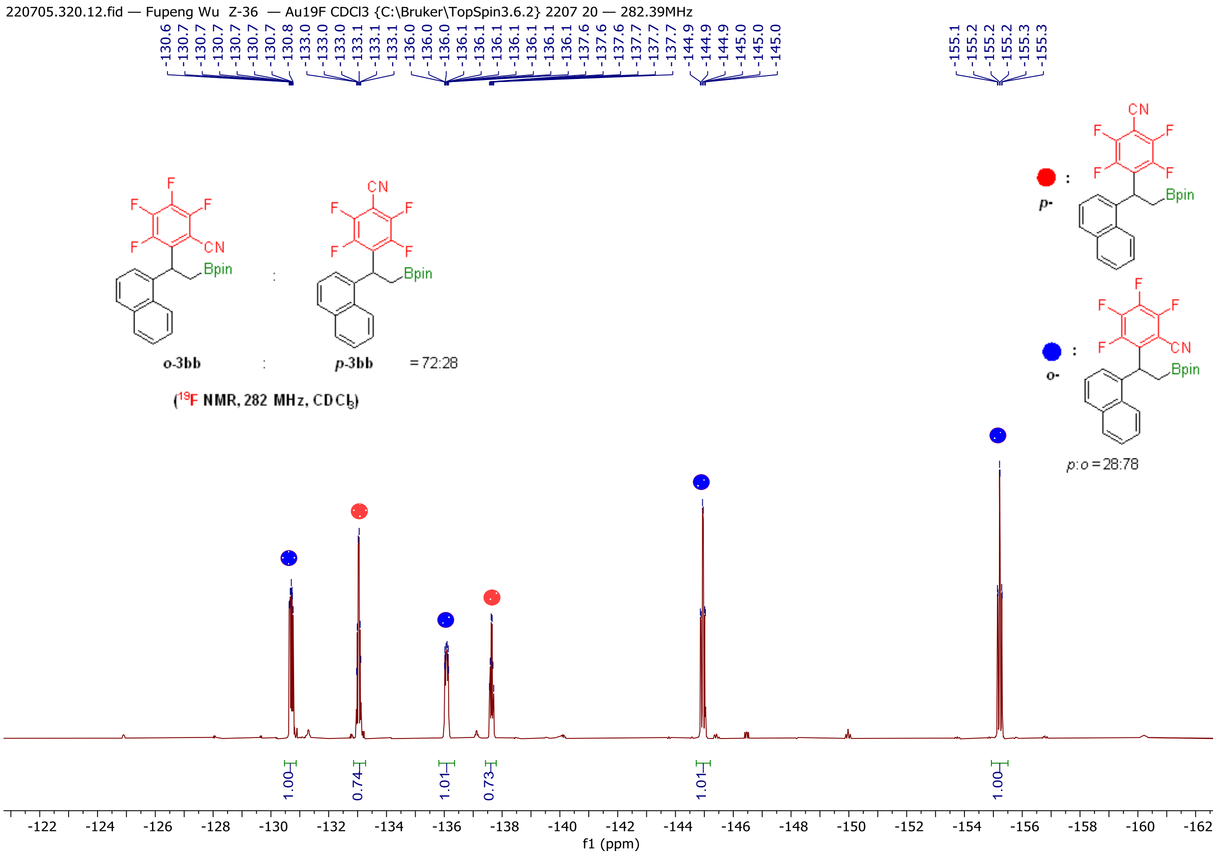
220705.320.12.fid — Fupeng Wu Z-36 — Au19F CDCl3 {C:\Bruker\TopSpin3.6.2} 2207 20 — 282.39MHz
o-3bb : p-3bb = 72:28
(19F NMR, 282 MHz, CDCl3)
p-
o-
p:o = 28:78
f1 (ppm)

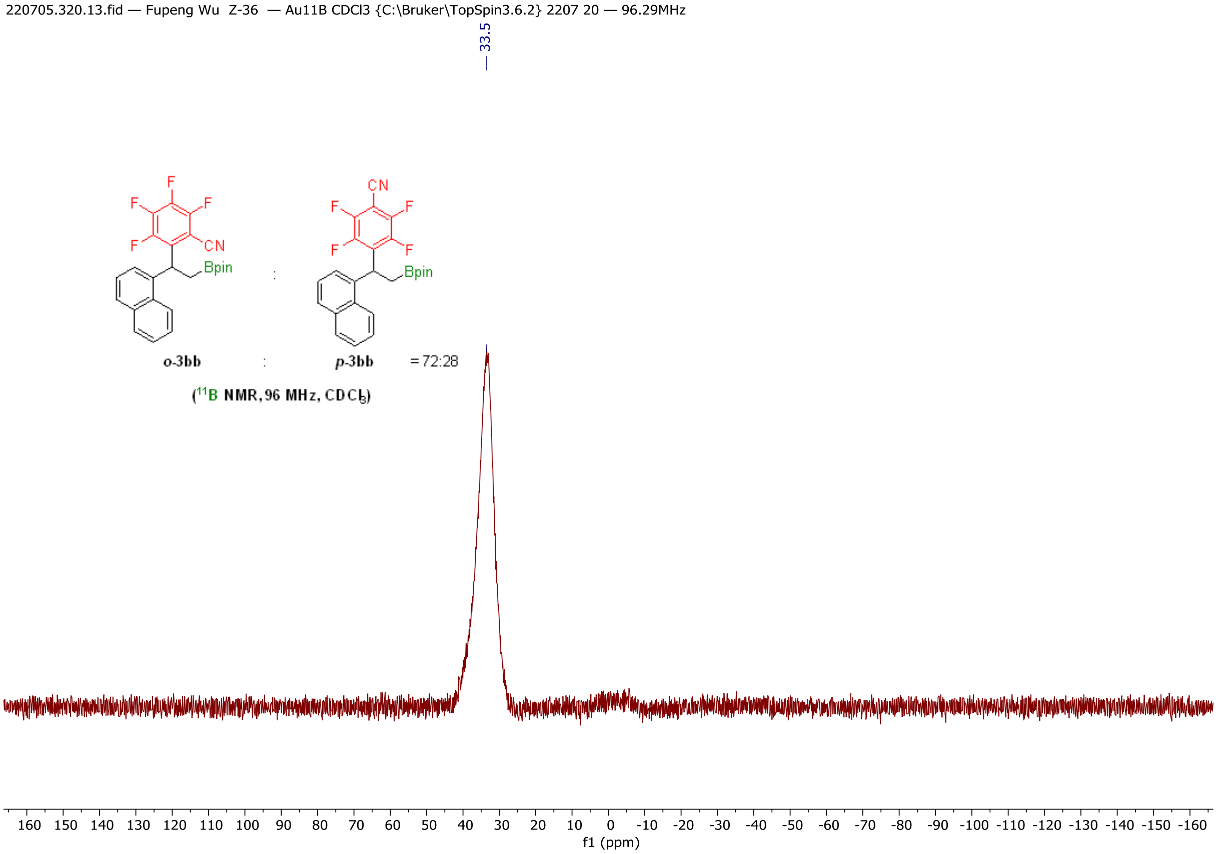
220705.320.13.fid — Fupeng Wu Z-36 — Au11B CDCl3 {C:\Bruker\TopSpin3.6.2} 2207 20 — 96.29MHz
33.5
o-3bb : p-3bb = 72:28
(11B NMR, 96 MHz, CDCl3)
f1 (ppm)

NMR Spectra of **3cc**

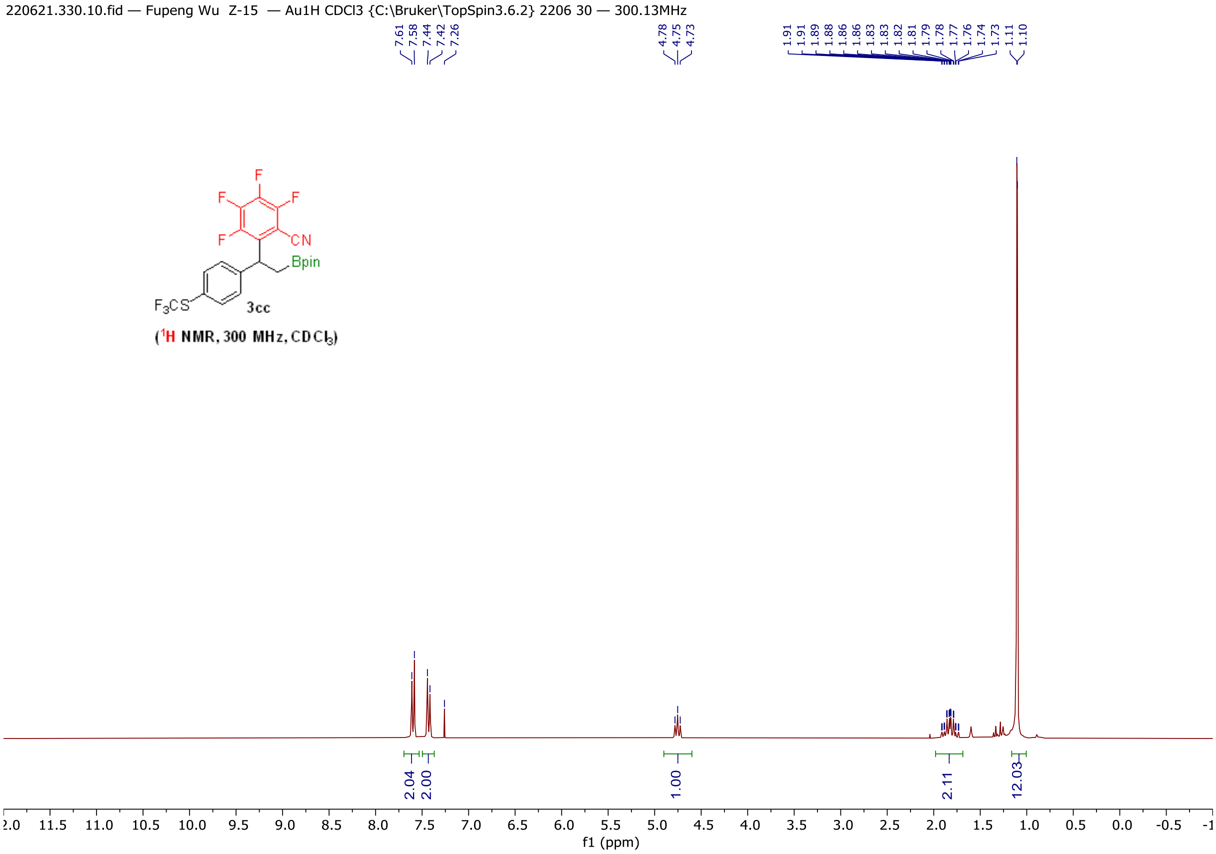

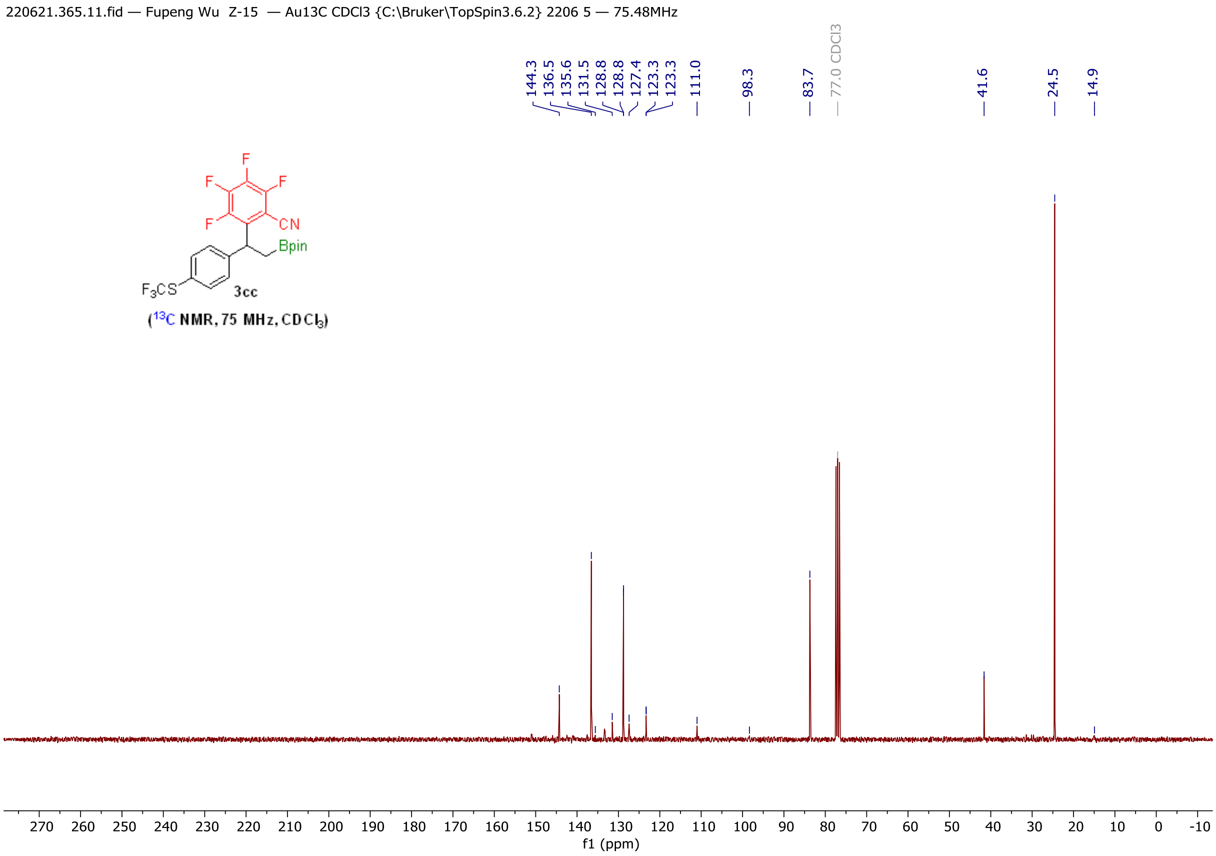

220621.365.12.fid — Fupeng Wu Z-15 — Au19F CDCl3 {C:\Bruker\TopSpin3.6.2} 2206 5 — 282.39MHz

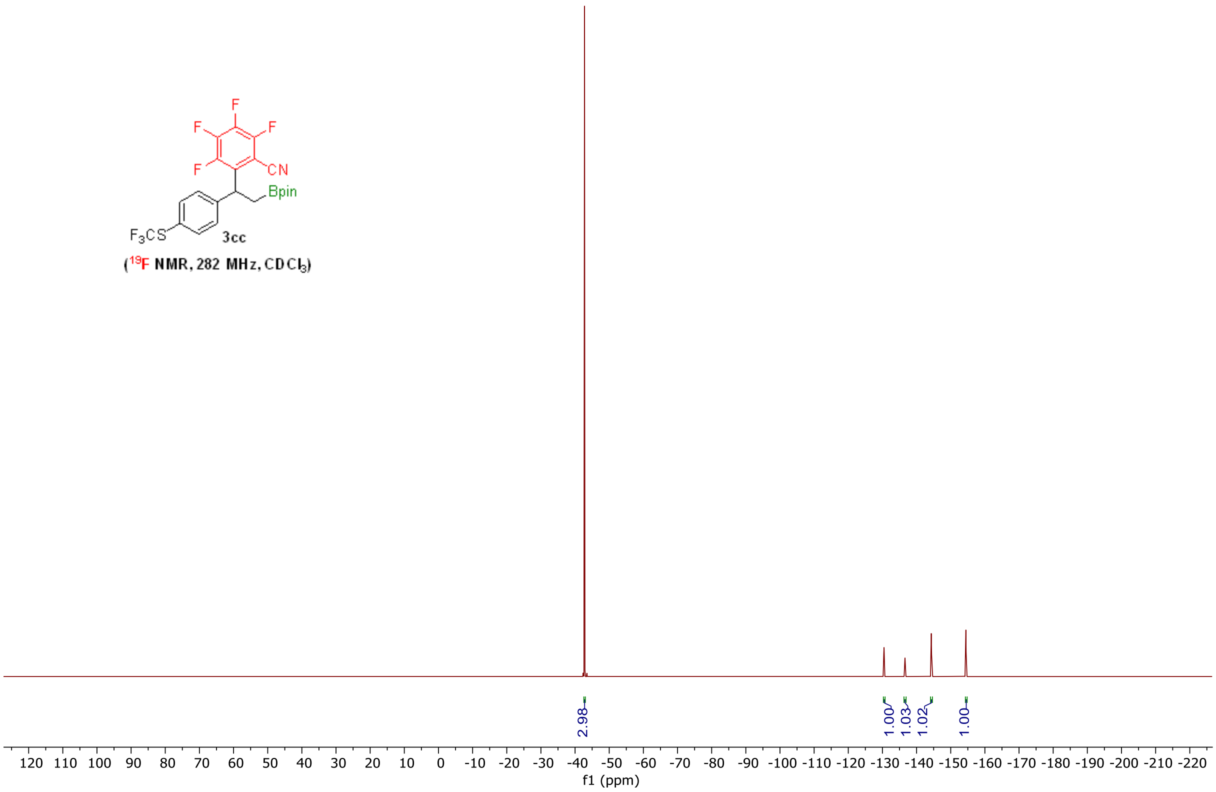

220621.365.10.fid — Fupeng Wu Z-15 — Au11B CDCl3 {C:\Bruker\TopSpin3.6.2} 2206 5 — 96.29MHz

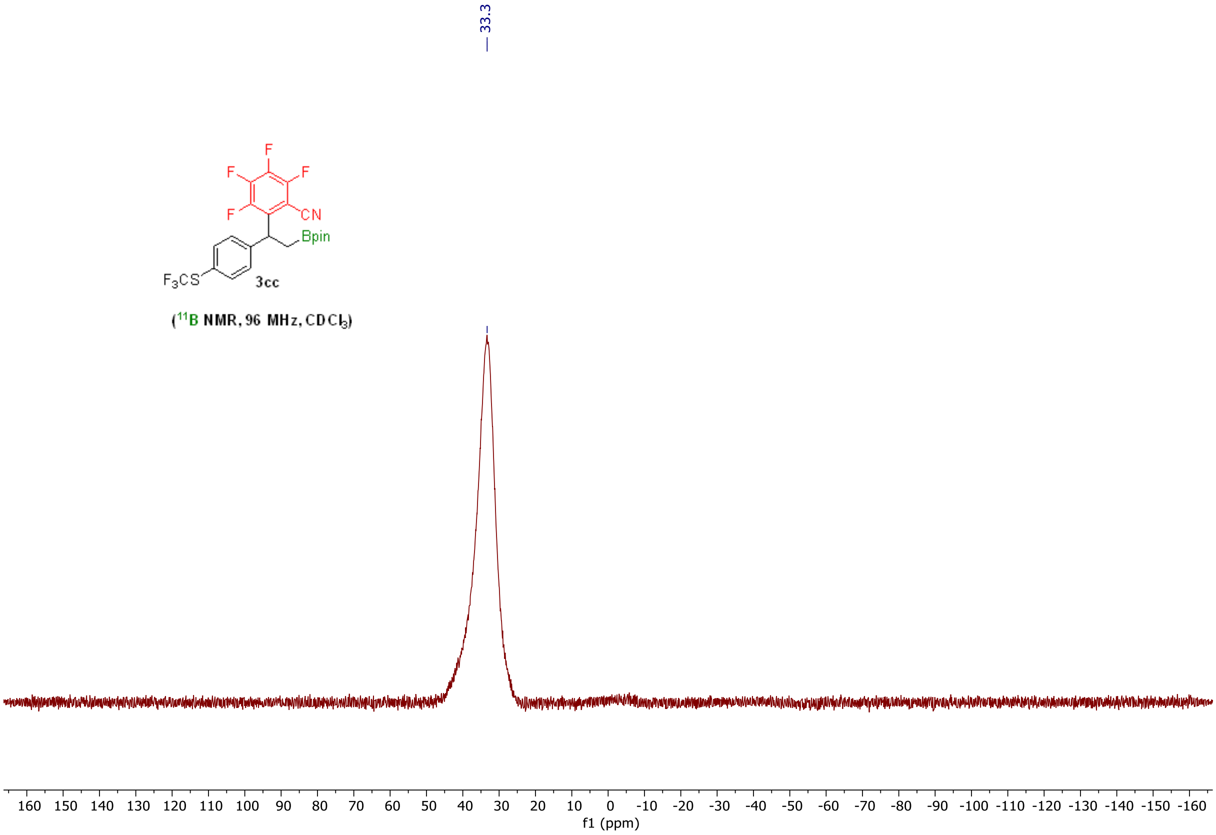

NMR Spectra of **3dd**

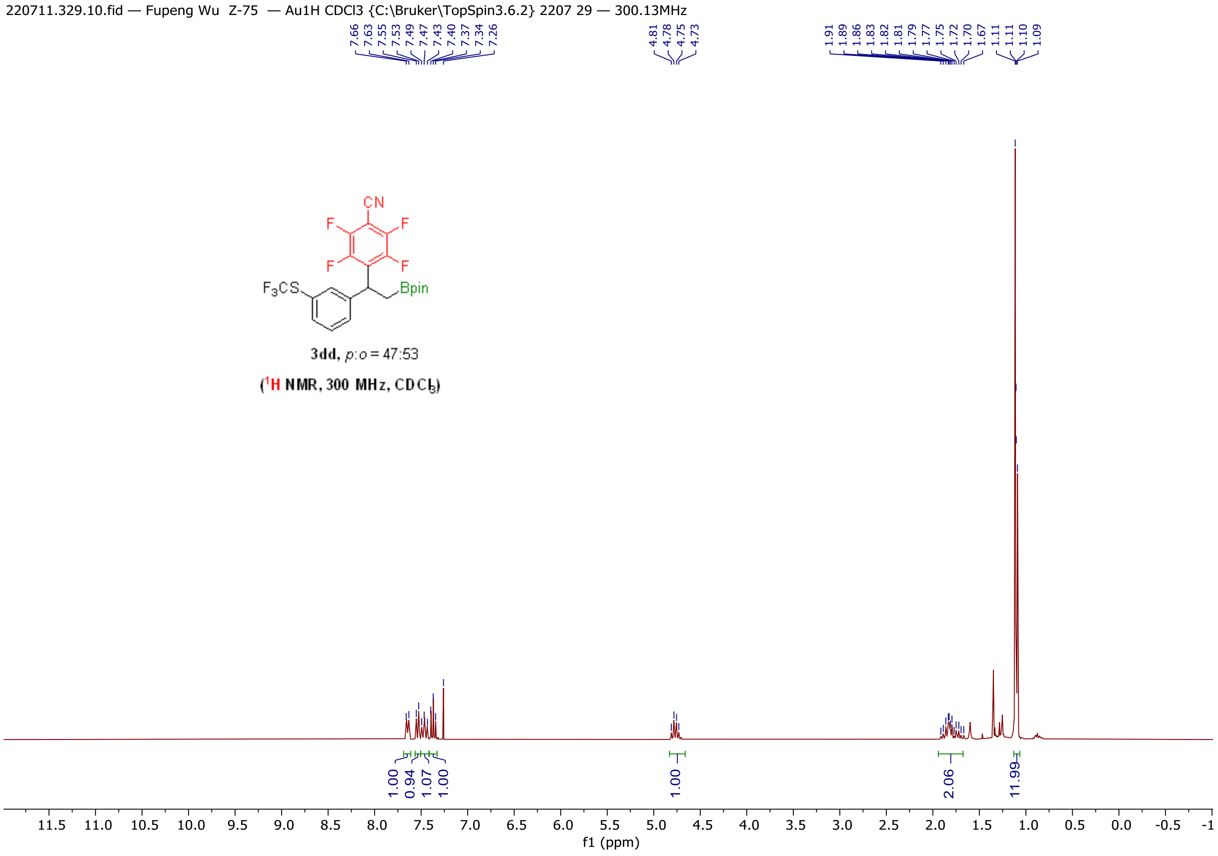

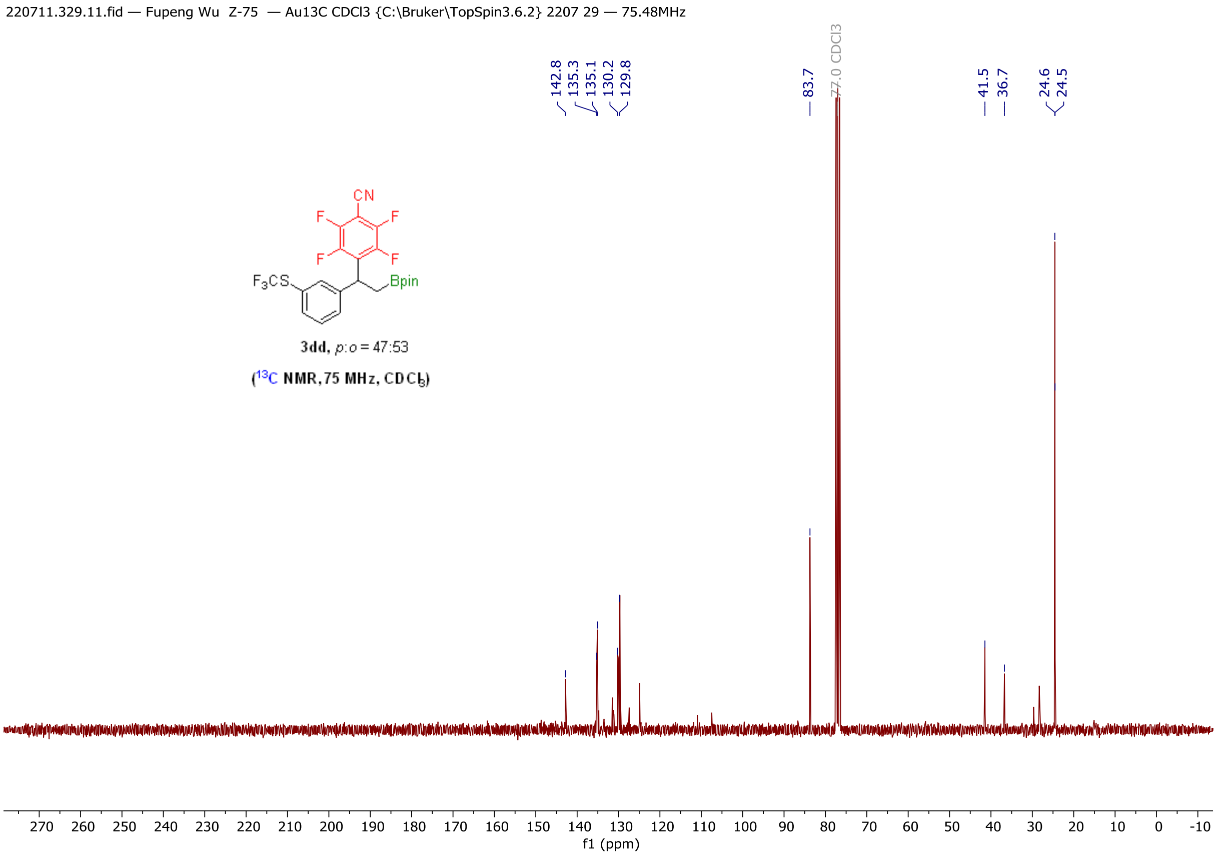

220711.329.12.fid — Fupeng Wu Z-75 — Au19F CDCl3 {C:\Bruker\TopSpin3.6.2} 2207 29 — 282.39MHz

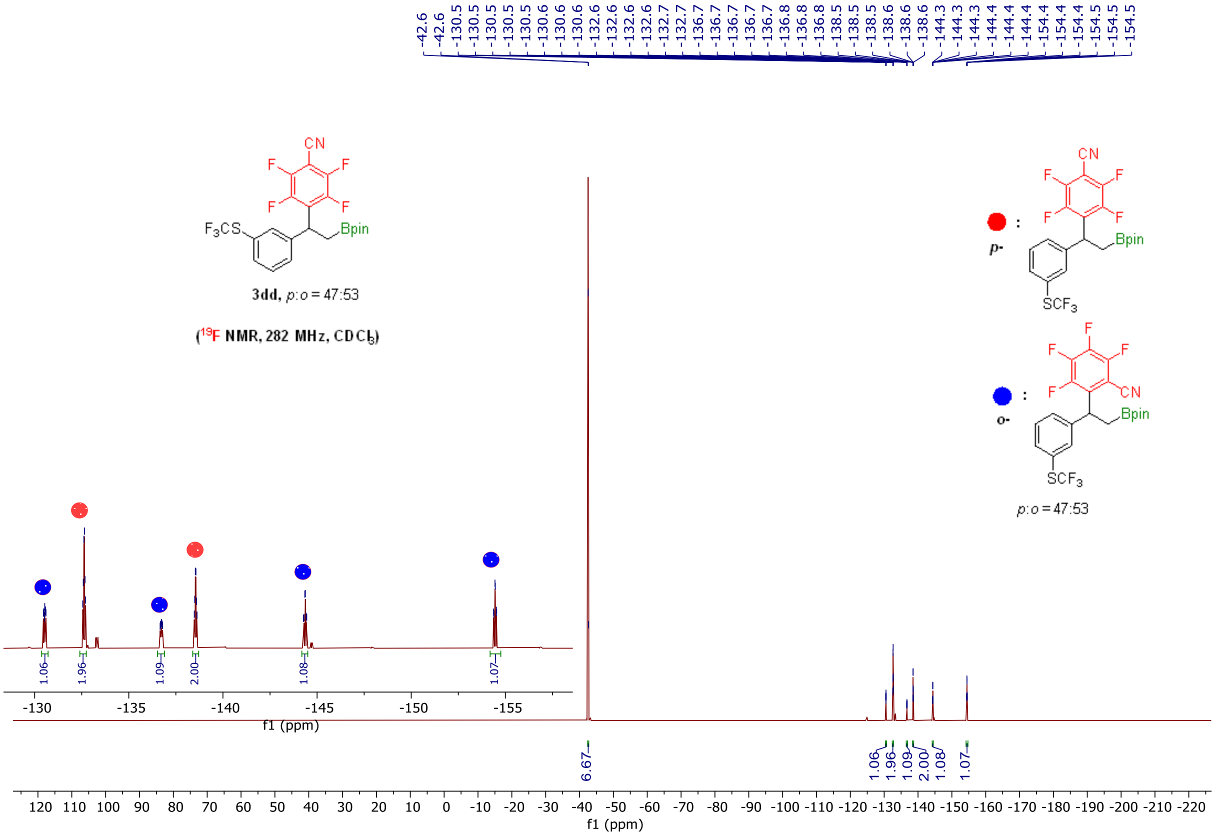

220711.329.13.fid — Fupeng Wu Z-75 — Au11B CDCl3 {C:\Bruker\TopSpin3.6.2} 2207 29 — 96.29MHz

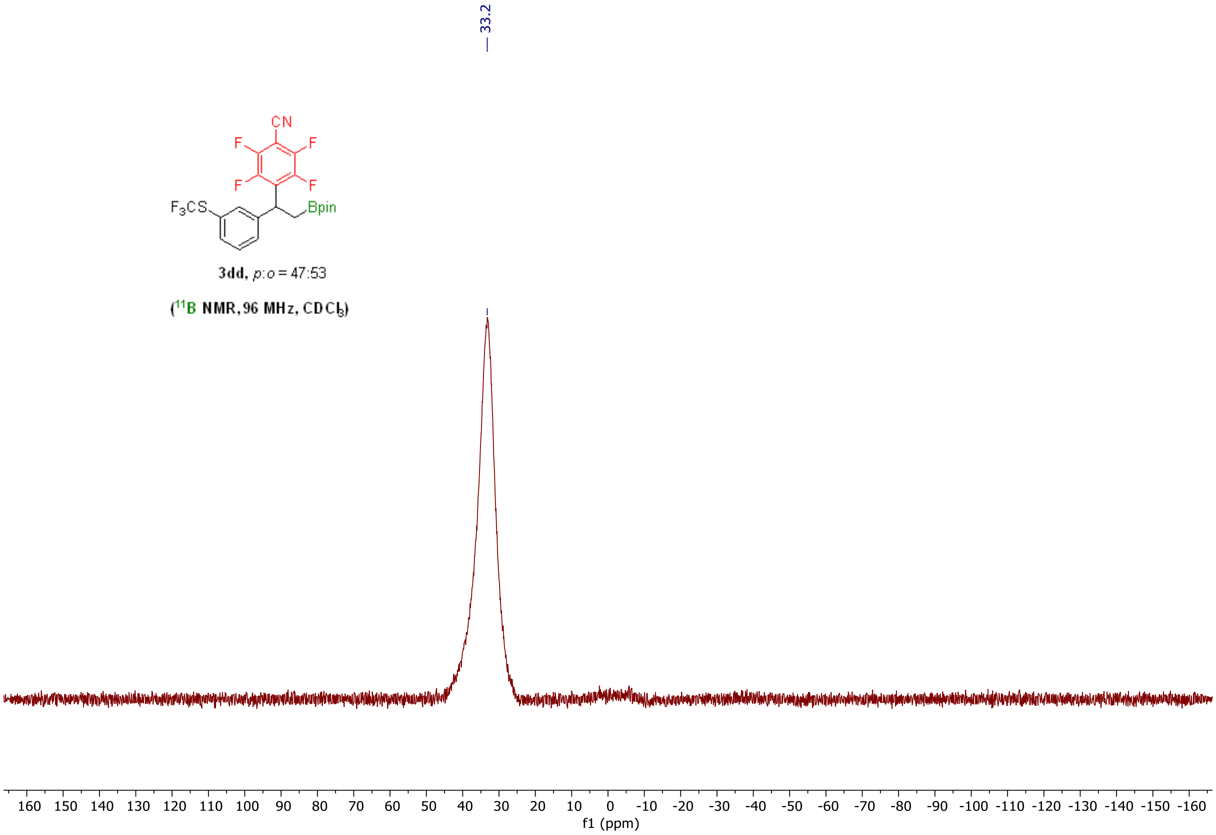

NMR Spectra of **4a**

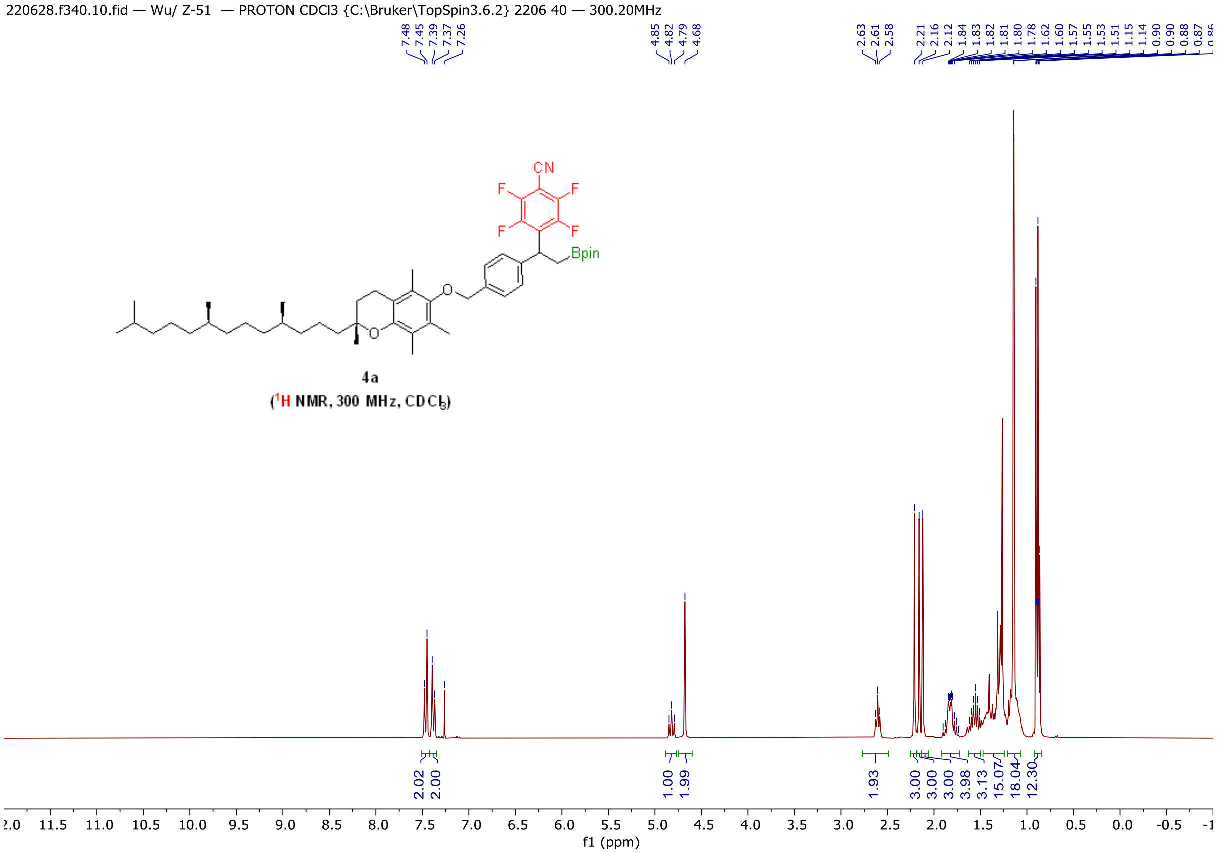

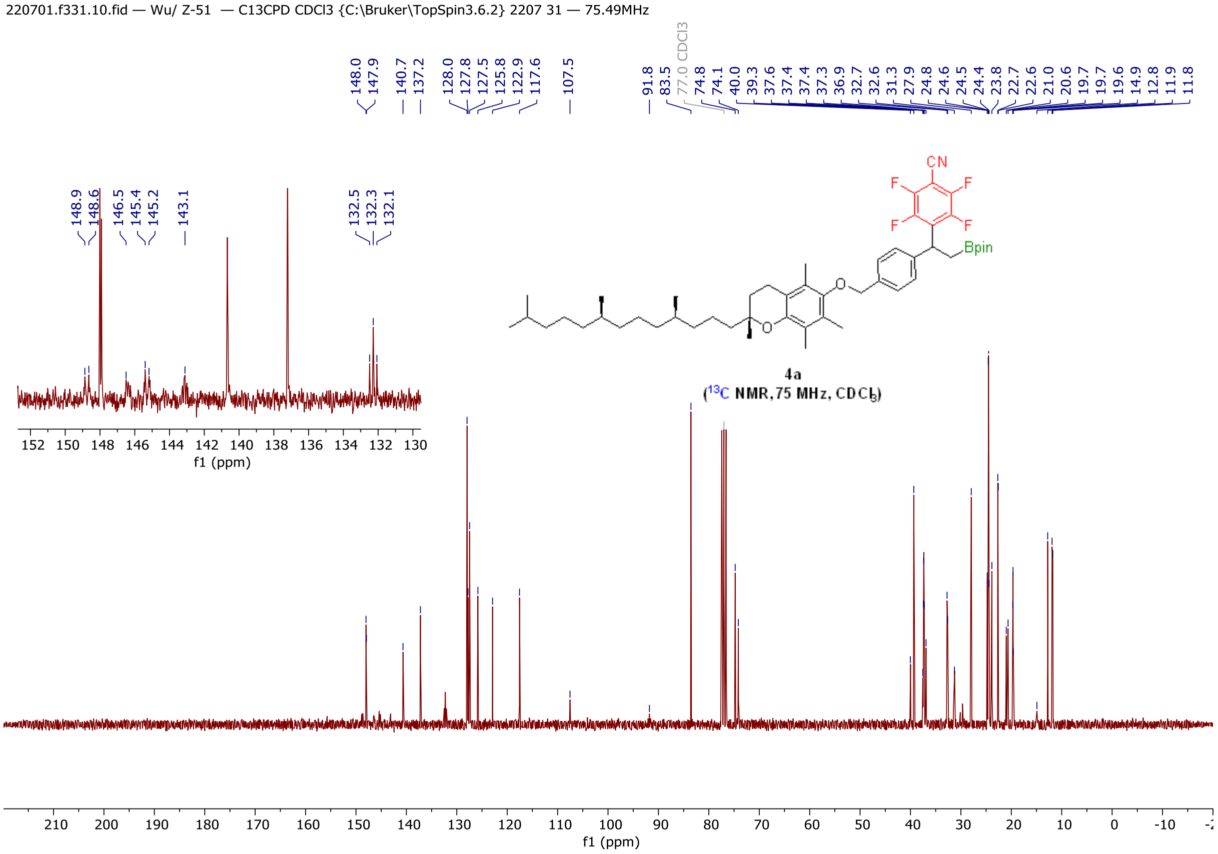

220628.f340.12.fid — Wu/ Z-51 — F19 CDCl3 {C:\Bruker\TopSpin3.6.2} 2206 40 — 282.44MHz

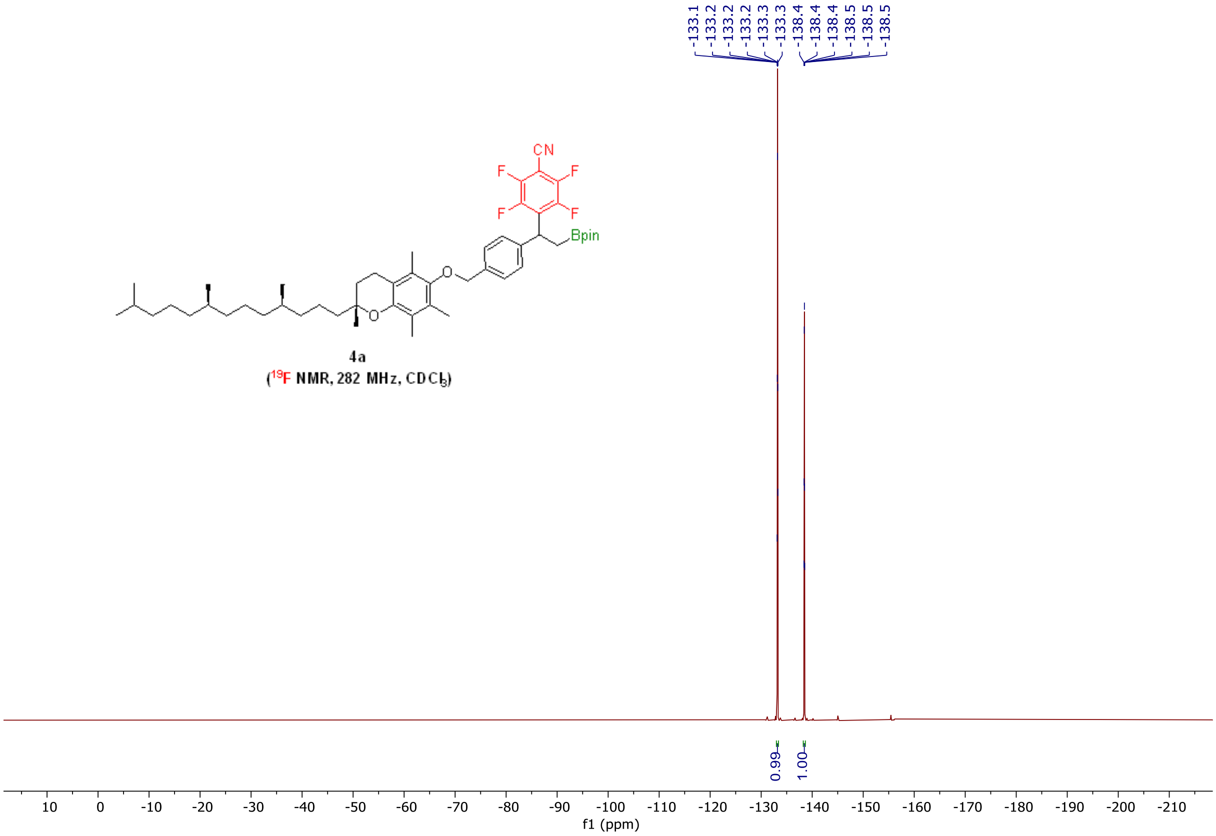

220628.f340.13.fid — Wu/ Z-51 — 11B CDCl3 {C:\Bruker\TopSpin3.6.2} 2206 40 — 96.32MHz

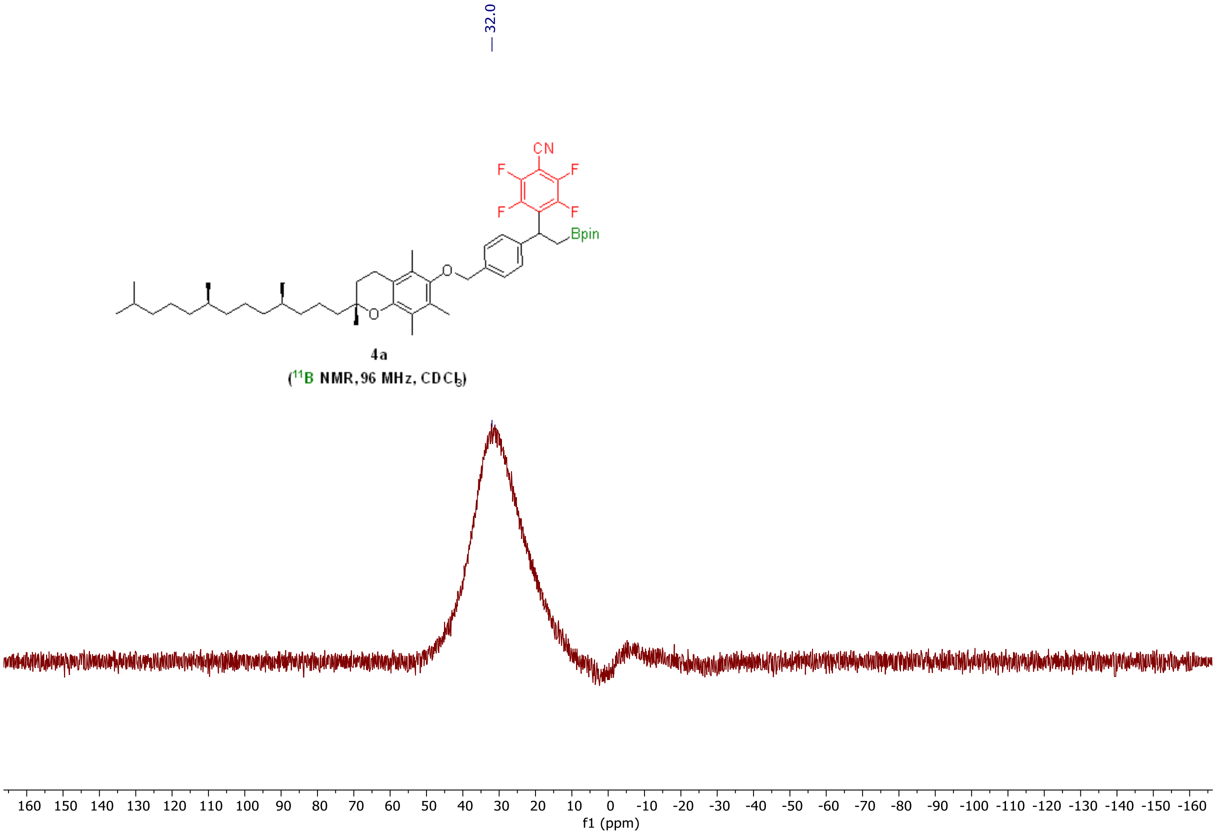

NMR Spectra of **4b**

220711.332.10.fid — Fupeng Wu Z-77 — Au1H CDCl3 {C:\Bruker\TopSpin3.6.2} 2207 32 — 300.13MHz

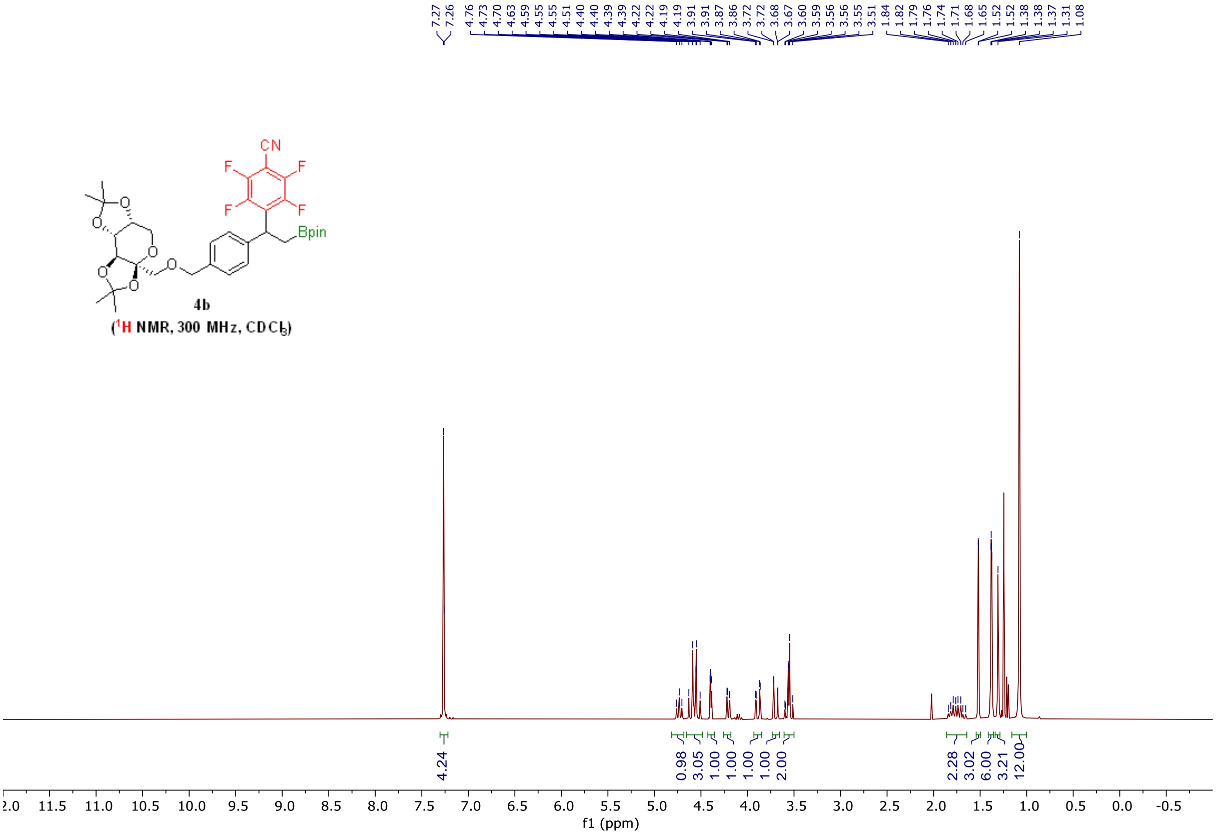

220711.332.11.fid — Fupeng Wu Z-77 — Au13C CDCl3 {C:\Bruker\TopSpin3.6.2} 2207 32 — 75.48MHz

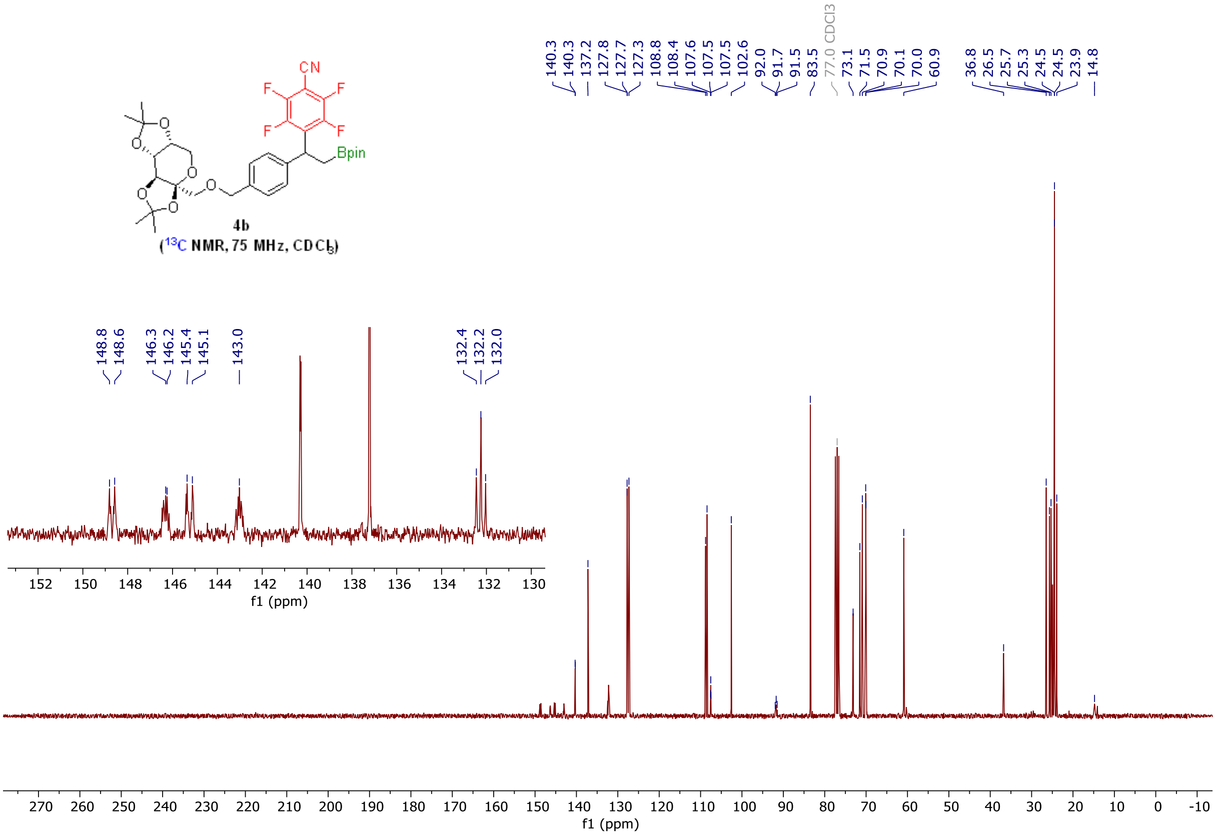

220711.332.12.fid — Fupeng Wu Z-77 — Au19F CDCl3 {C:\Bruker\TopSpin3.6.2} 2207 32 — 282.39MHz

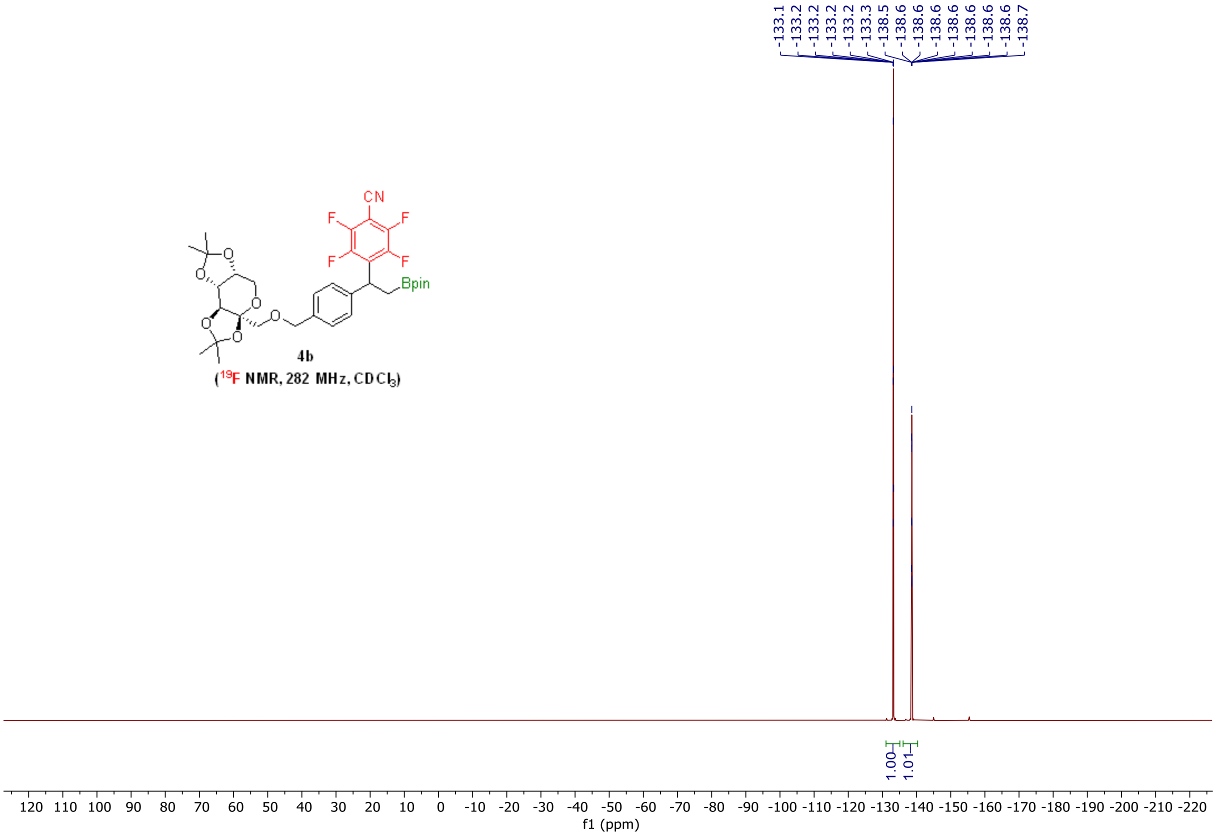

220711.332.13.fid — Fupeng Wu Z-77 — Au11B CDCl3 {C:\Bruker\TopSpin3.6.2} 2207 32 — 96.29MHz

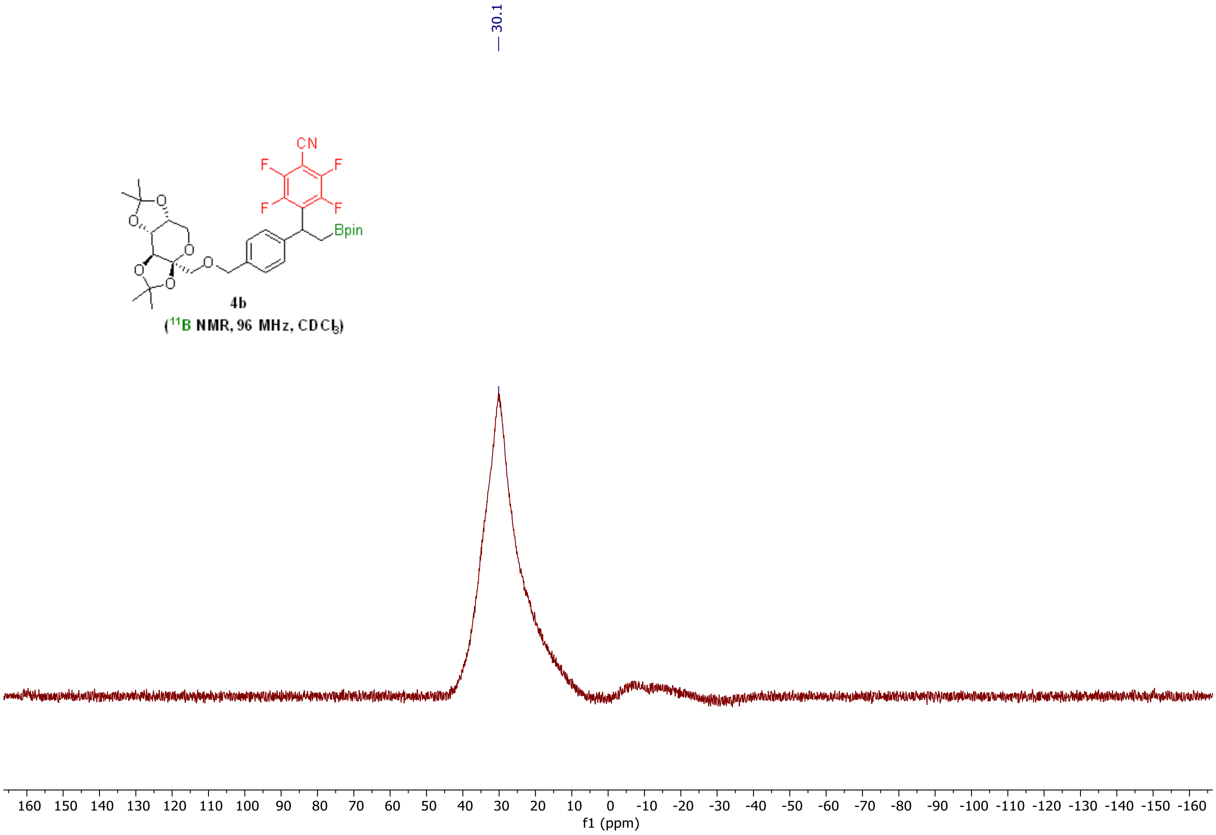

NMR Spectra of **4c**

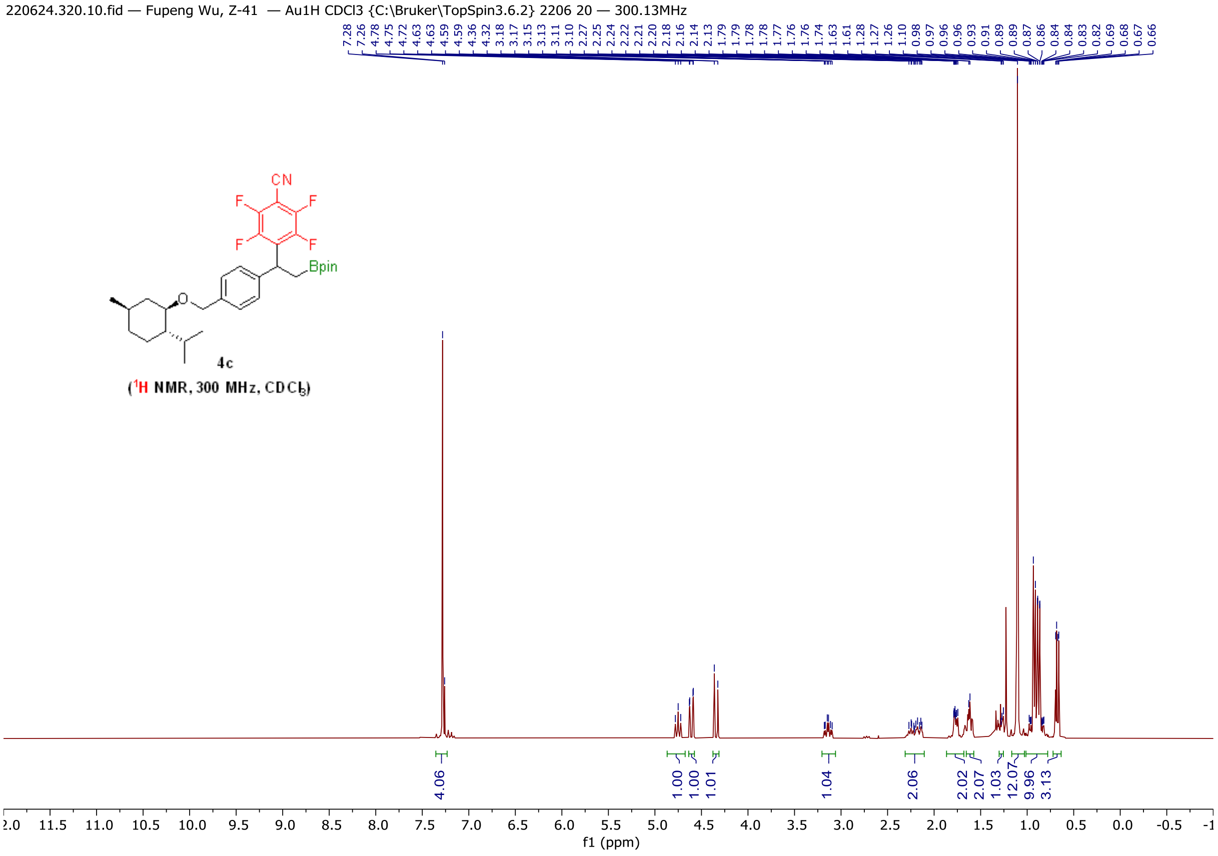

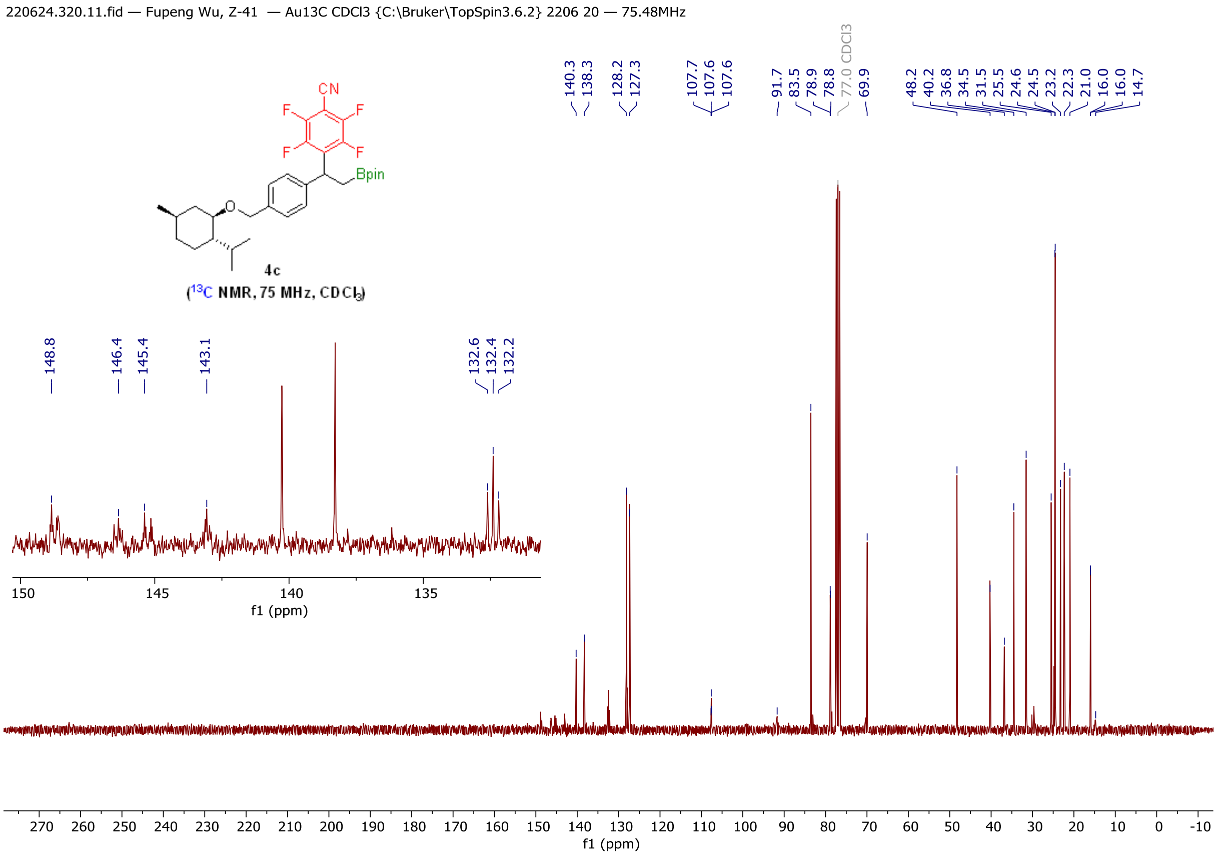

220624.320.12.fid — Fupeng Wu, Z-41 — Au19F CDCl3 {C:\Bruker\TopSpin3.6.2} 2206 20 — 282.39MHz

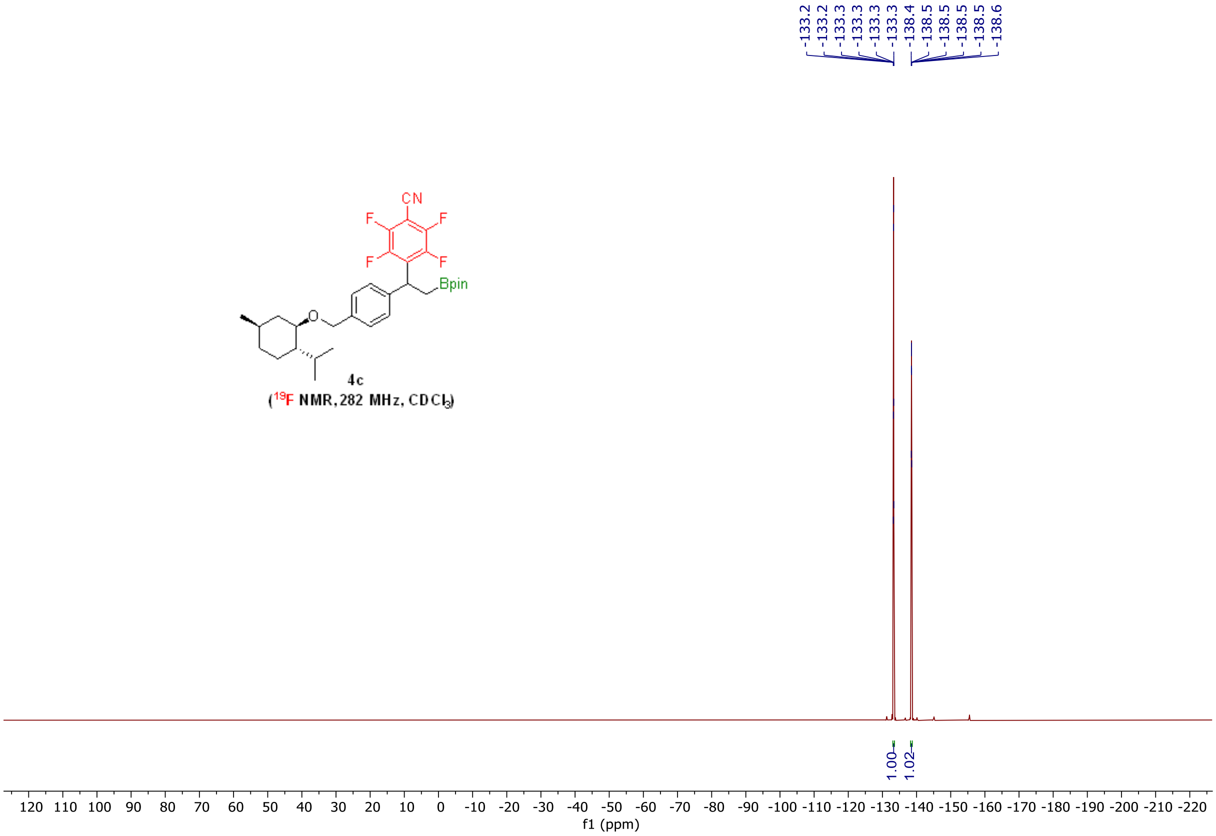

220624.320.13.fid — Fupeng Wu, Z-41 — Au11B CDCl3 {C:\Bruker\TopSpin3.6.2} 2206 20 — 96.29MHz

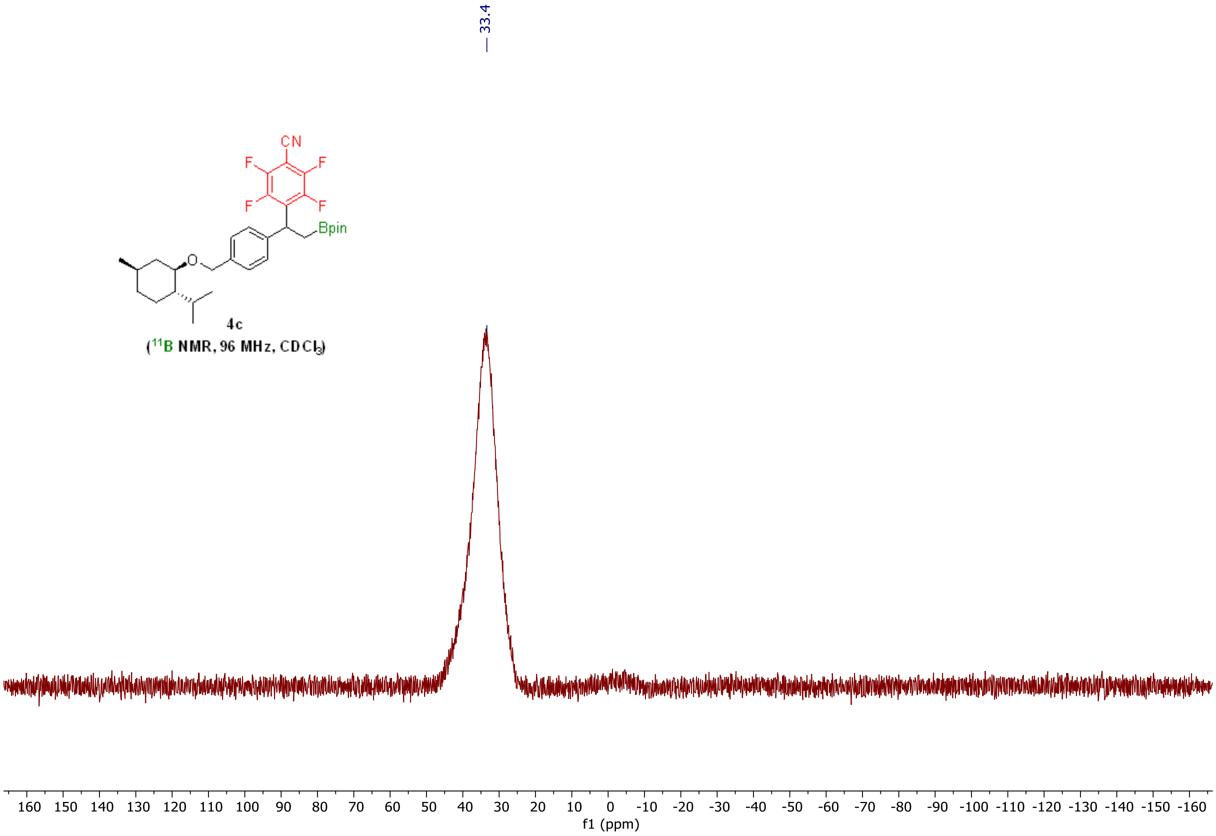

NMR Spectra of **5a**

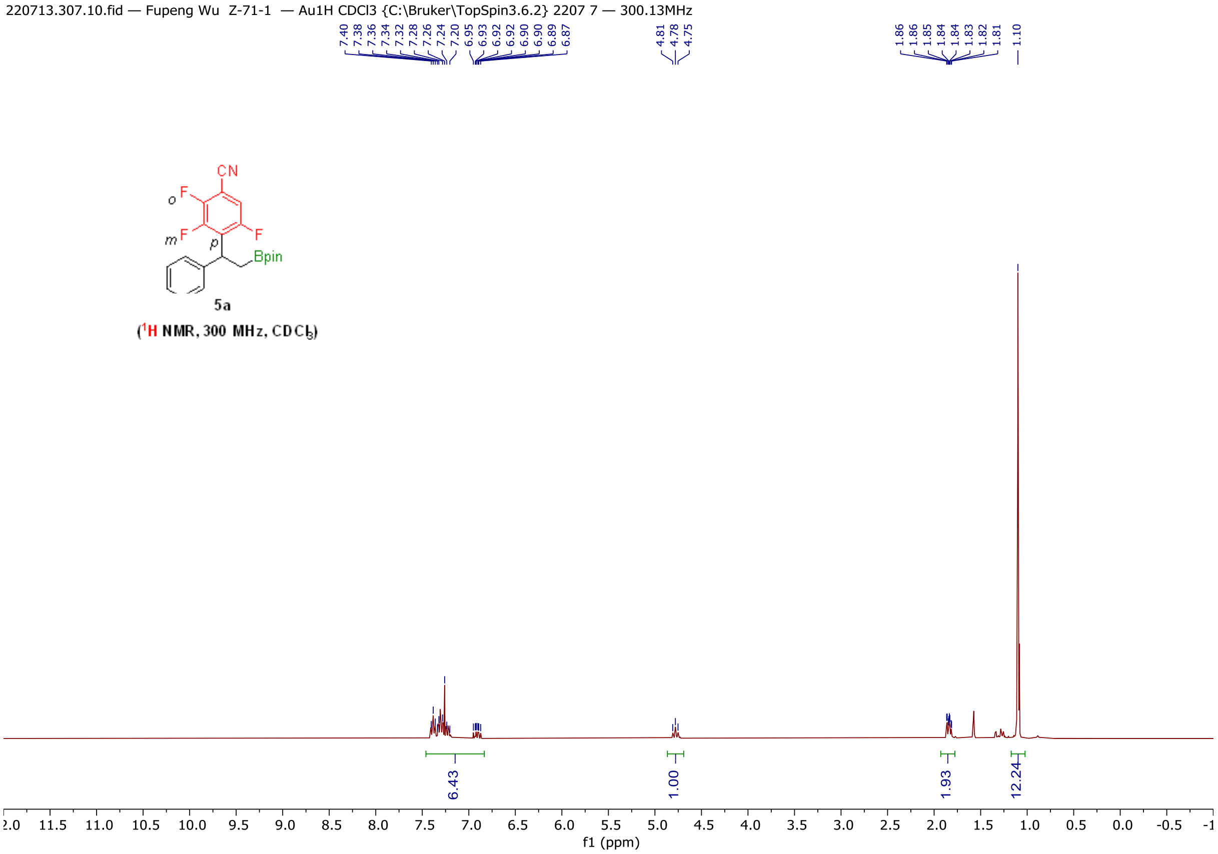

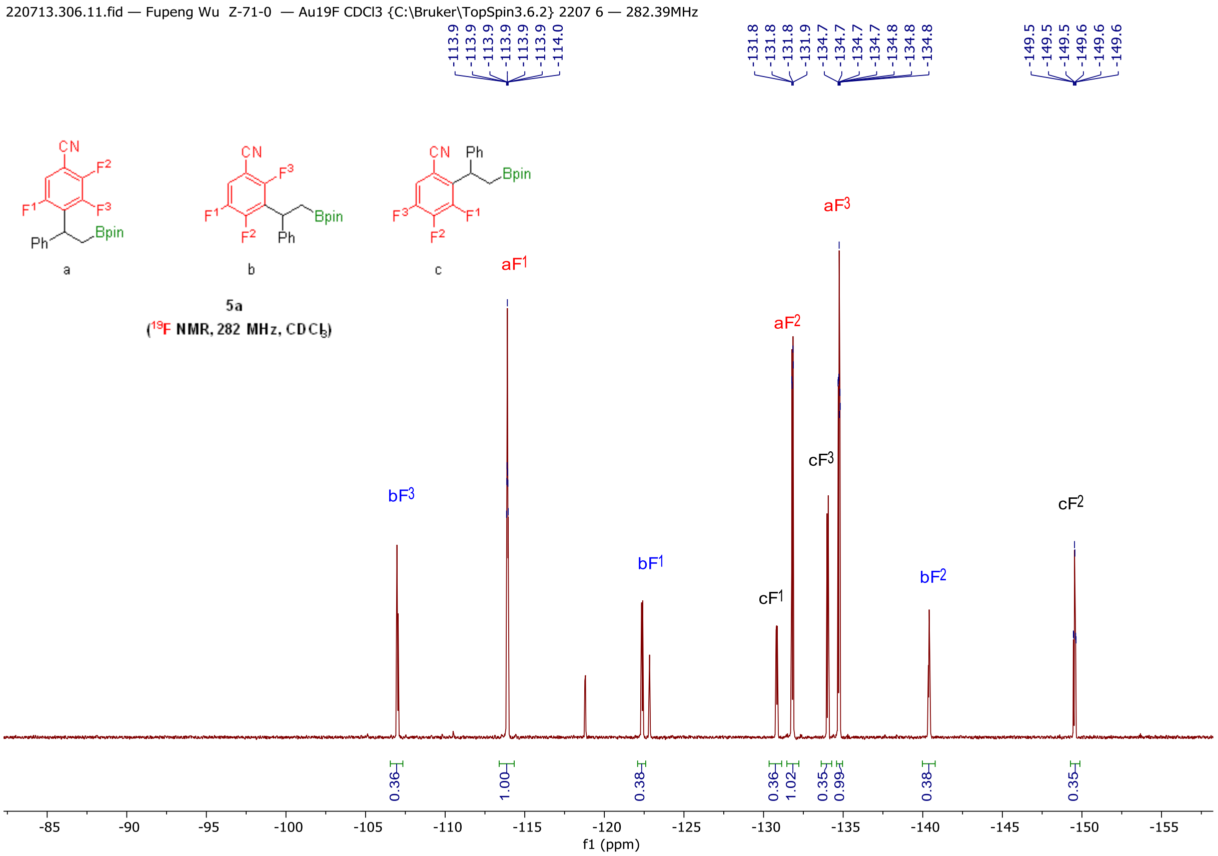

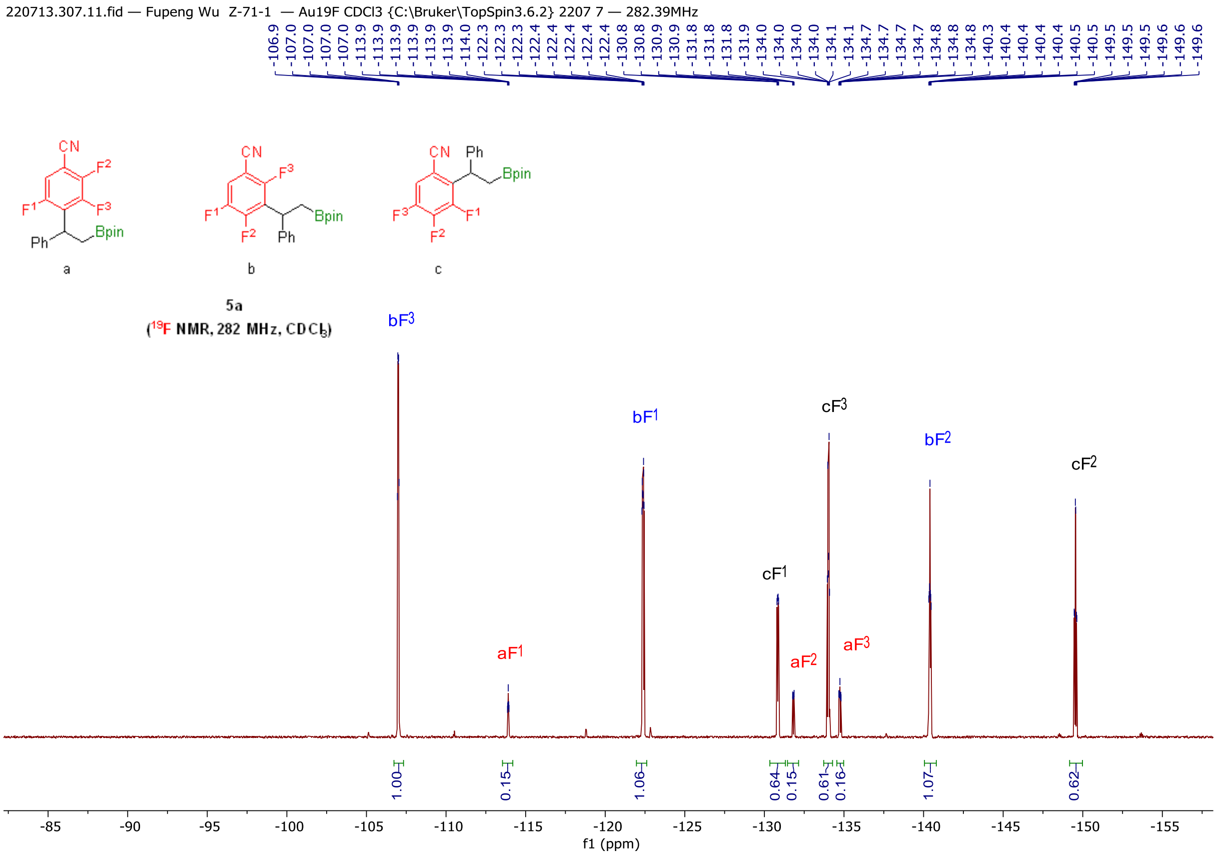
220713.307.11.fid — Fupeng Wu Z-71-1 — Au19F CDCl3 {C:\Bruker\TopSpin3.6.2} 2207 7 — 282.39MHz
a
b
c
5a
(19F NMR, 282 MHz, CDCl3)
bF3
aF1
bF1
cF1
aF2
cF3
aF3
bF2
cF2
f1 (ppm)

NMR Spectra of **5b**

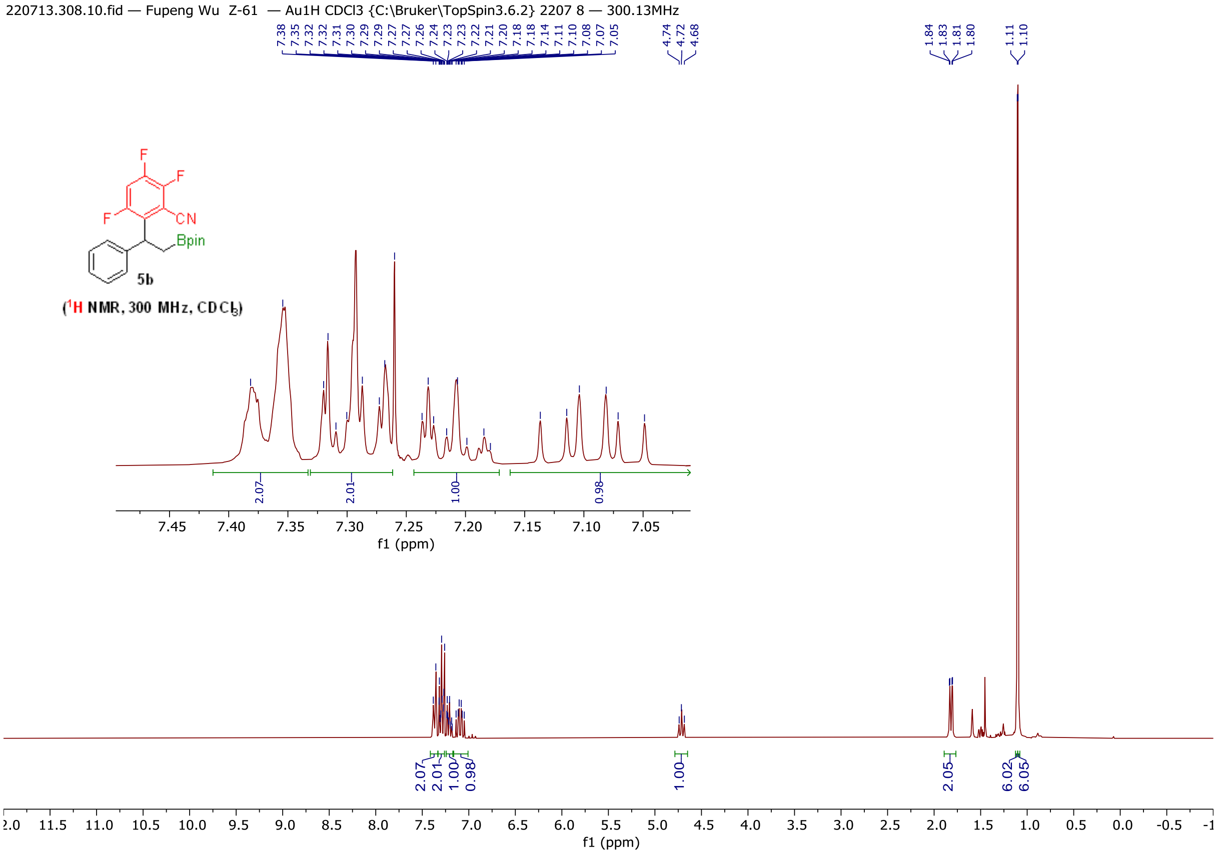

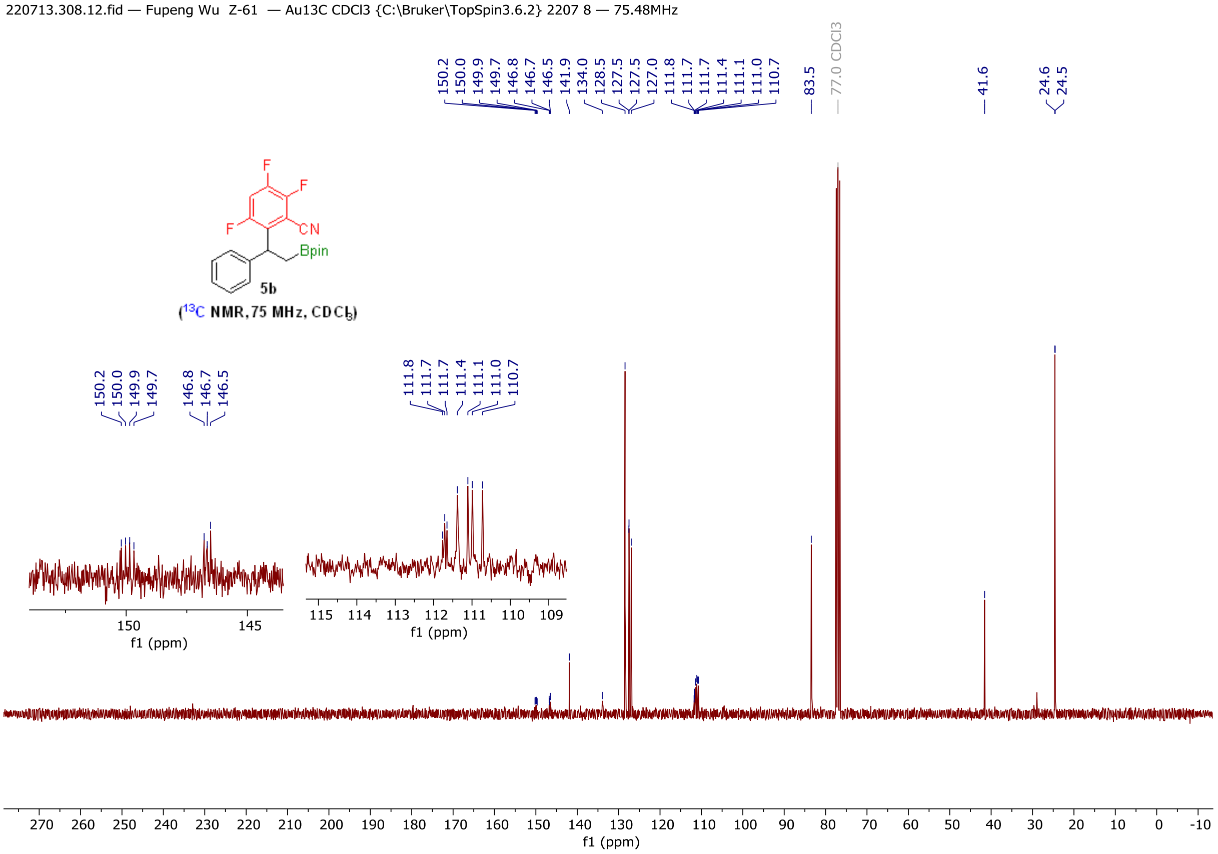

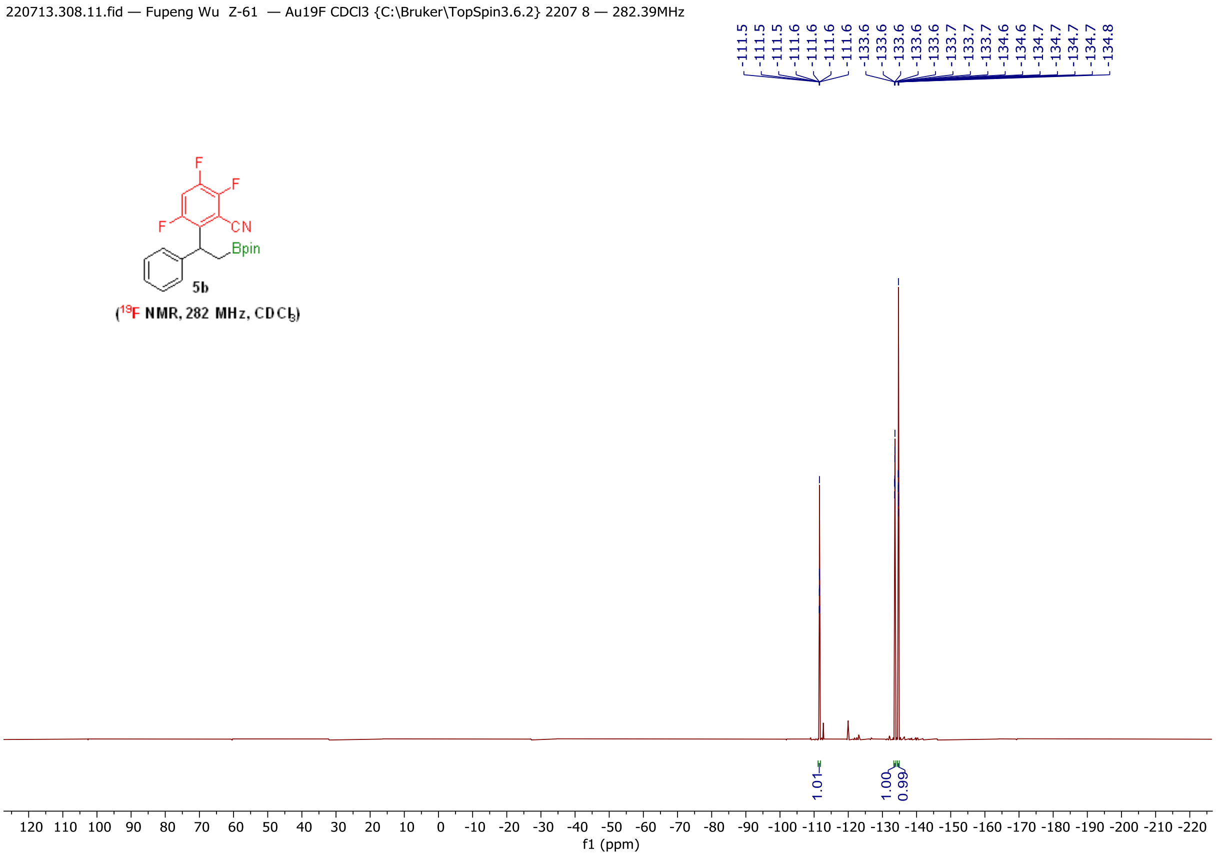
220713.308.11.fid — Fupeng Wu Z-61 — Au19F CDCl3 {C:\Bruker\TopSpin3.6.2} 2207 8 — 282.39MHz
-111.5
-111.5
-111.5
-111.6
-111.6
-111.6
-111.6
-133.6
-133.6
-133.6
-133.6
-133.6
-133.7
-133.7
-133.7
-134.6
-134.6
-134.7
-134.7
-134.7
-134.7
-134.8
F
F
F
CN
Bpin
5b
(19F NMR, 282 MHz, CDCl3)
1.01
1.00
0.99
120 110 100 90 80 70 60 50 40 30 20 10 0 -10 -20 -30 -40 -50 -60 -70 -80 -90 -100 -110 -120 -130 -140 -150 -160 -170 -180 -190 -200 -210 -220
f1 (ppm)

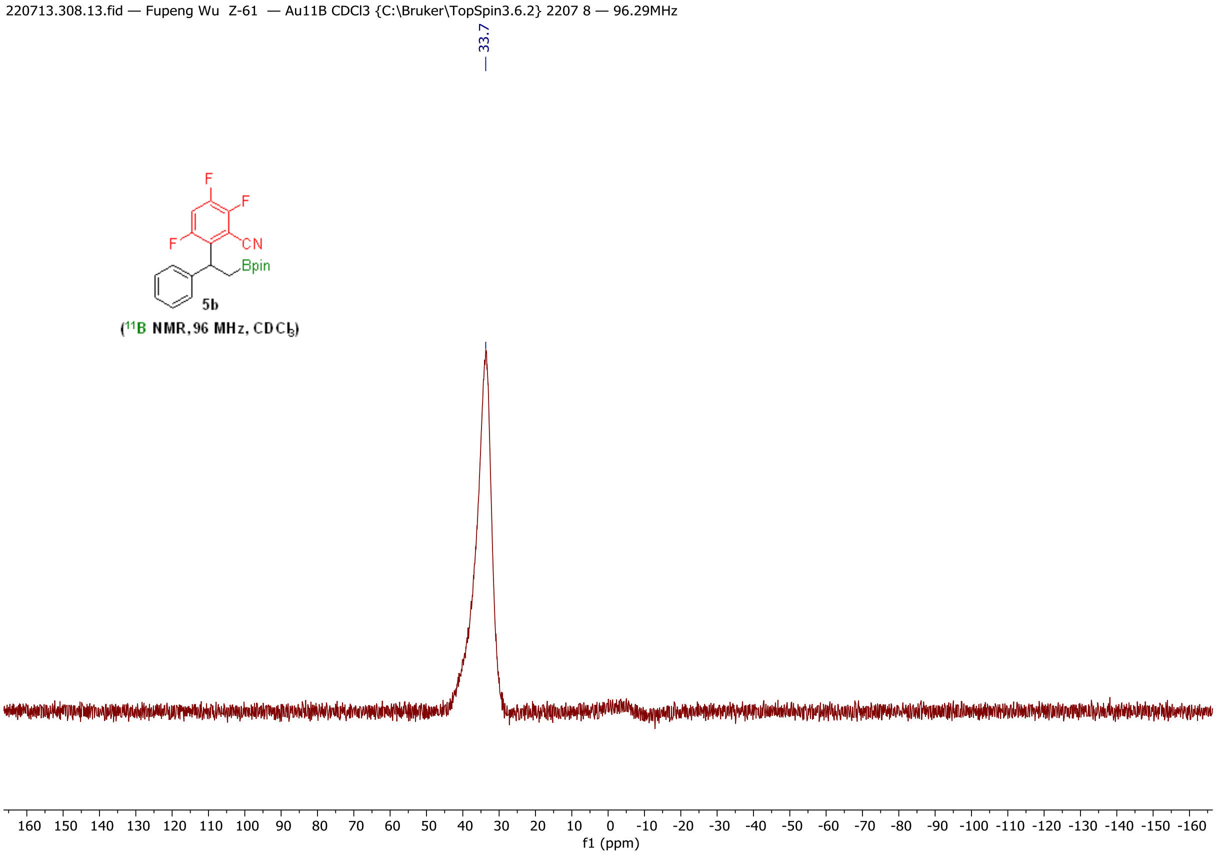
220713.308.13.fid — Fupeng Wu Z-61 — Au11B CDCl3 {C:\Bruker\TopSpin3.6.2} 2207 8 — 96.29MHz
33.7
F
F
F
CN
Bpin
5b
(11B NMR, 96 MHz, CDCl3)
160 150 140 130 120 110 100 90 80 70 60 50 40 30 20 10 0 -10 -20 -30 -40 -50 -60 -70 -80 -90 -100 -110 -120 -130 -140 -150 -160
f1 (ppm)

NMR Spectra of **5c**

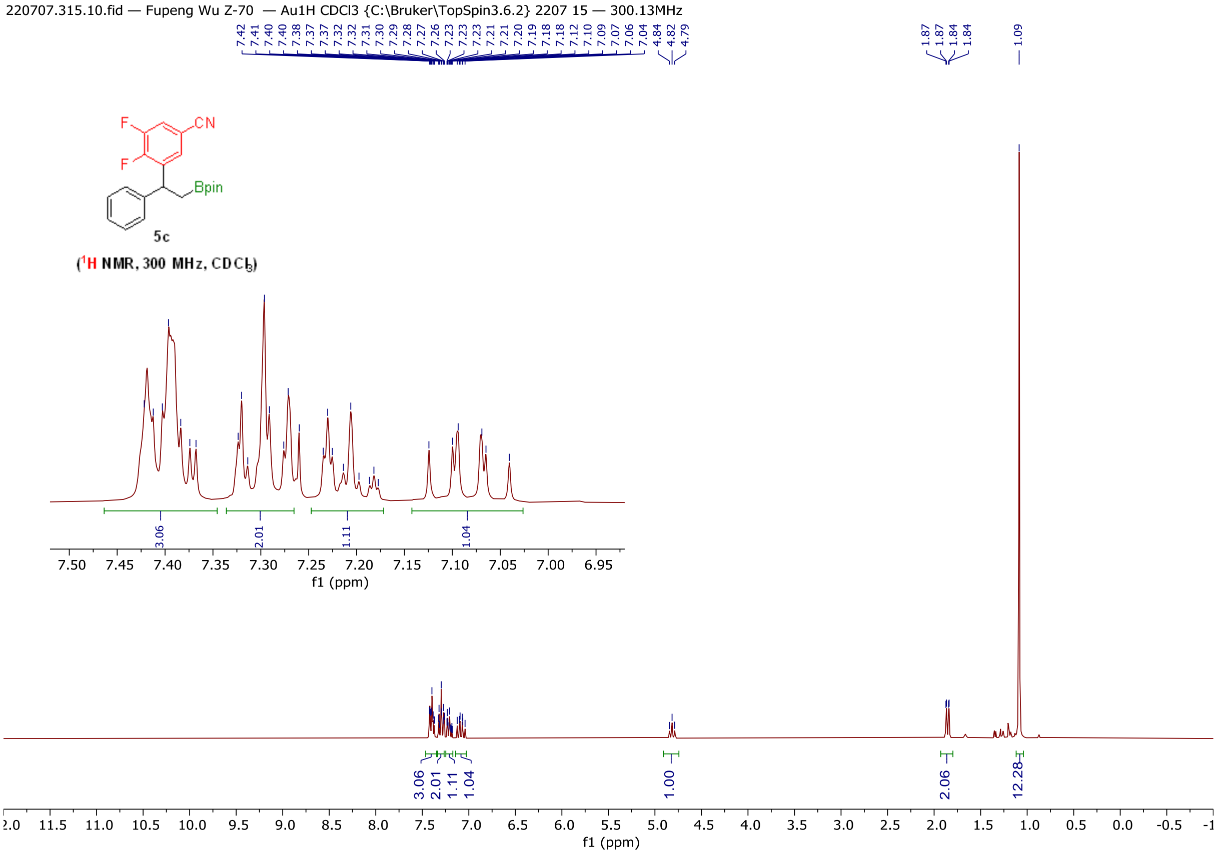

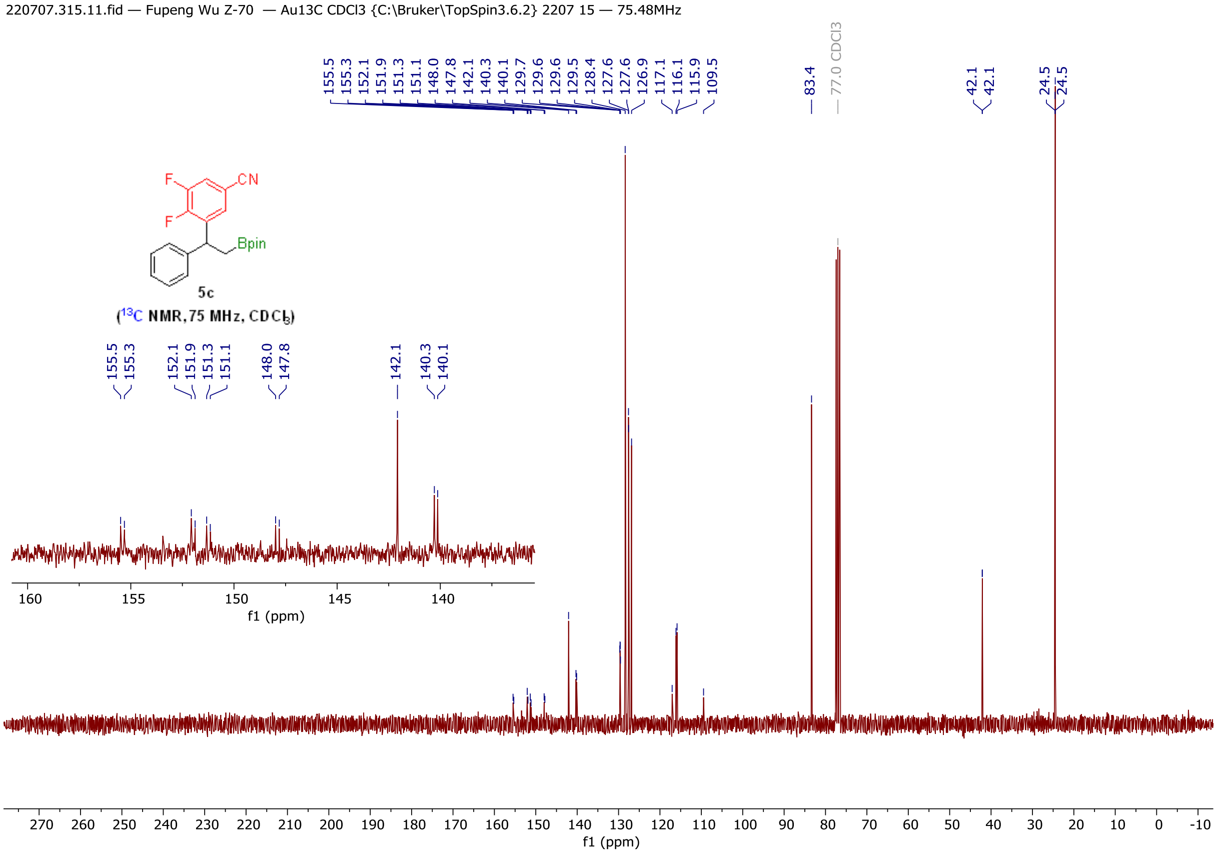

220707.315.12.fid — Fupeng Wu Z-70 — Au19F CDCl3 {C:\Bruker\TopSpin3.6.2} 2207 15 — 282.39MHz

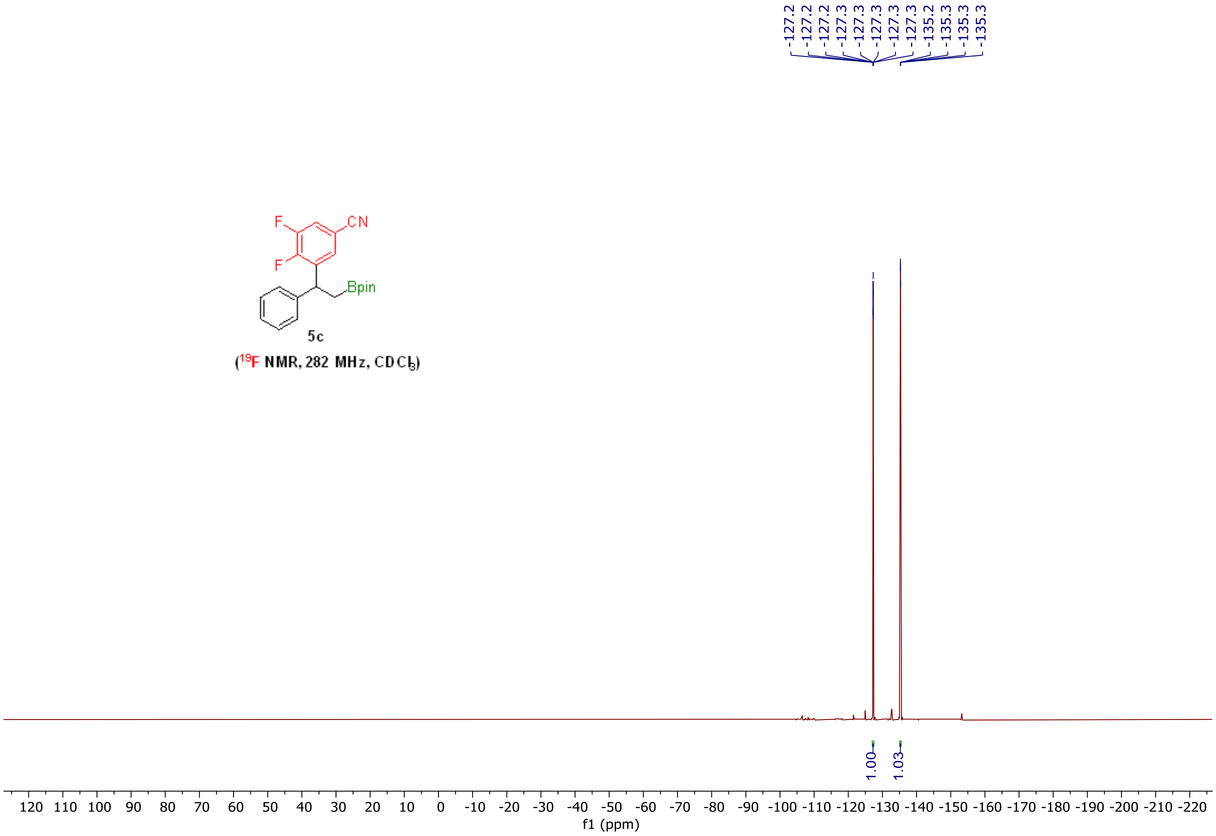

220707.315.13.fid — Fupeng Wu Z-70 — Au11B CDCl3 {C:\Bruker\TopSpin3.6.2} 2207 15 — 96.29MHz

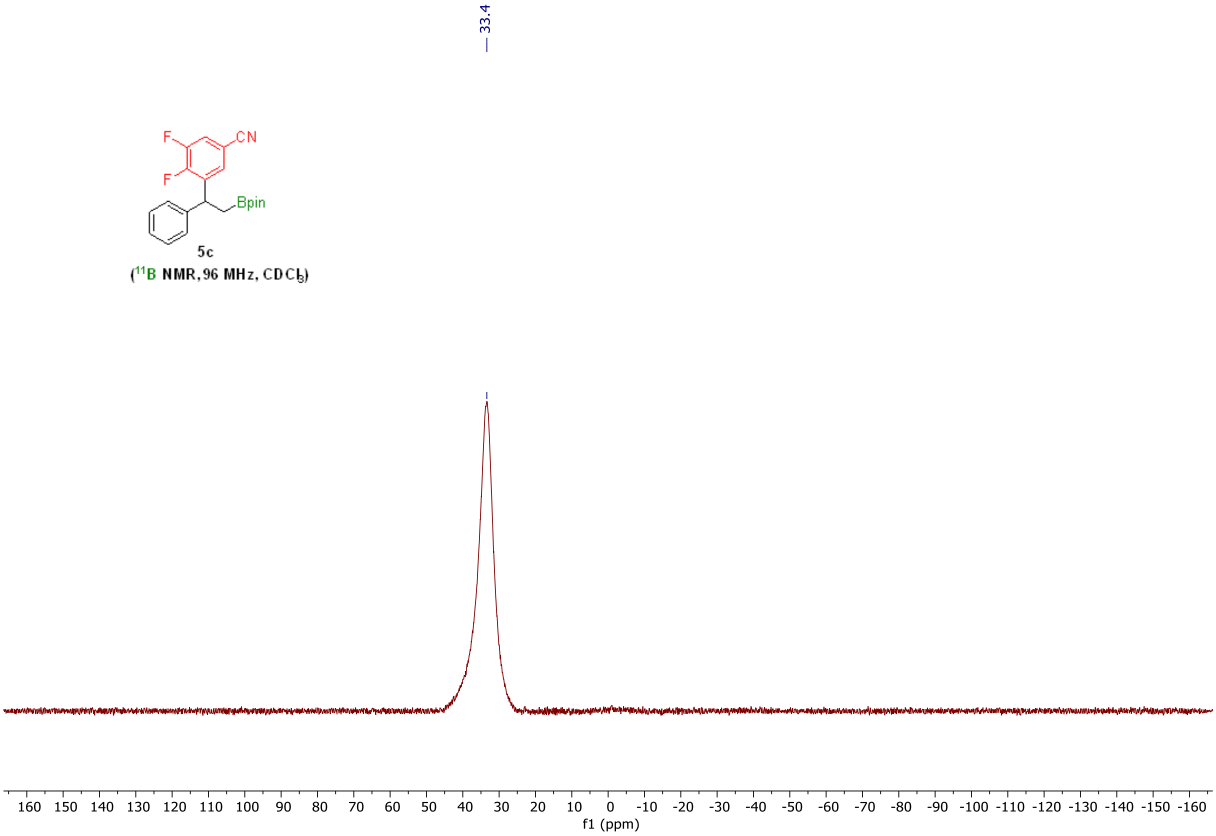

NMR Spectra of **5e**

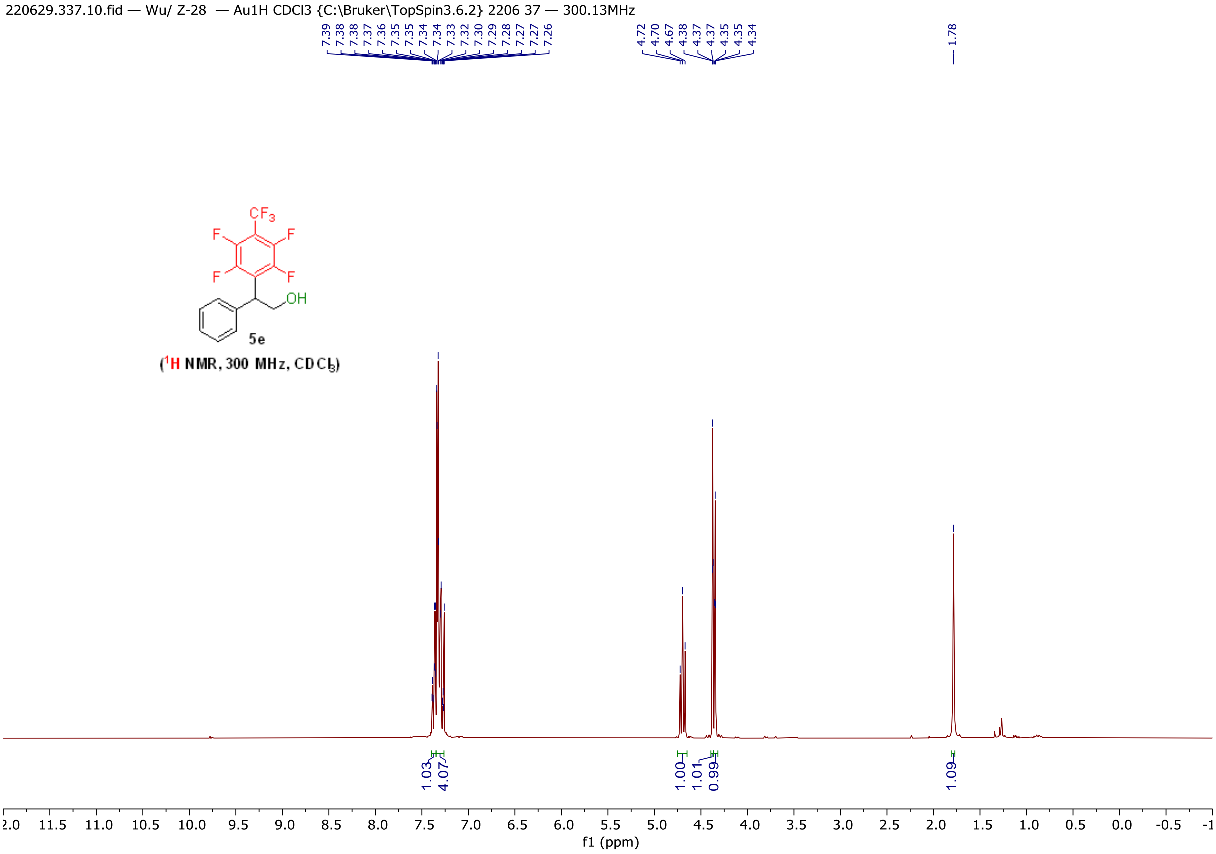

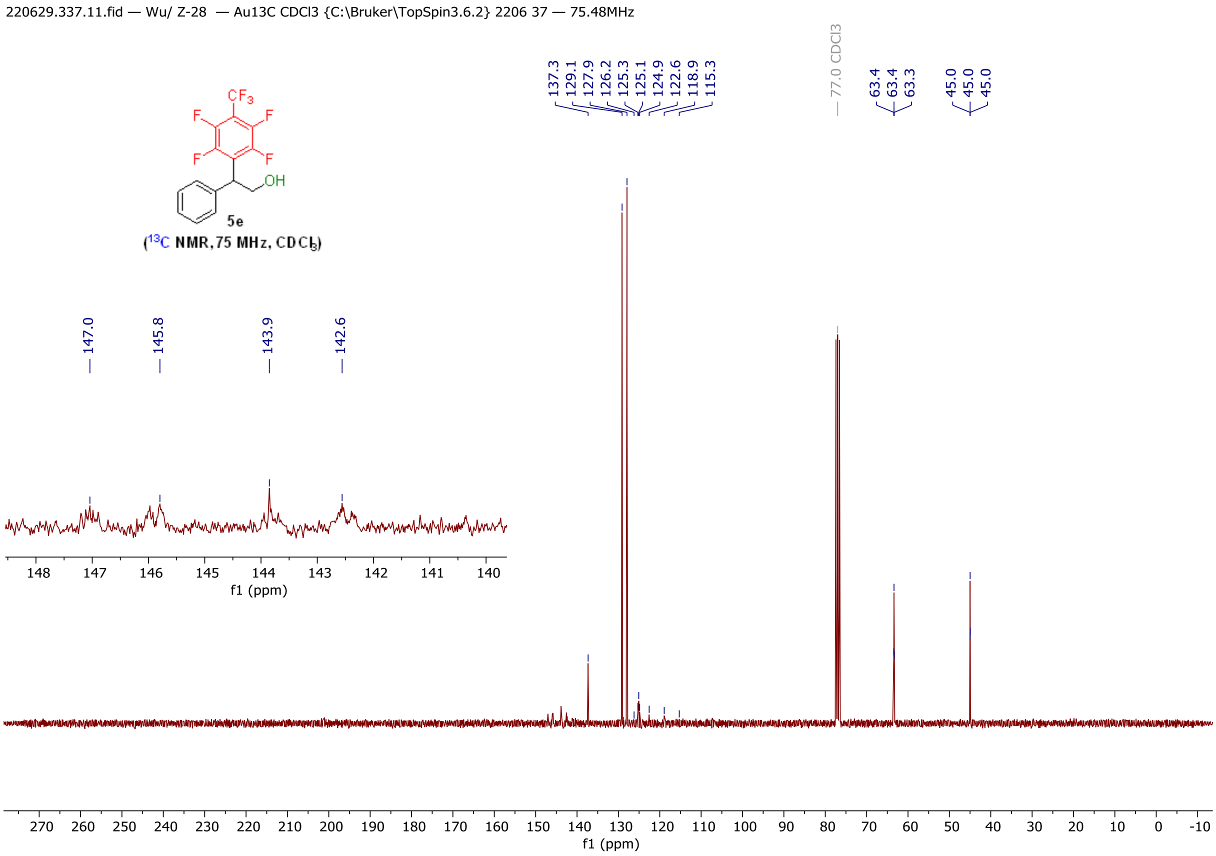

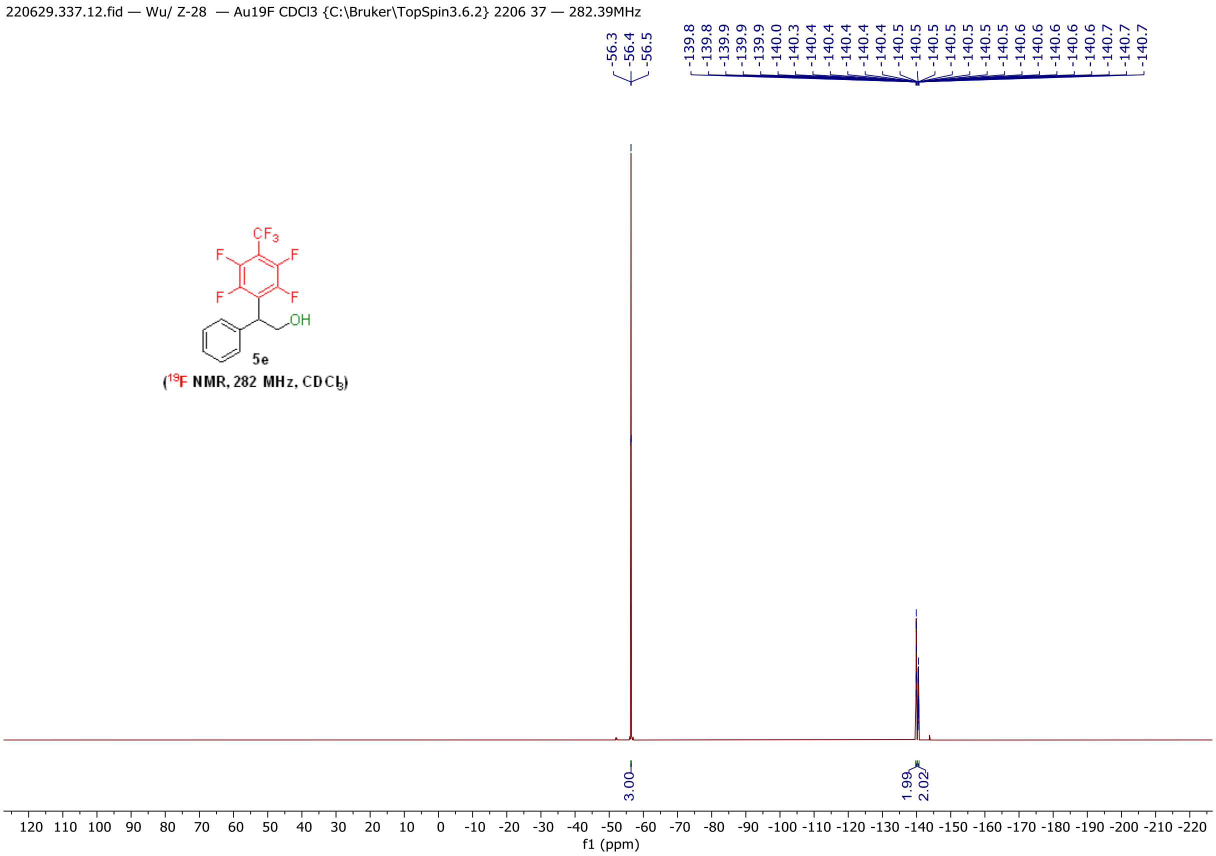
220629.337.12.fid — Wu/ Z-28 — Au19F CDCl3 {C:\Bruker\TopSpin3.6.2} 2206 37 — 282.39MHz
-56.3
-56.4
-56.5
-139.8
-139.8
-139.9
-139.9
-139.9
-140.0
-140.3
-140.4
-140.4
-140.4
-140.4
-140.4
-140.5
-140.5
-140.5
-140.5
-140.5
-140.5
-140.6
-140.6
-140.6
-140.6
-140.7
-140.7
CF3
F
F
F
F
OH
5e
(19F NMR, 282 MHz, CDCl3)
3.00
1.99
2.02
120 110 100 90 80 70 60 50 40 30 20 10 0 -10 -20 -30 -40 -50 -60 -70 -80 -90 -100 -110 -120 -130 -140 -150 -160 -170 -180 -190 -200 -210 -220
f1 (ppm)

NMR Spectra of **5f**

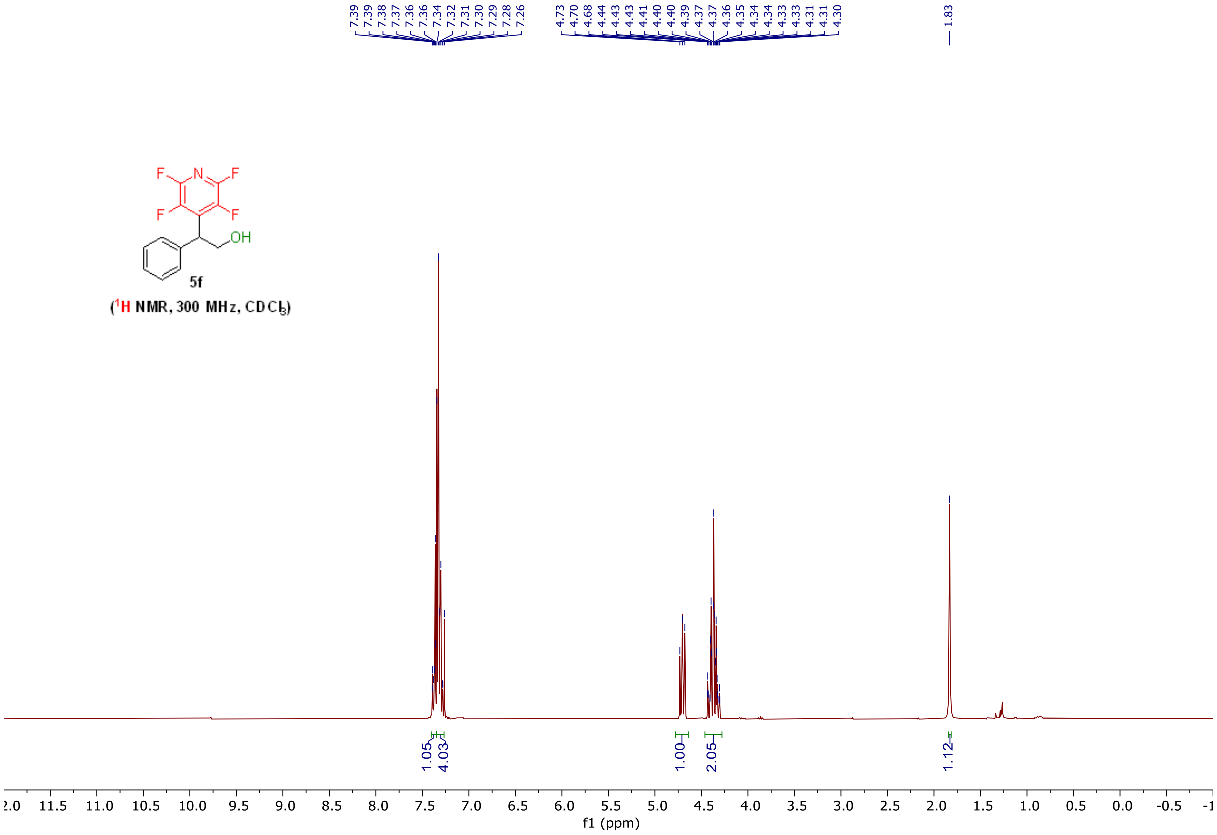

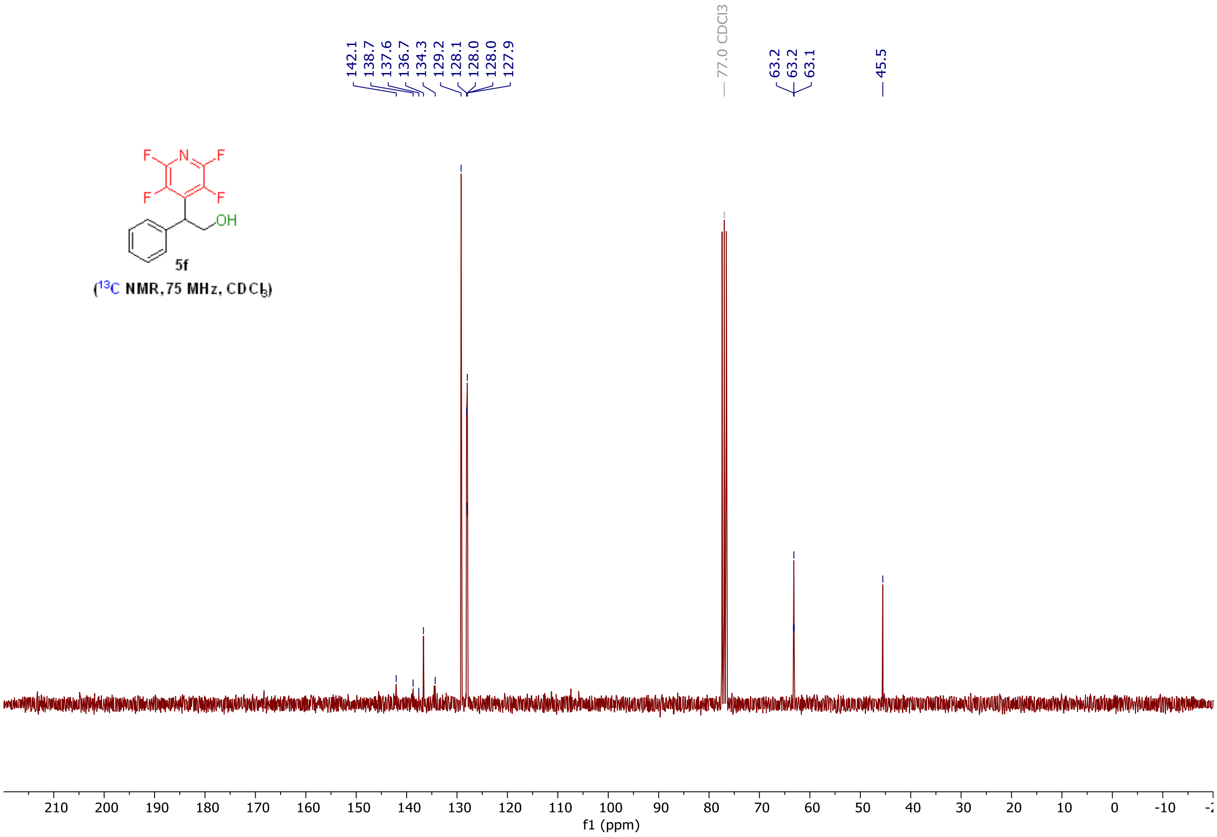

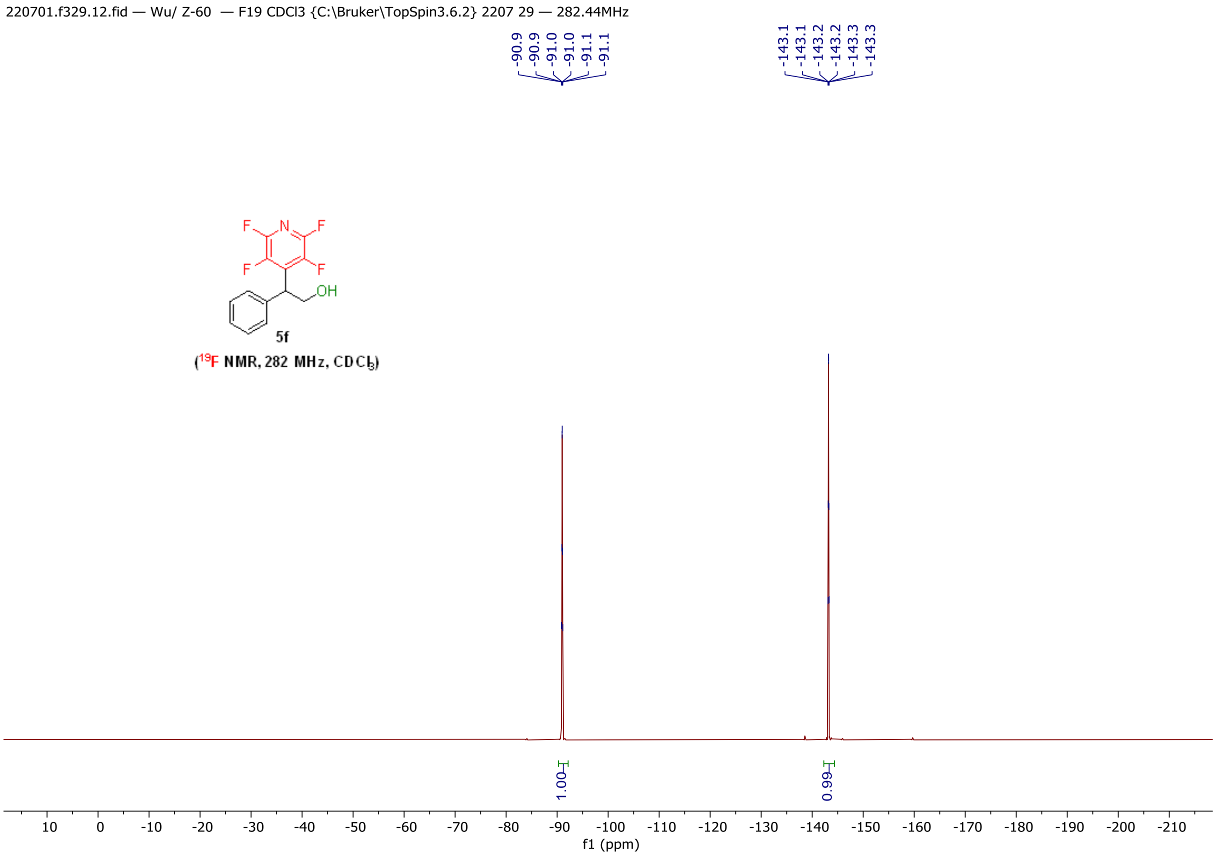
220701.f329.12.fid — Wu/ Z-60 — F19 CDCl3 {C:\Bruker\TopSpin3.6.2} 2207 29 — 282.44MHz
-90.9
-90.9
-91.0
-91.0
-91.1
-91.1
-143.1
-143.1
-143.2
-143.2
-143.3
-143.3
5f
(19F NMR, 282 MHz, CDCl3)
1.00
0.99
10 0 -10 -20 -30 -40 -50 -60 -70 -80 -90 -100 -110 -120 -130 -140 -150 -160 -170 -180 -190 -200 -210
f1 (ppm)

NMR Spectra of **6a**

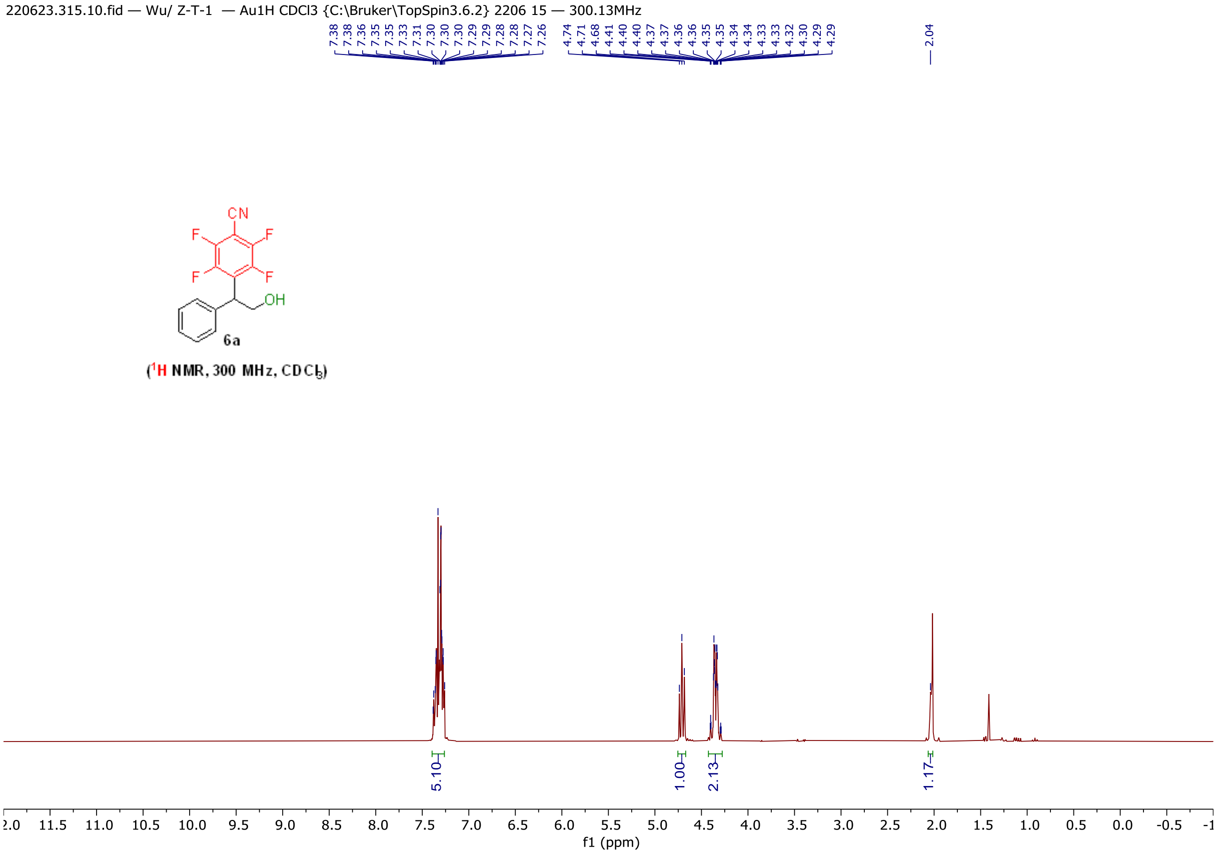

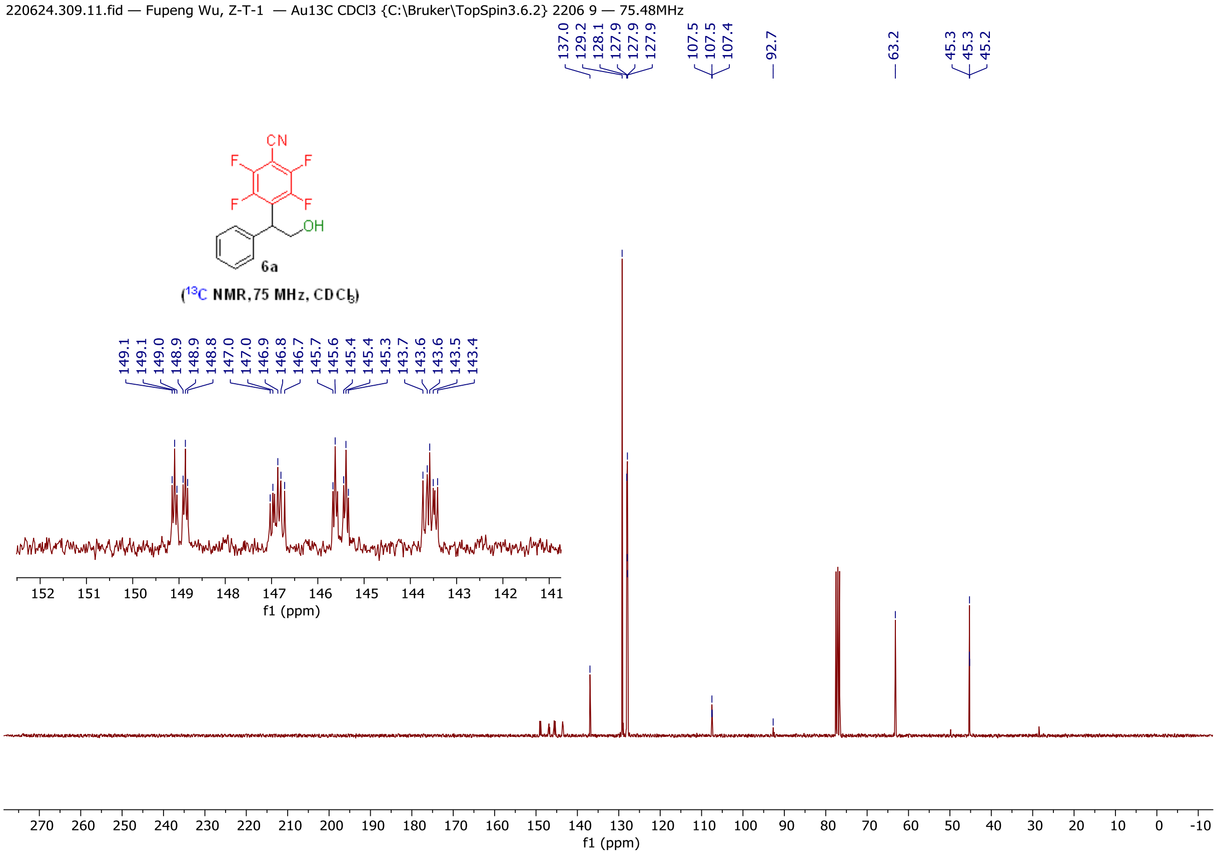

220624.309.12.fid — Fupeng Wu, Z-T-1 — Au19F CDCl3 {C:\Bruker\TopSpin3.6.2} 2206 9 — 282.39MHz

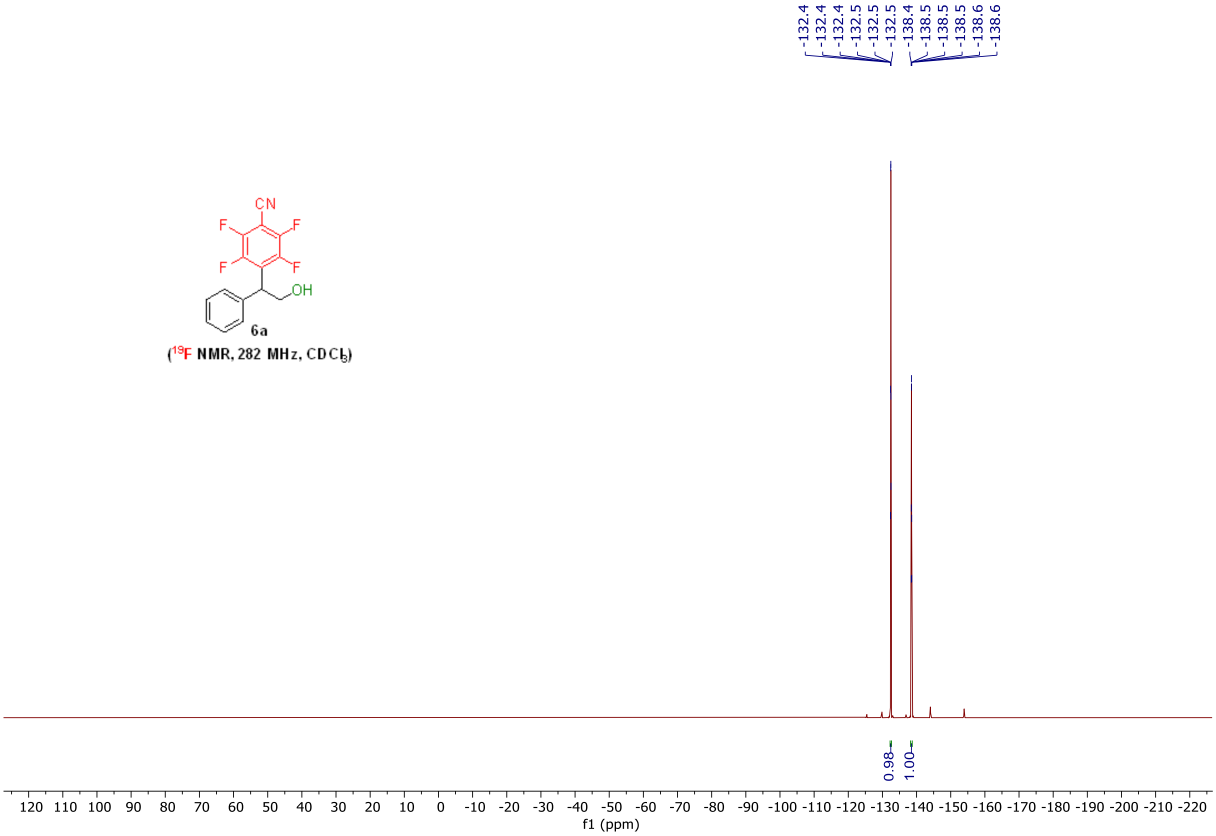

NMR Spectra of **6b**

220715.f316.10.fid — Fupeng Wu Z-T-5 — PROTON DMSO {C:\Bruker\TopSpin3.6.2} 2207 16 — 300.20MHz

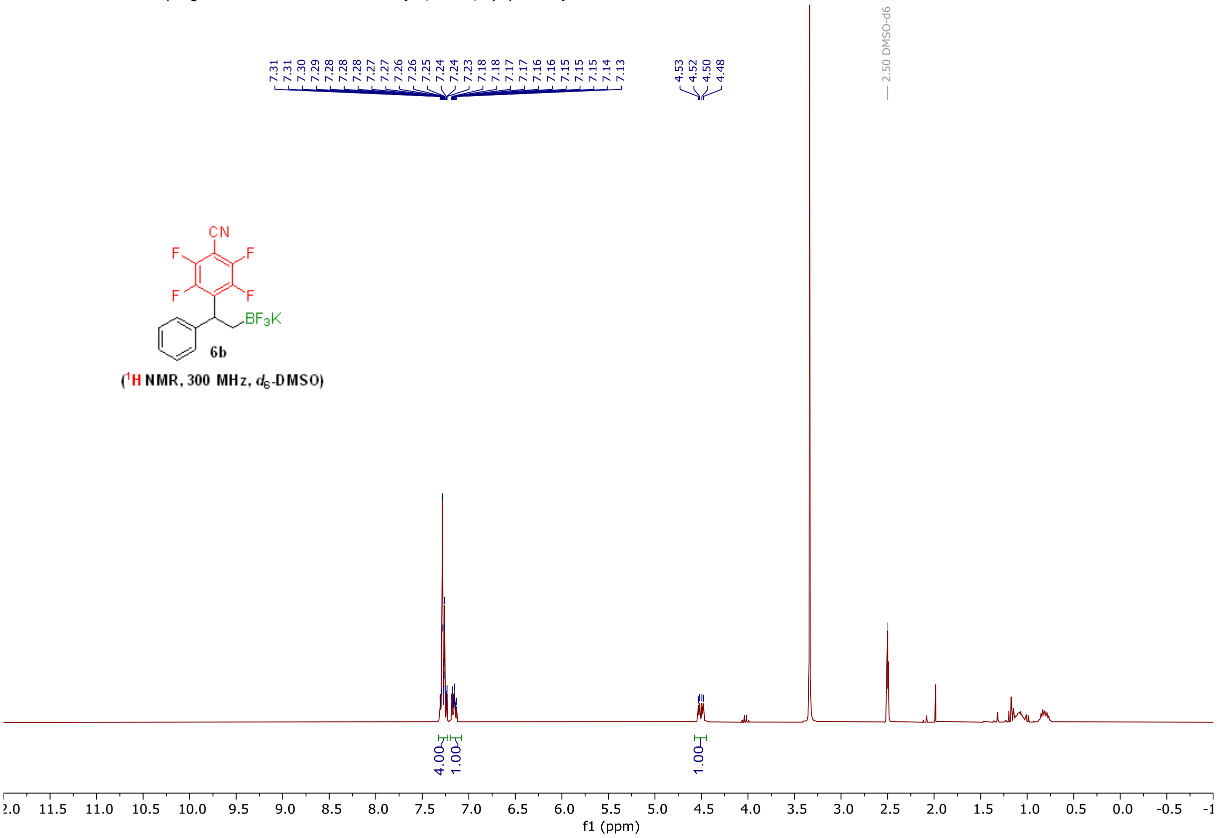

220715.f316.11.fid — Fupeng Wu Z-T-5 — C13CPD DMSO {C:\Bruker\TopSpin3.6.2} 2207 16 — 75.49MHz

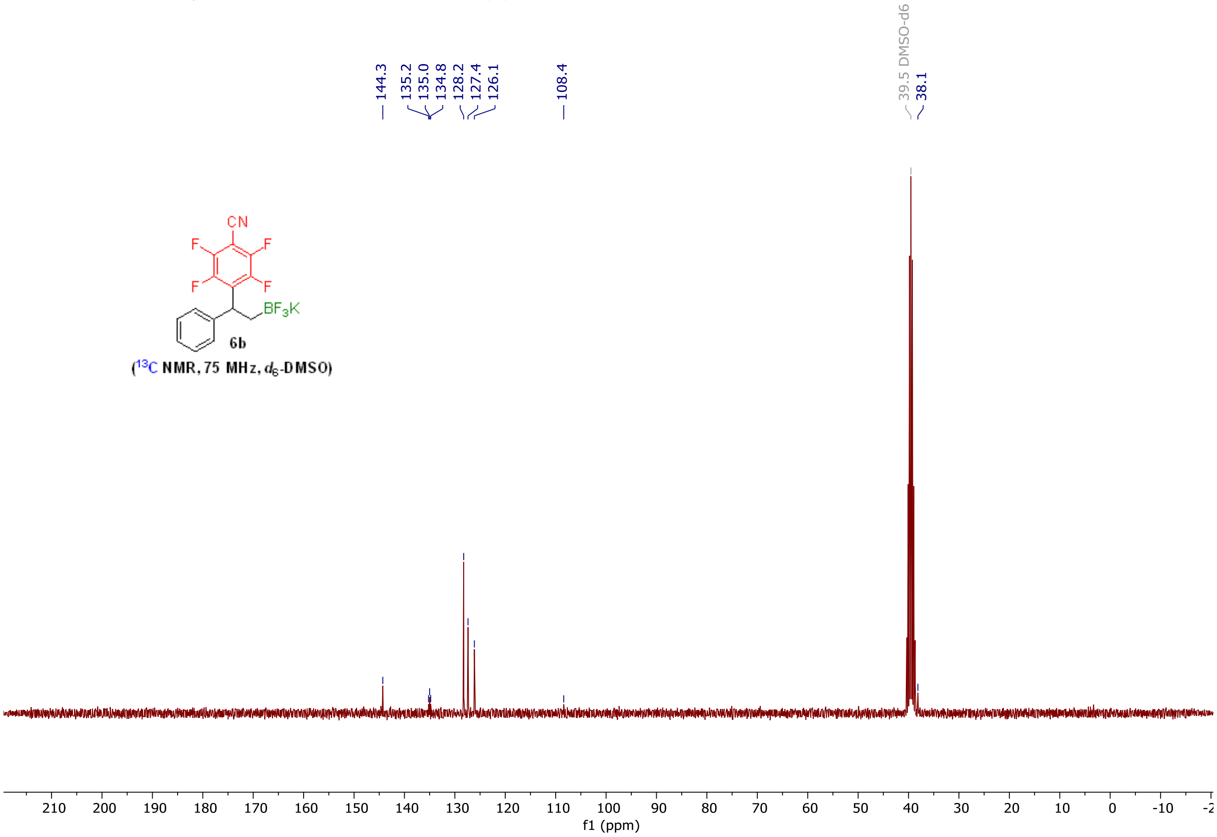

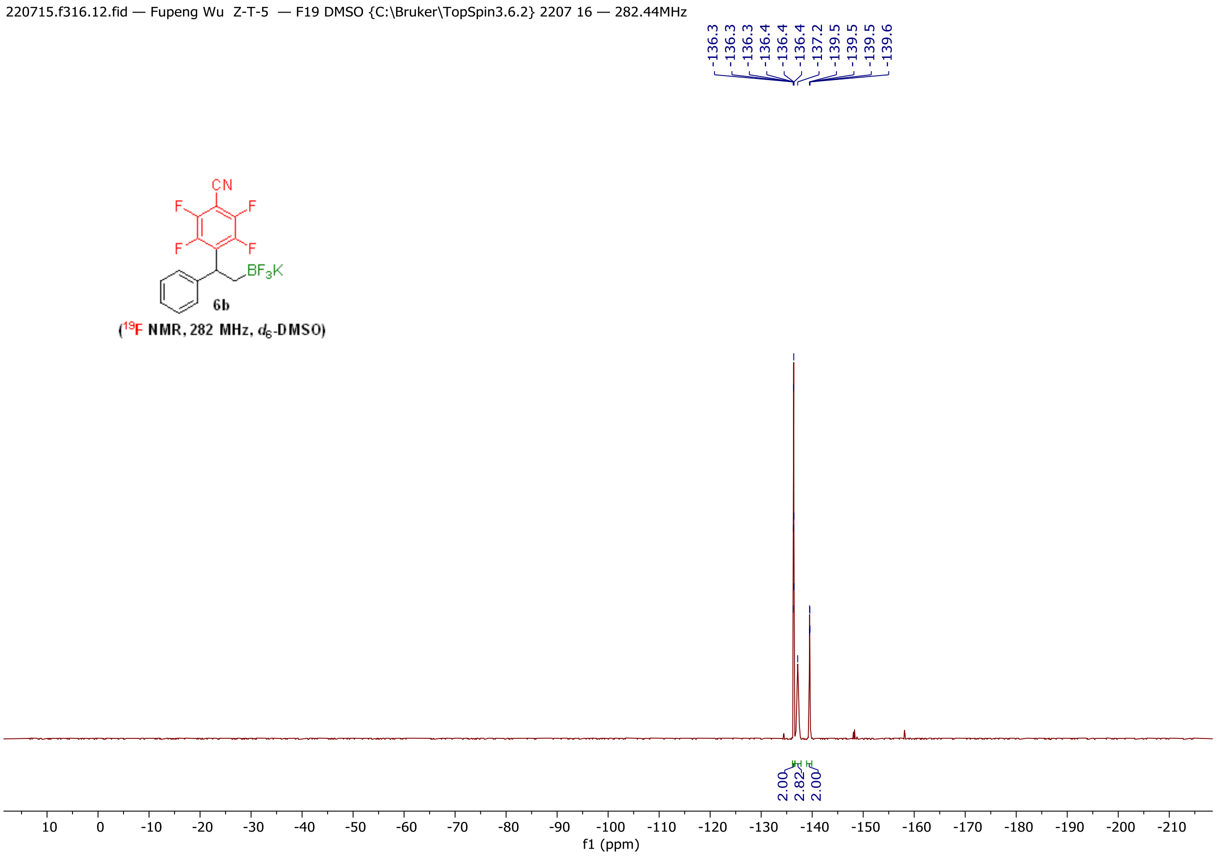
220715.f316.12.fid — Fupeng Wu Z-T-5 — F19 DMSO {C:\Bruker\TopSpin3.6.2} 2207 16 — 282.44MHz
-136.3
-136.3
-136.3
-136.4
-136.4
-136.4
-137.2
-139.5
-139.5
-139.5
-139.6
CN
F
F
F
F
BF3K
6b
(19F NMR, 282 MHz, d6-DMSO)
2.00
2.82
2.00
10 0 -10 -20 -30 -40 -50 -60 -70 -80 -90 -100 -110 -120 -130 -140 -150 -160 -170 -180 -190 -200 -210
f1 (ppm)

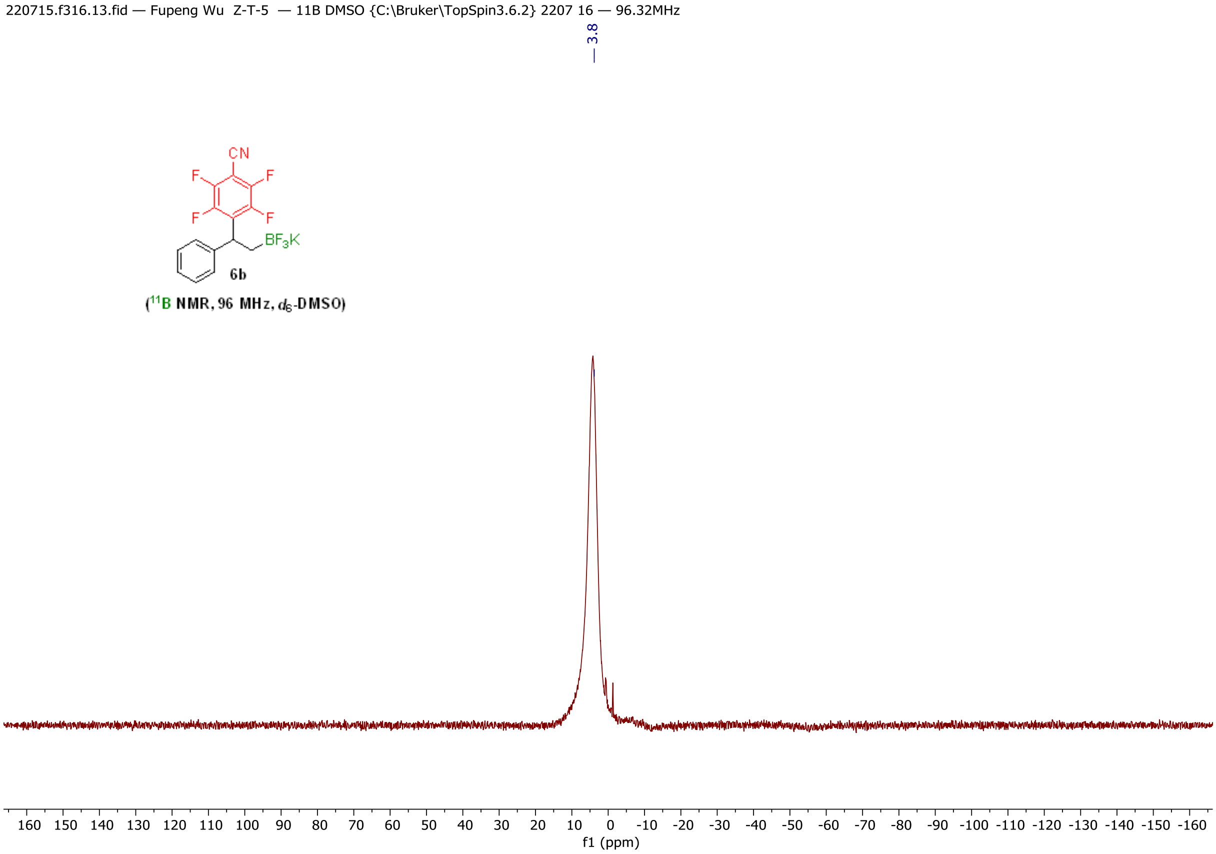
220715.f316.13.fid — Fupeng Wu Z-T-5 — 11B DMSO {C:\Bruker\TopSpin3.6.2} 2207 16 — 96.32MHz
3.8
CN
F
F
F
F
BF3K
6b
(11B NMR, 96 MHz, d6-DMSO)
160 150 140 130 120 110 100 90 80 70 60 50 40 30 20 10 0 -10 -20 -30 -40 -50 -60 -70 -80 -90 -100 -110 -120 -130 -140 -150 -160
f1 (ppm)

NMR Spectra of **6c**

220804.304.10.fid — Fupeng Wu Z-T-11 — Au1H CDCl3 {C:\Bruker\TopSpin3.6.2} 2208 4 — 300.13MHz

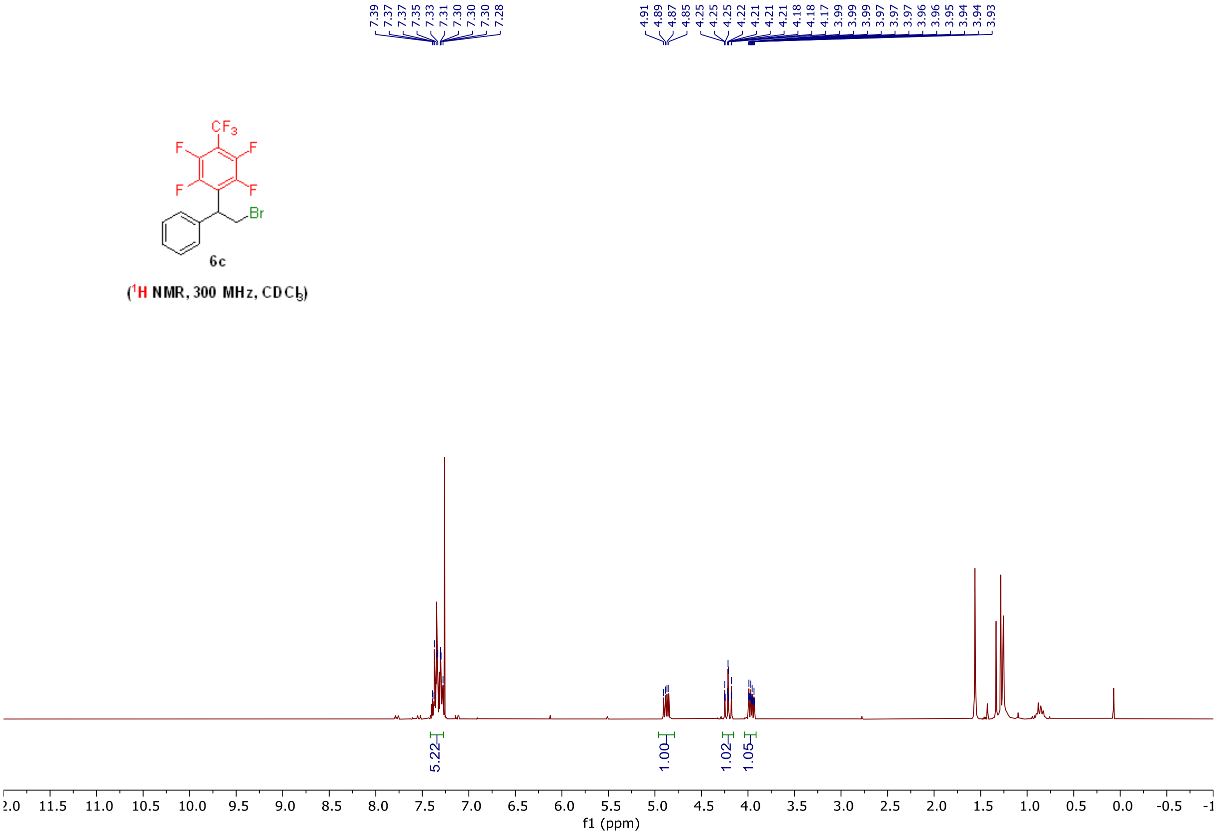

6c

(^{1}H NMR, 300 MHz, CDCl$_3$)

220804.304.11.fid — Fupeng Wu Z-T-11 — Au13C CDCl3 {C:\Bruker\TopSpin3.6.2} 2208 4 — 75.48MHz

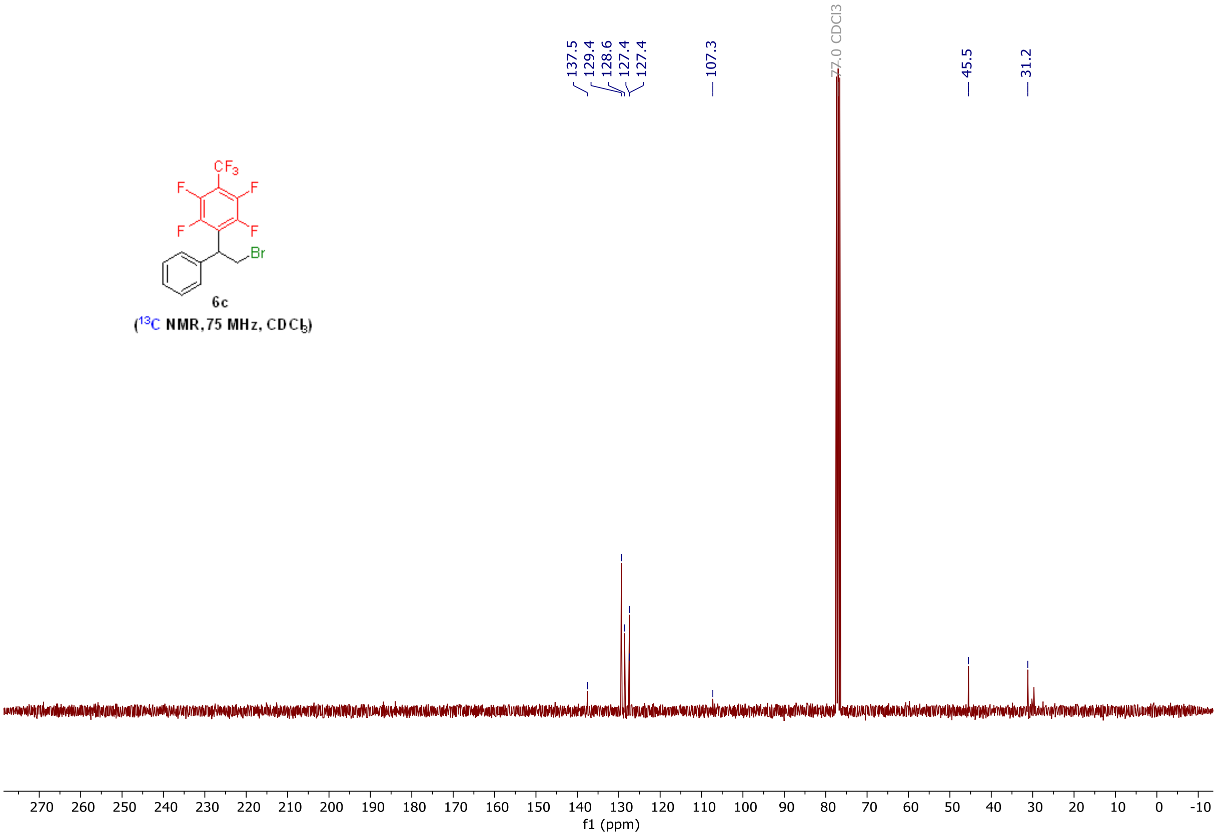

6c

(^{13}C NMR, 75 MHz, CDCl$_3$)

220804.304.12.fid — Fupeng Wu Z-T-11 — Au19F CDCl3 {C:\Bruker\TopSpin3.6.2} 2208 4 — 282.39MHz

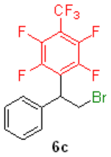

6c

(19F NMR, 282 MHz, CDCl3)

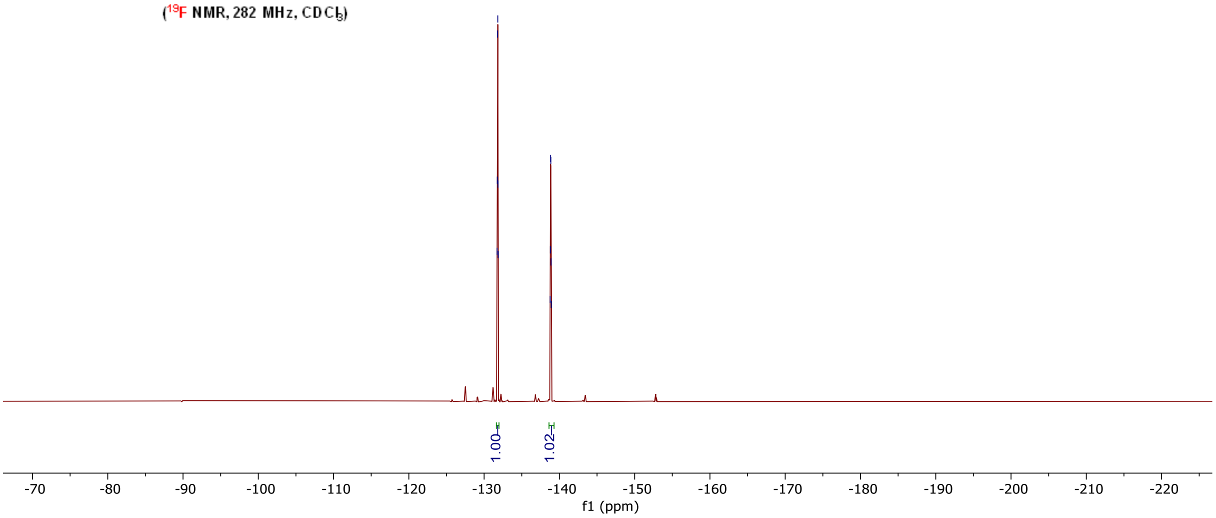

NMR Spectra of **6d**

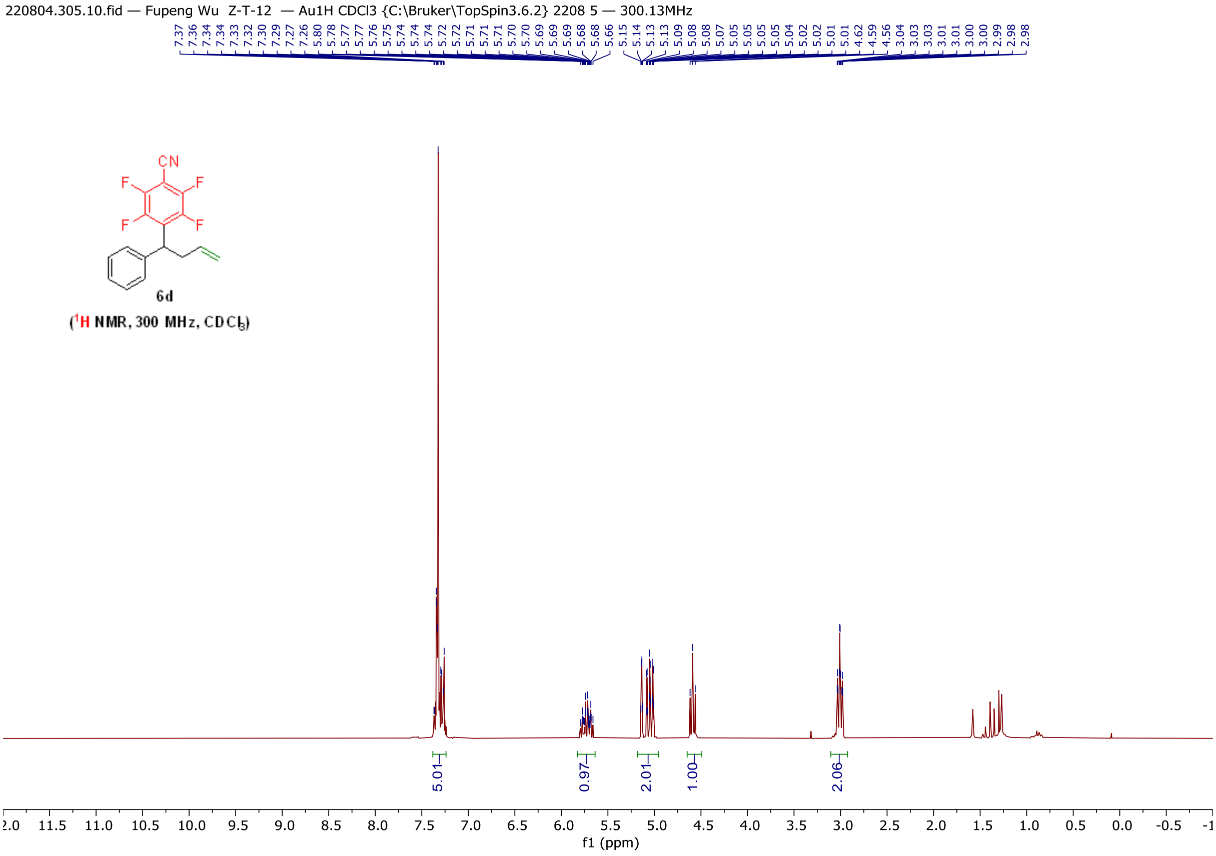

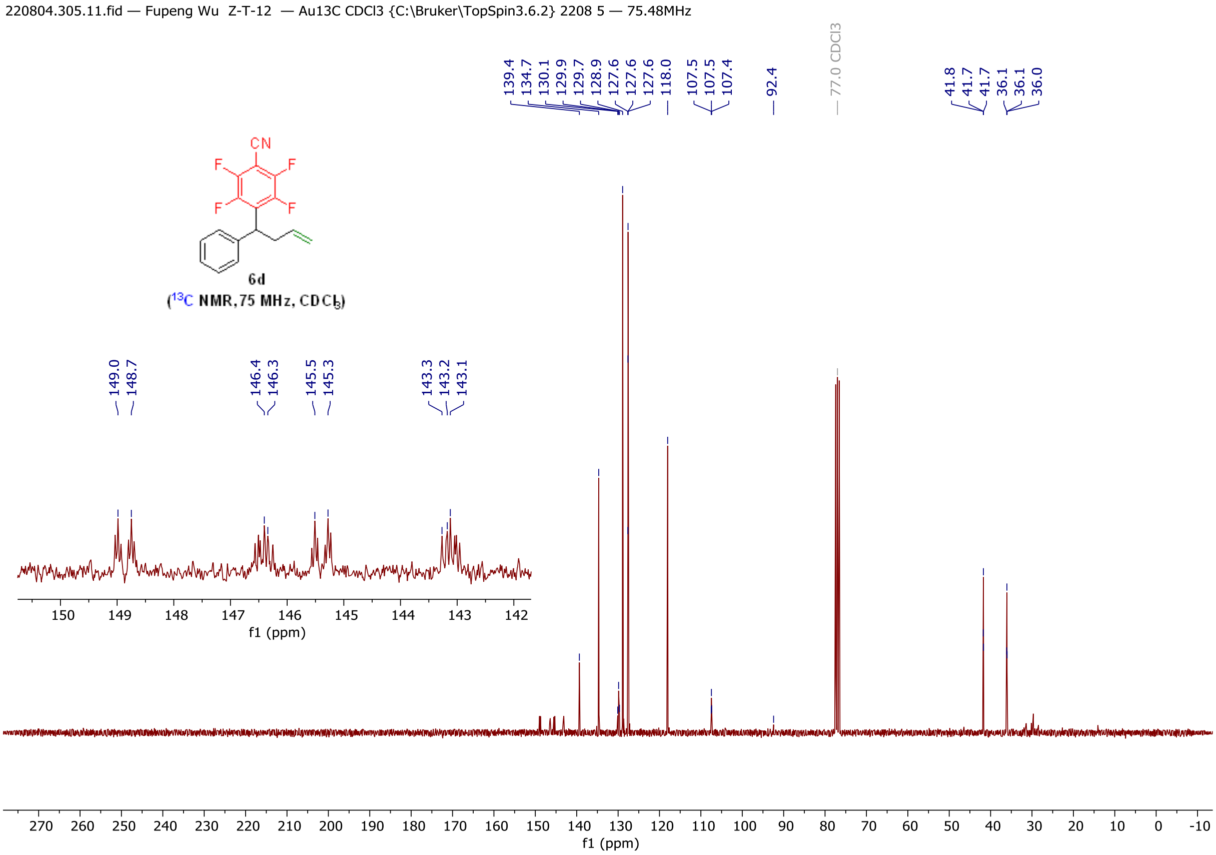

220804.305.12.fid — Fupeng Wu Z-T-12 — Au19F CDCl3 {C:\Bruker\TopSpin3.6.2} 2208 5 — 282.39MHz

6d

(^{19}F NMR, 282 MHz, $CDCl_3$)

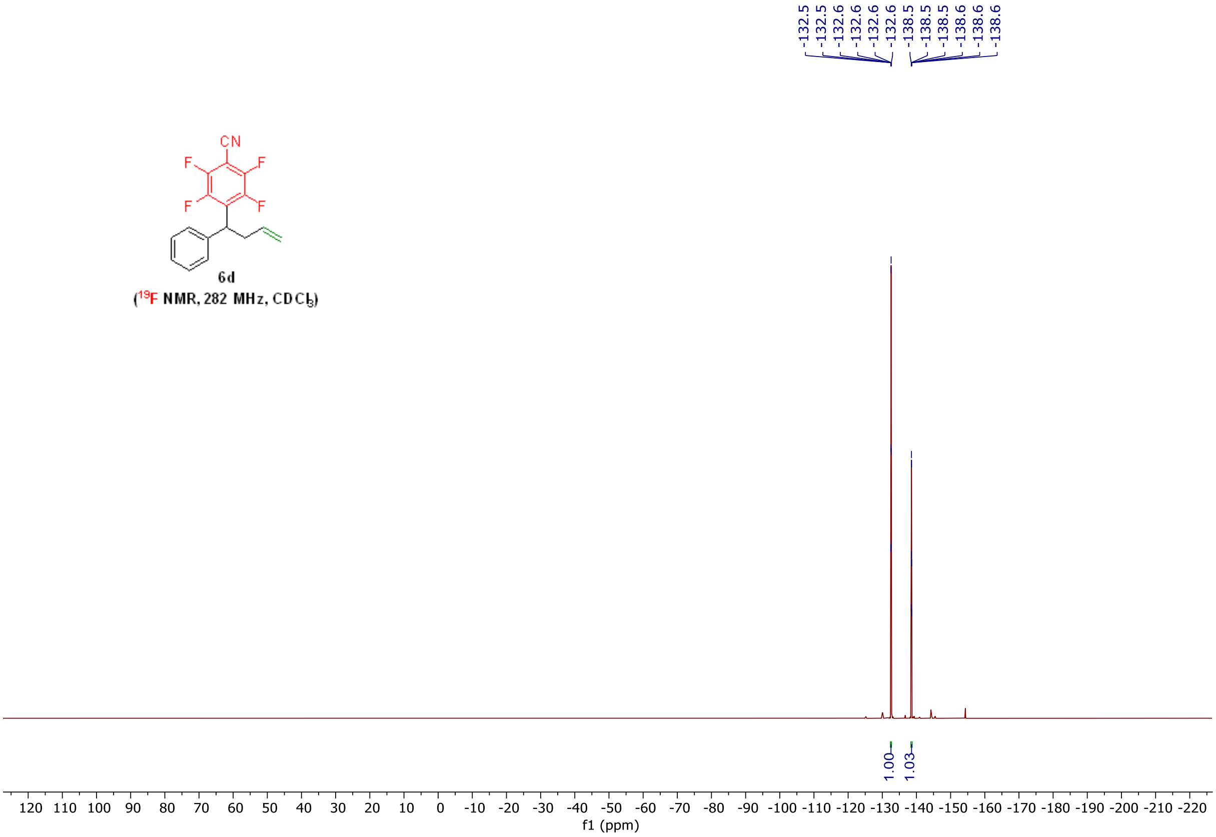

NMR Spectra of **3c'-alcohol**

220802.317.10.fid — Fupeng Wu Z-9-0 — Au1H CDCl3 {C:\Bruker\TopSpin3.6.2} 2208 17 — 300.13MHz

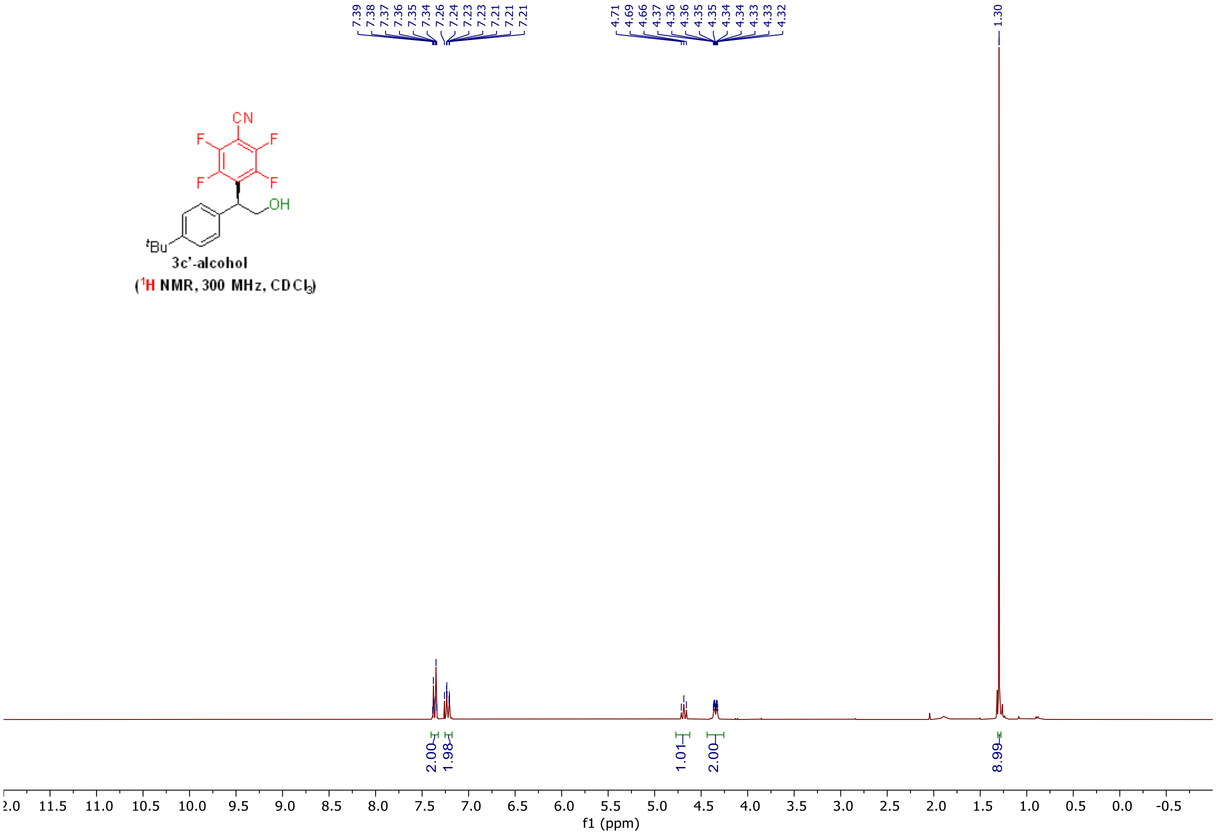

220802.317.11.fid — Fupeng Wu Z-9-0 — Au13C CDCl3 {C:\Bruker\TopSpin3.6.2} 2208 17 — 75.48MHz

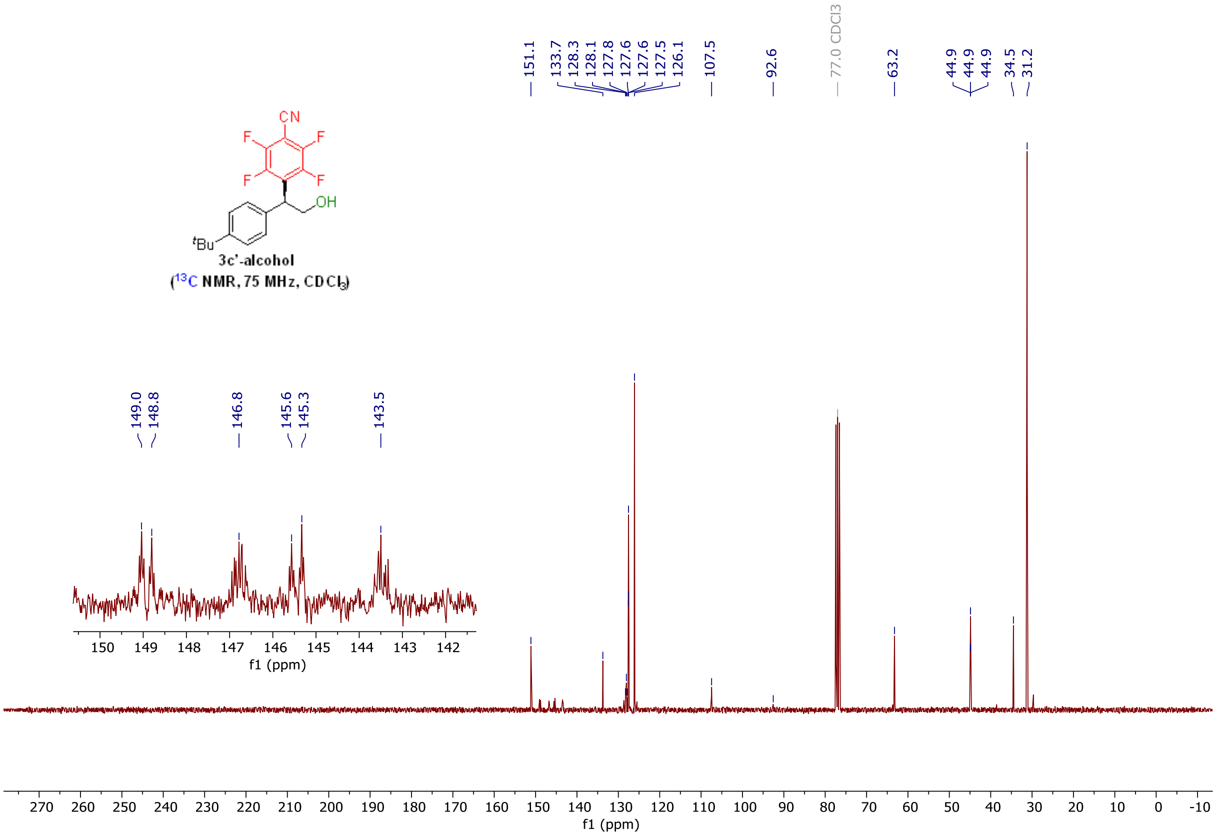

220802.317.12.fid — Fupeng Wu Z-9-0 — Au19F CDCl3 {C:\Bruker\TopSpin3.6.2} 2208 17 — 282.39MHz

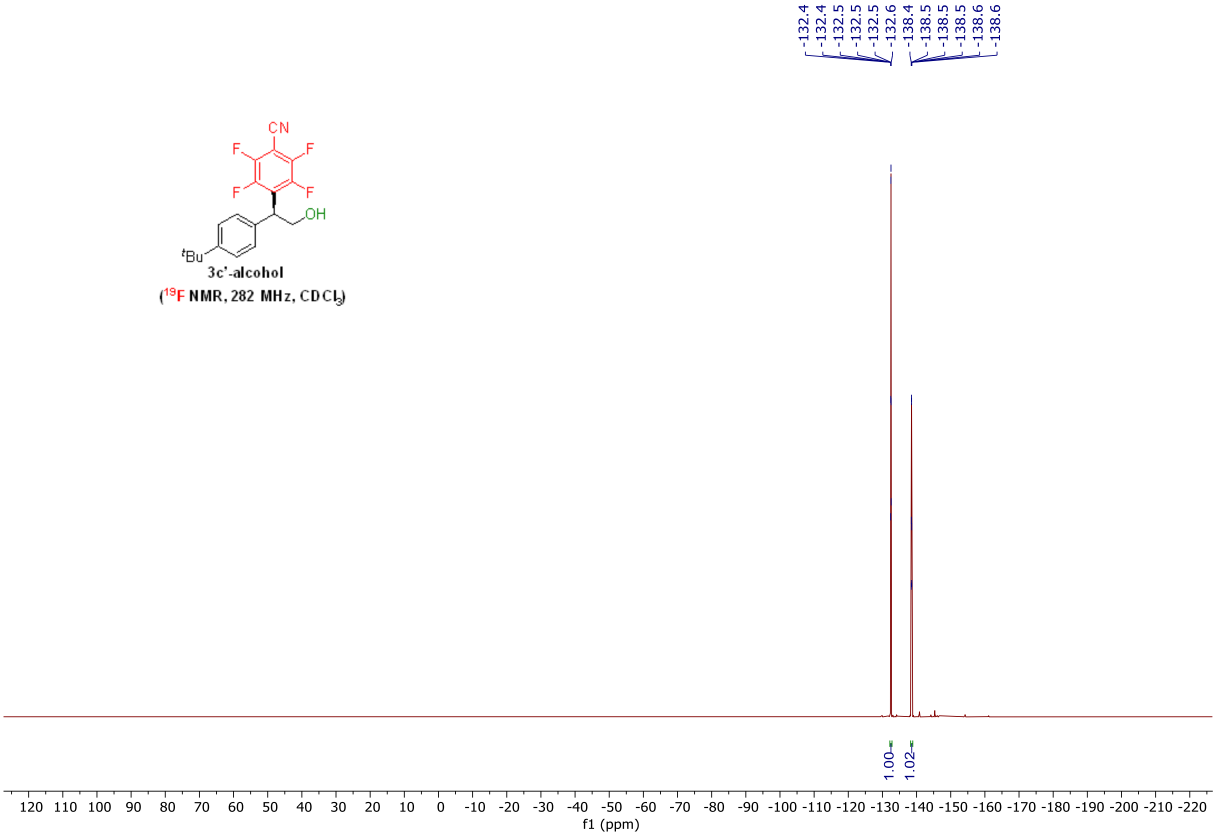

NMR Spectra of **3d'-alcohol**

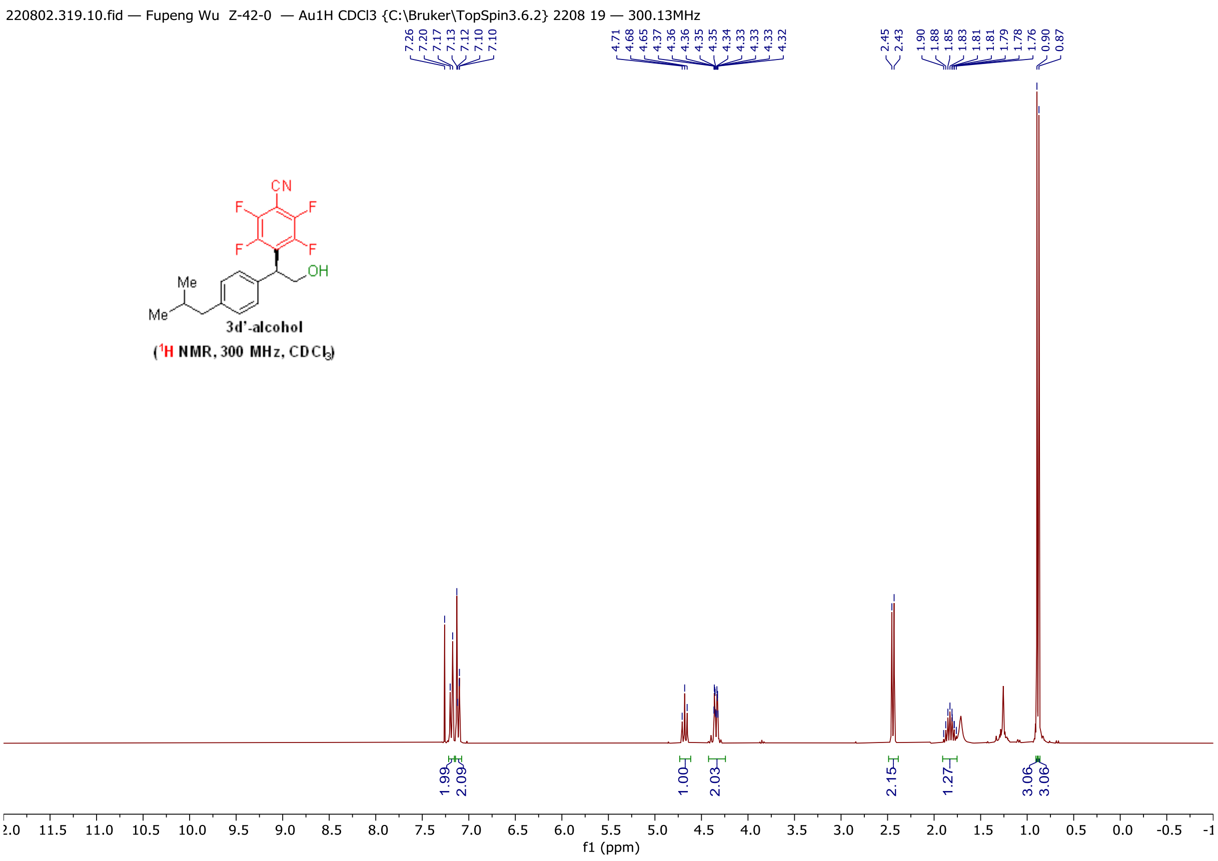

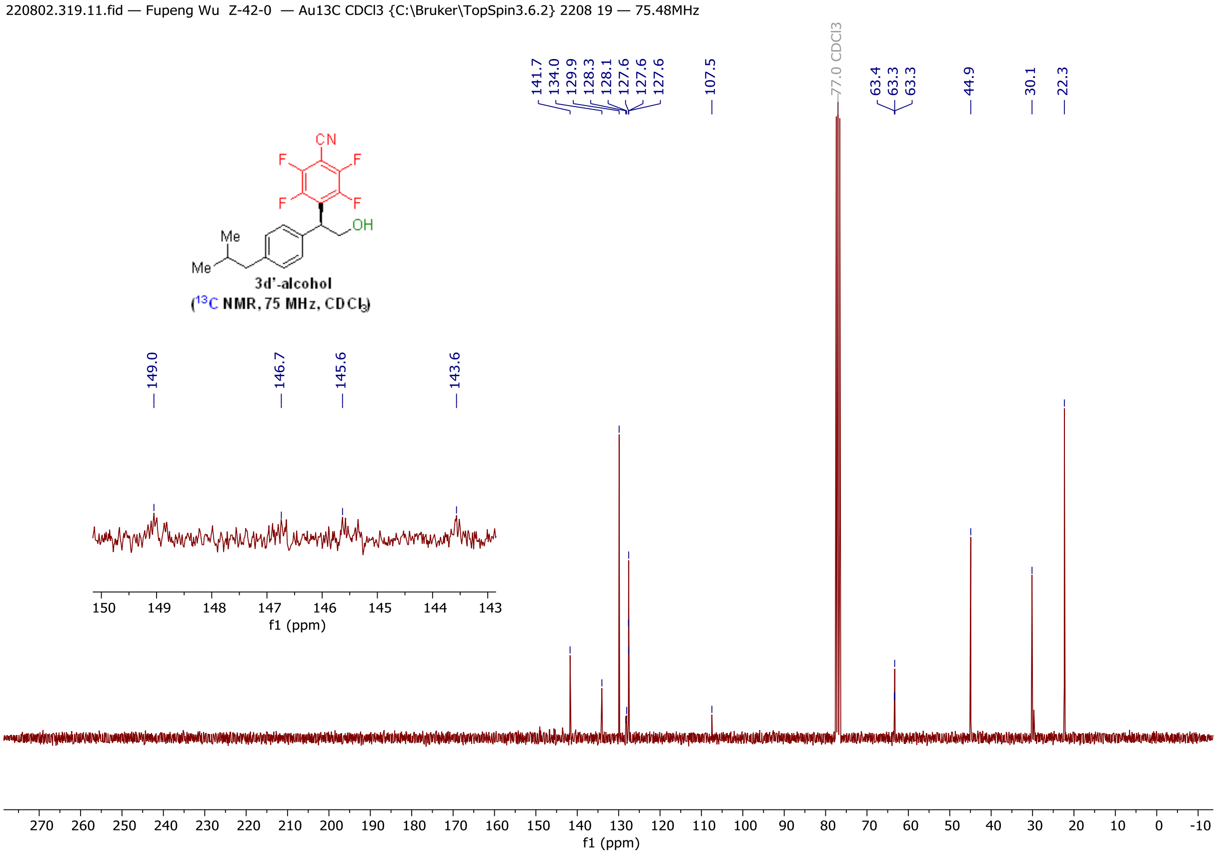

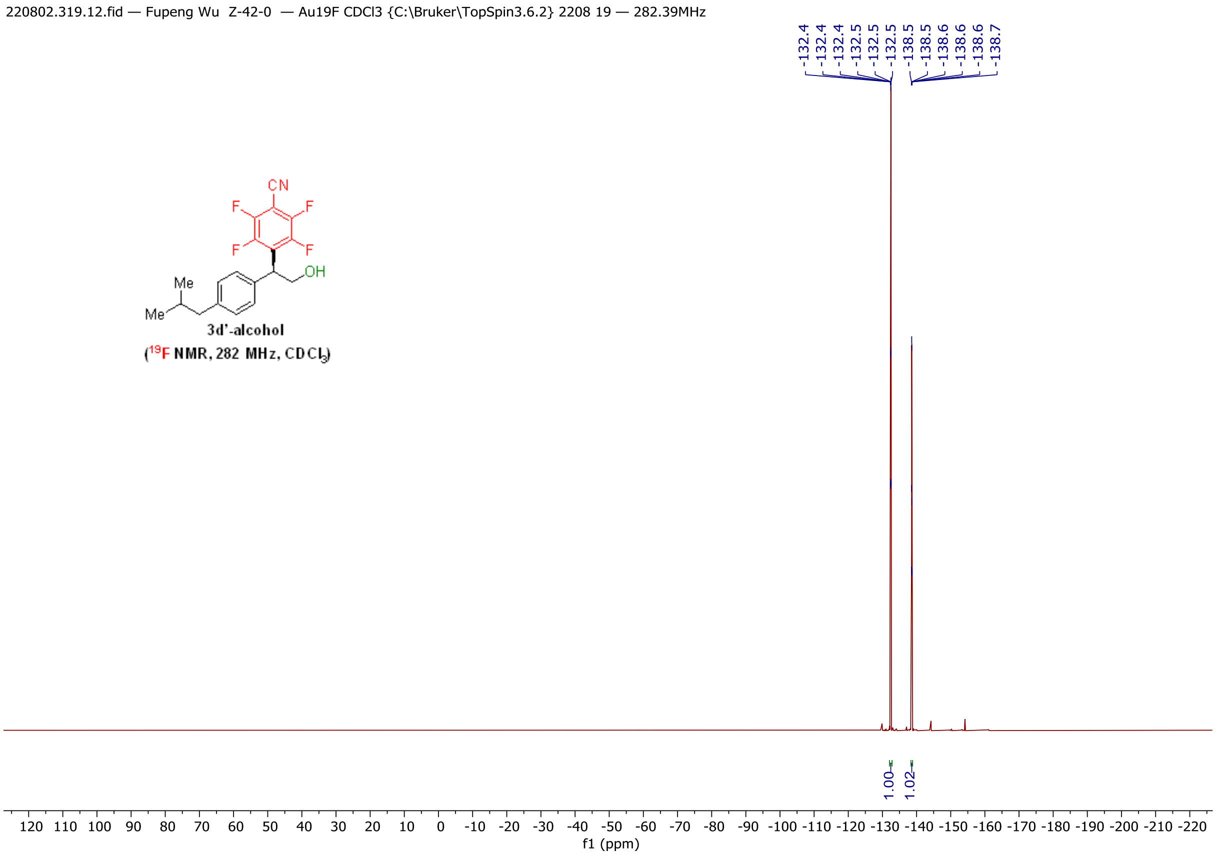
220802.319.12.fid — Fupeng Wu Z-42-0 — Au19F CDCl3 {C:\Bruker\TopSpin3.6.2} 2208 19 — 282.39MHz
-132.4
-132.4
-132.4
-132.5
-132.5
-132.5
-138.5
-138.5
-138.6
-138.6
-138.6
-138.7
CN
F
F
F
F
OH
Me
Me
3d'-alcohol
(19F NMR, 282 MHz, CDCl3)
1.00
1.02
120 110 100 90 80 70 60 50 40 30 20 10 0 -10 -20 -30 -40 -50 -60 -70 -80 -90 -100 -110 -120 -130 -140 -150 -160 -170 -180 -190 -200 -210 -220
f1 (ppm)

NMR Spectra of **3r'-alcohol**

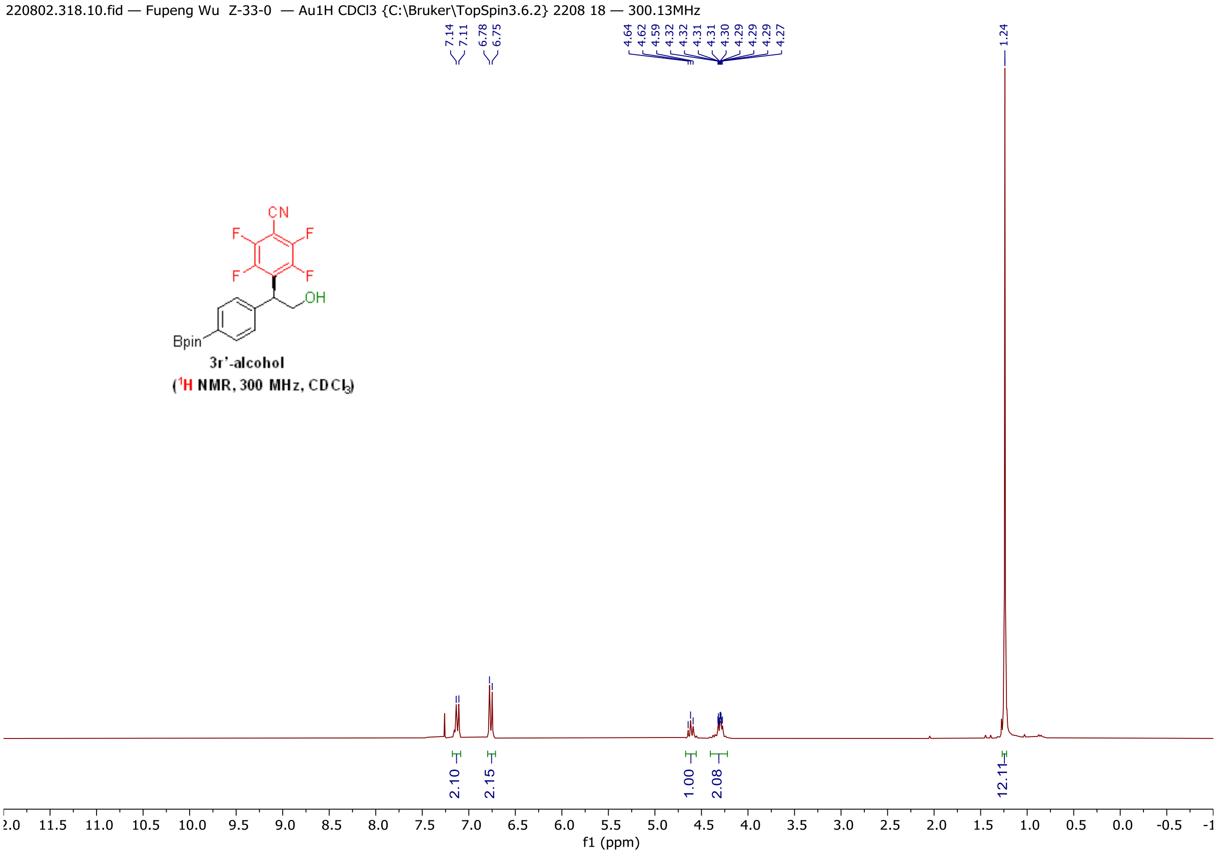

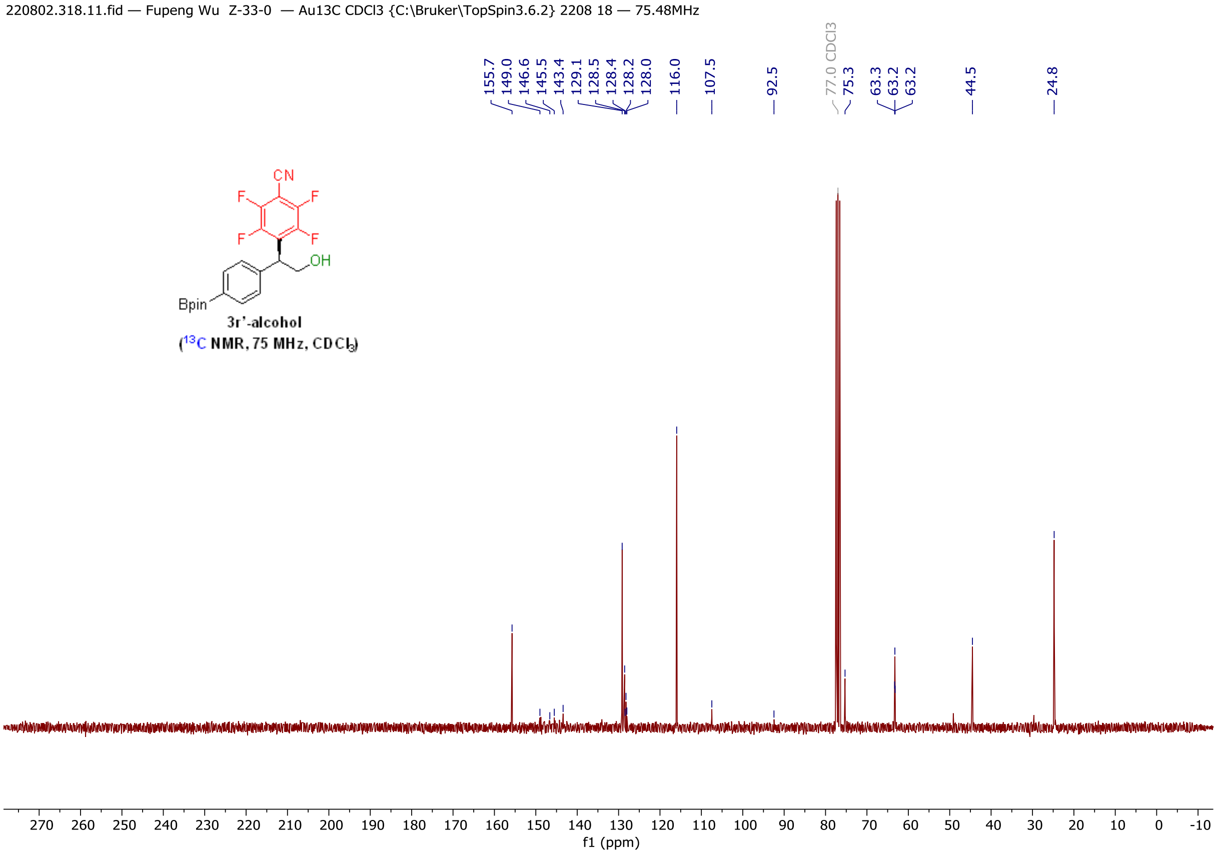

220802.318.12.fid — Fupeng Wu Z-33-0 — Au19F CDCl3 {C:\Bruker\TopSpin3.6.2} 2208 18 — 282.39MHz

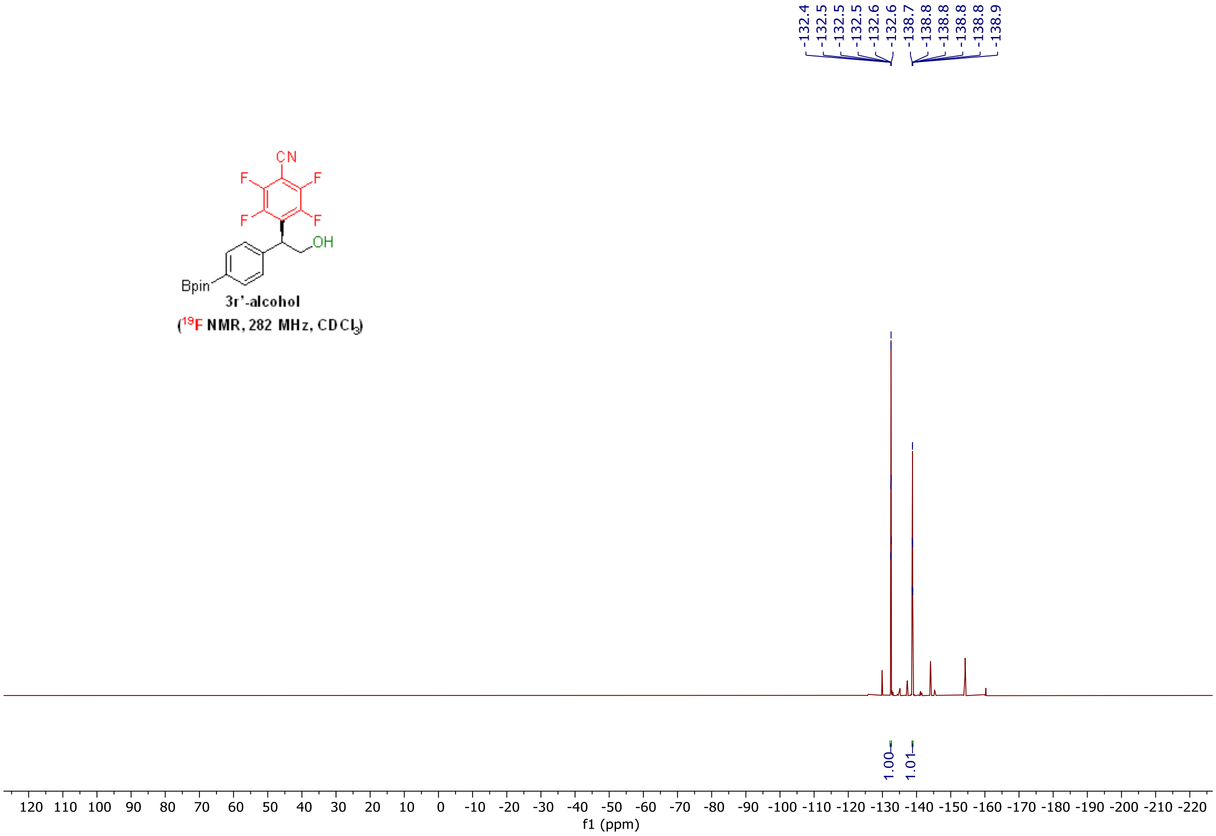

NMR Spectra of **3z’-alcohol**

220802.320.10.fid — Fupeng Wu Z-52-0 — Au1H CDCl3 {C:\Bruker\TopSpin3.6.2} 2208 20 — 300.13MHz

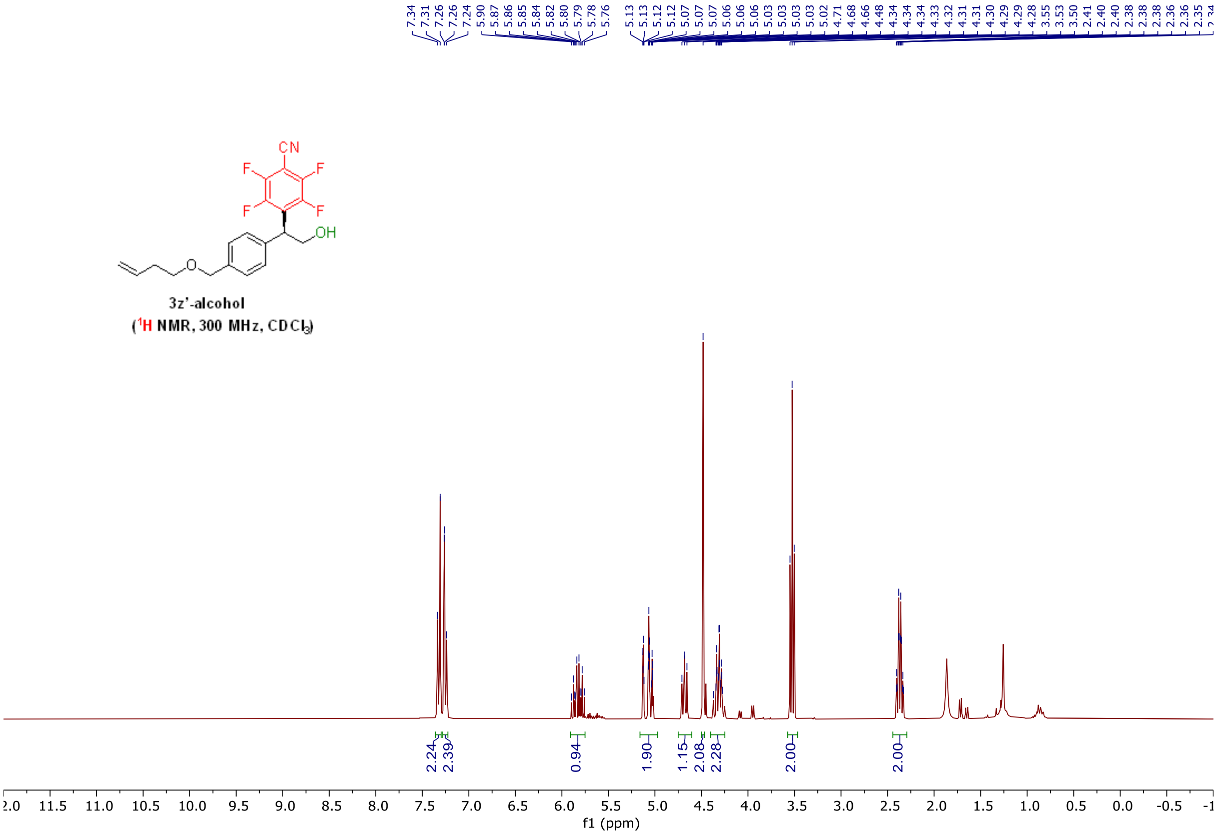

220802.320.11.fid — Fupeng Wu Z-52-0 — Au13C CDCl3 {C:\Bruker\TopSpin3.6.2} 2208 20 — 75.48MHz

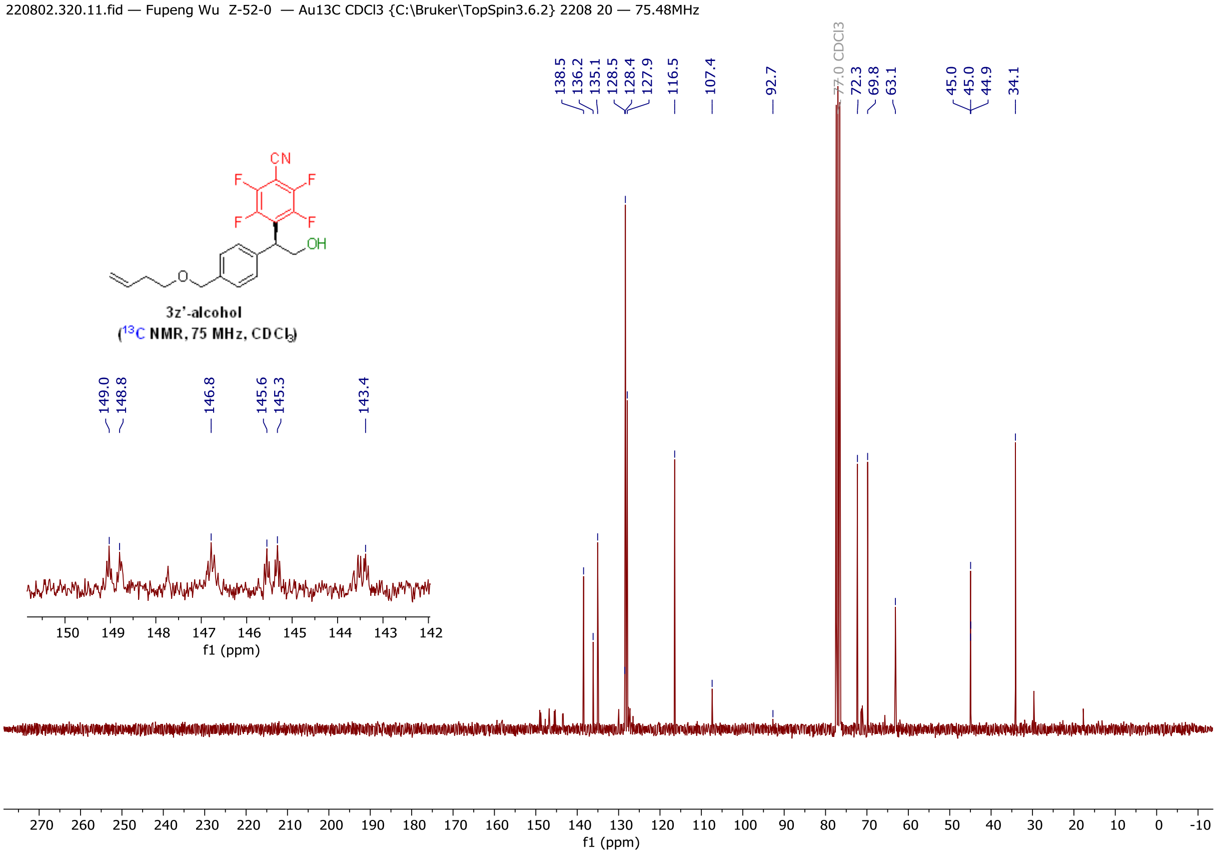

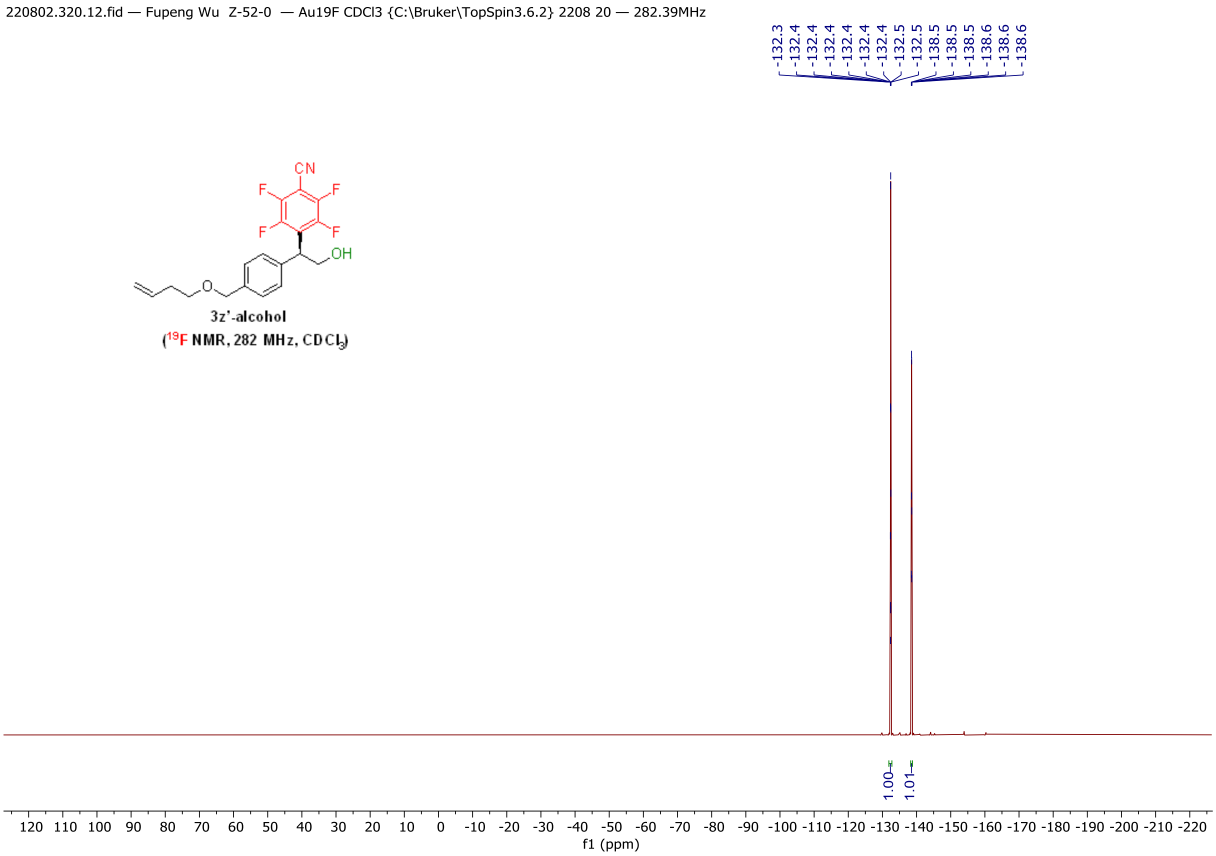
220802.320.12.fid — Fupeng Wu Z-52-0 — Au19F CDCl3 {C:\Bruker\TopSpin3.6.2} 2208 20 — 282.39MHz
-132.3
-132.4
-132.4
-132.4
-132.4
-132.4
-132.4
-132.5
-132.5
-138.5
-138.5
-138.5
-138.6
-138.6
-138.6
CN
F
F
F
F
OH
O
3z'-alcohol
(19F NMR, 282 MHz, CDCl3)
1.00
1.01
120 110 100 90 80 70 60 50 40 30 20 10 0 -10 -20 -30 -40 -50 -60 -70 -80 -90 -100 -110 -120 -130 -140 -150 -160 -170 -180 -190 -200 -210 -220
f1 (ppm)

9. Enantiomeric ratio monitoring

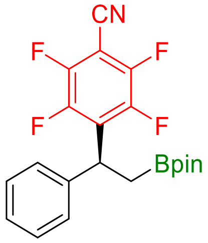

(*S*)-**3a'**

32.0 mg, 40%, 99% ee.

[α]$^{24}_{D}$ = -52.6 (c = 0.30, $CHCl_3$).

The enantiomeric excess was determined by AD-H, column, heptane/EtOH = 99:1 v/v, *v* = 0.5 mL/min, λ= 229 nm, t_R (minor) = 12.396 min, t_R (major) = 10.246 min.

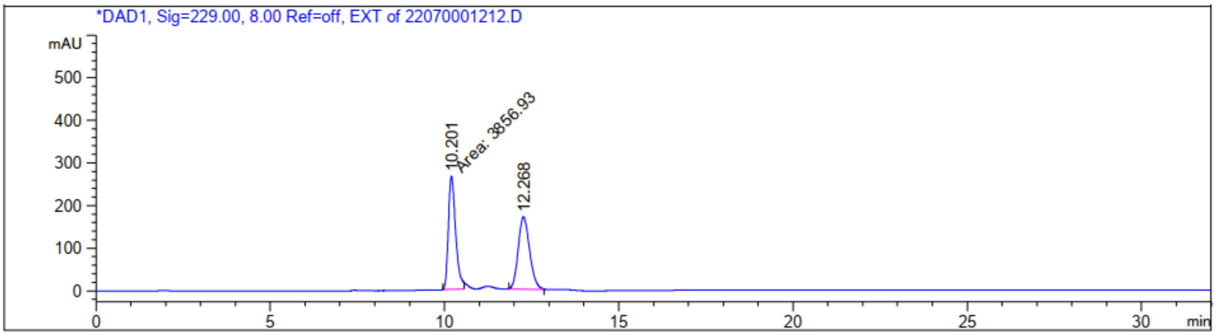

Peak #	RetTime [min]	Type	Width [min]	Area [mAU*s]	Height [mAU]	Area %
1	10.201	MF	0.2410	3856.92676	266.75510	49.9751
2	12.268	BB	0.3516	3860.77515	169.56587	50.0249

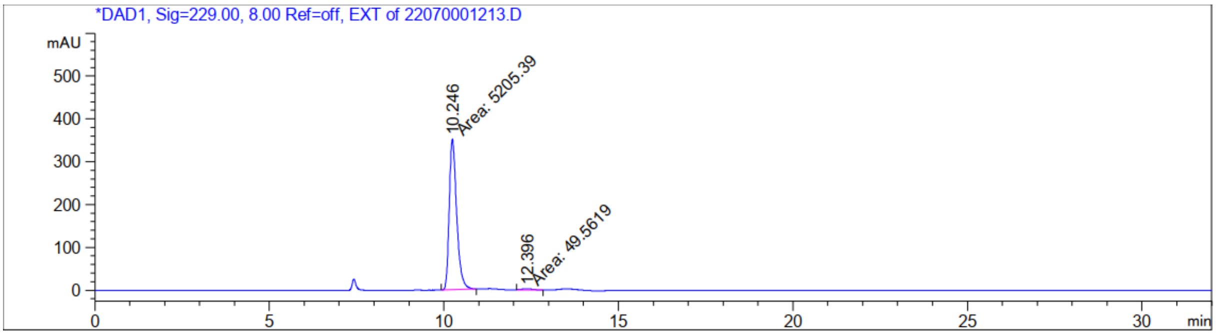

Peak #	RetTime [min]	Type	Width [min]	Area [mAU*s]	Height [mAU]	Area %
1	10.246	MM	0.2470	5205.39063	351.28882	99.0569
2	12.396	MM	0.3612	49.56193	2.28697	0.9431

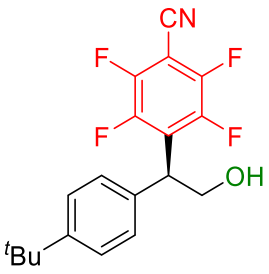

(*S*)-**3c'-alcohol**

3c': 47.8 mg, 52% yield.

3c'-alcohol: 98% ee.

^{1}H NMR (300 MHz, CDCl$_3$) δ 7.40 – 7.33 (m, 2H), 7.25 – 7.18 (m, 2H), 4.69 (t, *J* = 8.1 Hz, 1H), 4.35 (ddt, *J* = 7.7, 2.9, 1.4 Hz, 2H), 1.30 (s, 9H). **^{13}C NMR (75 MHz, CDCl$_3$)** δ 151.1, 147.2 (dm, *J* = 262.2 Hz), 145.1 (dm, *J* = 247.7 Hz), 133.7, 128.1 (t, *J* = 16.2 Hz), 127.6 (t, *J* = 1.7 Hz), 126.1, 107.5 (t, *J* = 3.7 Hz), 92.6, 63.2, 44.9 (t, *J* = 1.7 Hz), 34.5, 31.2. **^{19}F NMR (282 MHz, CDCl$_3$)** δ -132.18 – -132.80 (m), -138.52 (td, *J* = 16.4, 7.2 Hz). **$[\alpha]_D^{24}$** = -9.2 (c = 0.24, CHCl$_3$). The enantiomeric excess was determined by Reprosil column, heptane/EtOH = 98:2 v/v, *v* = 0.4 mL/min, λ= 237 nm, t_R (minor) = 21.415 min, t_R (major) = 23.446 min.

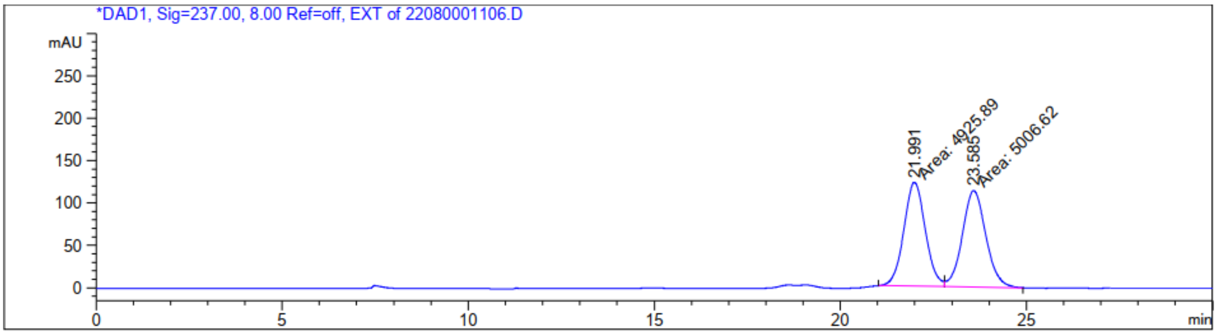

Peak #	RetTime [min]	Type	Width [min]	Area [mAU*s]	Height [mAU]	Area %
1	21.991	MF	0.6706	4925.89404	122.41784	49.5936
2	23.585	FM	0.7334	5006.62305	113.77729	50.4064

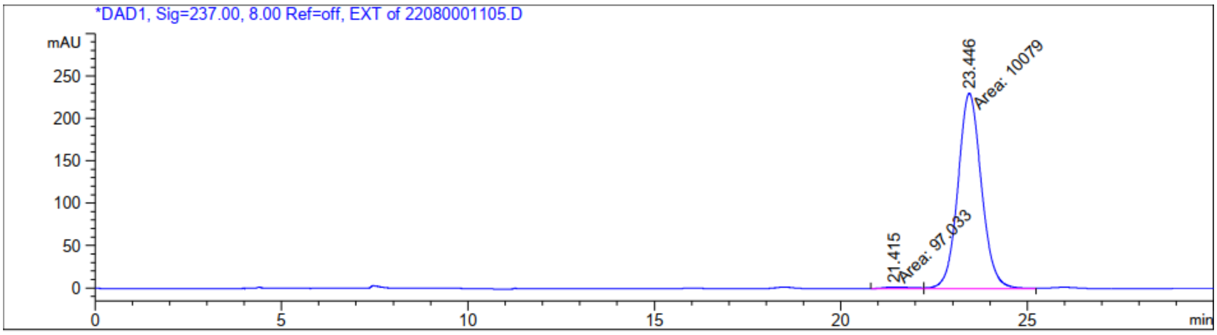

Peak #	RetTime [min]	Type	Width [min]	Area [mAU*s]	Height [mAU]	Area %
1	21.415	MF	0.8698	97.03295	1.85920	0.9535
2	23.446	FM	0.7298	1.00790e4	230.18336	99.0465

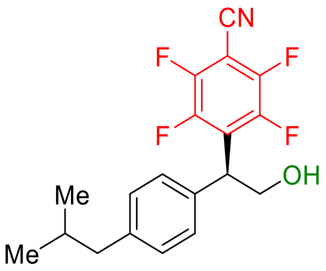

(*S*)-**3d'-alcohol**

3d': 35.5 mg, 39%.

3d'-alcohol: 96% ee.

^{1}H NMR (300 MHz, $CDCl_3$) δ 7.19 (d, *J* = 8.0 Hz, 2H), 7.11 (d, *J* = 8.4 Hz, 2H), 4.68 (t, *J* = 8.1 Hz, 1H), 4.34 (ddt, *J* = 7.7, 2.1, 1.3 Hz, 2H), 2.44 (d, *J* = 7.2 Hz, 2H), 1.91 – 1.75 (m, 1H), 0.90 (s, 3H), 0.87 (s, 3H). **^{13}C NMR (75 MHz, $CDCl_3$)** δ 147.3 (dm, *J* = 265.4 Hz), 145.2 (dm, *J* = 239.6 Hz), 141.7, 134.0, 129.9, 128.1 (t, *J* = 16.5 Hz), 127.6 (t, *J* = 1.7 Hz), 107.5 (t, *J* = 3.3 Hz), 63.3 (t, *J* = 4.0 Hz), 44.9, 30.1, 22.3. **^{19}F NMR (282 MHz, $CDCl_3$)** δ -132.08 – -132.81 (m), -138.59 (td, *J* = 16.4, 7.2 Hz). **$[\alpha]_D^{24}$** = -7.2 (c = 0.25, $CHCl_3$).

The enantiomeric excess was determined by AS-H column, heptane/EtOH = 99:1 v/v, *v* = 1.0 mL/min, λ= 210 nm, t_R (minor) = 10.002 min, t_R (major) = 11.499 min.

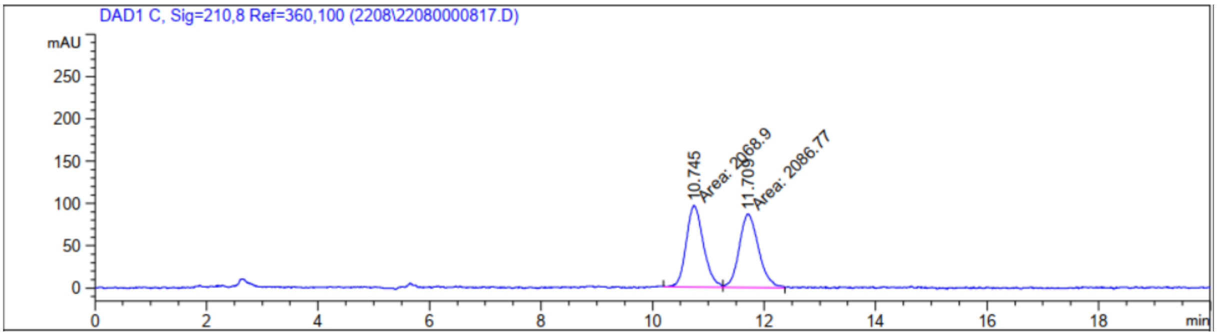

Peak #	RetTime [min]	Type	Width [min]	Area [mAU*s]	Height [mAU]	Area %
1	10.745	MF	0.3581	2068.90405	96.30323	49.7850
2	11.709	FM	0.4012	2086.77368	86.69592	50.2150

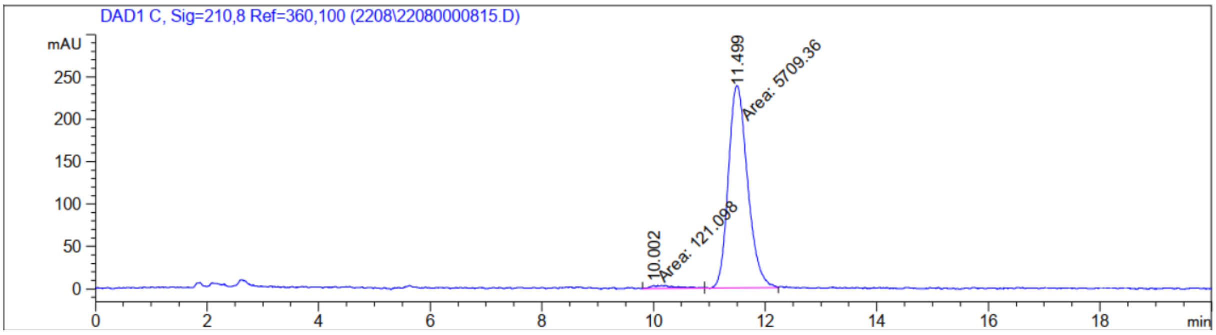

Peak #	RetTime [min]	Type	Width [min]	Area [mAU*s]	Height [mAU]	Area %
1	10.002	MF	0.5342	121.09766	3.77809	2.0770
2	11.499	FM	0.3994	5709.35547	238.24101	97.9230

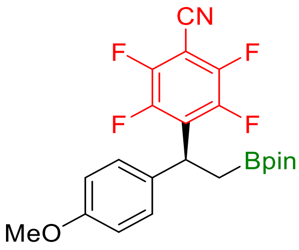

(*S*)-**3e’**

35.1 mg, 40%, 99% ee

[α]$_D^{25}$ = -45.8 (c = 0.19, $CHCl_3$).

The enantiomeric excess was determined by AD-H, column, heptane/EtOH = 99:1 v/v, *v* = 0.5 mL/min, λ= 229 nm, t_R (minor) = 17.779 min, t_R (major) = 13.979 min.

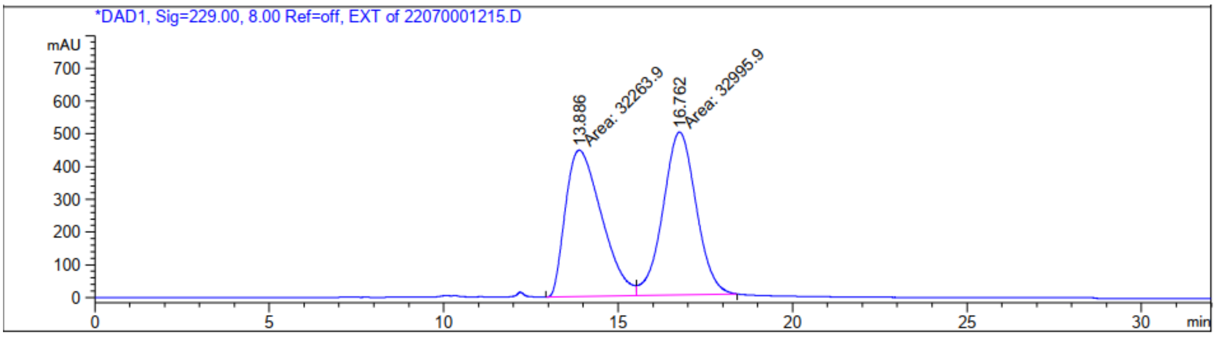

Peak #	RetTime [min]	Type	Width [min]	Area [mAU*s]	Height [mAU]	Area %
1	13.886	MF	1.2016	3.22639e4	447.52405	49.4391
2	16.762	FM	1.1048	3.29959e4	497.78049	50.5609

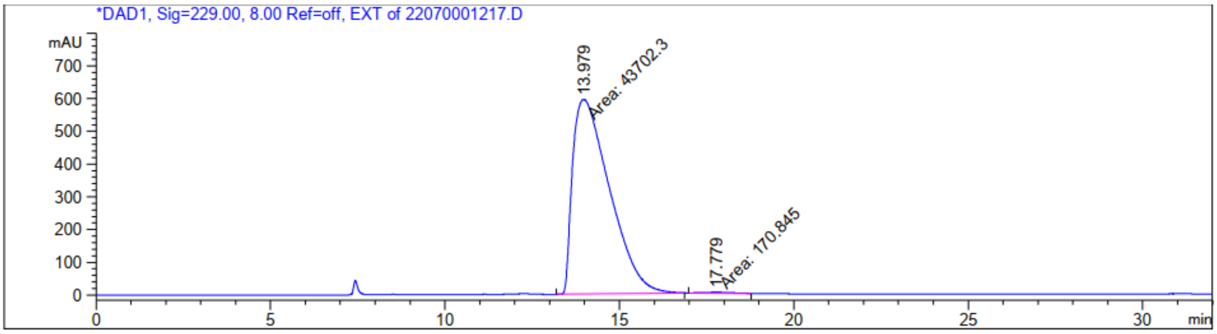

Peak #	RetTime [min]	Type	Width [min]	Area [mAU*s]	Height [mAU]	Area %
1	13.979	MM	1.2260	4.37023e4	594.10181	99.6106
2	17.779	MM	0.9810	170.84515	2.90263	0.3894

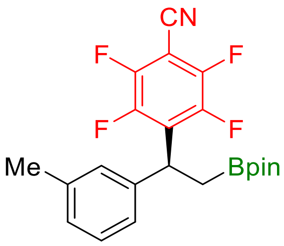

(*S*)-**3i'**

35.7 mg, 43%, 99% ee.

[α]$_D^{25}$ = -45.0 (c = 0.40, $CHCl_3$).

The enantiomeric excess was determined by AD-H, column, heptane/EtOH = 99:1 v/v, *v* = 0.5 mL/min, λ= 229 nm, t_R (minor) = 10.365 min, t_R (major) = 9.400 min.

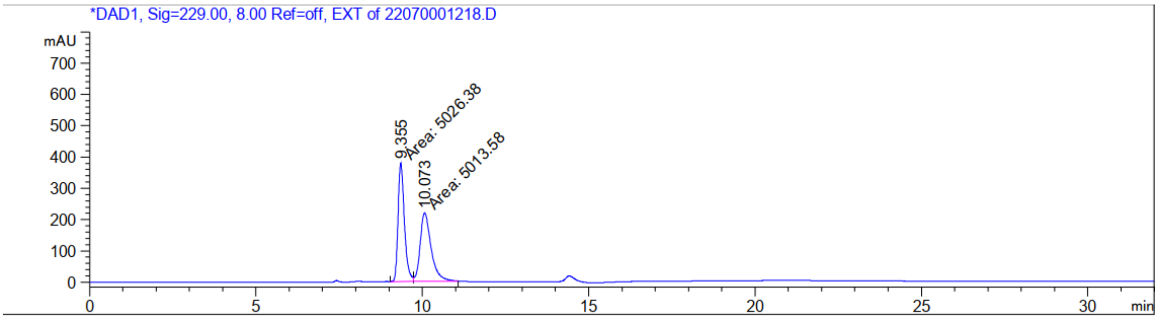

Peak #	RetTime [min]	Type	Width [min]	Area [mAU*s]	Height [mAU]	Area %
1	9.355	MF	0.2214	5026.38037	378.40521	50.0638
2	10.073	FM	0.3813	5013.57813	219.15622	49.9362

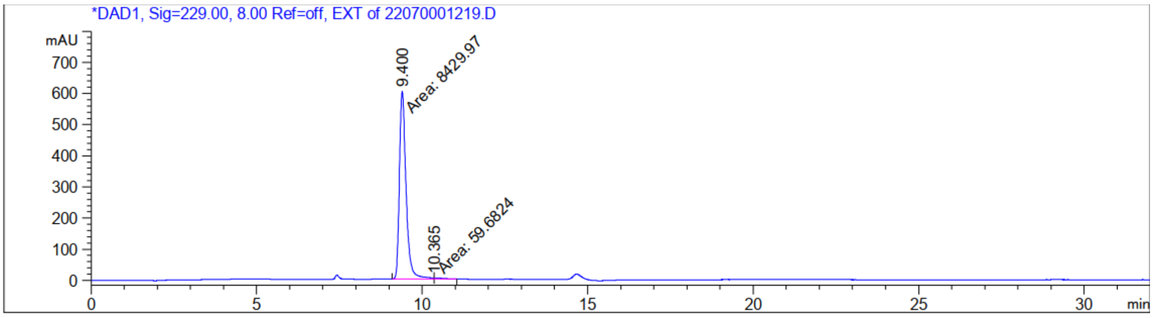

Peak #	RetTime [min]	Type	Width [min]	Area [mAU*s]	Height [mAU]	Area %
1	9.400	MF	0.2324	8429.97461	604.43665	99.2970
2	10.365	FM	0.3289	59.68241	3.02467	0.7030

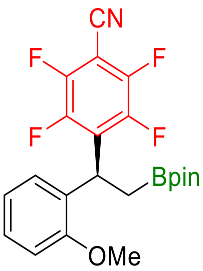

(*S*)-**3m'**

35.4 mg, 41%, 92% ee.

[α]$_D^{26}$ = -18.6 (c = 0.42, $CHCl_3$).

The enantiomeric excess was determined by Cellulose1 column, heptane/EtOH = 99.5:0.5 v/v, *v* = 1.0 mL/min, λ= 229 nm, t_R (minor) = 3.9000 min, t_R (major) = 4.471 min.

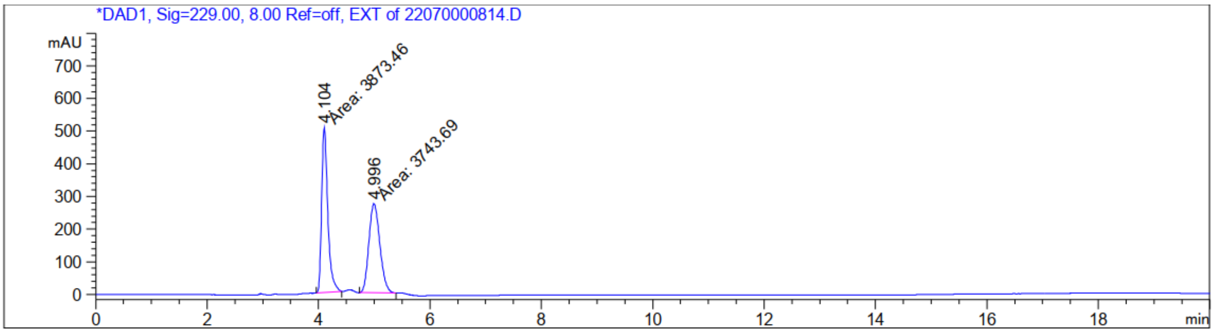

Peak #	RetTime [min]	Type	Width [min]	Area [mAU*s]	Height [mAU]	Area %
1	4.104	MM	0.1279	3873.46265	504.84290	50.8518
2	4.996	MM	0.2288	3743.69458	272.73911	49.1482

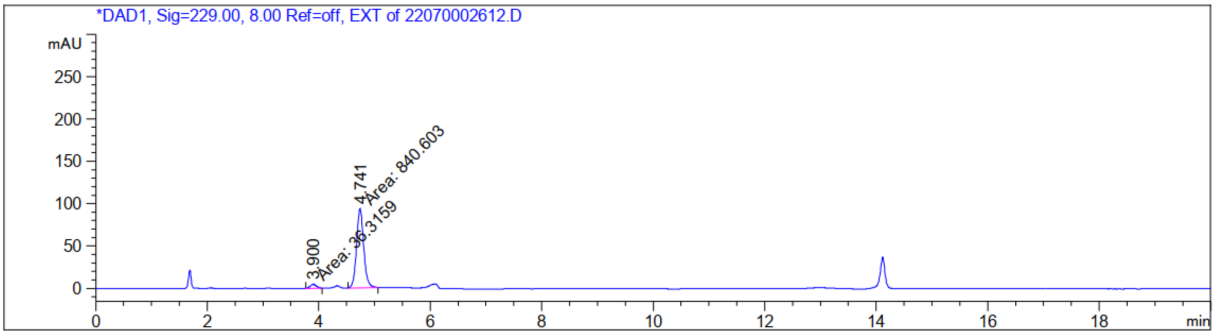

Peak #	RetTime [min]	Type	Width [min]	Area [mAU*s]	Height [mAU]	Area %
1	3.900	MM	0.1267	36.31593	4.77859	4.1413
2	4.741	MM	0.1495	840.60321	93.70737	95.8587

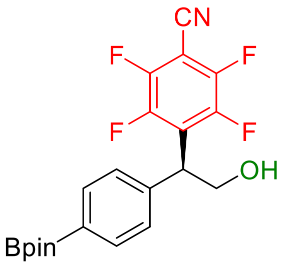

(*S*)-**3r'-alcohol**

3r':63.5 mg, 60%.

3r'-alcohol:96% ee.

^{1}H NMR (300 MHz, $CDCl_3$) δ 7.12 (d, *J* = 8.7 Hz, 2H), 6.76 (d, *J* = 8.7 Hz, 2H), 4.62 (t, *J* = 8.1 Hz, 1H), 4.30 (ddt, *J* = 6.0, 4.5, 2.3 Hz, 2H), 1.24 (s, 12H). **^{13}C NMR (75 MHz, $CDCl_3$)** δ 155.7, 147.3 (dm, *J* = 261.5 Hz), 145.0 (dm, *J* = 241.3 Hz), 129.1, 128.5, 128.1 (t, *J* = 15.9 Hz), 116.0, 107.5, 92.5, 75.3, 63.3 (t, *J* = 4.1 Hz), 44.5, 24.8. **^{19}F NMR (282 MHz, $CDCl_3$)** δ -132.22 – -132.67 (m), -138.80 (td, *J* = 16.4, 7.2 Hz). **$[\alpha]_D^{24}$** = -12.3 (c = 0.21, $CHCl_3$).

The enantiomeric excess was determined by OJ-H column, heptane/EtOH = 90:10 v/v, *v* = 1.0 mL/min, λ= 210 nm, t_R (minor) = 26.461 min, t_R (major) = 20.722 min.

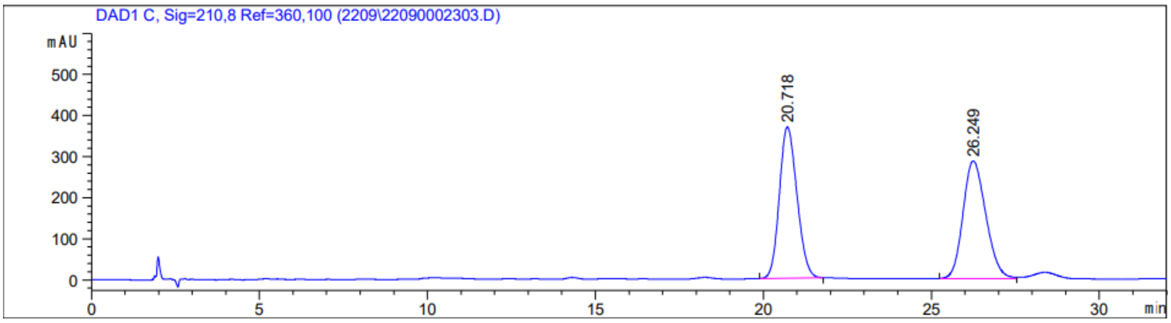

Peak #	RetTime [min]	Type	Width [min]	Area [mAU*s]	Height [mAU]	Area %
1	20.718	BB	0.5677	1.34422e4	368.35800	49.7579
2	26.249	BB	0.7302	1.35730e4	286.78946	50.2421

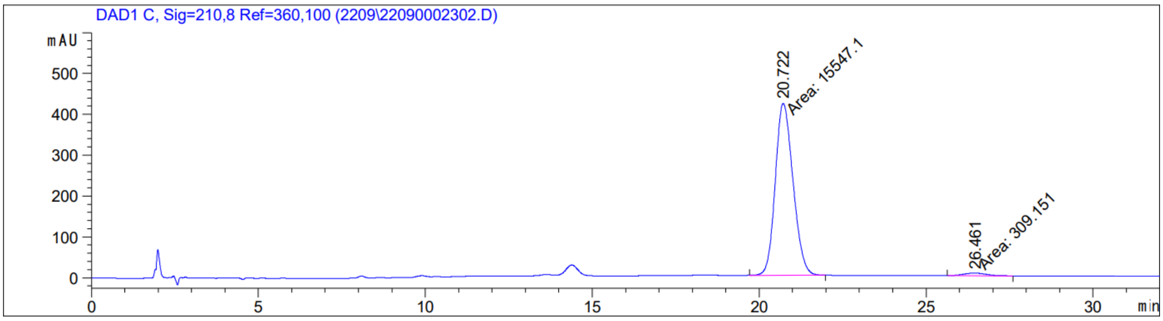

Peak #	RetTime [min]	Type	Width [min]	Area [mAU*s]	Height [mAU]	Area %
1	20.722	MM	0.6155	1.55471e4	420.96844	98.0503
2	26.461	MM	0.7798	309.15054	6.60723	1.9497

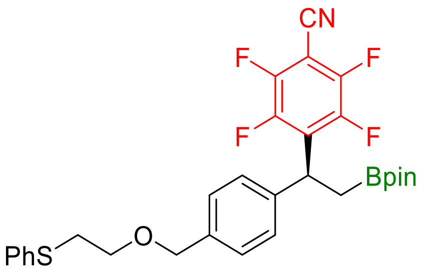

(S)-**3v’**

62.2 mg, 55%, 98% ee.

[α]$_D^{26}$ = -37.4 (c = 0.42, $CHCl_3$).

The enantiomeric excess was determined by Cellulose1 column, heptane/EtOH = 99.5:0.5 v/v, v = 1.0 mL/min, λ= 229 nm, t_R (minor) = 16.959 min, t_R (major) = 15.220 min.

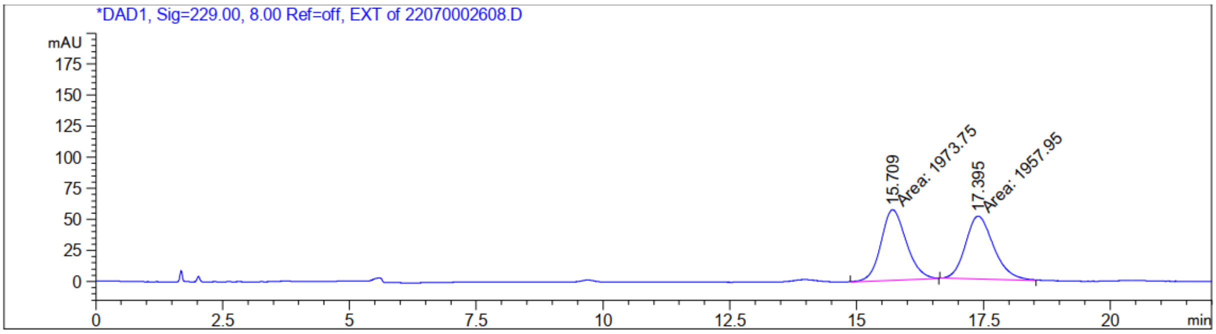

Peak #	RetTime [min]	Type	Width [min]	Area [mAU*s]	Height [mAU]	Area %
1	15.709	MM	0.5790	1973.75171	56.81250	50.2009
2	17.395	MM	0.6444	1957.95496	50.64277	49.7991

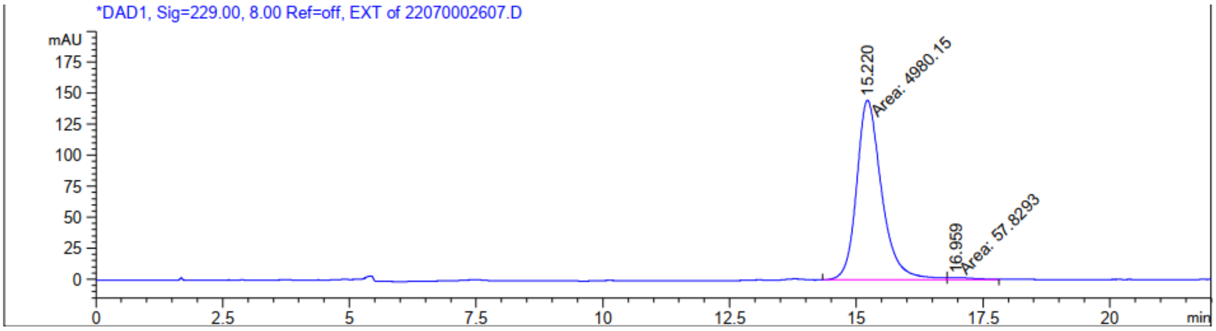

Peak #	RetTime [min]	Type	Width [min]	Area [mAU*s]	Height [mAU]	Area %
1	15.220	MF	0.5735	4980.15137	144.72643	98.8521
2	16.959	FM	0.5725	57.82930	1.68342	1.1479

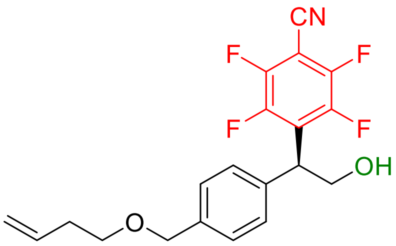

(*S*)-**3z'-alcohol**

3z':55.1 mg, 56%.

3z'-alcohol: 97% ee

^{1}H NMR (300 MHz, $CDCl_3$) δ 7.32 (d, *J* = 8.6 Hz, 2H), 7.28 – 7.22 (m, 2H), 5.83 (ddt, *J* = 17.1, 10.3, 6.7 Hz, 1H), 5.16 – 4.97 (m, 2H), 4.75 – 4.60 (m, 1H), 4.48 (s, 2H), 4.31 (ddt, *J* = 7.9, 6.6, 1.5 Hz, 2H), 3.53 (t, *J* = 6.7 Hz, 2H), 2.37 (qt, *J* = 6.7, 1.4 Hz, 2H). **^{13}C NMR (75 MHz, $CDCl_3$)** δ 147.2 (dm, *J* = 261.9 Hz), 145.1 (dm, *J* = 248.4 Hz), 138.5, 136.2, 135.1, 128.5, 128.4, 127.9, 116.5, 107.4 (t, *J* = 3.6 Hz), 92.7, 72.3, 69.8, 63.1, 45.0 (t, *J* = 1.9 Hz), 34.1. **^{19}F NMR (282 MHz, $CDCl_3$)** δ -131.89 – -132.78 (m), -138.55 (td, *J* = 16.4, 7.2 Hz). **$[\alpha]_D^{24}$** = -11.7 (c = 0.29, $CHCl_3$).

The enantiomeric excess was determined by Reprosil column, heptane/EtOH = 98:2 v/v, *v* = 0.4 mL/min, λ= 237 nm, t_R (minor) = 15.534 min, t_R (major) = 17.146 min.

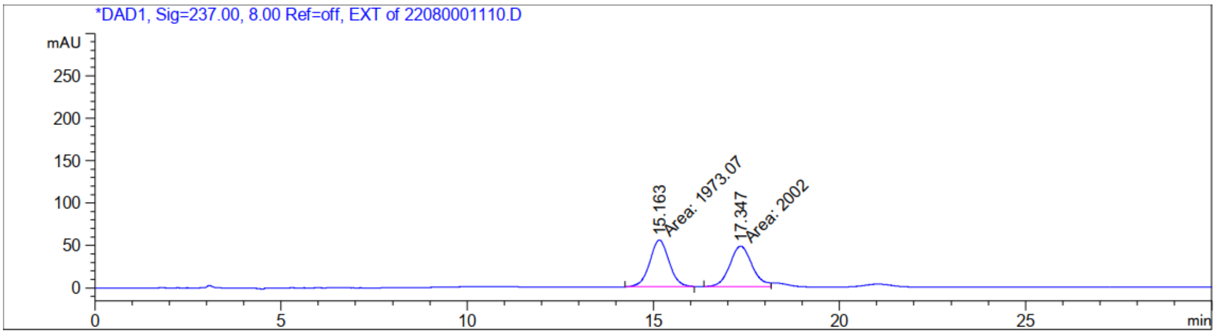

Peak #	RetTime [min]	Type	Width [min]	Area [mAU*s]	Height [mAU]	Area %
1	15.163	MM	0.5993	1973.07483	54.87591	49.6362
2	17.347	MM	0.6966	2001.99536	47.90133	50.3638

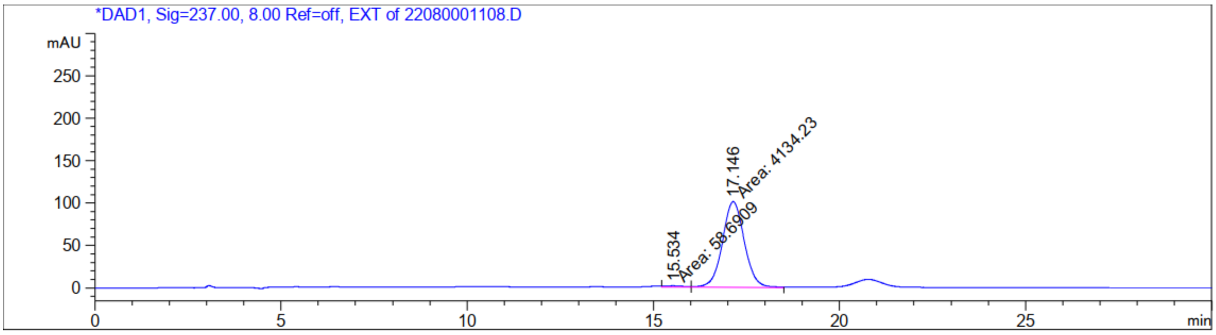

Peak #	RetTime [min]	Type	Width [min]	Area [mAU*s]	Height [mAU]	Area %
1	15.534	MF	0.5884	58.69086	1.66249	1.3998
2	17.146	FM	0.6821	4134.22559	101.01692	98.6002
